# Uncovering Dynamic Brain Reconfiguration in MEG Working Memory *n*-Back Task Using Topological Data Analysis

**DOI:** 10.3390/brainsci9060144

**Published:** 2019-06-19

**Authors:** Ali Nabi Duman, Ahmet Emin Tatar, Harun Pirim

**Affiliations:** 1 Department of Mathematics and Statistics, King Fahd University of Petroleum and Minerals, Dhahran 31261, Saudi Arabia; atatar@kfupm.edu.sa; 2 Department of Systems Engineering, King Fahd University of Petroleum and Minerals, Dhahran 31261, Saudi Arabia; harunpirim@kfupm.edu.sa

**Keywords:** working memory, topological data analysis, dynamic functional connectivity, mapper, MEG

## Abstract

The increasing availability of high temporal resolution neuroimaging data has increased the efforts to understand the dynamics of neural functions. Until recently, there are few studies on generative models supporting classification and prediction of neural systems compared to the description of the architecture. However, the requirement of collapsing data spatially and temporally in the state-of-the art methods to analyze functional magnetic resonance imaging (fMRI), electroencephalogram (EEG) and magnetoencephalography (MEG) data cause loss of important information. In this study, we addressed this issue using a topological data analysis (TDA) method, called Mapper, which visualizes evolving patterns of brain activity as a mathematical graph. Accordingly, we analyzed preprocessed MEG data of 83 subjects from Human Connectome Project (HCP) collected during working memory *n*-back task. We examined variation in the dynamics of the brain states with the Mapper graphs, and to determine how this variation relates to measures such as response time and performance. The application of the Mapper method to MEG data detected a novel neuroimaging marker that explained the performance of the participants along with the ground truth of response time. In addition, TDA enabled us to distinguish two task-positive brain activations during 0-back and 2-back tasks, which is hard to detect with the other pipelines that require collapsing the data in the spatial and temporal domain. Further, the Mapper graphs of the individuals also revealed one large group in the middle of the stimulus detecting the high engagement in the brain with fine temporal resolution, which could contribute to increase spatiotemporal resolution by merging different imaging modalities. Hence, our work provides another evidence to the effectiveness of the TDA methods for extracting subtle dynamic properties of high temporal resolution MEG data without the temporal and spatial collapse.

## 1. Introduction

Understanding relations or interactions that link many elements of neurobiological systems is highly important area of research due to the recent development of imaging methods and the increasing availability of neural data. Several analytical methods to explore the time-evolving patterns of interaction between the regions of the brain are proposed to extract valuable insight from mesoscale neuroimaging data including functional magnetic resonance imaging (fMRI), electrocorticography (ECoG), magnetoencephalography (MEG), and electroencephalography (EEG). These approaches include the tools from topology and graph theory in mathematics, algorithm development in computer science, statistical mechanics in physics, and systems engineering.

Earlier techniques focusing on the inter-regional functional connectivity (FC) over the entire time scan time successfully investigate modular structure of brain regions in fMRI [1,2,3] and [4,5,6]. However, more recent studies suggest that the assessment of temporal dynamics is a more accurate way in describing cognitive processes as FC patterns may change over time [7,8,9,10,11]. As noted in [12], there are two main approaches to measure dynamic FC. The first one uses multiple successive time points and is mainly used for experiment without trial locking (e.g., resting state). The most abundant method that falls into the category is the sliding windows method MEG/EEG [13,14,15], which is formulated by dividing the whole time interval into sections, where FC is investigated. Other approaches include Hidden Markov Models [16,17] and phase difference derivative [18]. The second one, which is used in multiple task trials, is aggregation across the same time point of multiple trials to generate connectivity dynamics. In any of these pipelines, collapsing the data in temporal or spatial scale may result in loss of important information. For example, the sliding window time intervals reduces the temporal resolution of the raw data. Moreover, choosing a window width is an issue: As too short widows are negatively affected by noise, while the long ones may fail to capture rapid changes in FC. Saggar et al. recently addressed this issue of collapsing data for fMRI using topological data analysis (TDA) based on a method called Mapper [19].

TDA methods (i.e., persistent homology and Mapper) are not new to the neuroimaging community. Some remarkable applications of persistent homology include the detection of intrinsic structure in correlations between neural spike trains [20], analysis of the formation of spatial maps in the hippocampus [21] and characterization of the global architecture of fMRI data [22]. All these application concentrate on the static topological structure of the data and they need to be modified in order to be applied to detect dynamic characteristics.

In this study, we resorted to the Mapper method to investigate the dynamic features of the MEG data during cognition. One advantage of the Mapper over the other unsupervised machine learning (ML) techniques such as independent component analysis (ICA) [23] and clustering via correlation [24] is the compact and easily readable visual presentation of the data preserving the geometric structure. This visual output of Mapper is a graph using a single node for each cluster determined by a filtration function on a data cloud and laying edges between nodes whose corresponding clusters share at least one point. It has recently been applied to biological datasets successfully to extract novel insights such as identification of subpopulations of cancer, identification of genomic biomarkers, disease association, RNA folding, viral evolution, immunology, diabetes, and preclinical spinal cord injury [25,26,27].

Mapper can visualize the complex multivariate relationship of various variables simultaneously as a network diagram. The resulting topological map can be investigated to understand the relationship of each variable across the topological space to identify groups of clustered individuals that can be further probed for specific relationships among outcomes, validation and hypothesis testing. In [28], application of the Mapper to structural MRI data revealed two large subgroups of fragile X syndrome. Through direct visualization of functional and health outcomes, Mapper uncovered interactions between spinal cord injury and co-occurring traumatic brain injury (TBI), as well as detrimental drug effects [26]. In [29], it identified a unique diagnostic subgroup of patients with unfavorable outcome after mild TBI. It is also shown to be useful in defining less heterogeneous clinical subgroups in ADHD [30].

In [19], the authors applied Mapper to fMRI data to investigate the dynamics of overall brain reconfiguration during different cognitive tasks. Even though fMRI has been used to study brain dynamics before due to the maturity and availability of this technology, its temporal resolution is too low to detect subtle changes in brain dynamics [31]. In fMRI, neural activation is indirectly measured via local changes in the level of blood oxygenation that makes harder to collect real-time information of neural activities. In our study, we hence applied Mapper to a high temporal resolution MEG dataset from 83 individuals who performed working memory *n*-back task. With MEG, the sequence of neural events can be readily tracked with millisecond resolution. Hence, it is ideal for investigating the cognitive tasks that involve rapid decision making as it can detect directly the fast fluctuations in neuronal coherence. It is therefore natural to expect temporally more subtle biomarkers when applying Mapper pipeline to a MEG dataset rather than a fMRI dataset of the same cognitive task.

Here, we suggest an alternative visualization of MEG dataset from Human Connectome Project (HCP) using the Mapper algorithm to get new insights from the brain patterns dynamics. The nature of Mapper, which represents similar data points closer than dissimilar ones in a mathematical graph, enabled us to visually trace the brain states during the 0-back and 2-back working memory tasks. To show efficacy of our method, we tested the following hypotheses:

- Application of the Mapper method to MEG data would detect novel neuroimaging marker that explains the performance of the participants along with the ground truth of response time.
- TDA would distinguish two task-positive brain activations during 0-back and 2-back tasks, which are hard to detect with other pipelines due to the low temporal resolution of the imaging modality and the collapse of the data in the spatial and temporal domain.
- The Mapper graphs of the individuals would reveal one large group in the middle of the stimulus detecting the high engagement in the brain with fine temporal resolution. Hence, our pipeline with other imaging modalities (e.g., fMRI) could increase the spatiotemporal resolution in the analysis of brain dynamics.

## 2. Methods

### 2.1. Subjects and Data

We used the human non-invasive resting state and task Magnetoencephalography (MEG) dataset. It is publicly available from the Human Connectome Project (HCP) consortium [32]. It was acquired on a Magnes 3600 MEG (4D NeuroImaging, San Diego, USA) with 248 magnetometers. The *Working memory* data are available for a subset of 89 subjects (mean 28.7 years, range 22–35, 41 female/48 male) with two sessions per task. Each task has a similar or identical experimental design to the corresponding task acquired during fMRI imaging. In working memory task, people have to remember the occurrence of a *n*-back previously shown item (with n=0 and 2) with the items being either tools or faces [32]. Data are segmented to the onset of the non-target item (WM task).

We used the parcellated results (using Yeo et al.’s (2011) 17-network parcellation [33]) of the srcavgdics pipeline on the source-reconstructed (megconnectome pipeline v3) Working Memory data averaged event-related and time-frequency responses created by the tmegpreproc pipeline. The data created by the tmegpreproc pipeline have frequency 512. More information on these pipelines can be found in [32].

To these data, we also added subjects’ accuracies from 0-back and 2-back tests and median reaction time for these tests. If one of these pieces of information were missing for a subject, we excluded it from the dataset.

### 2.2. Topological Data Analysis: Mapper

The TDA-based Mapper method is considered as an unsupervised machine learning technique to analyze high-dimensional data. Unlike the supervised methods such as support vector machines (SVM) that compare predefined groups, it is used to discover previously unknown groups within a dataset. Mapper algorithm returns a compact representation in the shape of a graph which preserves the geometric properties of the underlying dataset; hence, it helps to point out complex multivariate features of the input data in an unbiased and data-driven manner. Moreover, Mapper can apply a set of dimension reduction techniques (e.g., principal component analysis (PCA), singular value decomposition (SVD), and stochastic neighborhood estimation (SNE)) to the data without the necessity of prior categorization or extensive cleaning, which might result in the loss of salient information.

We now explain the Mapper pipeline that we used to produce the Mapper graph. One can refer to [34,35,36] for theoretical details of the TDA-based methods. As with many other machine learning techniques, it starts with a n×m data matrix whose rows and columns correspond to the variables of interest (e.g., time points) and their observed values (e.g., voxel). One can consider the matrix as a point cloud consisting of *m* points in *n*-dimensional space. The first step of the Mapper is to assign values to the data points; these values, called *filter values*, can be the output of a density estimator, measure of centrality, PCA, SVD, stochastic neighborhood estimation (SNE), etc. In the second step, Mapper divides the filter space into the overlapping bins. The shape of the bins (e.g., interval, rectangle, rectangular prism, etc.) depends on the filter values. For example, if the range of the filter values is the interval [0,9], one can choose the bins as [0,3], [2,5], [4,7] and [6,9]. The purpose of the filter values is to collapse high dimensional data to a single data point and detect the meaningful properties of the data. Once the filter values and overlapping bins are determined, Mapper distributes the data points into the point sets based on their filter values. In the last step, Mapper clusters the data points in each set using a chosen distance metric, which can be any similarity function such as correlation, Euclidean, $L_{1}$ or $L_{\infty }$ metrics along with a clustering algorithm such as single linkage clustering and density based clustering. The outcome of Mapper is a combinatorial graph whose nodes are the clusters and edges are between the nodes which share one or more data points. The resulting graph captures the geometric structure of the dataset indicating the spectra and clusters in a compact manner.

We note that the Mapper algorithm has several parameters: the number of intervals, the overlapping percentage, the distance, and the clustering algorithm. Increasing the number of intervals increases the complexity of the final visualization as the number nodes increase. As a result, each node contains only the highly similar data points. On the other hand, the change in the overlapping percentage effects the number of edges in the final graph. An increase in the percentage results in an increase in the number of edges. Once the network is extracted, the parameters can be tuned to focus the network similar to focusing a microscope on an image.

Mapper graphs can be investigated by focusing its structures at different scales, which include the local scale analysis of nodes (or edges), the intermediate (mesoscale) structures such as core-periphery and modality [19] and the global level of summary statistics. In this study, we concentrated on local level analysis. We detected the time points where the brain state returns to the baseline and statistically compared the result with ground truth obtained from HCP database.

### 2.3. Constructing the Mapper Graph for MEG Dataset

We applied the Mapper algorithm to the MEG working memory task data from HCP. Our goal was to trace the brain activation patterns during the 0-back and 2-back memory tests of each participant.

**Figure 1:**
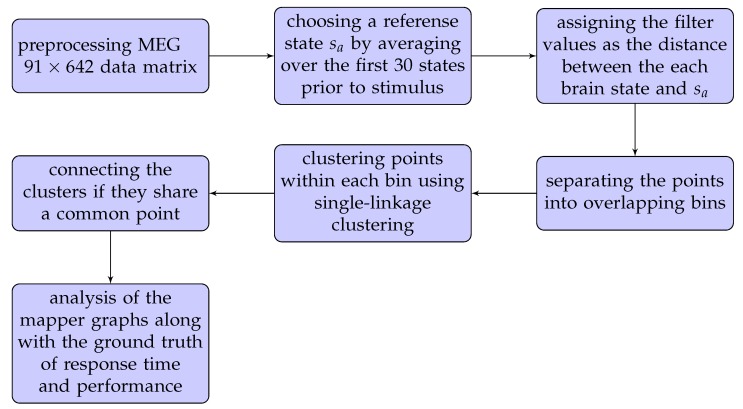
Flowchart illustrating the steps used to construct the Mapper graph from magnetoencephalography (MEG) dataset.

We refer to the flowchart in Figure 1 for a quick illustration of our methods.

As a first step in the flowchart, we obtained the source-level processed data from HCP, which has 91 time-points vs. 642 voxels. The preprocessing step returned the parcellated results of source-reconstructed Working Memory averaged event-related and time-frequency responses using the Yeo et al.’s (2011) [33] 17-network parcellation. In this case, our point cloud of 91 time points whose coordinates are the readings from 642 MEG locations, is a high-dimensional space, referred to as time space. This process of extracting an ordered list from the raw data, such as a row in a matrix, is called vectorization, and is a common first step in machine learning methods. Even though vectorization causes the loss of the locations of voxels relative to each other, the process does not affect the outcome of the machine learning methods (see [37]).

To proceed with the algorithm, we next chose a metric to measure the similarity between the vectors (i.e., brain states) in the time space. We used the Euclidean metric, which has proven to be successful in previous applications of neuroimage data [26,28].

The second step in the flowchart is to set a reference state to be used in filter selection. The first 30 out of 91 time-points in our data were given as the *baseline* brain states recorded before the stimulus. We chose a reference brain state $s_{a}$ as the average of this 30 baseline states.

As a next step, we assigned a filter value to each brain state as the Euclidean distance between that state and the $s_{a}$. This is an idea suggested in [38] to trace the brain activation patterns during the 0-back (2-back) memory test of each participant. This method enables us to use the 2D representation of the brain dynamics by visually tracking if the brain sates return to earlier states or form new states, as displayed by loops, dead zones, and branches.

**Figure 2:**
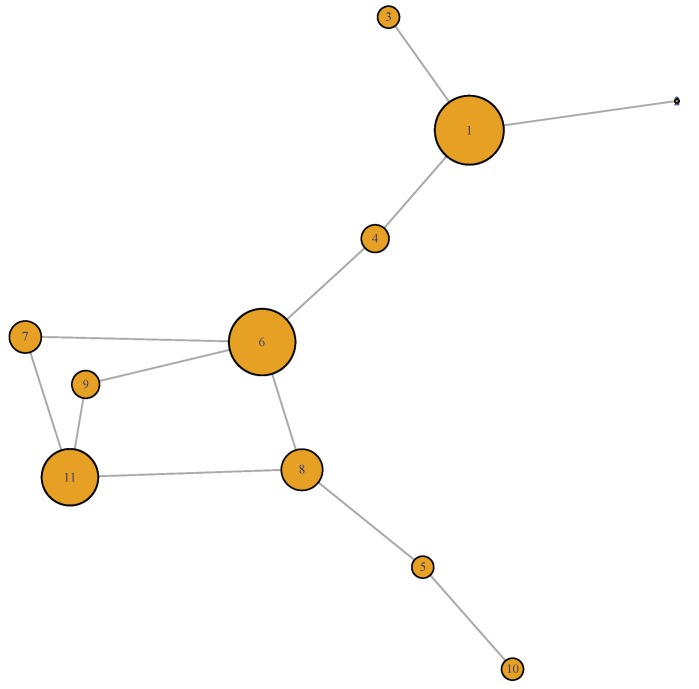
The Mapper graph of subject No.156334 illustrating the clusters of the 91 time-points during the 0-back memory test. The size of the nodes are directly proportional to the number of time-points they contain.

The metric and the filter together lead to the construction of the mapper graph via the next three steps indicated in the flowchart. In the first step, the brain states are binned by dividing the filter values into overlapping intervals. The number of intervals as well as overlapping percentage (i.e., parameters) is rigorously discussed in the next subsection. Two brain-states fall in the same bin if their distances from $s_{a}$ are similar. In the second step, each bin is clustered in a way that two brain-states are included in the same cluster if they are close to each other with respect to the Euclidean metric. This is done using the single linkage clustering, which is widely used in TDA. The third step is the construction of edges. As the bins are allowed to overlap, the clusters from different bins may share common points. The clusters share a common point are joined by an edge (see Figure 2).

**Figure 3:**
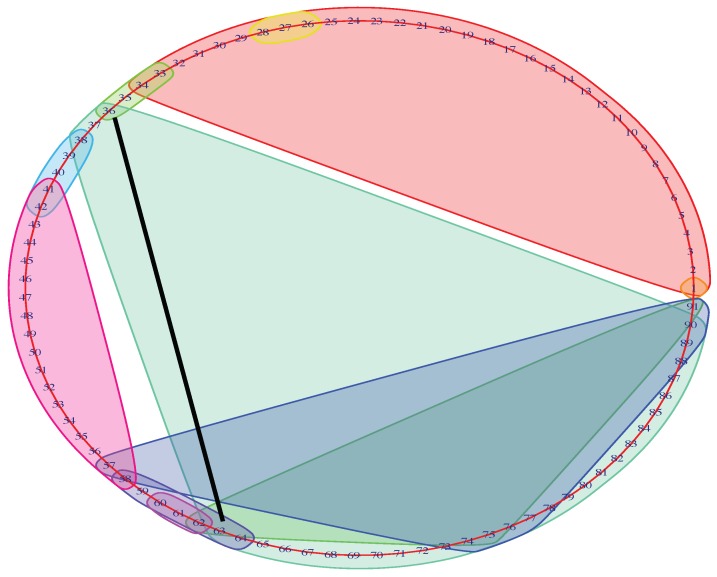
Unconventional Mapper graph of subject No. 156334 with 97% accuracy in 0-back memory test. The graph shows the members (i.e., time-points) of the each node in the Mapper graph. The black straight line indicates the first time-point t=63 during the stimulus that returns to the beginning of the stimulus.

To trace the brain patterns, we used an unconventional visualization of the Mapper graph. In Figure 3, each cluster corresponds to a node in the mapper graph. In this way, we can visually track the changes in brain states during the task.

The input MEG data have temporally ordered 91-brain-states. From the raw data, we know that the first 30 time-points represent the time interval before the stimulus. We then recorded the first time-point *t*, called return-point, during the stimulus where the brain state re-visits the beginning of the stimulus (see black line in Figure 3). In the next subsection, we show that this choice return-point was reliable across the majority of parameter variations.

As a final step, we analyzed different features of the mapper graph along with the ground truth of task performance and response time obtained from HCP database.

We used TDAmapper package in R to obtain the mapper graphs. For reading and normalizing the data, we used Fieldtrip package in MATLAB. The codes are available in the Supplementary Materials.

### 2.4. Parameter Selection

As explained in Section 2.2, Mapper algorithm has four parameters and, for the distance parameter, we selected the Euclidian metric. In this section, we discuss how to select the remaining parameters: the number of intervals (Int), the overlapping percentage (Ovr), and the number of bins of the clustering algorithm (Bin).

First, we defined a grid G with 18 points where Int∈{5,6,7}, Ovr∈{50,60,70}, and Bin∈{5,10}. A sample point in this grid is a triple (5,50,10) where the first component is the number of intervals, the second component is the overlapping percentage, and the third component is the number of bins. We used the notation (Int−Ovr−Bin) to represent a generic point in G.

**Figure 4:**
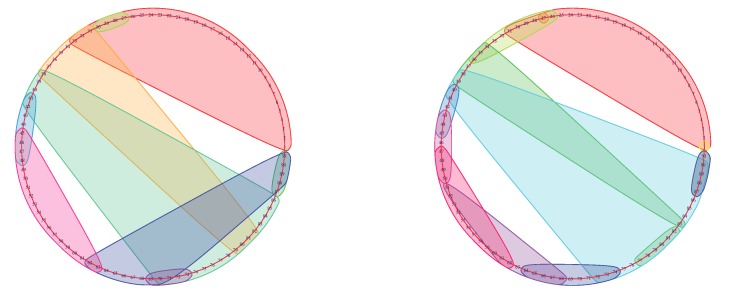
Mapper graphs of subject No. 175237 during the 0-back memory test. The graph on the left was obtained using parameters (5,50,10) and the one on the right with the parameters (7,70,5). The return to the beginning of the stimulus was at the time-point t=79 for the graph on the left and at t=83 for the graph on the right.

Then, we ran the Mapper algorithm with each point in G. The resulting Mapper graphs are visualized as in Figure 4.

**Figure 5:**
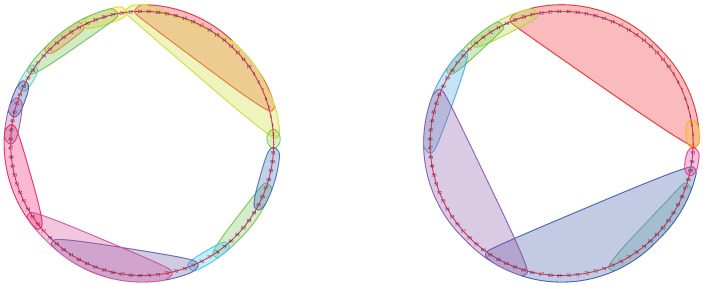
Subjects No. 353740 (left) and No. 917255 (right) without a return-point.

We recorded the time-point marking the return to the beginning of the stimulus for every subject and point in G for both memory tests. For some points in G, the subjects may not have a return point. In these cases, the mapper graphs appear as in Figure 5.

If a subject does not have a return-point for at least half of the points in G, we excluded them from the data. If a subject does not have a return-point for fewer than half of the points in G, we complete those missing return-points for that subject using the mean of the existing return-points.

To complete cleaning our data, we chose subjects common in both tests. As a result, 70 subjects were left in both tests.

The cleaned dataset has seven columns. The column Subject represents the subject number. The columns Mapper0B and Mapper2B represent the return-points obtained from the mapper graphs with the selected parameters of the 0-back and 2-back tests, respectively. The columns 0B Median Reaction and 2B Median Reaction represent the median reaction time of the subjects from the tests 0-back and 2-back, respectively. Reaction time is the time from the onset of the image to the response in milliseconds. Hence, for a subject, 0B Median Reaction is the median of the reaction times of that subject to the 0-back tasks, and similarly for the 2B Median Reaction. The columns 0BAccuracy and 2BAccuracy represent the accuracy scores of the subjects obtained from the the tests 0-back and 2-back, respectively. Accuracy is the percentage of the correctly responded tasks.

Once the dataset was cleaned, we selected the parameters for the Mapper that are highly correlated with the accuracy scores.

### 2.5. Statistical Analysis

For both 0-back and 2-back tests, we sketched the return-time points against the accuracy and median reaction time against the accuracy.

**Figure 6:**
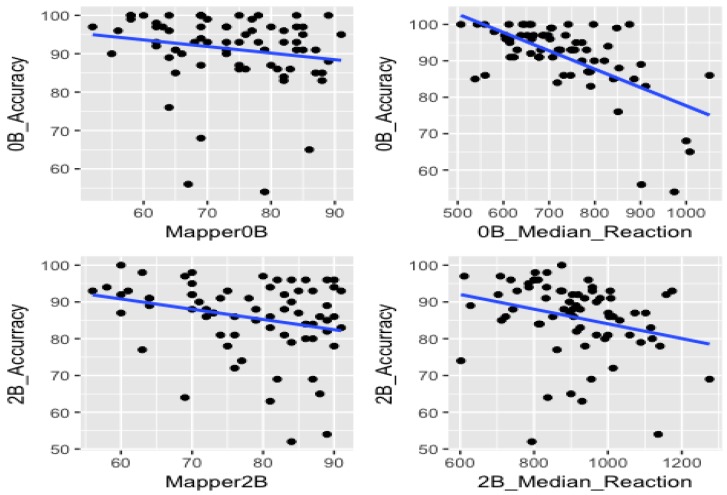
The blue lines represent the regression lines for the return point in the mapper graph 0-Back vs. accuracy in 0-back(upper left), the return point in the mapper graph 2-Back vs. accuracy in 2-back (lower left), accuracy in 0-back vs reaction-time in 0-back (upper right) and accuracy in 0-back vs reaction-time in 0-back (lower right). They all have negative slopes, meaning that both return-time points and median reaction times are negatively correlated with the accuracy.

In our statistical analysis, we investigated whether the combined effects of return-time points and the median reaction times can enhance correlation with the accuracy scores of the subjects observed in the plots in Figure 6.

## 3. Results

We determined the Mapper parameters based on the strength of the correlation between the parameters and the accuracy score.

### 3.1. Distinguishing 0-Back and 2-Back Memory Tasks

**Figure 7:**
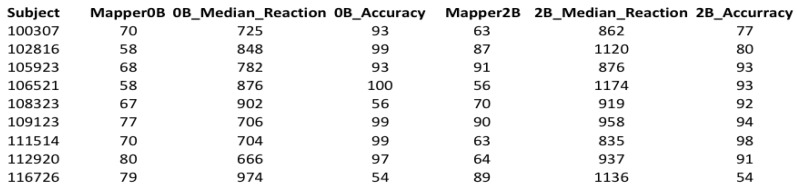
A snapshot of the cleaned dataset showing the first 10 subjects.

We statistically analyzed the results along with the ground truth of response time and performance of the participants (see Figure 7 and Supplementary Materials for the complete table). The Mapper graphs for all participants are available in the Supplementary Materials.

**Figure 8:**
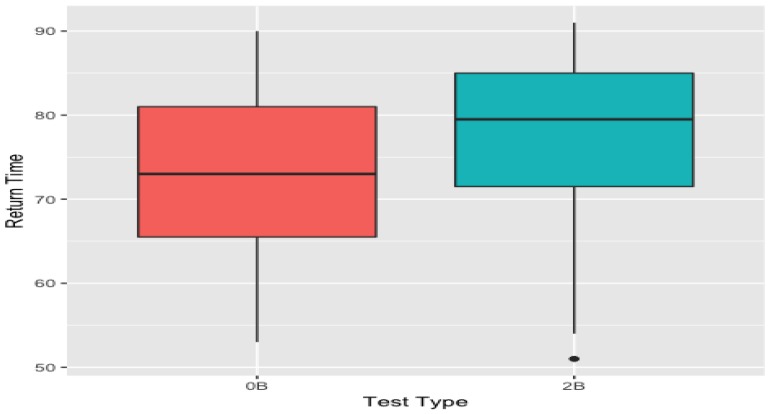
The box plots of the return-points obtained from 0-back and 2-back mapper graphs. 0-back values are significantly lower than 2-back values. These box plots were obtained using the return-points from the parameters (7−70−10) as it had the most significant correlation score of −0.20065 (see Figure 9).

The return-point obtained from 0-back mapper graph is significantly lower than its 2-back counterpart (*p* = 2 ×${}^{-10}$) (see Figure 8).

### 3.2. Predicting Task Performance

We next compared the return-points obtained from 0-back (mapper0B) and 2-back (mapper2B) graphs of each participant with the accuracy and response time data from HCP (see the Supplementary Materials for the corresponding table and detailed statistical analysis).

Using linear regression, we compared the three models for both memory tests.

Model 1: Accuracy∼MedianReactionTime; and
Model 2: Accuracy∼ReturnTime+MedianReactionTime.

**Figure 9:**
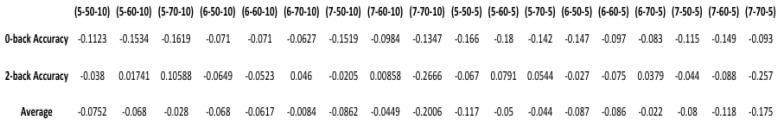
The table shows correlation scores between accuracy scores of each test and the points in G. We also recorded the column wise mean of the correlation scores as we selected the coefficient when we compared the two types of the memory test based on this mean. It is also worth noting that the scores on the table are consistent with the scatter plots in Figure 6.

In case of 0-back test, we used the Mapper data with parameters (5−60−5) as they provide the strongest correlation with accuracy (see Figure 9). We obtained the following results for each one of the above models:


**Models**

**RSE**

**R squared**

***p*-value**





**1**
7.3740.38635.64 × 10${}^{-9}$




**2**
7.3110.39681.65 × 10${}^{-8}$

For the 2-back test, we used parameters (7−70−10) and obtained the results below.


**Models**

**RSE**

**R squared**

***p*-value**





**1**
9.8330.06122 × 10${}^{-2}$




**2**
9.6270.10.1

From these results, we observed that Model 2 is the best model for both tests as it had the highest R squared value and lowest RSE and *p*-values. Thus, we can conclude that return-point from the Mapper graphs is a useful feature in the description of the accuracy scores. However, it is also worth mentioning that the linear models were not the best models to describe the relation between these features as the RSE values were significantly high and R square values were significantly low. In a good linear model, these values should be close to 0 and 1, respectively.

### 3.3. Cluster Analysis

**Figure 10:**
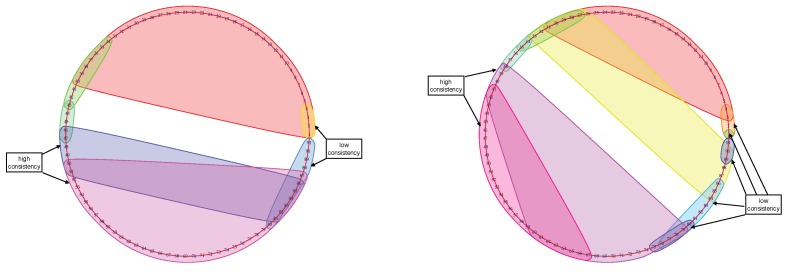
Mapper graph of subject No. 1125525 (left) during the 2-back memory test on the left. Mapper graph of subject No. 149741 (right) during the 0-back memory test on the right. The big clusters in the middle of stimulation where subjects were highly engaged indicate the higher consistency between brain activity patterns. The smaller clusters at the beginning and the end of the stimulus where the subjects’ engagement was lower indicate low consistency.

The resulting Mapper graphs for each participant shows the time evolution of the brain states. The clusters detect the consistency brain states. The big clusters seen during the middle of the stimulus were observed for most of the participants. These clusters show the similarity in the whole brain configuration when the brain was highly engaged in the memory task. On the other hand, smaller clusters at the beginning and the end with lower engagement disclose the lower similarity in the brain configuration (see Figure 10 for examples). Another noticeable cluster is the baseline before the stimulus.

A significant variability in the Mapper graph was also observed across subjects. For example, the start and end time of apparent big clusters differ between subjects.

## 4. Discussion

It is important to develop new mathematical methods and computational tools in order to investigate the role of the dynamic nature of the brain in cognition. Mapper, based on topology, is one of the unconventional candidates to extract meaningful information from high spatiotemporal dimensional neuroimaging data, such fMRI, MEG and EEG. It has already proven its power in different fields by stratifying the complex datasets to identify biomarkers of viral evolution, diabetes, and preclinical spinal cord injury [25,26,27]. Using this novel tool on fMRI datasets, the behavioral dynamics of the brain was previously studied by Saggar et al. [19]. Unlike with the common techniques [39,40], the resulting graph is obtained without spatially or temporally reducing the dimension of the data; hence, it provides significant insight about the temporal changes in the whole brain.

In this study, we unraveled brain dynamics further in time via a similar methodological pipeline at single-participant level. As the temporal resolution of fMRI is a limitation for this purpose, we resorted to high temporal resolution MEG data from working memory *n*-task. The characteristic that makes Mapper suitable for studying the high temporal dimensional MEG dataset is its scalability. It divides the complete set into small subsets that can be run in parallel and merges at the end to obtain final graph. This makes it good candidate over slower clustering algorithms already applied in neuroimaging data: hierarchical clustering [41], spectral clustering, k-means clustering, or fuzzy clustering [42].

Our approach has several contributions to the examination of the dynamics of electrophysiological data. First, the application of our method to MEG data detected a novel neuroimaging marker that explained the performance of the participants along with the ground truth of response time. The earlier work provides evidence to the fact that using graphs obtained from electrophysiological signals (i.e., EEG and MEG), it is possible to find online markers for the relationship between dynamic neural networks and task outcomes [43]. Following the suggestion in [38], we explored the Mapper graph visualizing the dynamic evolution of the brain states during the working memory task. Even though there is no correlation between the ground truth of response time and the return-point, where the brain state in the Mapper graph comes back or close to the baseline prior to the stimulus, these two measures together better explain the high performance of the participants. Our result are also consistent with the previous electrophysiological data studies, suggesting that the relationship between working memory capacity is more strongly reflected in large-scale brain dynamics than in spatially localized activity [44].

Secondly, TDA enabled us to distinguish two task-positive brain activations during 0-back and 2-back tasks, which is hard to detect with the other pipelines that require collapsing the data in the spatial and temporal domain. We demonstrated that the return-point of 0-back tasks are significantly smaller than the return-points of 2-back task providing a novel online marker to differ 0-back and 2-back tasks. This subtle marker might not be possible to obtain with a fMRI dataset because of the low temporal resolution. Similarly, more conventional methods such as the sliding window might fail as there is loss of high temporal resolution while choosing time-course intervals during implementation of these methods. Our approach is robust to parameter perturbation unlike the other methods for which the choice of the parameters is crucial to detect the characteristics of the dynamic evolution of the brain states.

Furthermore, the Mapper graphs of the individuals reveal one large group in the middle of the stimulus, detecting the high engagement in the brain with fine temporal resolution, which could contribute to increase spatiotemporal resolution with other imaging modalities. The resulting big clusters during the stimulus indicate the similarity in the whole brain configuration during a high engagement, while the size of the clusters gets smaller at the beginning and the end of the stimulus. This is in line with the earlier study using ICA based characterization of dynamic MEG networks during a working memory task. The transiently forming and dissolving networks in different time intervals detected and characterized in [6] are compactly indicated in a Mapper graph as large and small nodes that contain brain states sharing similar temporal profiles. As suggested in [19], by combining MEG (or EEG) and fMRI datasets using Mapper may enable the investigation of the brain dynamics in the highest spatiotemporal resolution, for which other conventional methods fail. Our result support this hypothesis by finding meaningful patterns in high temporal resolution MEG data.

It is also notable that the mapper graphs are highly variable across subject. This variation across subjects has been noticed in earlier static MEG connectivity studies [45,46]. As noted in [47], these variations across subjects may be due to identifiable intrinsic processes, which are subject specific.

As a future work, one can also investigate the MEG dataset that contains different tasks such as math-story, motor and working memory. Due to the high temporal resolution of MEG, such data are very high dimensional and require high computational power to apply Mapper algorithm, unless they are collapsed on the temporal scale. Alternatively, the algorithm can be run in parallel for every interval and the overlapping clusters can be connected in the next step. Another future direction is to analyze the Mapper clusters quantitatively using graph centrality scores. In that case, we expect that the main clusters (i.e., nodes of mapper graph) during stimulus will posses high centrality scores. We can also look for a better model than linear regression to describe accuracy in terms of return points and reaction time. Finally, other TDA methods such as persistent homology might give interesting insight on the dynamic features of brain imaging data. One possible approach for studying graph representations of time series of dynamical systems is suggested in [48].

Our approach can be later used to explore mental disorders such as autism and schizophrenia where cognitive impairment is common and unrelated brain regions are expected involve during the cognition. The certain characteristics of the Mapper graphs may differ between control and patient groups; hence, it might help to find the underlying neural reason for the cognitive impairment.

## 5. Data Availability

The MEG data used in this study are publicly available from the Human Connectome Project (HCP) consortium: http://db.humanconnectome.org.

## 6. Conclusions

In summary, our work demonstrates that TDA methods can be successfully applied to extract new perception from the complex brain dynamics associated with high temporal MEG data during a cognitive task. Unlike the standard techniques, it achieves these results without the need of temporally and spatially collapsing the data. It is also robust to parameter perturbation while the parameter choice is critical in the widely used methods such as the sliding window. Finally, it provides a compact and easily readable visual presentation of the data preserving the geometric structure.

## Supplementary Materials

The following are available online at https://www.mdpi.com/2076-3425/9/6/144/s1.
