# Supplementary material for: Uncovering Dynamic Brain Reconfiguration in MEG Working Memory n-Back Task Using Topological Data Analysis
Source: Brain Sci. 2019 Jun 19;9(6):144. doi: 10.3390/brainsci9060144 (PMC6628086; doi:10.3390/brainsci9060144)
Supplement: Supplementary file 1 [file brainsci-09-00144-s001.zip › Supplementary 2/Codes.pdf]

MATLAB code for reading and normalizing

```
timeseries=[];
for i=1:length(freqnames)
   %this loads the data for 107 brain regions and 91 timepoints for one
   %frequency band
   dat=ft_read_cifti(['872764_MEG_Wrkmem_srcavgdics_[LM-TIM-2B]_[FB-'
freqnames{i} '].power.Yeo2011.ptseries.nii']);
   %combine spatial locations and frequencies in one dimension
   timeseries=[timeseries; dat.power];
end
```

R code for to obtain and plot Mapper graphs

```
A <- read.csv(file="data100307_0B.txt")
#distance function
eucl <- dist(as.data.frame(t(B)), method = "euclidean")

#filter function
baseline <- matrix(0, nrow = 1, ncol = 641)
for(i in 1:30){baseline <- baseline + B[,i]}
baseline <- baseline/30
filter <- matrix(0, nrow=1, ncol=91)
for(i in 1:91){filter[1,i] <- dist(rbind(baseline, B[,i]), method="euclidean")}

#mapper
B.mapper <- mapper(
  dist_object  = as.matrix(eucl),
  filter_values = t(filter),
  num_intervals = 5,
  percent_overlap = 50,
  num_bins_when_clustering = 10)


#visualization
library(igraph)
for(i in 1:B.mapper$num_vertices){assign(paste0("f",i),
sort(B.mapper$points_in_vertex[[i]]))}
gring <- make_ring(91)
V(gring)$shape <- "none"
#gringnew <- add_edges(gring,c(36,89,88,91))
mylist <- vector("list",length = B.mapper.tsne$num_vertices)
for(i in 1:B.mapper$num_vertices){mylist[[i]]<-get(paste0("f",i))}
plot(gring, layout = layout.circle, vertex.label.cex=0.7, edge.curved=F,
edge.color="red",mark.groups=mylist)
```
