# Supplementary material for: Uncovering Dynamic Brain Reconfiguration in MEG Working Memory n-Back Task Using Topological Data Analysis
Source: Brain Sci. 2019 Jun 19;9(6):144. doi: 10.3390/brainsci9060144 (PMC6628086; doi:10.3390/brainsci9060144)
Supplement: Supplementary file 1 [file brainsci-09-00144-s001.zip › Supplementary 2/statistical_analysis.pdf]

Statistical Analysis of the Mapper Data

In this document, we do some statistical analysis on the mapper results and the HCP data combined.

Reading the Data

First of all, we read our data into the following data frames after removing NAs.

A 65×5 data frame for 0B test data:

```
##   Subject Map0B Acc0B Time0B Acc0B_Grade
## 1  100307    72    93    725           A
## 2  102816    66    99    848           A
## 3  105923    68    93    782           A
## 4  106521    57   100    876           A
## 5  108323    58    56    902           F
## 6  109123    72    99    706           A
```

A 70×5 data frame for 2B test data:

```
##   Subject Map2B Acc2B Time2B Acc2B_Grade
## 1  100307    88    77    862           C
## 2  102816    89    80   1120           B
## 3  105923    86    93    876           A
## 4  106521    87    93   1174           A
## 5  108323    86    92    919           A
## 6  109123    88    94    958           A
```

A 63×5 data frame where the above two frames are joined by the Subject column:

```
##   Subject Map0B Acc0B Time0B Acc0B_Grade Map2B Acc2B Time2B Acc2B_Grade
## 1  100307    72    93    725           A    88    77    862           C
## 2  102816    66    99    848           A    89    80   1120           B
## 3  105923    68    93    782           A    86    93    876           A
## 4  106521    57   100    876           A    87    93   1174           A
## 5  108323    58    56    902           F    86    92    919           A
## 6  109123    72    99    706           A    88    94    958           A
```

In the above frames, the column AccxB_Grade classifies the accuracy score on the column AccxB as A, B, C, D, F according to the rule: A if AccxB $\in [90, 100]$, B if AccxB $\in [80, 89]$, C if AccxB $\in [70, 79]$, D if AccxB $\in [60, 69]$, and F for the rest.

We note that the χ^2-test applied to the contingency table

```
##     2B
## 0B    A  B  C  D  F
##   A  24 17  2  1  0
##   B   1 10  2  1  1
##   D   0  0  0  2  0
##   F   1  0  0  0  1
```

gives a p-value of $6.792e - 08$. This p-value shows strong relation between the accuracies of the test 0B and the test 2B as it is significantly less than 0.05. We also note that there are no C's in test 0B.

0B Test Results

In this paragraph, we study the relation between the mapper, time and accuracy category features of the 0B test. These features correspond to the columns Map0B, Time0B, and Acc0B_Grade columns in the first data frame whoe first 6 rows are given at the beginning of the document.

Accuracy as the independant feature

We investigate the relation between Acc0B_Grade and Map0B. We assume Acc0B_Grade is the independant variable and Map0B is the dependant variable. We use the ANOVA test to confirm the relation. The test gives a p-value of 0.00492. Based on this p-value which is less than the assumed significant level of 0.05, we deduce that there are significant differences between some of the mean values of the accuracy classes. To determine which accuracy classes' averages exhibit significant differences, we use Tukey Honest Significant Differences, a.k.a. Tukey HSD, pariwise comparison.

```
##   Tukey multiple comparisons of means
##     95% family-wise confidence level
##
## Fit: aov(formula = results_0B$Map0B ~ results_0B$Acc0B_Grade)
##
## $`results_0B$Acc0B_Grade`
##           diff         lwr       upr     p adj
## B-A   6.395652   0.4158446 12.375460 0.0315575
## D-A   5.695652  -8.8312677 20.222572 0.7293296
## F-A -11.304348 -25.8312677  3.222572 0.1795240
## D-B  -0.700000 -15.8394699 14.439470 0.9993417
## F-B -17.700000 -32.8394699 -2.560530 0.0156271
## F-D -17.000000 -37.1116099  3.111610 0.1259769
```

From this table, the only significant differences of means are observed between the accuracy classes A and B and the accuracy classes B and F with the adjusted p-values of 0.0315 and 0.0156, respectively.

In order for the ANOVA test results to be reliable, Map0B should follow normal distribution and the variances of MAP0B and Acc0B_Grade should be equal. As the number of subjects involved in the test 0B is 65 and more than 30, by the central limit theorem, we can assume Map0B ditributes normally. However it is a good practice to verify normality both visually and numerically.

For visual verification of the normalitiy of Map0B, we sketch the histogram

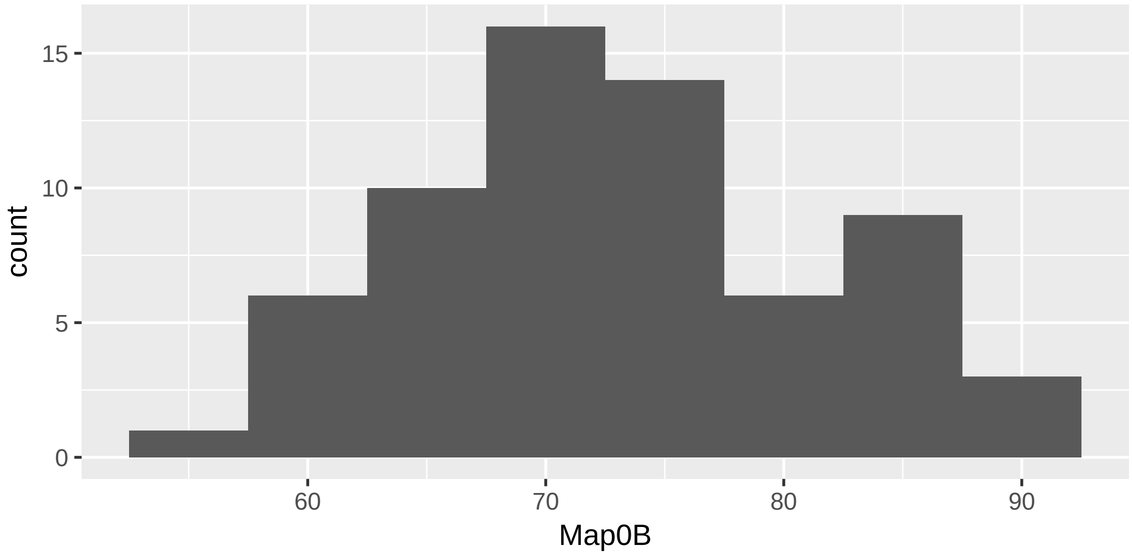

and the Q-Q plot

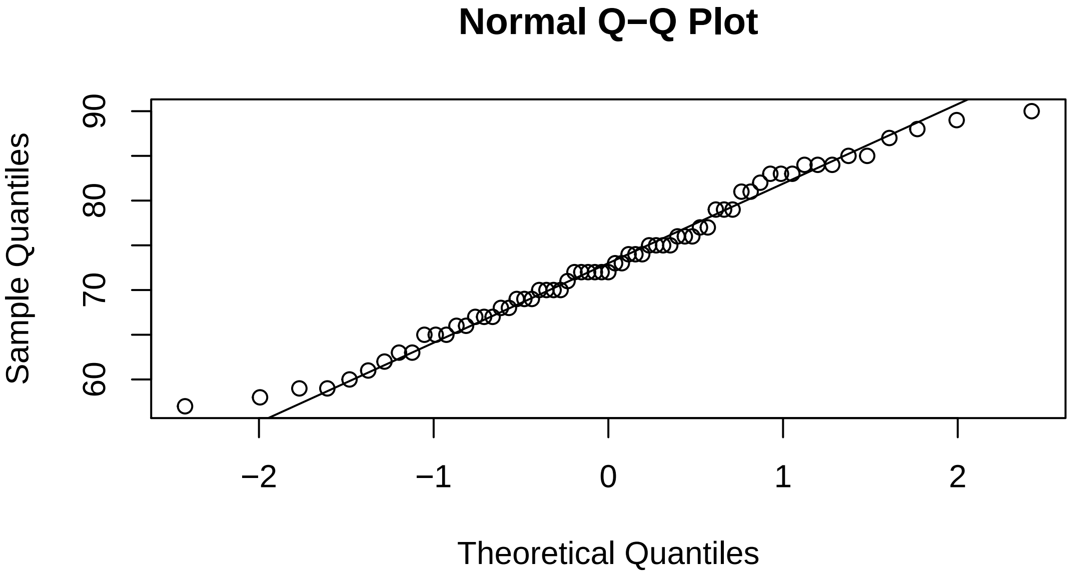

and for numerical verification, we use Shapiro-Wilk Normality test which gives a p-value of 0.4128 that is greater than 0.05 and therefore confirms the nomality of the data.

As for the equality of the variances of Map0B and Acc0B_Grade, we use Levene's test. It gives a p-value of 0.6906 which is greater than 0.05 and therefore the equality of the variances is confirmed.

We can conclude that there is a significant difference between the Map0B means of subjects in the accuracy class A and the accuracy class B and in the accuracy class B and the accuracy class F.

Accuracy as the dependant feature

In this paragraph, we use logistic regression to investigate whether Map0B, or Time0B, or Map0B and Time0B together better explains the subjects who got A from Accuracy in test 0B. We first filtered the test 0B data for subjects who got A in accuracy.

```
##   Subject Map0B Acc0B Time0B Acc0B_Grade Grade0A
## 1  100307    72    93    725           A       1
```

```
## 2  102816     66     99     848             A        1
## 3  105923     68     93     782             A        1
## 4  106521     57    100     876             A        1
## 5  108323     58     56     902             F        0
## 6  109123     72     99     706             A        1
```

Then, in all of our models, we used 80% of the above data for the training and the remaining 20% for the testing.

1) The accuracy of the model Grade0A~Map0B is 75% meaning the model predicted whether the accuracy grade is A or not corrcet 75% of the time.
2) The accuracy of the model Grade0A~Time0B is 83.3%.
3) The accuracy of the model Grade0A~Time0B+Map0B is 91.7%.

So we can conclude that adding mapper to the observed time increased the accuracy prediction.

2B Test Results

We repeat the analysis we did for the 0B test data for the 2B test data. We give the summary of our findings:

1) The ANOVA test does not detect any significant difference between the means of the mapper of different accuracy classes. We shall also note that the mapper data does not distribute normally. Therefore ANOVA results might not be reliable. As the number of subjects in the 2B test is 70 and so more than 30, the normality is not a major concern, however it is a common practice to use Kruskal-Wallis test which is the non-parametric alternative to ANOVA test, in such cases. The result of this test also does not either detect a significant difference between the means.
2) The logistic regression applied to 2B test data gave the accuracy 69.2% for all theree models namely Grade2A~Map2B, Grade2A~Time2B, and Grade0A~Time2B+Map2B.
