# Supplementary material for: Uncovering Dynamic Brain Reconfiguration in MEG Working Memory n-Back Task Using Topological Data Analysis
Source: Brain Sci. 2019 Jun 19;9(6):144. doi: 10.3390/brainsci9060144 (PMC6628086; doi:10.3390/brainsci9060144)
Supplement: Supplementary file 1 [file brainsci-09-00144-s001.zip › Supplementary 2/Table 1.pdf]

	Subject	Mapper0B (Label of the timepoint n<91)	Accuracy 0B(%)	Mean reaction time 0B(ms)	Mapper 2B	Accuracy 2B(%)	Mean Reaction Time 2B(ms)
1	100307	72	93	725	88	77	862
2	102816	66	99	848	89	80	1120
3	104012	77	85		86		
4	105923	68	93	782	86	93	876
5	106521	57	100	876	87	93	1174
6	108323	58	56	902	86	92	919
7	109123	72	99	706	88	94	958
8	111514	70	99	704	63	98	835
9	112920	79	97	666		91	937
10	113923		94	766	80	90	959
11	116726	63	54	974	77	54	1136
12	125525	85	65	1008	80	65	900
13	133019	79	91	678	84	86	851
14	140117	76	85	886	75	87	1102
15	146129	65	85		82		
16	149741	84	91	678	83	91	979
17	151526	81	86	1051	87	78	1141
18	156334	62	97	603	71	88	743
19	158136	79	87	787	83	52	794
20	162026	74	97	604	82	84	816
21	162935	69	87	790	89	91	1008
22	164636	69	94	829	63	93	1006
23	166438	75	100	642	82	88	881
24	172029	75	95	772		72	1014
25	175237	72	100	646	85	96	737
26	177746	67	90	821	81	92	1158
27	175540	67	91	615	84	90	896
28	182840	68	97	736	87	69	1275
29	185442	65	96	614	78	85	712
30	189349	60	97	638	68	92	904
31	191033	75	90	861	64	85	942
32	191437	76	93	682	76	78	938
33	191841	89	88	853	85	83	1116
34	192641	70	86	747	87	81	1000
35	195041	83	93	630	85	96	811
36	198653		97	686	87	93	754
37	200109	90	100	559	90	98	803
38	204521	87	85	841	90	81	969
39	205119	85	97	652	91	86	1004
40	212318	84	86	732	78	86	723
41	214524	72	98	585	78	92	614
42	223929	72	97	689	91	96	800
43	248339	77	86	560	83	89	628
44	250427	88	83	911	79	80	992
45	255639	83	95	759	86	82	915
46	257845	67	97	698	84	81	1059

47	283543	77	93	748	87	96	947
48	352738	59	91	708	69	88	925
49	353740	71	100	661	74	94	834
50	406836	69	100	507	74	97	611
51	433839	73	100	543	70	95	785
52	500222	63	100	797	87	88	905
53	512835	79	50		86		
54	568963	65	93	756	76	86	1014
55	581450	84	100	656	83	96	801
56	599671	72	81	767	78	88	860
57	601127		88	667	59	94	752
58	660951	82	67		91		
59	662551	84	47		82		
60	665254	59	90	799	77	87	897
61	667056	74	90	772	64	100	875
62	679770	70	100	612	84	87	1001
63	680957	61	89	901	75	87	1073
64	706040		63	1013	91	74	1013
65	707949	70	68	1000	82	63	930
66	715950		96	662	73	91	832
67	725751	76	96	605	85	93	872
68	735148		85	538	78	74	602
69	783463	81	93	751	89	89	914
70	814649	73	91	678	75	93	958
71	825048	66	75		67		
72	872764	65	100	742	71	90	969
73	877168	83	84	718	84	79	1089
74	891667	66	95	614	82	94	786
75	898179	74	93	720	88	85	1032
76	912447	82	83	791	88	69	955
77	917255	75	87	785	83	83	925
78	990366		91	623	74	92	703
