# Supplementary figures and images for: Uncovering Dynamic Brain Reconfiguration in MEG Working Memory n-Back Task Using Topological Data Analysis (part 2 of 2)
Source: Brain Sci. 2019 Jun 19;9(6):144. doi: 10.3390/brainsci9060144 (PMC6628086; doi:10.3390/brainsci9060144)

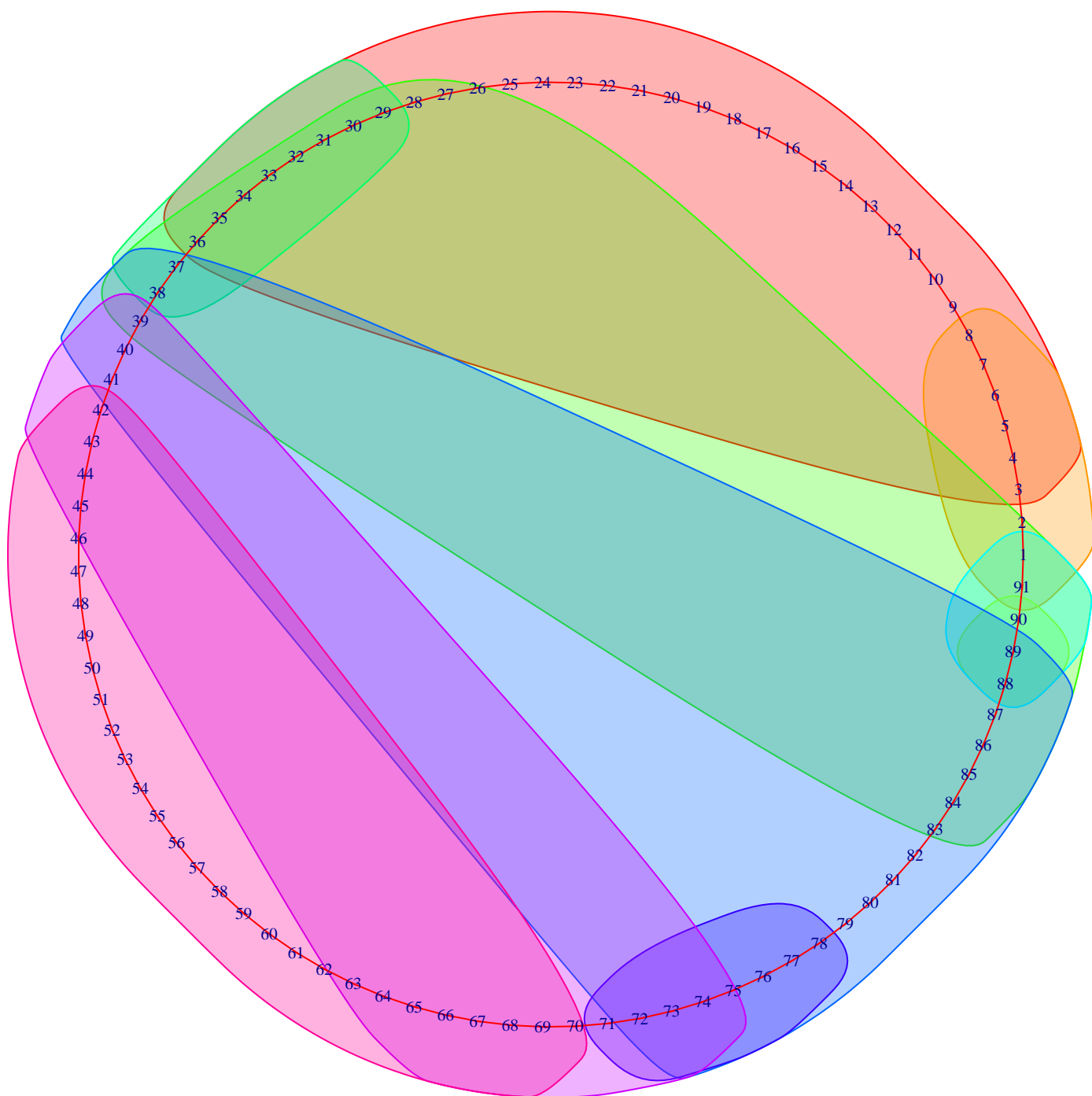

Supplement: Supplementary file 1 [file brainsci-09-00144-s001.zip › Supplementary 2/Mapper_graphs/581450_0B.pdf]

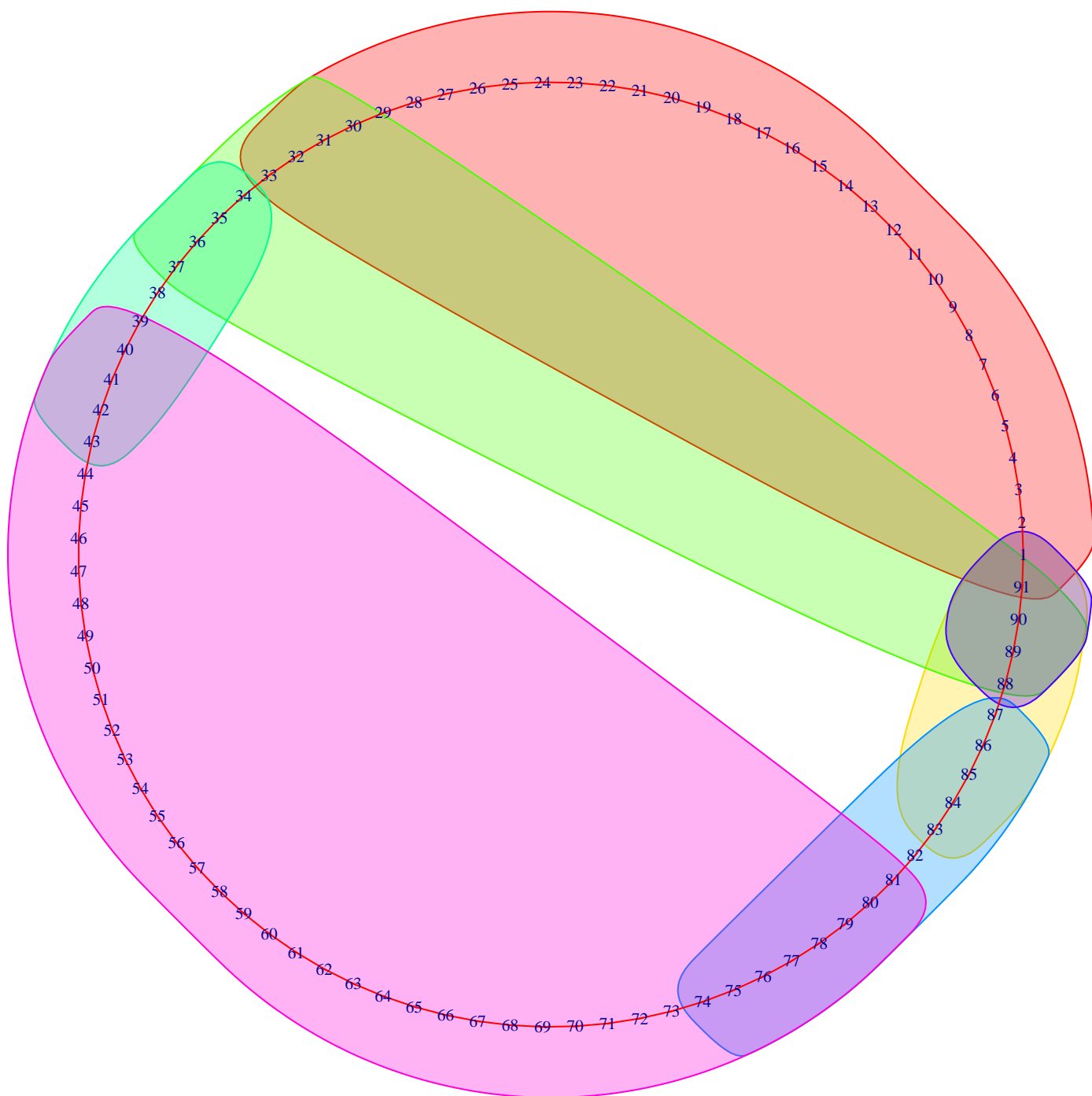

Supplement: Supplementary file 1 [file brainsci-09-00144-s001.zip › Supplementary 2/Mapper_graphs/109123_graph2B.pdf]

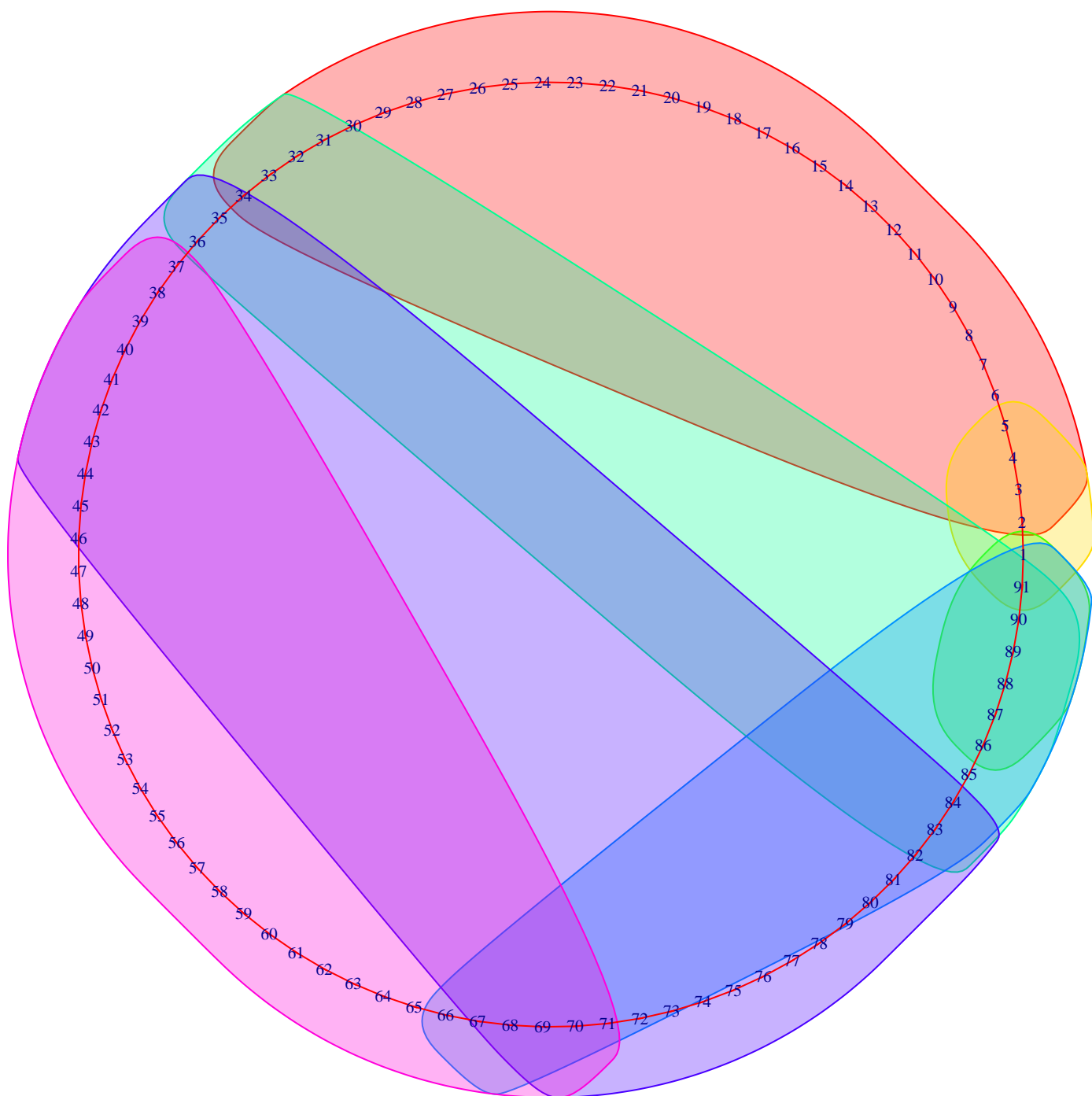

Supplement: Supplementary file 1 [file brainsci-09-00144-s001.zip › Supplementary 2/Mapper_graphs/248339_2B.pdf]

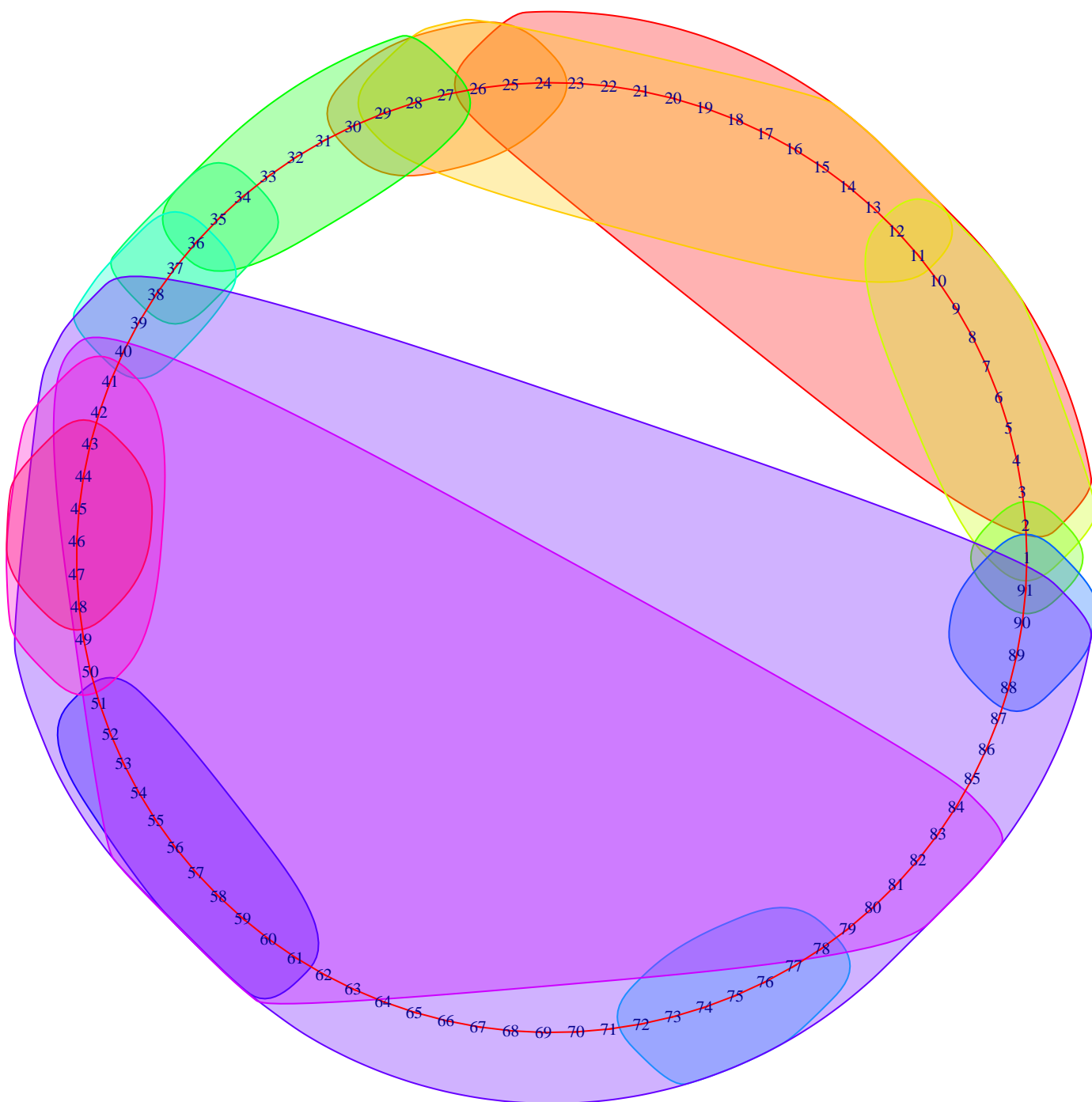

Supplement: Supplementary file 1 [file brainsci-09-00144-s001.zip › Supplementary 2/Mapper_graphs/113922_graph2B.pdf]

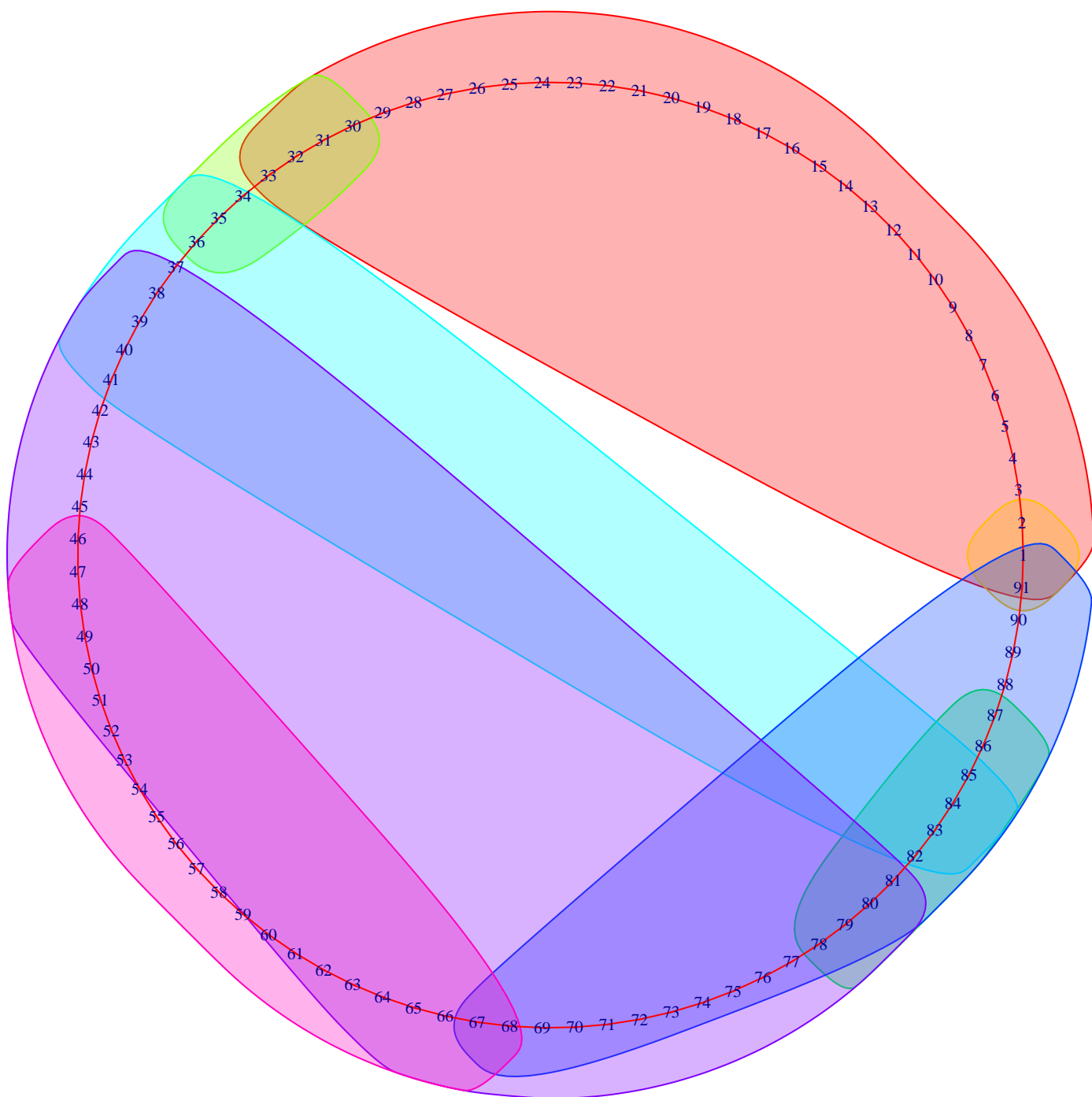

Supplement: Supplementary file 1 [file brainsci-09-00144-s001.zip › Supplementary 2/Mapper_graphs/877168_0B.pdf]

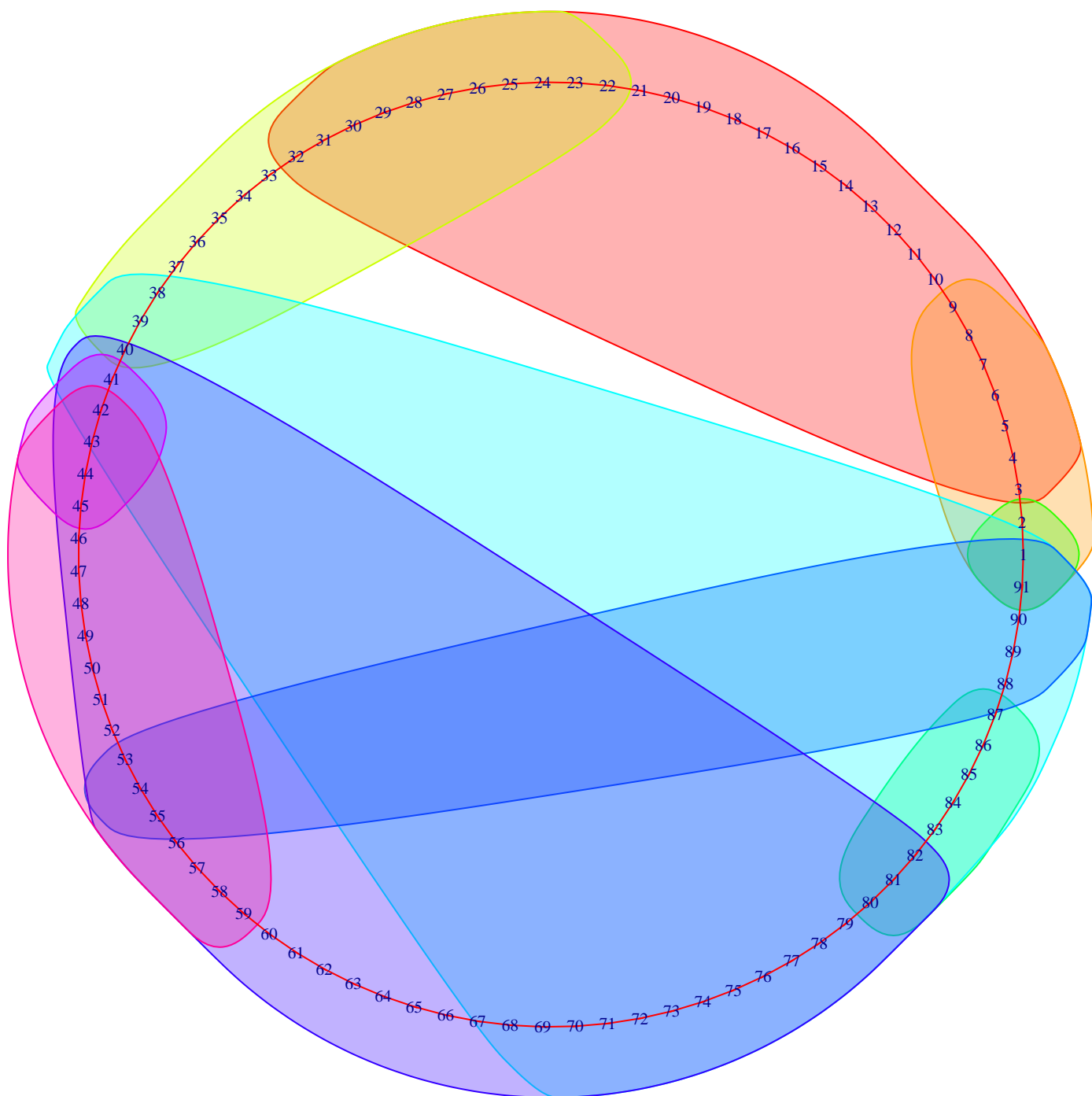

Supplement: Supplementary file 1 [file brainsci-09-00144-s001.zip › Supplementary 2/Mapper_graphs/182840_0B.pdf]

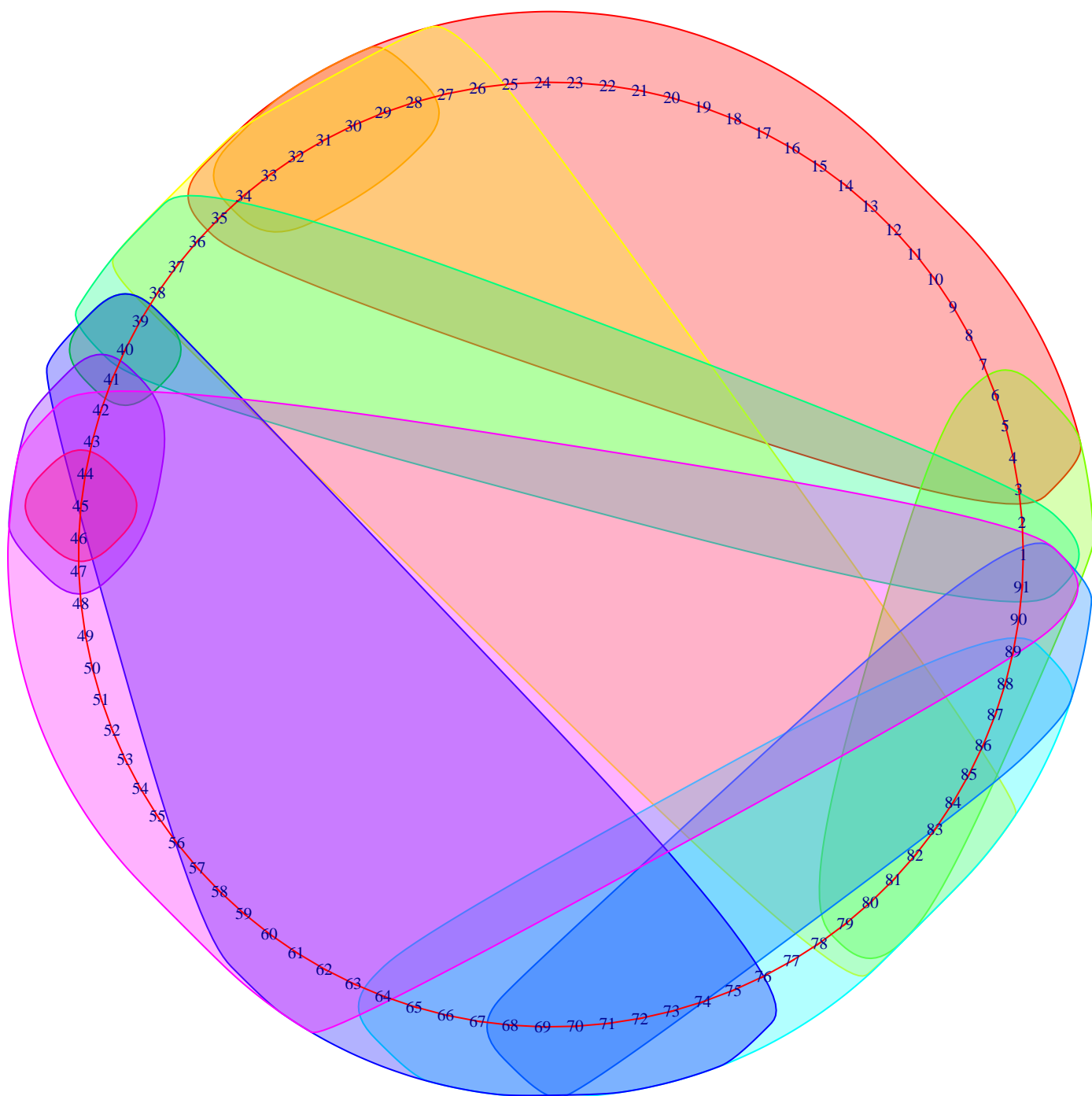

Supplement: Supplementary file 1 [file brainsci-09-00144-s001.zip › Supplementary 2/Mapper_graphs/158136_graph0B.pdf]

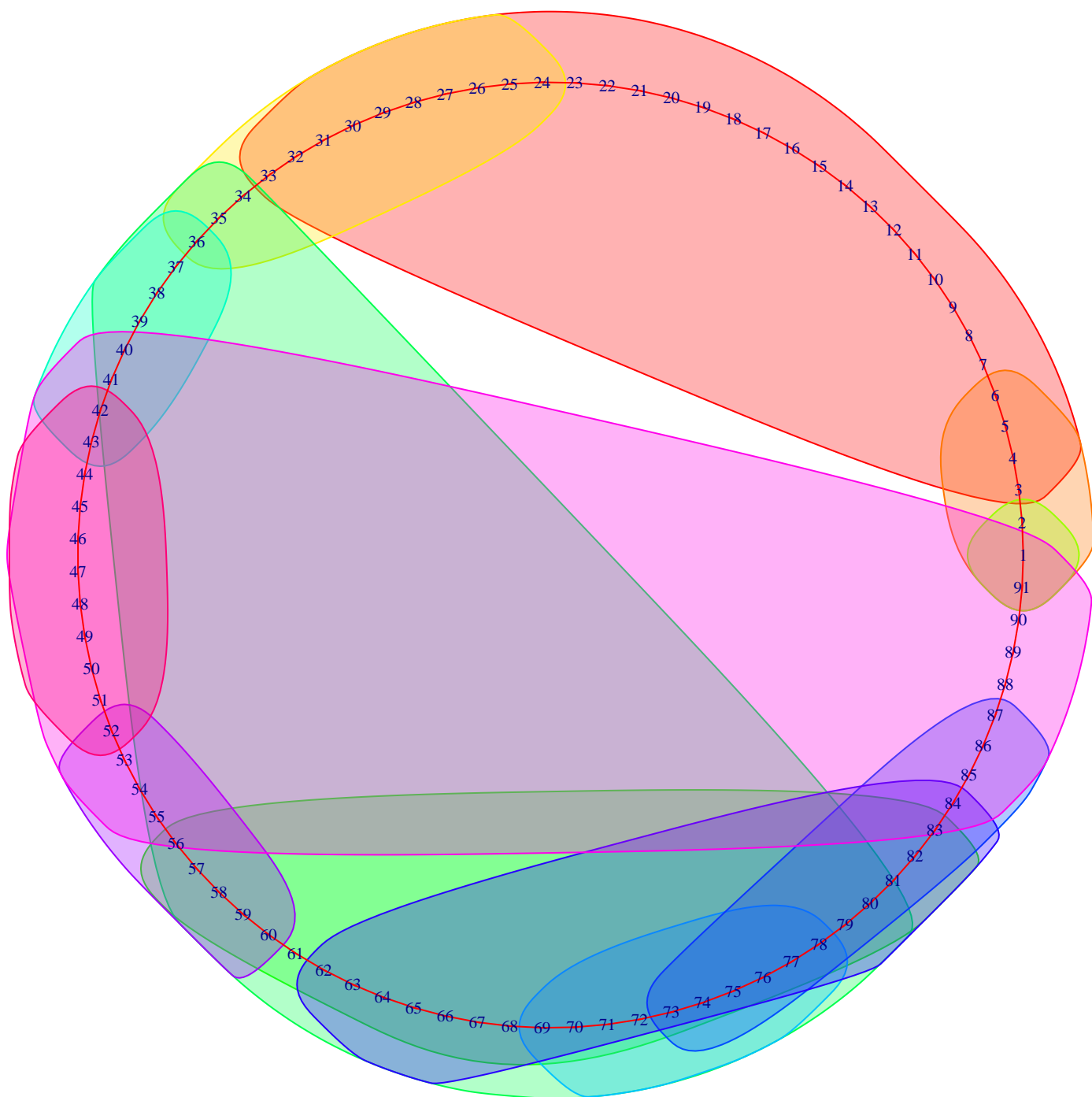

Supplement: Supplementary file 1 [file brainsci-09-00144-s001.zip › Supplementary 2/Mapper_graphs/106521_graph0B.pdf]

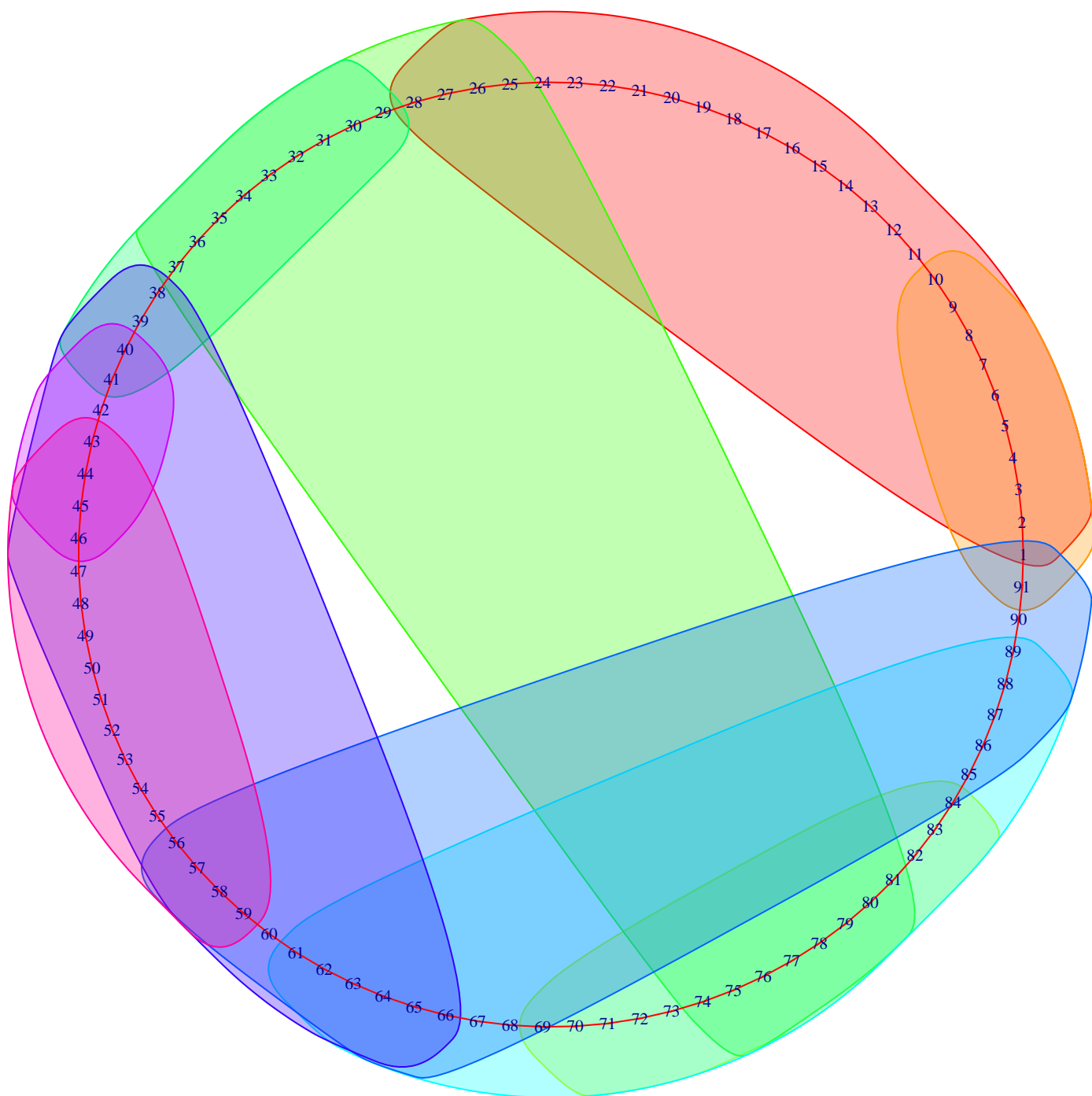

Supplement: Supplementary file 1 [file brainsci-09-00144-s001.zip › Supplementary 2/Mapper_graphs/917255_0B.pdf]

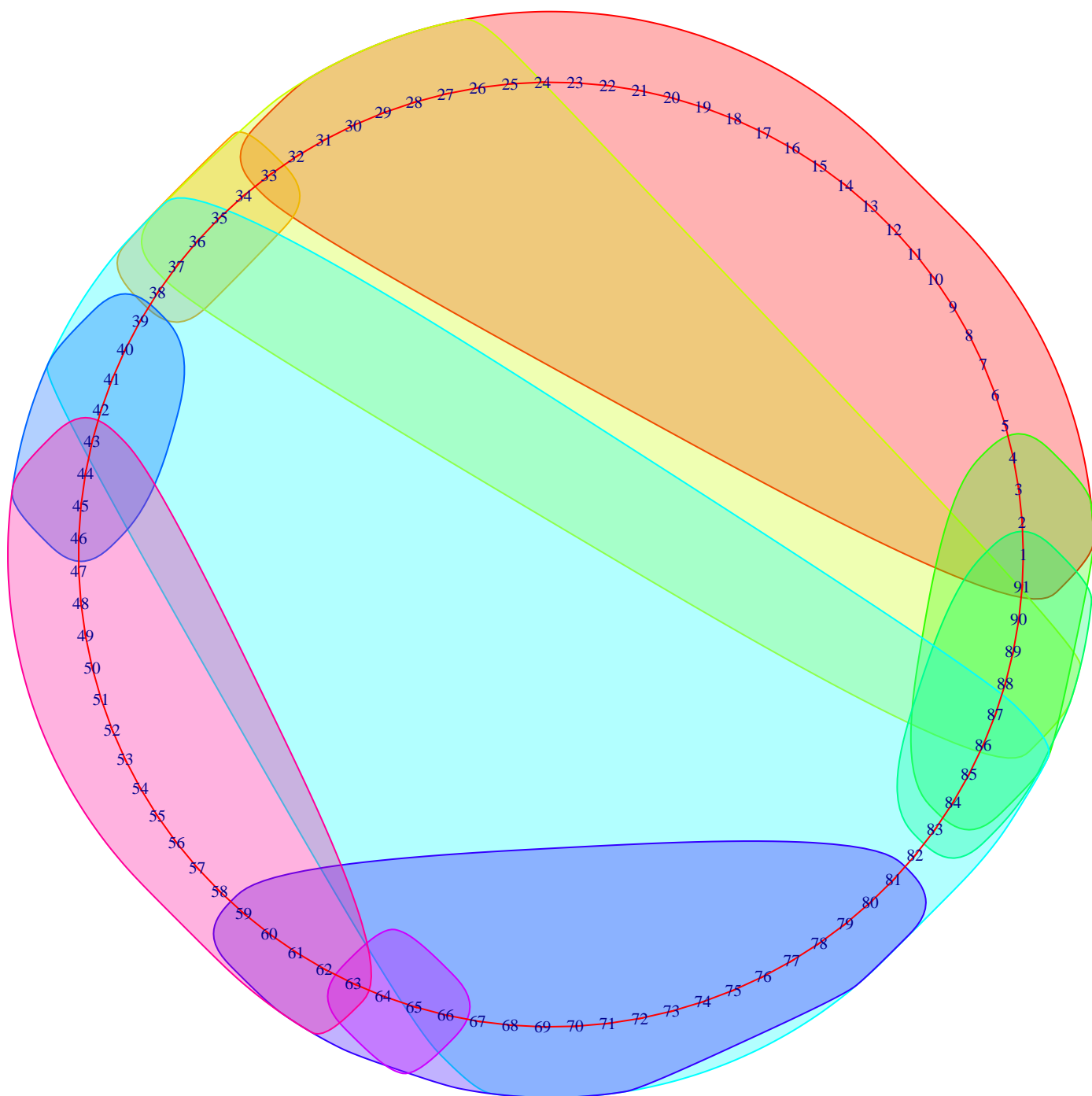

Supplement: Supplementary file 1 [file brainsci-09-00144-s001.zip › Supplementary 2/Mapper_graphs/177746_graph0B.pdf]

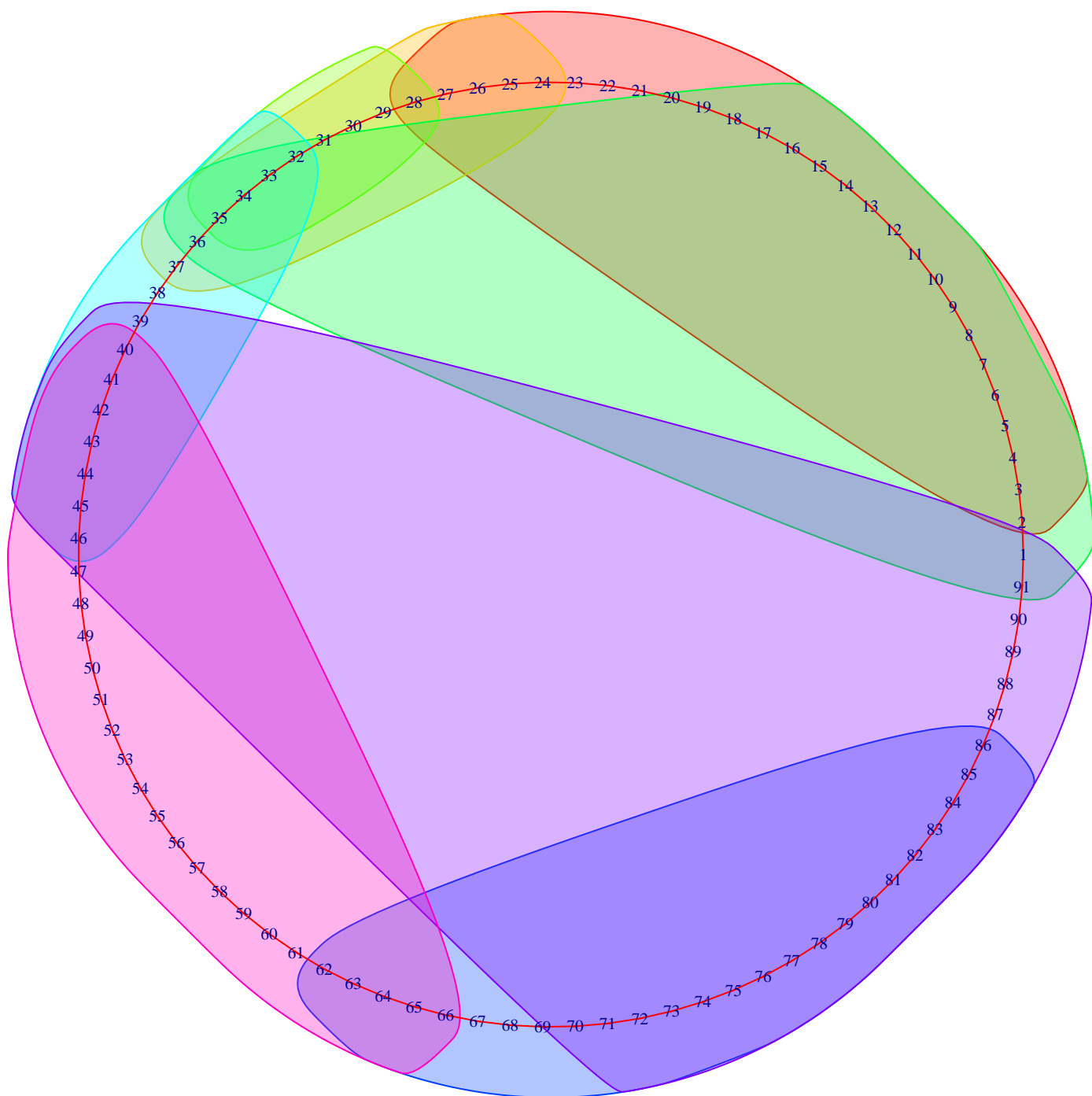

Supplement: Supplementary file 1 [file brainsci-09-00144-s001.zip › Supplementary 2/Mapper_graphs/599671_0B.pdf]

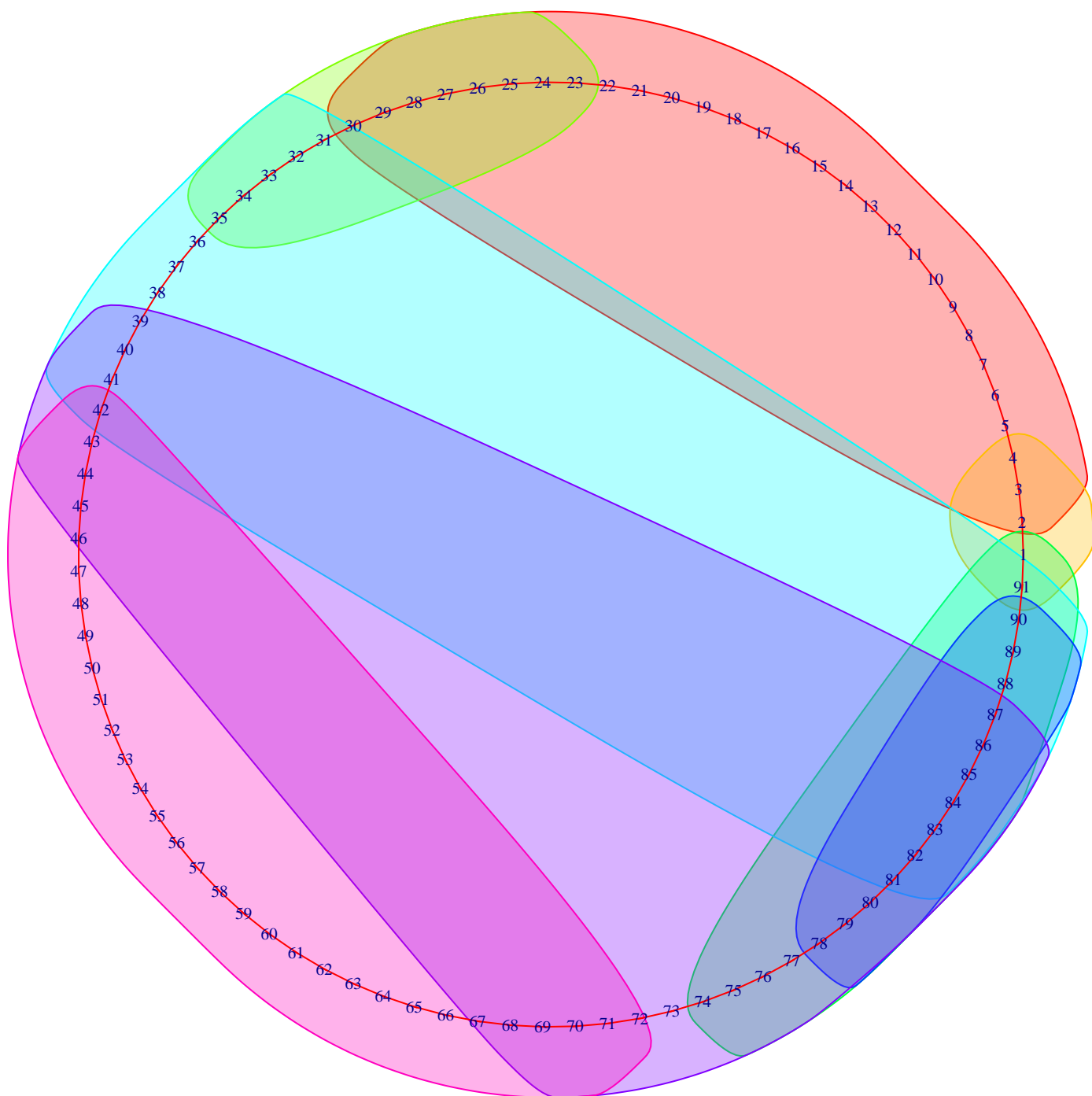

Supplement: Supplementary file 1 [file brainsci-09-00144-s001.zip › Supplementary 2/Mapper_graphs/662551_2B.pdf]

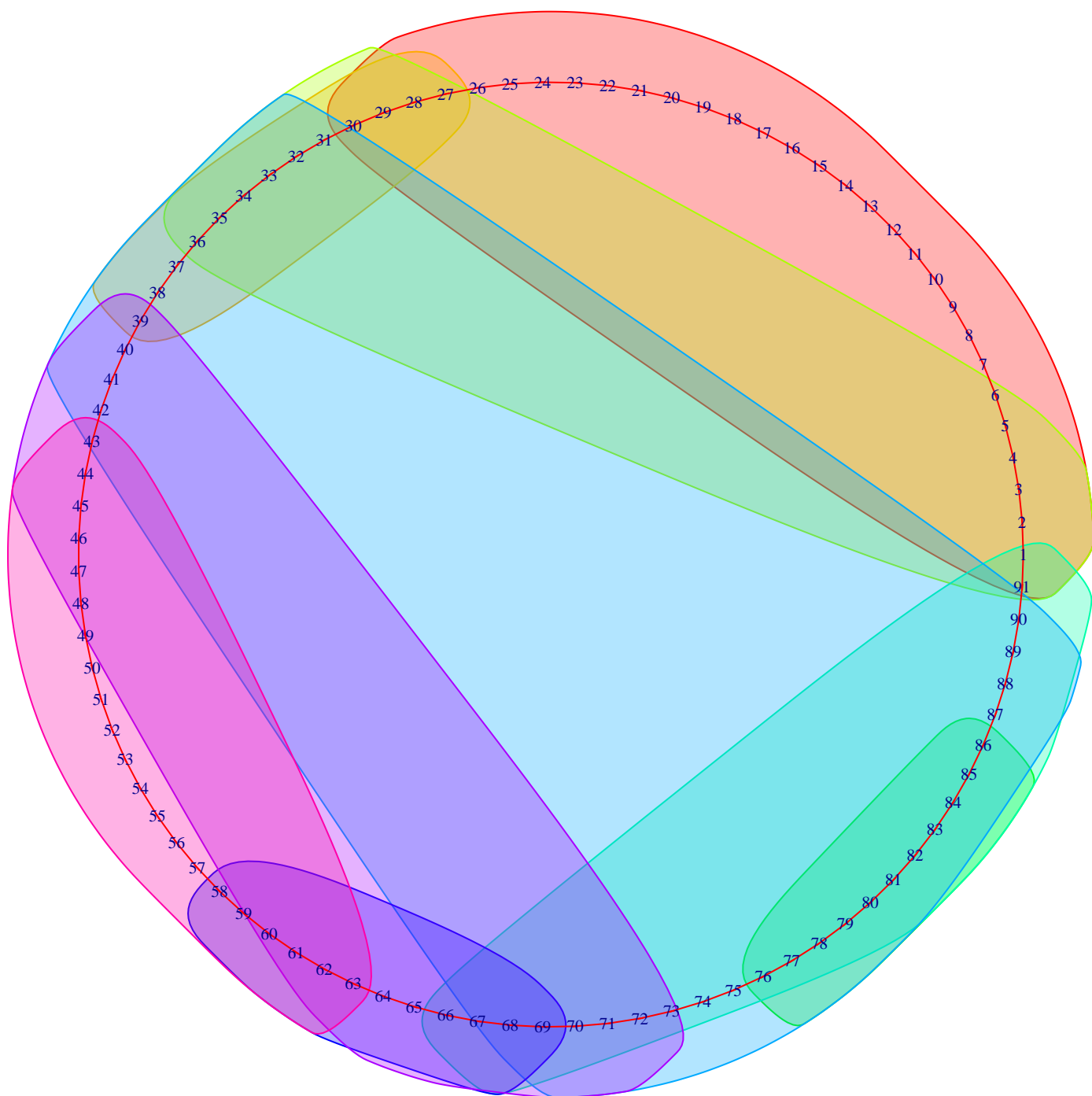

Supplement: Supplementary file 1 [file brainsci-09-00144-s001.zip › Supplementary 2/Mapper_graphs/162935_graph0B.pdf]

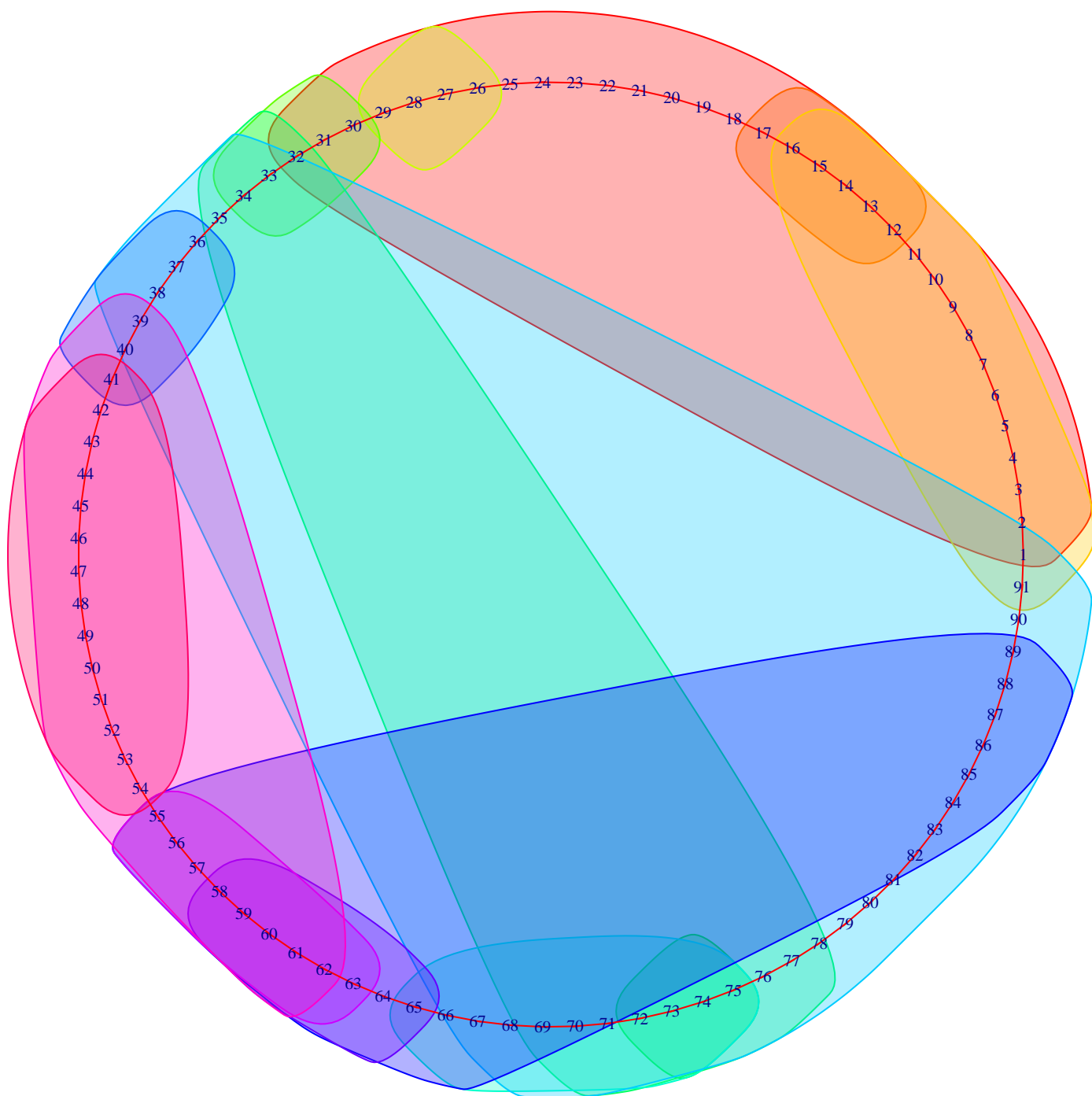

Supplement: Supplementary file 1 [file brainsci-09-00144-s001.zip › Supplementary 2/Mapper_graphs/189349_2B.pdf]

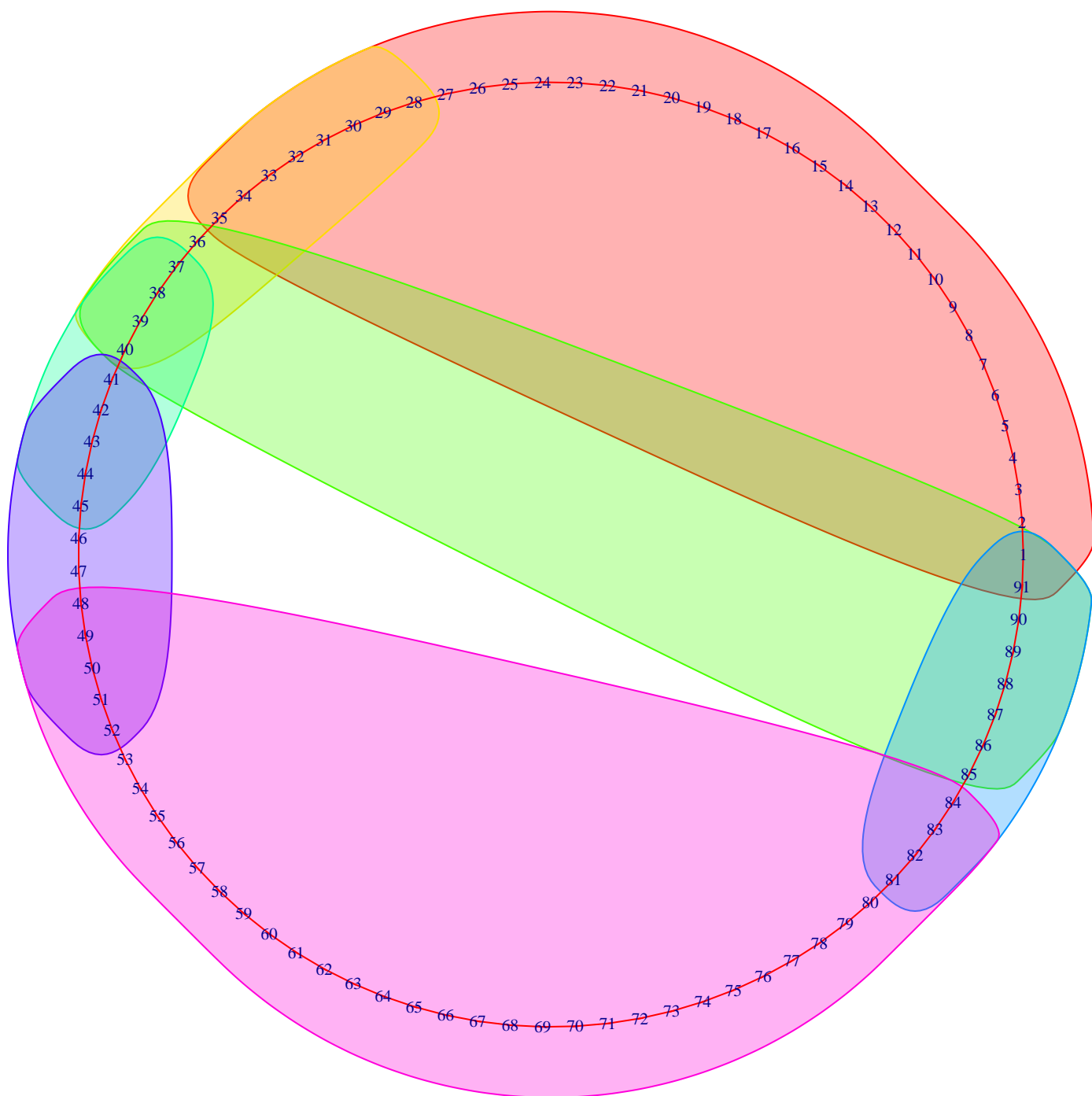

Supplement: Supplementary file 1 [file brainsci-09-00144-s001.zip › Supplementary 2/Mapper_graphs/125525_graph0B.pdf]

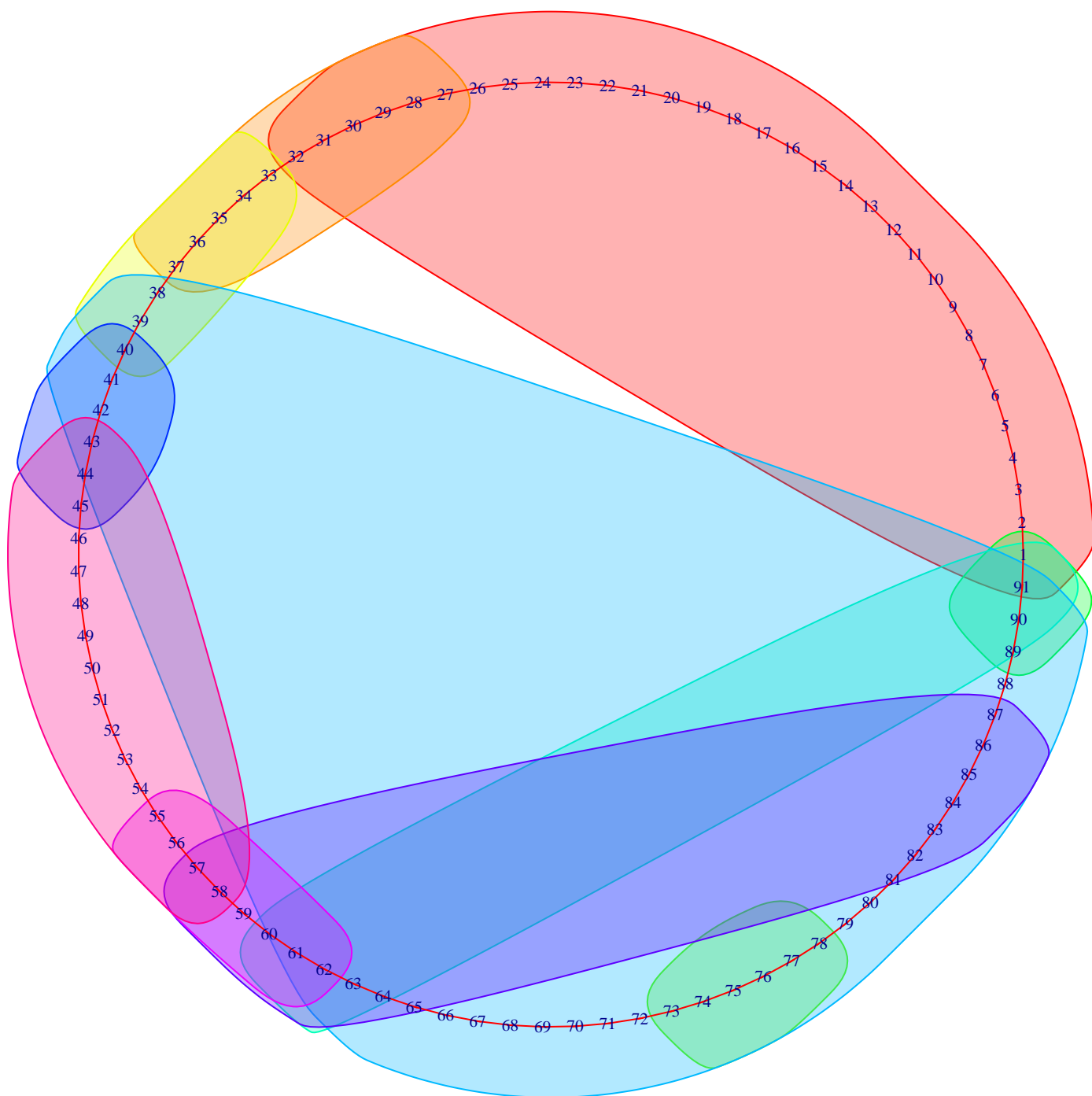

Supplement: Supplementary file 1 [file brainsci-09-00144-s001.zip › Supplementary 2/Mapper_graphs/164636_graph2B.pdf]

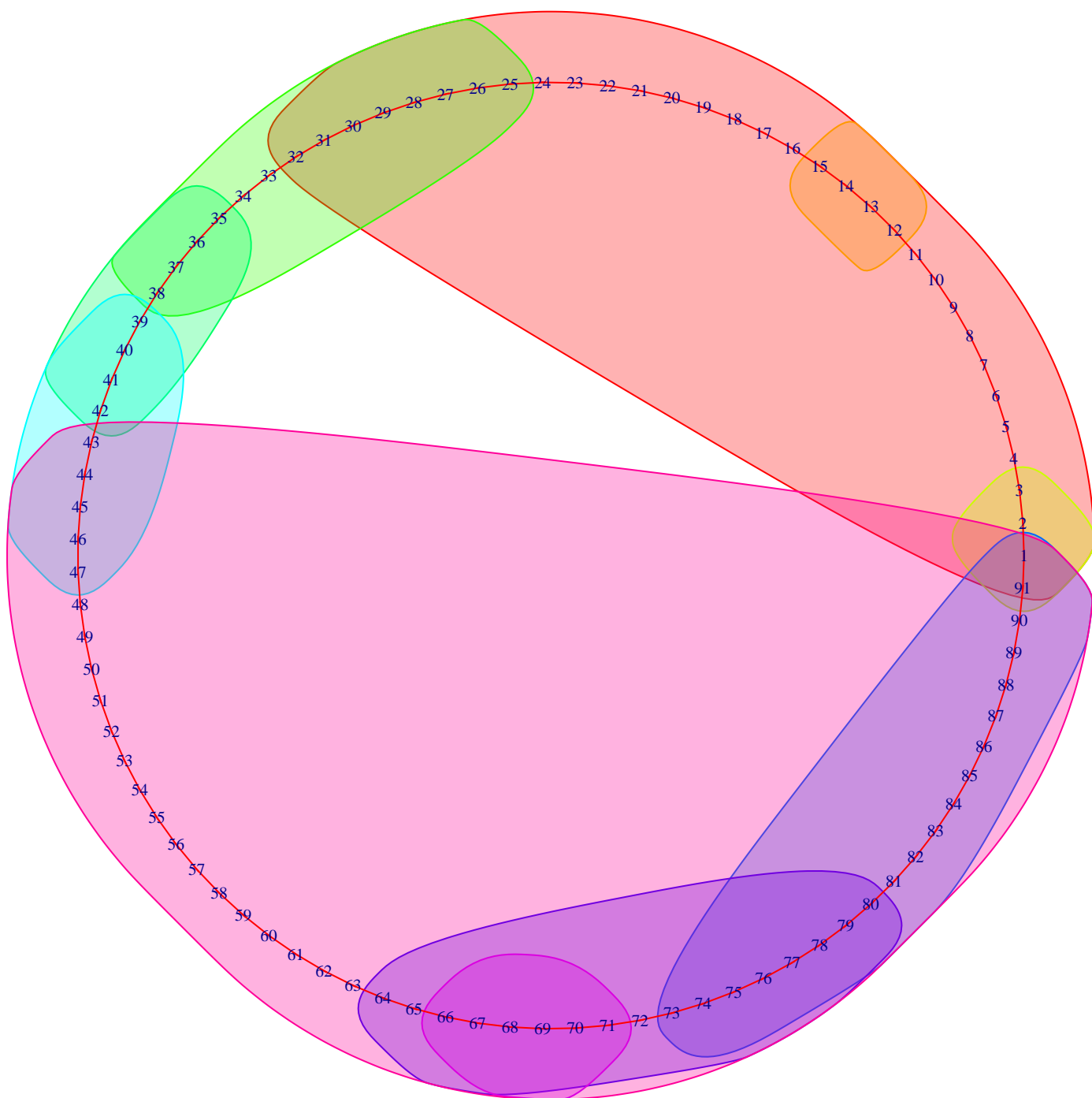

Supplement: Supplementary file 1 [file brainsci-09-00144-s001.zip › Supplementary 2/Mapper_graphs/706040_0B.pdf]

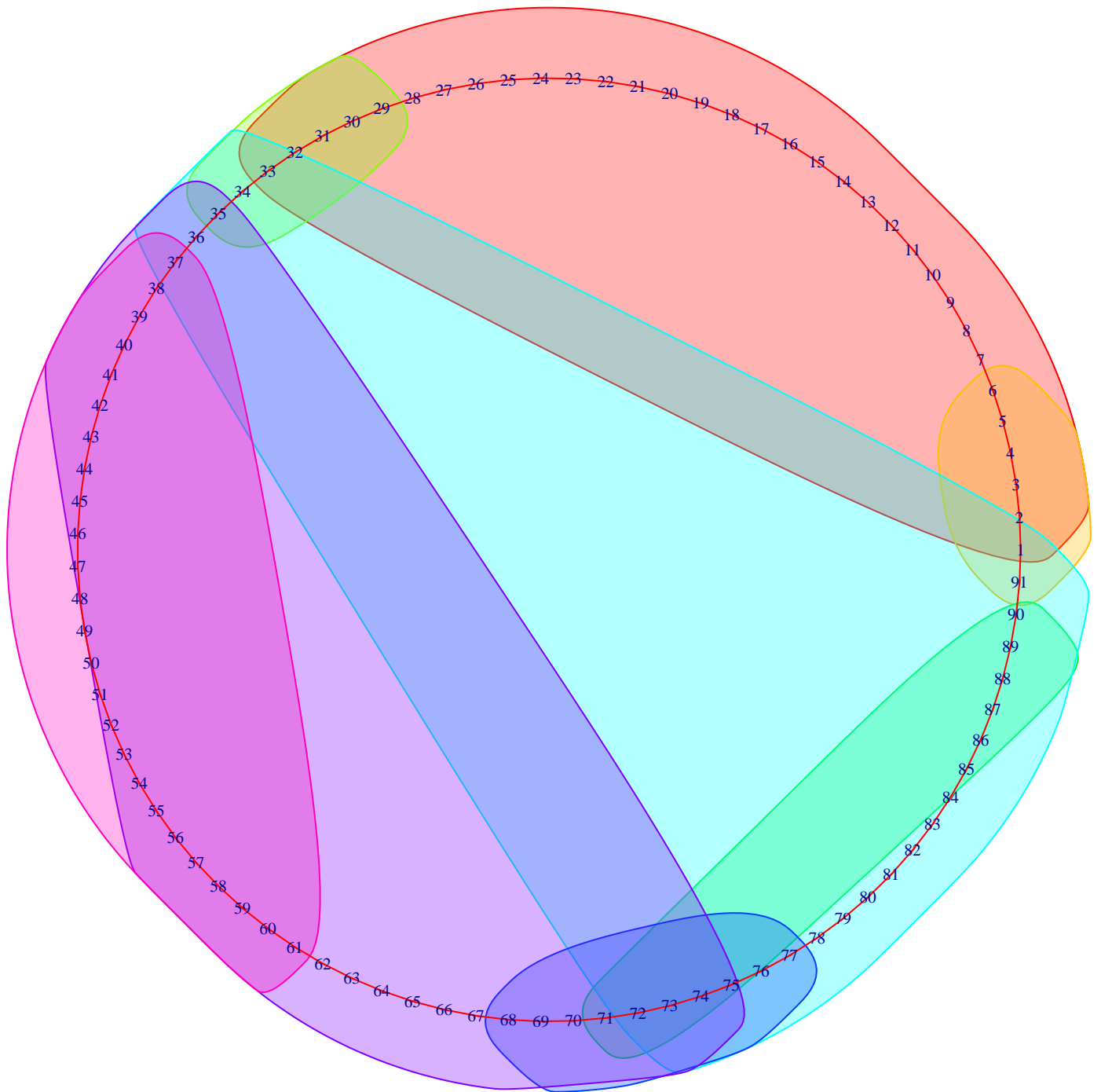

Supplement: Supplementary file 1 [file brainsci-09-00144-s001.zip › Supplementary 2/Mapper_graphs/433839_graph0B.pdf]

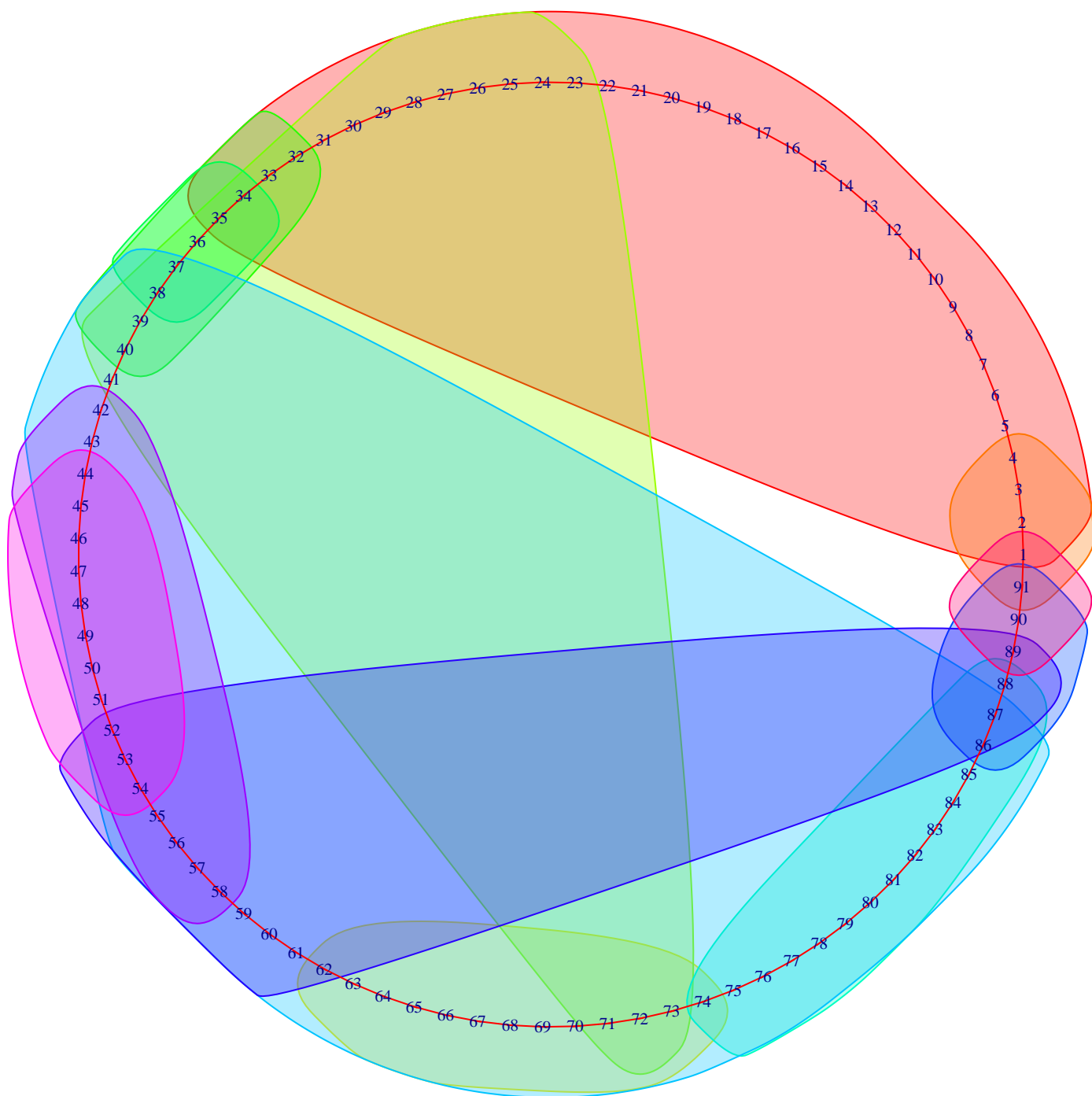

Supplement: Supplementary file 1 [file brainsci-09-00144-s001.zip › Supplementary 2/Mapper_graphs/214524_0B.pdf]

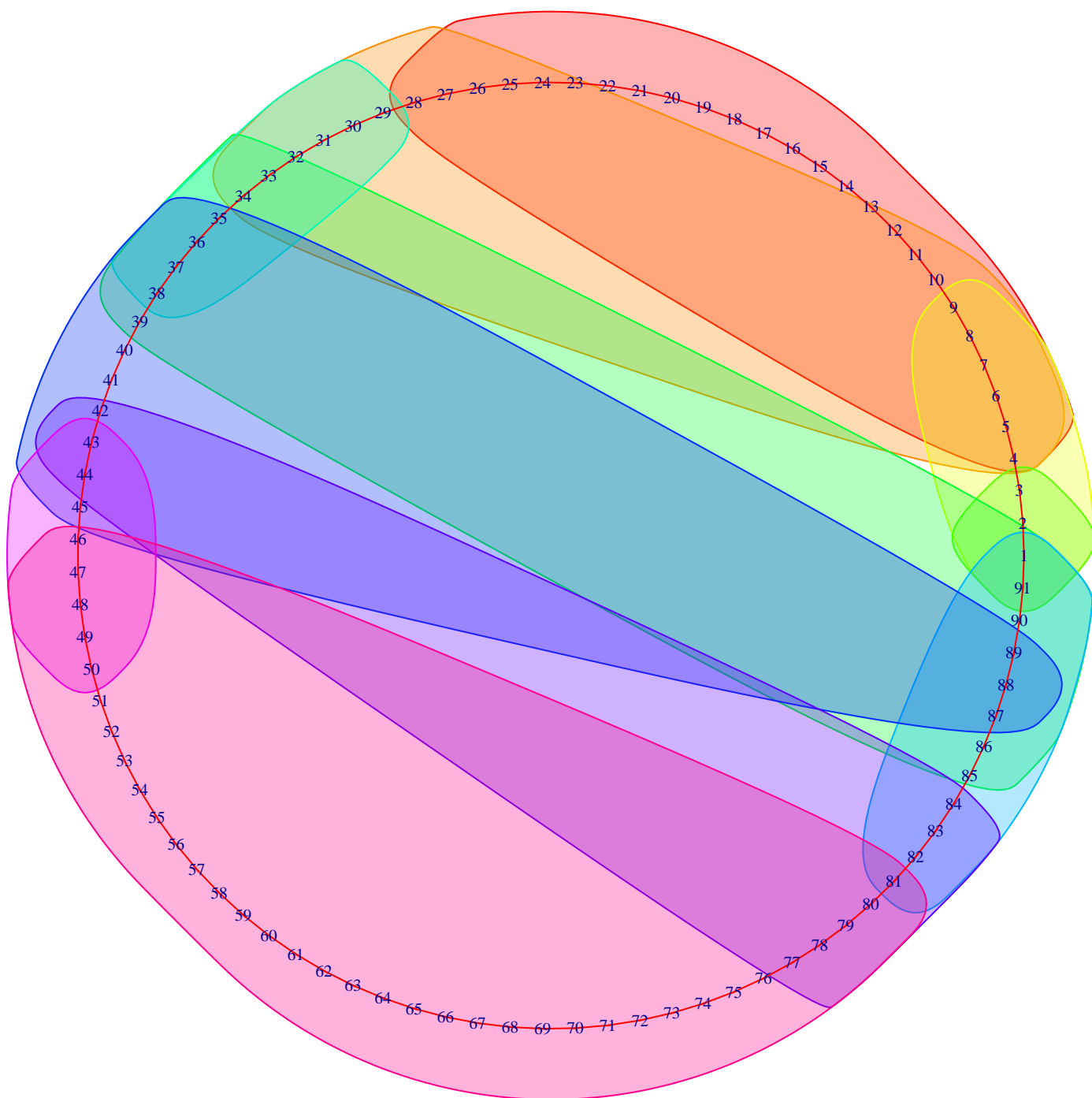

Supplement: Supplementary file 1 [file brainsci-09-00144-s001.zip › Supplementary 2/Mapper_graphs/255639_2B.pdf]

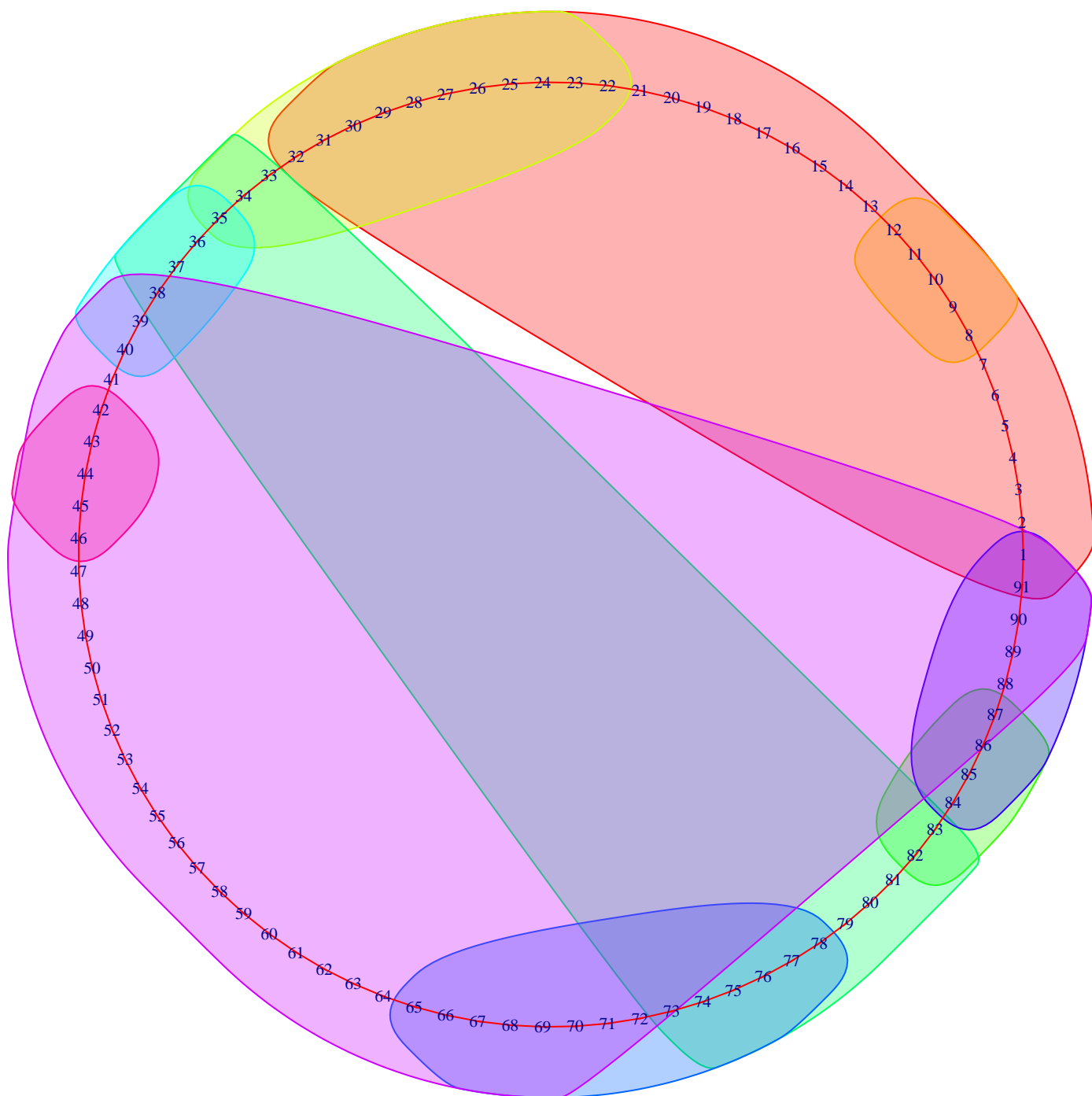

Supplement: Supplementary file 1 [file brainsci-09-00144-s001.zip › Supplementary 2/Mapper_graphs/898176_0B.pdf]

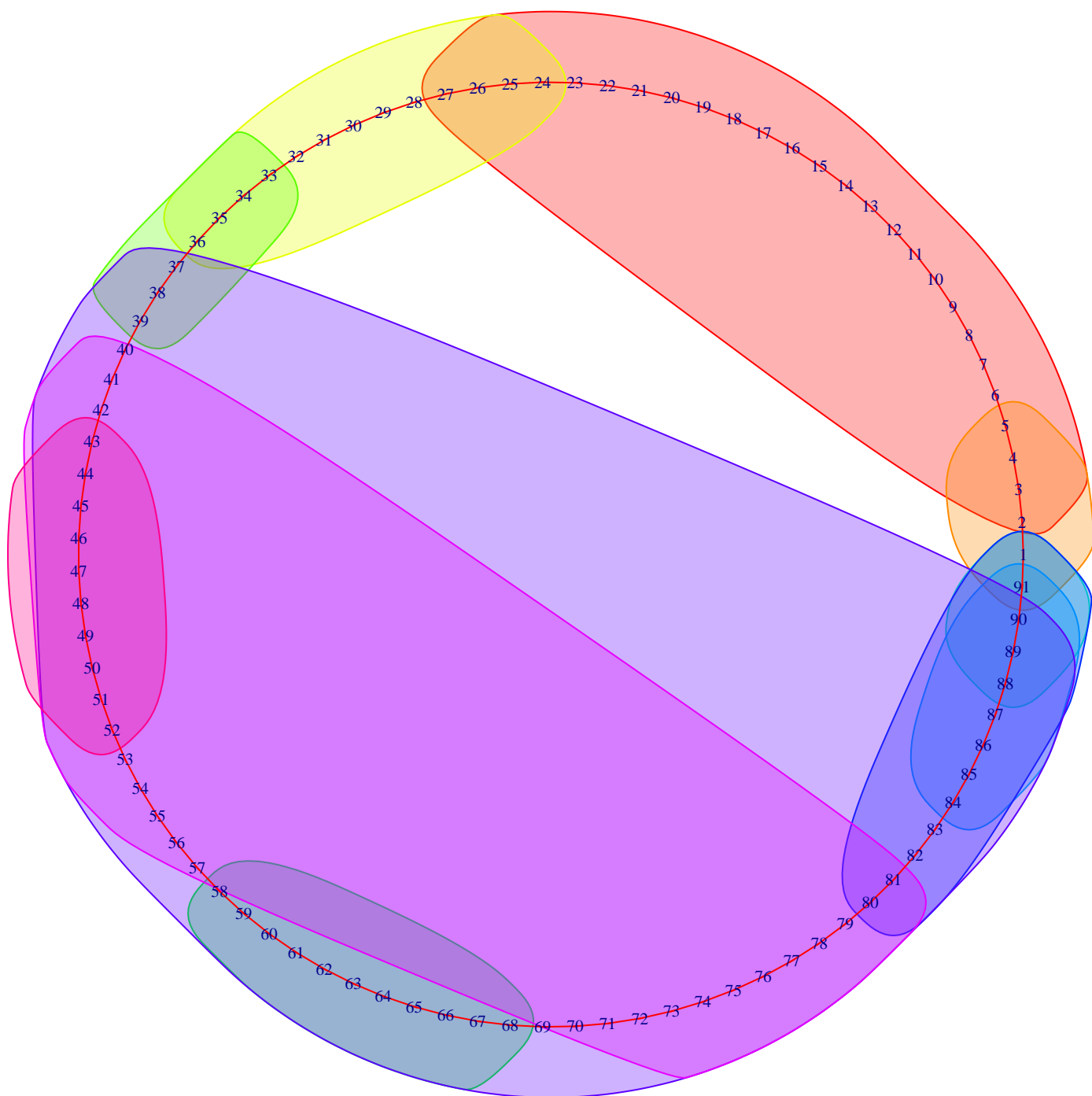

Supplement: Supplementary file 1 [file brainsci-09-00144-s001.zip › Supplementary 2/Mapper_graphs/990366_2B.pdf]

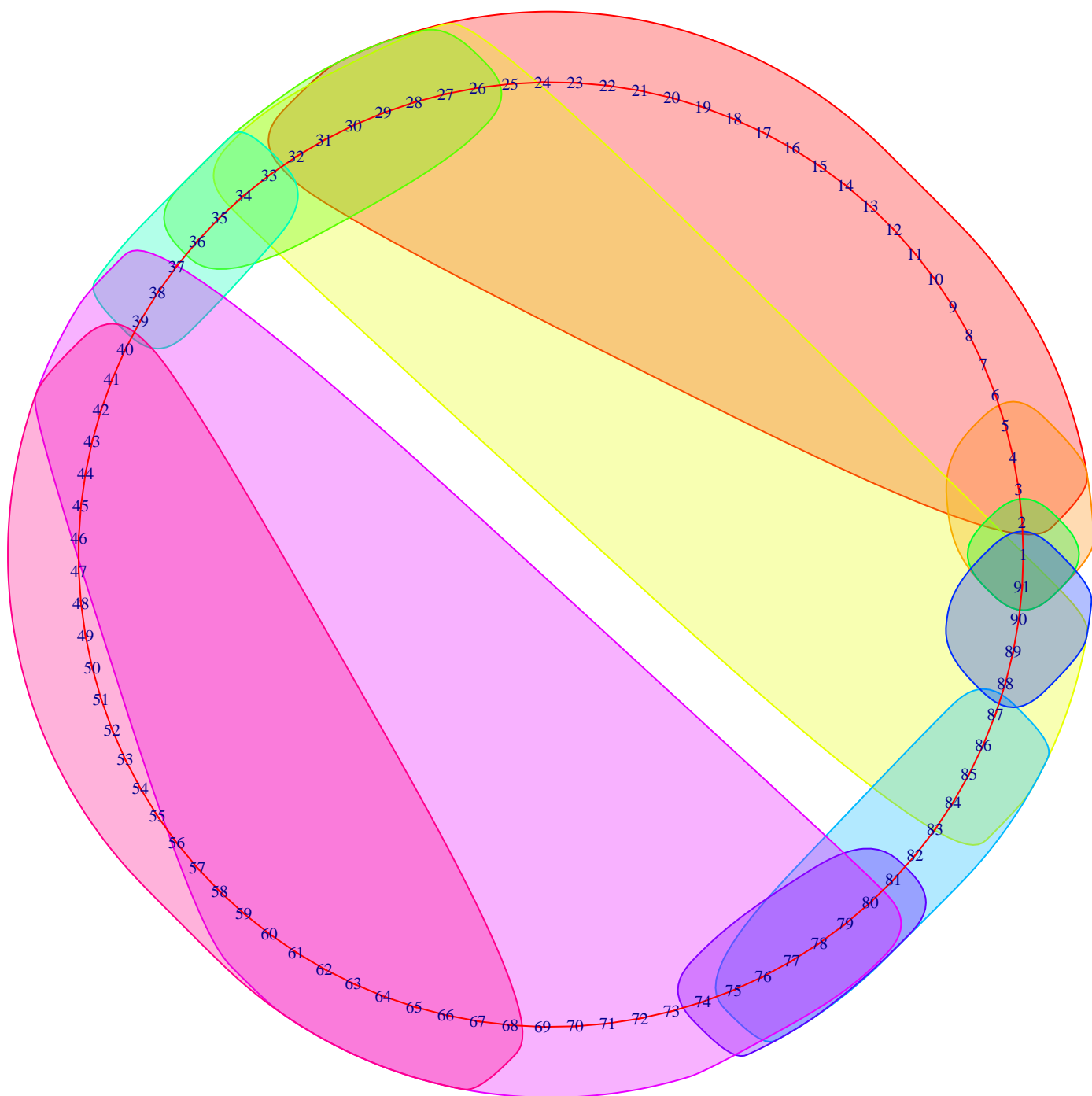

Supplement: Supplementary file 1 [file brainsci-09-00144-s001.zip › Supplementary 2/Mapper_graphs/149741_graph0B.pdf]

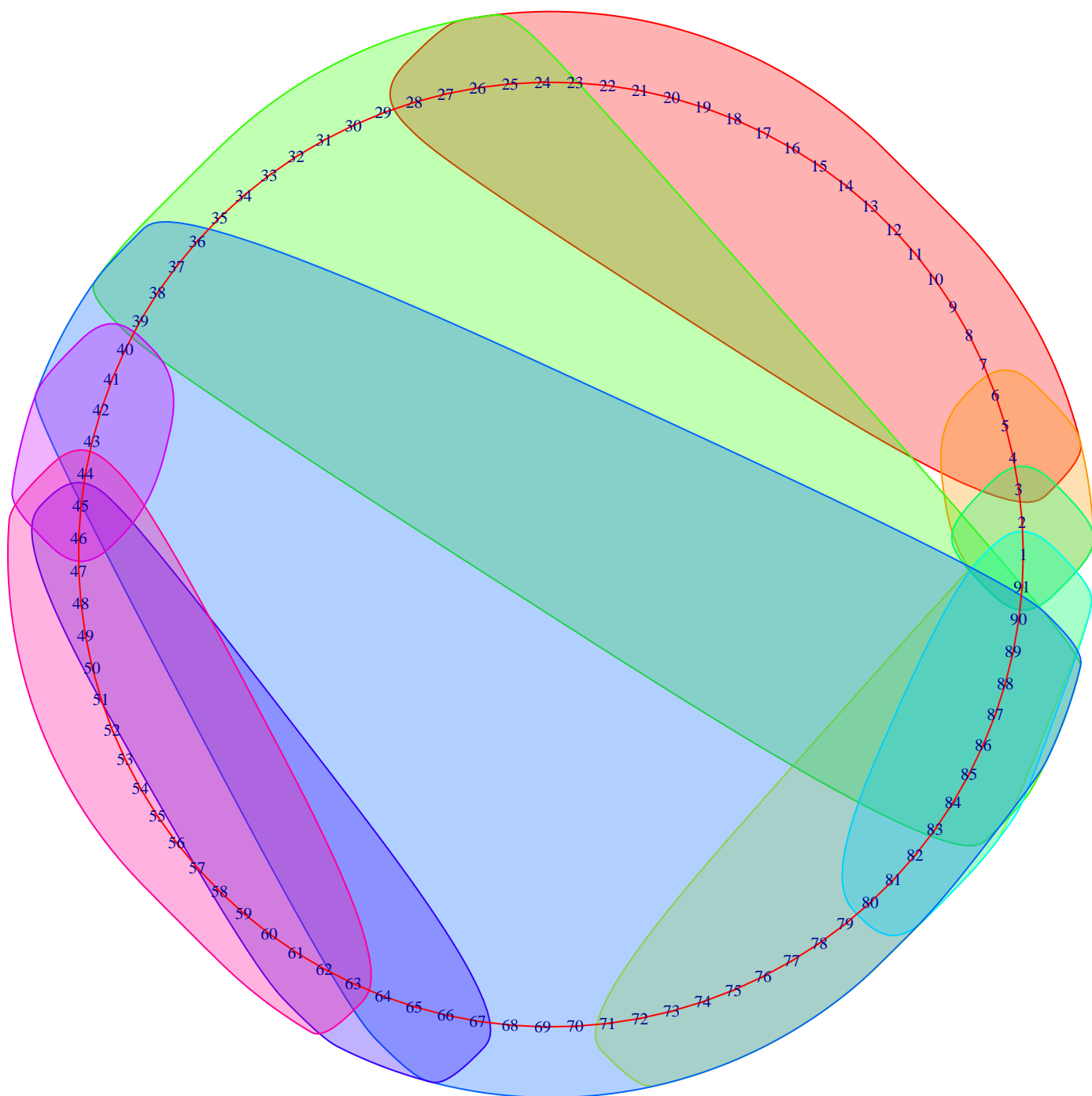

Supplement: Supplementary file 1 [file brainsci-09-00144-s001.zip › Supplementary 2/Mapper_graphs/212318_0B.pdf]

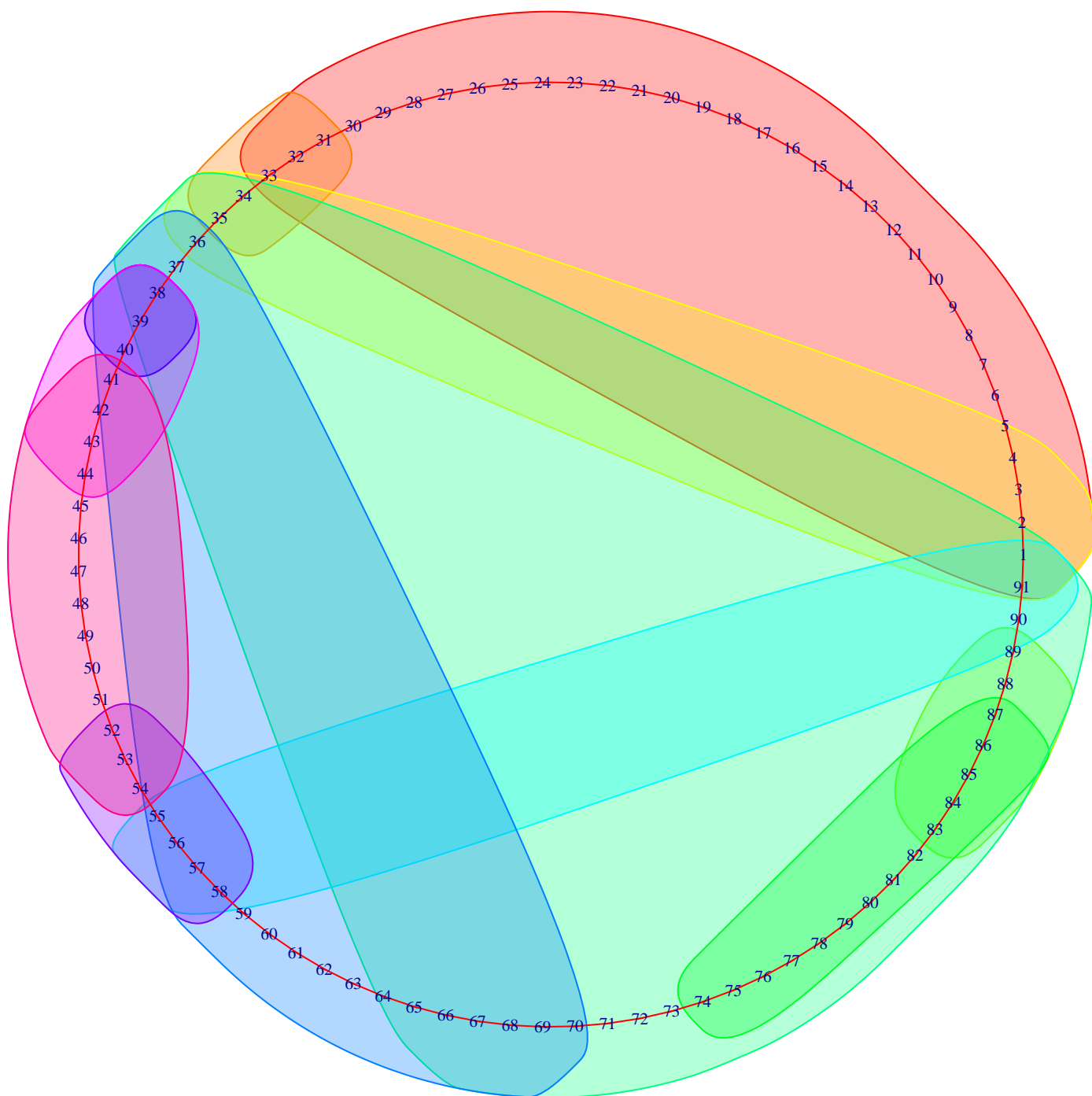

Supplement: Supplementary file 1 [file brainsci-09-00144-s001.zip › Supplementary 2/Mapper_graphs/257845_0B.pdf]

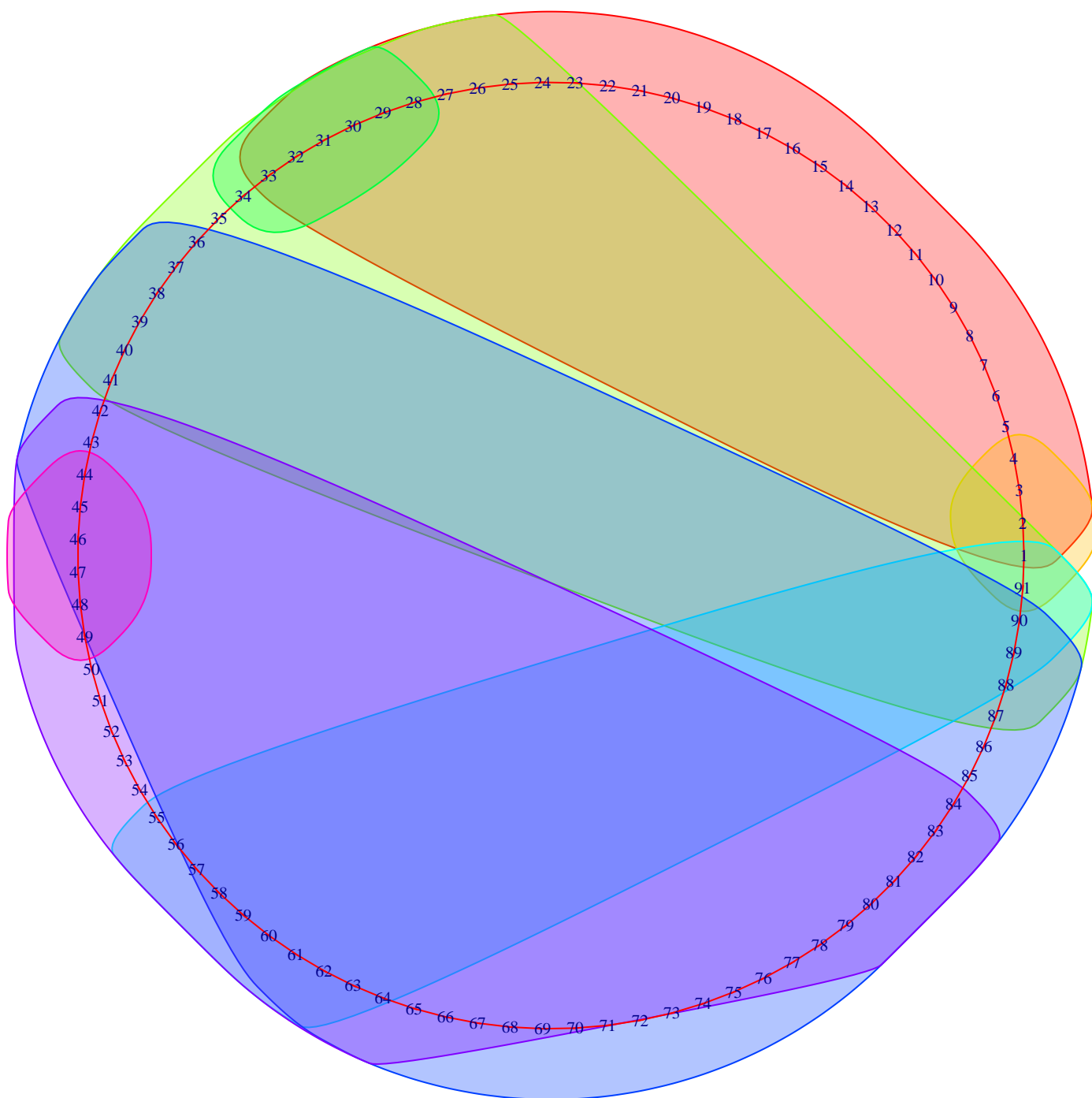

Supplement: Supplementary file 1 [file brainsci-09-00144-s001.zip › Supplementary 2/Mapper_graphs/100307_2B.pdf]

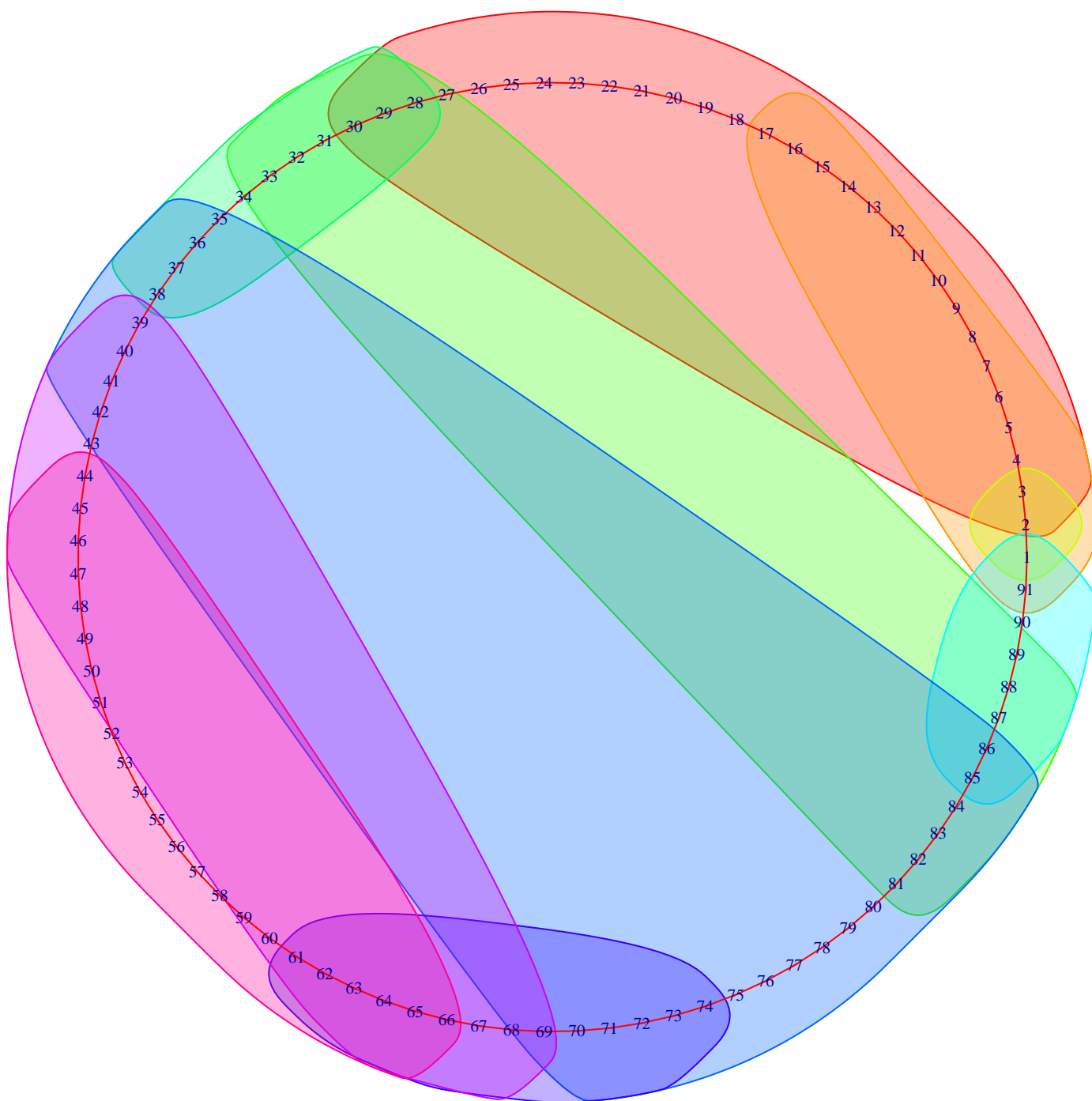

Supplement: Supplementary file 1 [file brainsci-09-00144-s001.zip › Supplementary 2/Mapper_graphs/707749_2B.pdf]

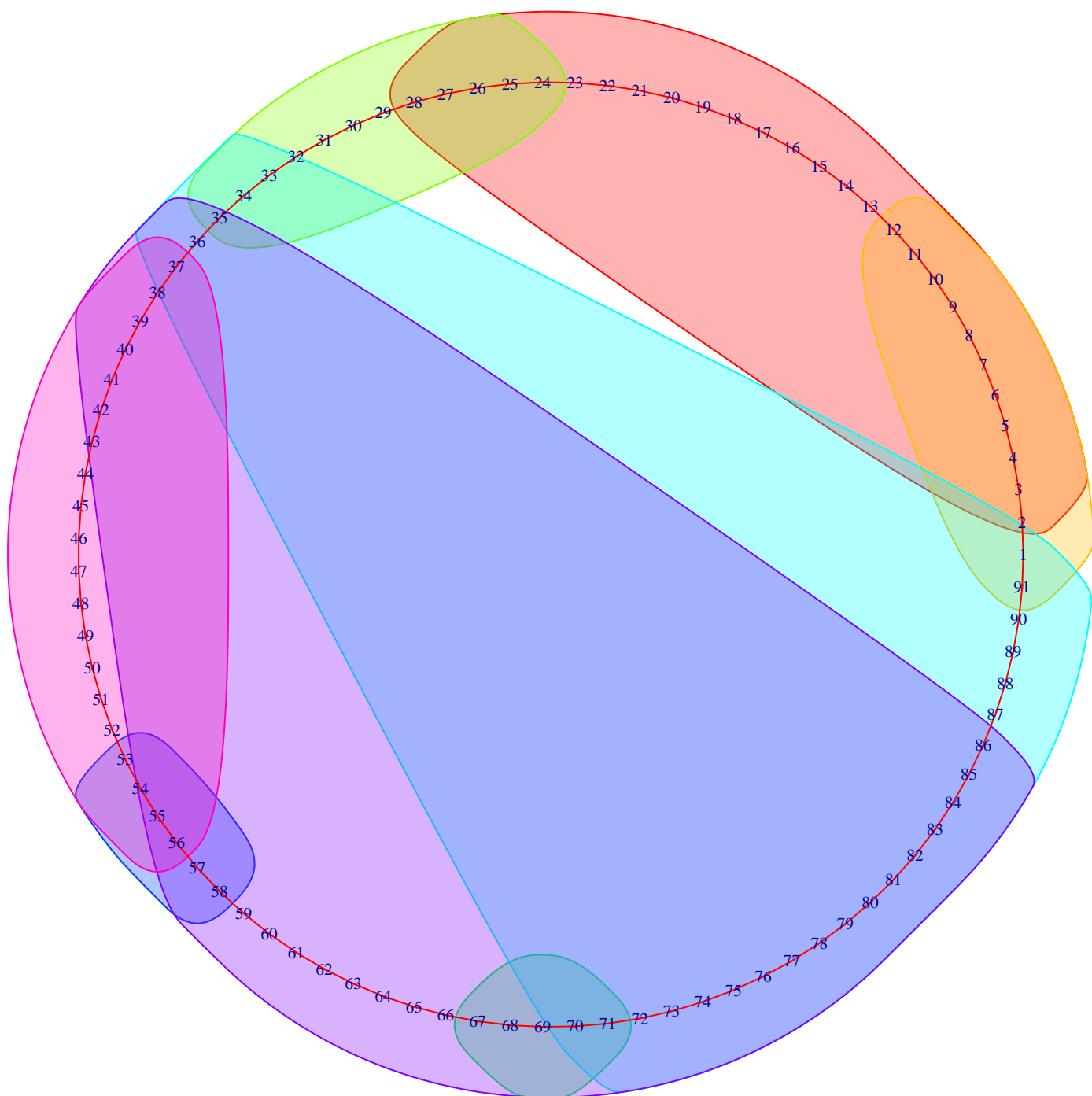

Supplement: Supplementary file 1 [file brainsci-09-00144-s001.zip › Supplementary 2/Mapper_graphs/872764_2B.pdf]

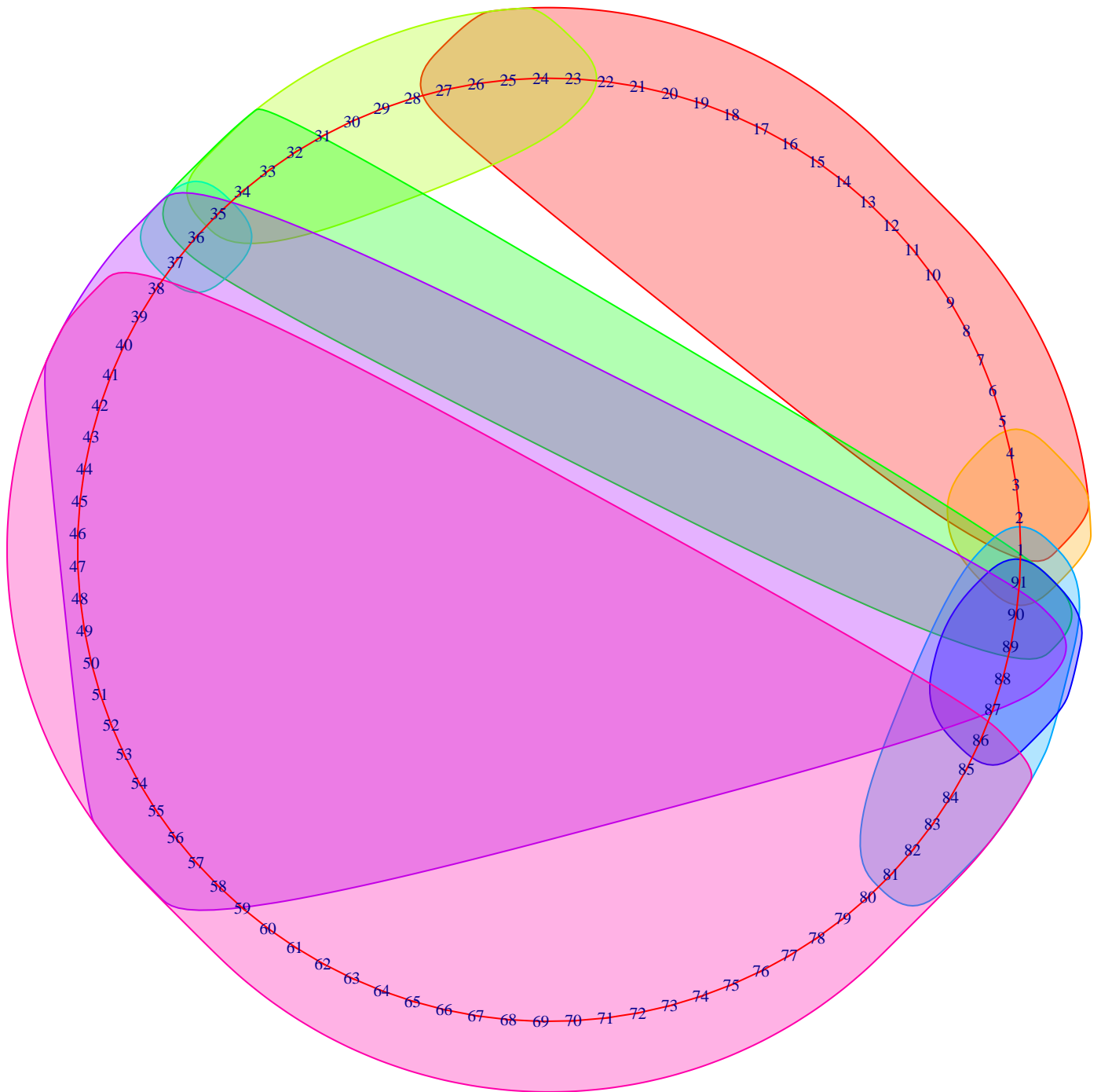

Supplement: Supplementary file 1 [file brainsci-09-00144-s001.zip › Supplementary 2/Mapper_graphs/102816_2B.pdf]

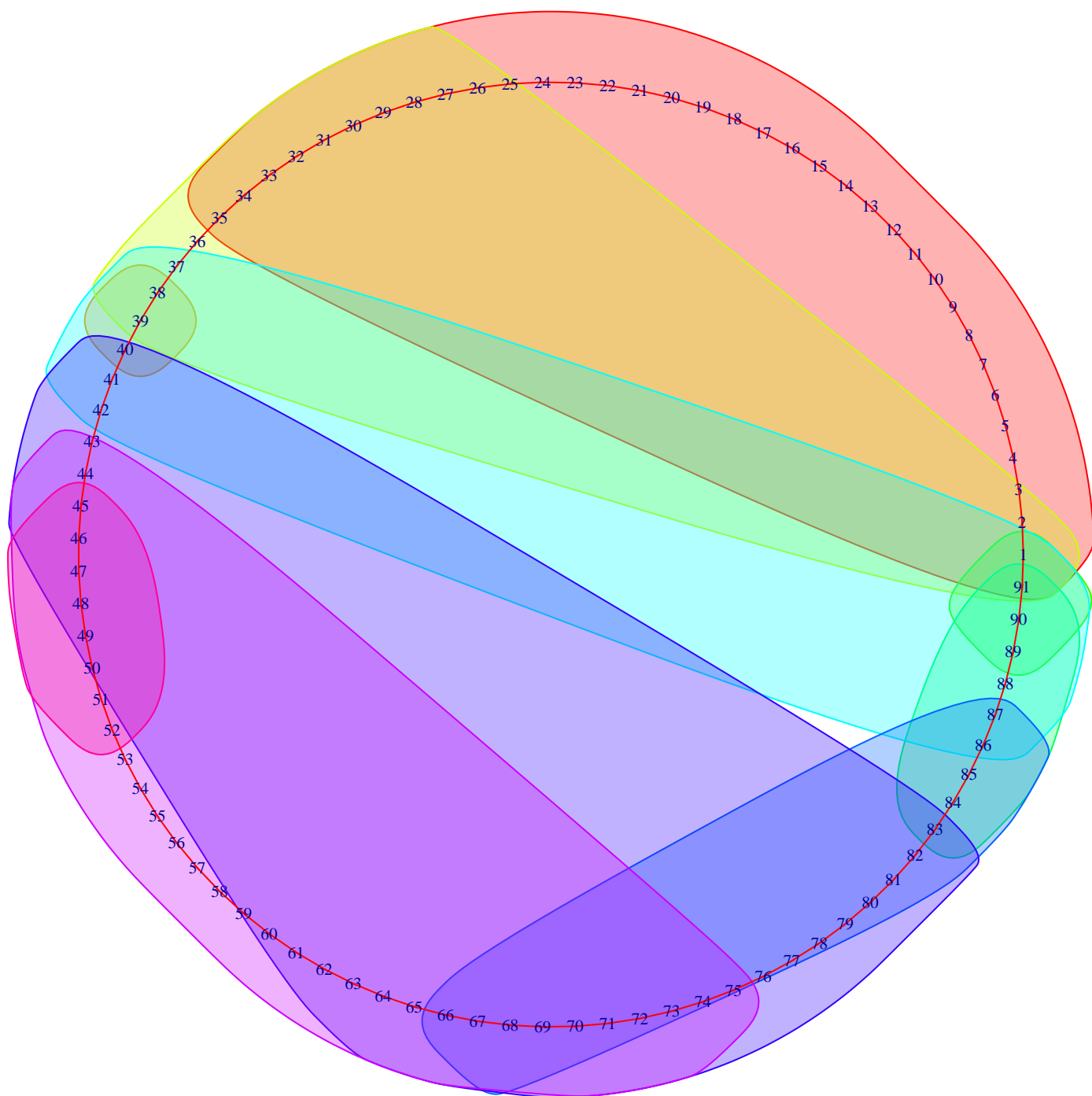

Supplement: Supplementary file 1 [file brainsci-09-00144-s001.zip › Supplementary 2/Mapper_graphs/198653_2B.pdf]

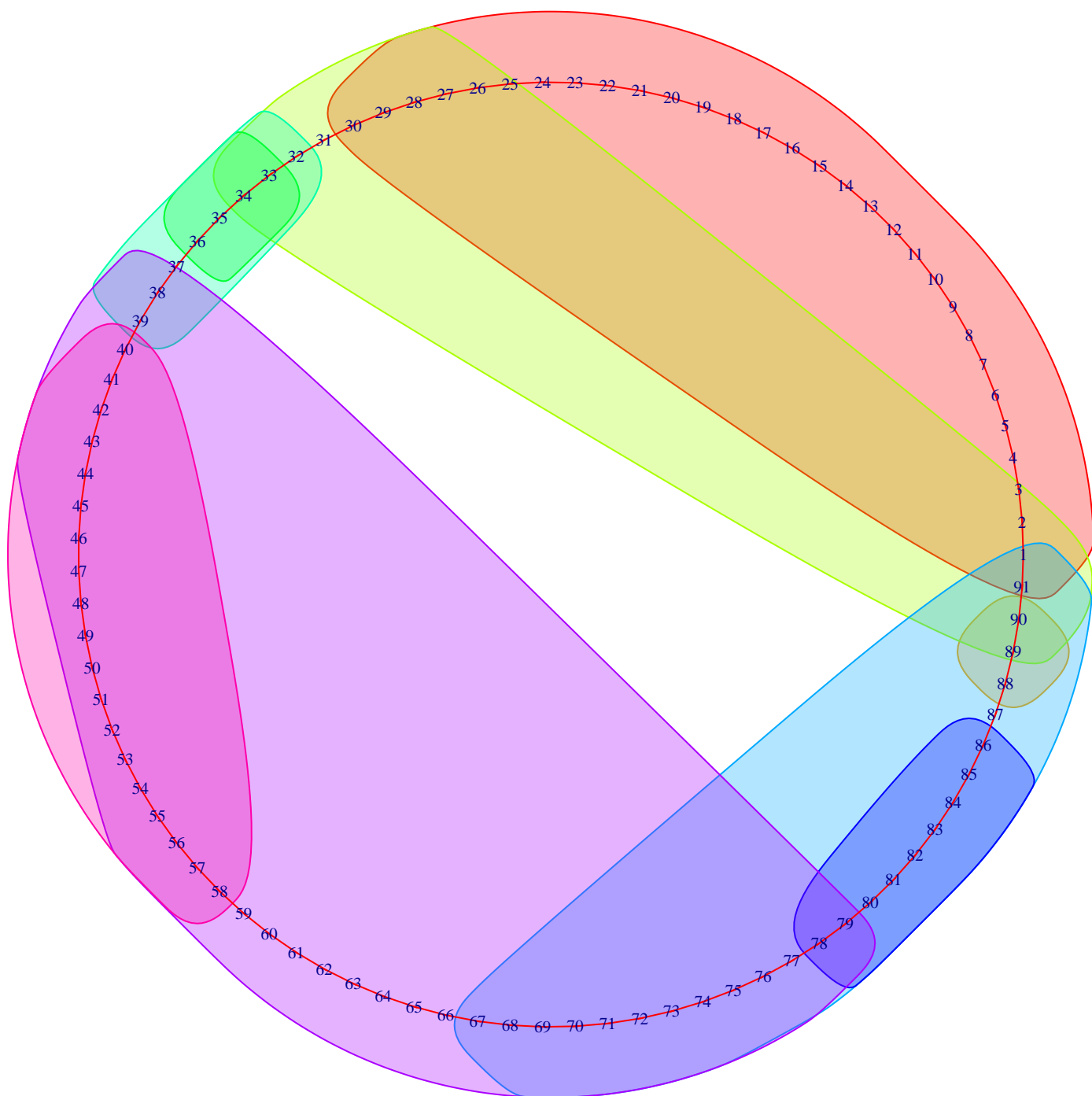

Supplement: Supplementary file 1 [file brainsci-09-00144-s001.zip › Supplementary 2/Mapper_graphs/200109_0B.pdf]

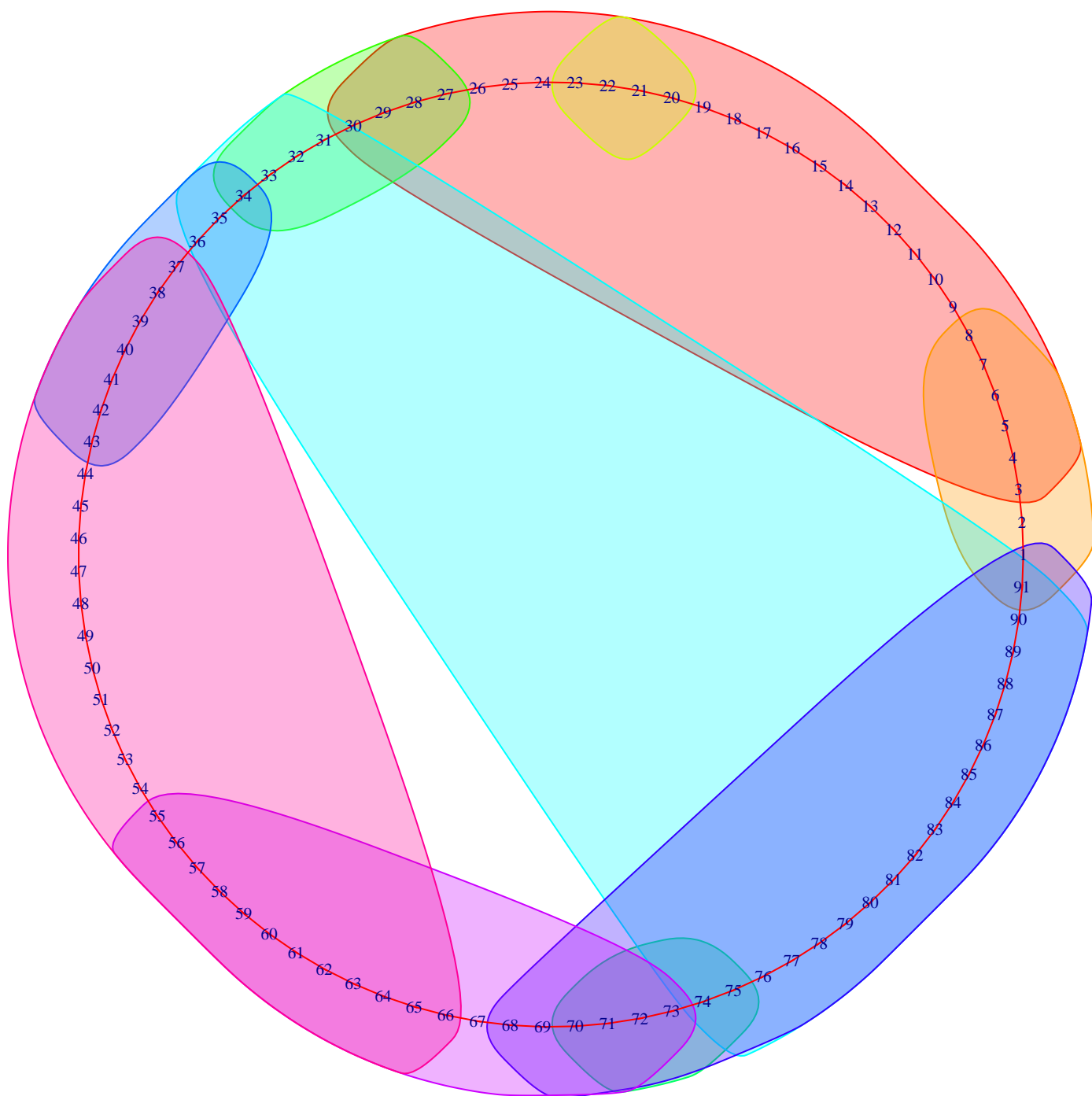

Supplement: Supplementary file 1 [file brainsci-09-00144-s001.zip › Supplementary 2/Mapper_graphs/680957_2B.pdf]

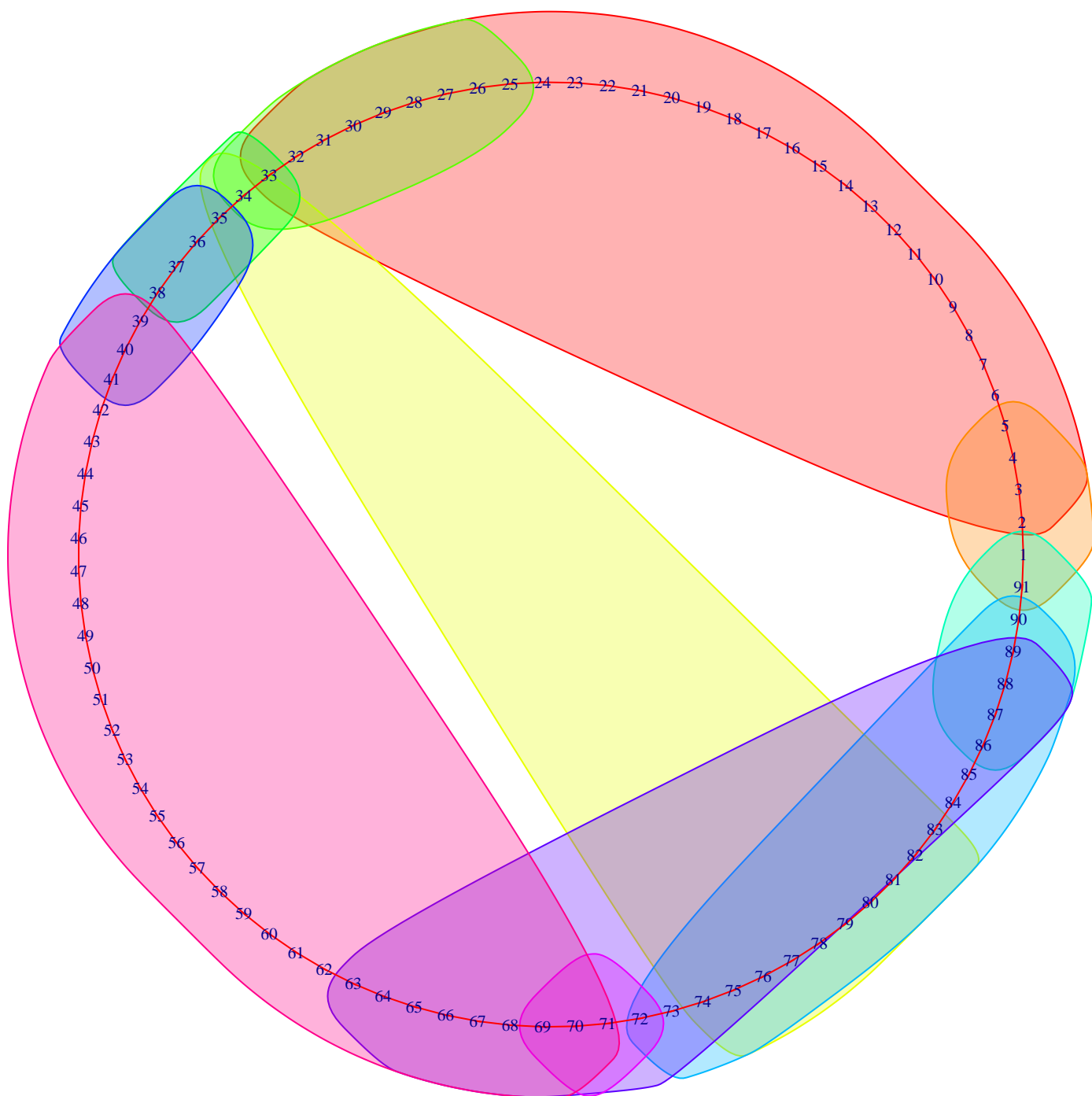

Supplement: Supplementary file 1 [file brainsci-09-00144-s001.zip › Supplementary 2/Mapper_graphs/814649_2B.pdf]

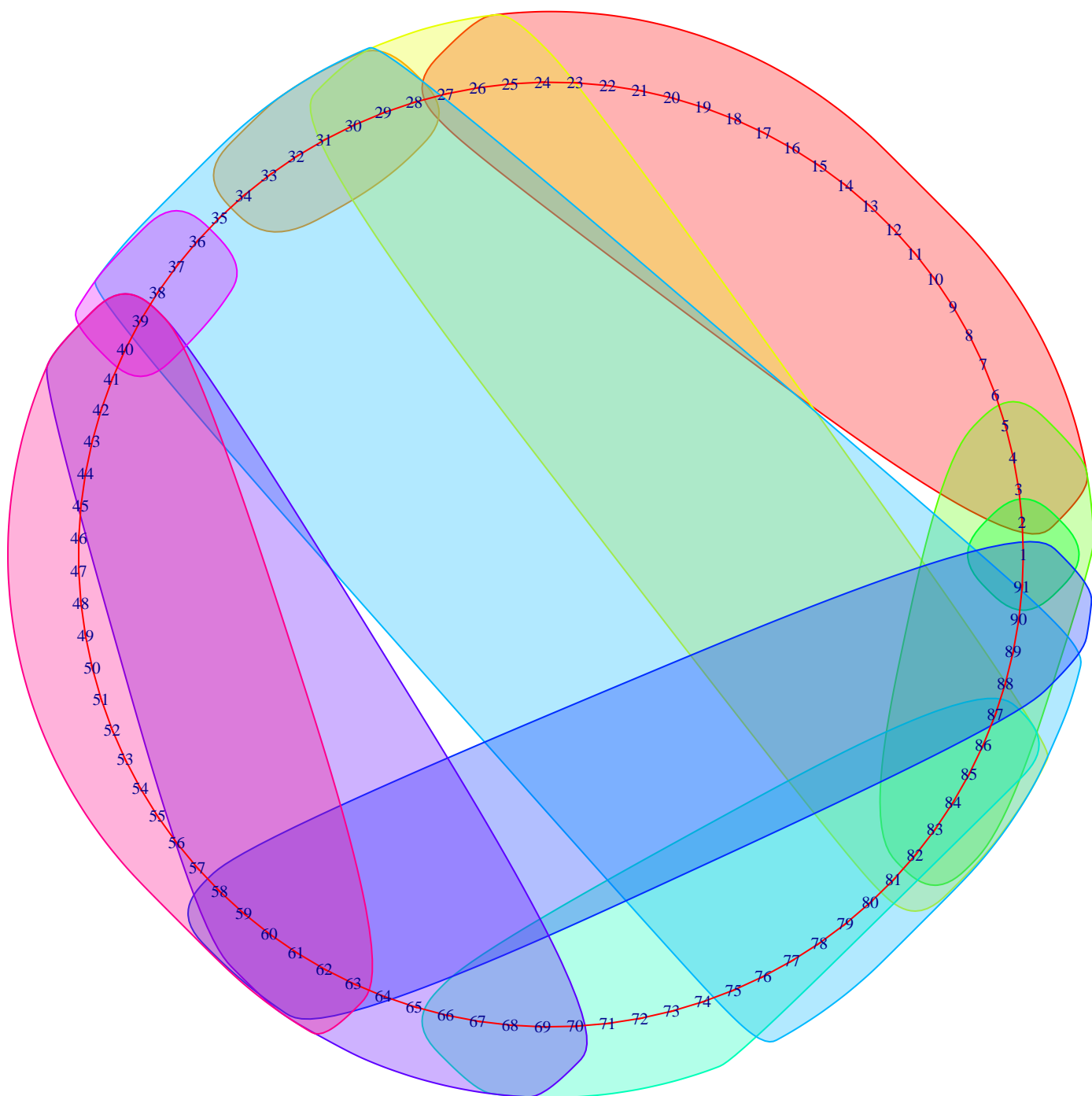

Supplement: Supplementary file 1 [file brainsci-09-00144-s001.zip › Supplementary 2/Mapper_graphs/191437_0B.pdf]

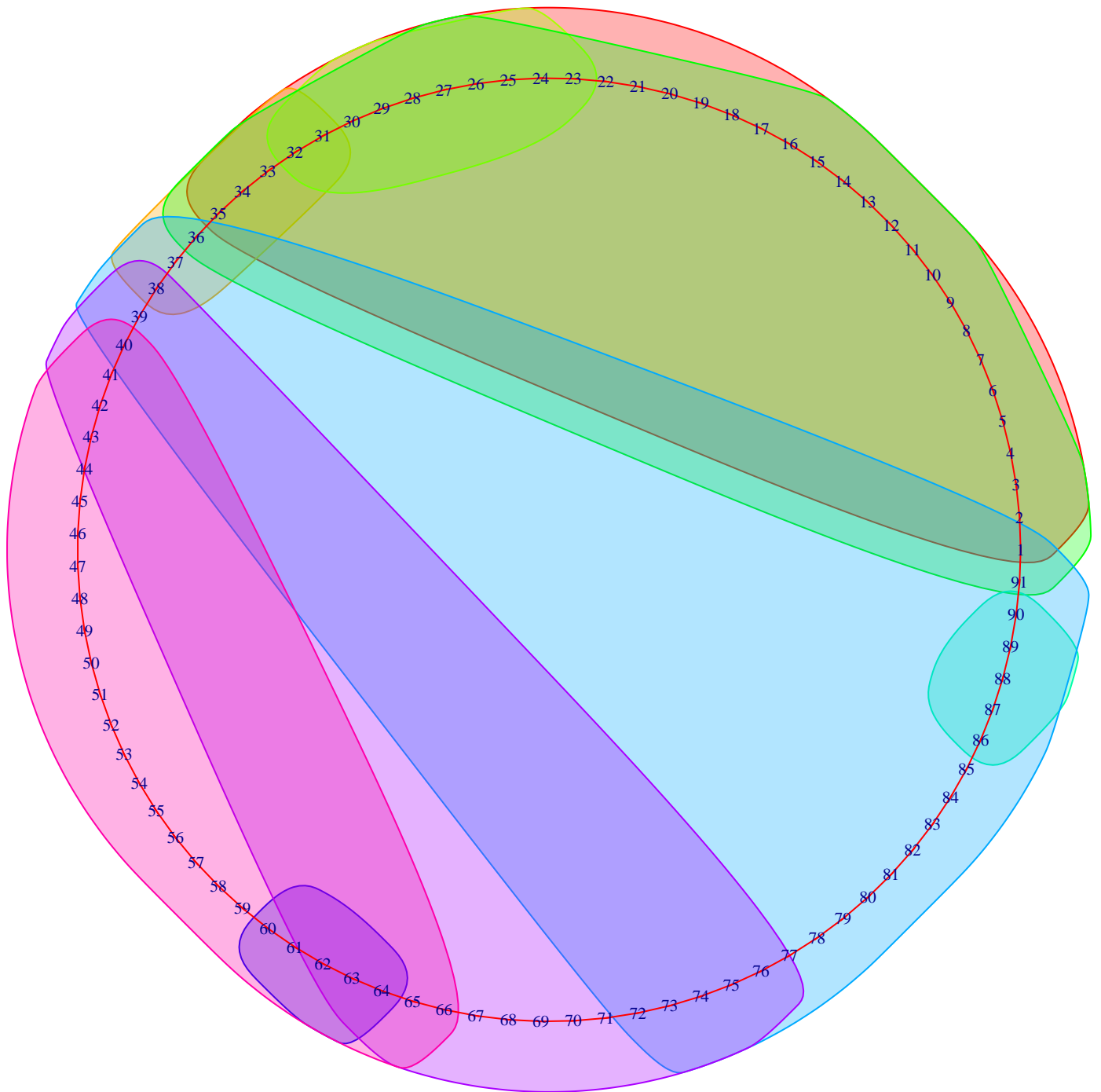

Supplement: Supplementary file 1 [file brainsci-09-00144-s001.zip › Supplementary 2/Mapper_graphs/715950_2B.pdf]

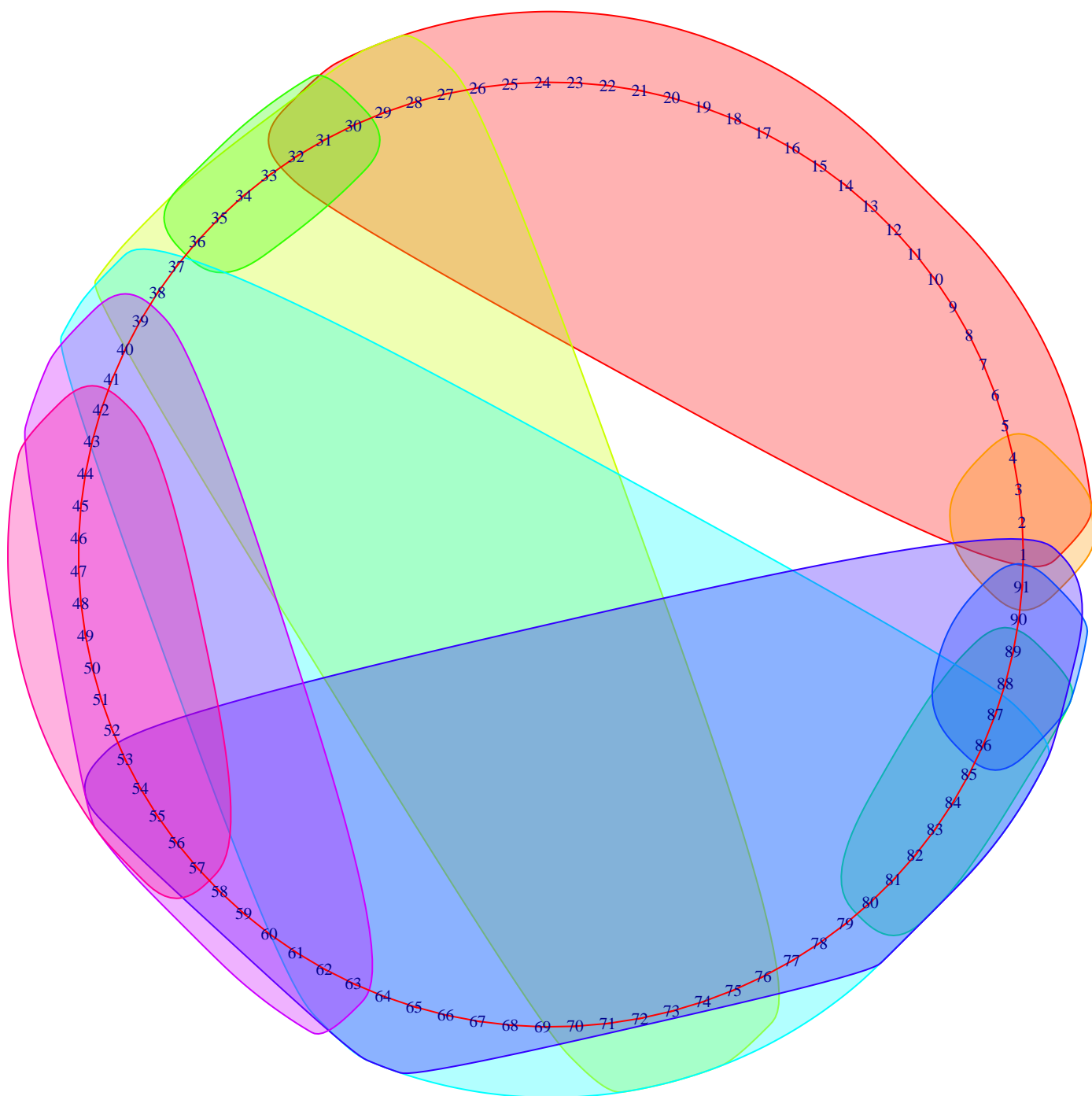

Supplement: Supplementary file 1 [file brainsci-09-00144-s001.zip › Supplementary 2/Mapper_graphs/353740_graph0B.pdf]

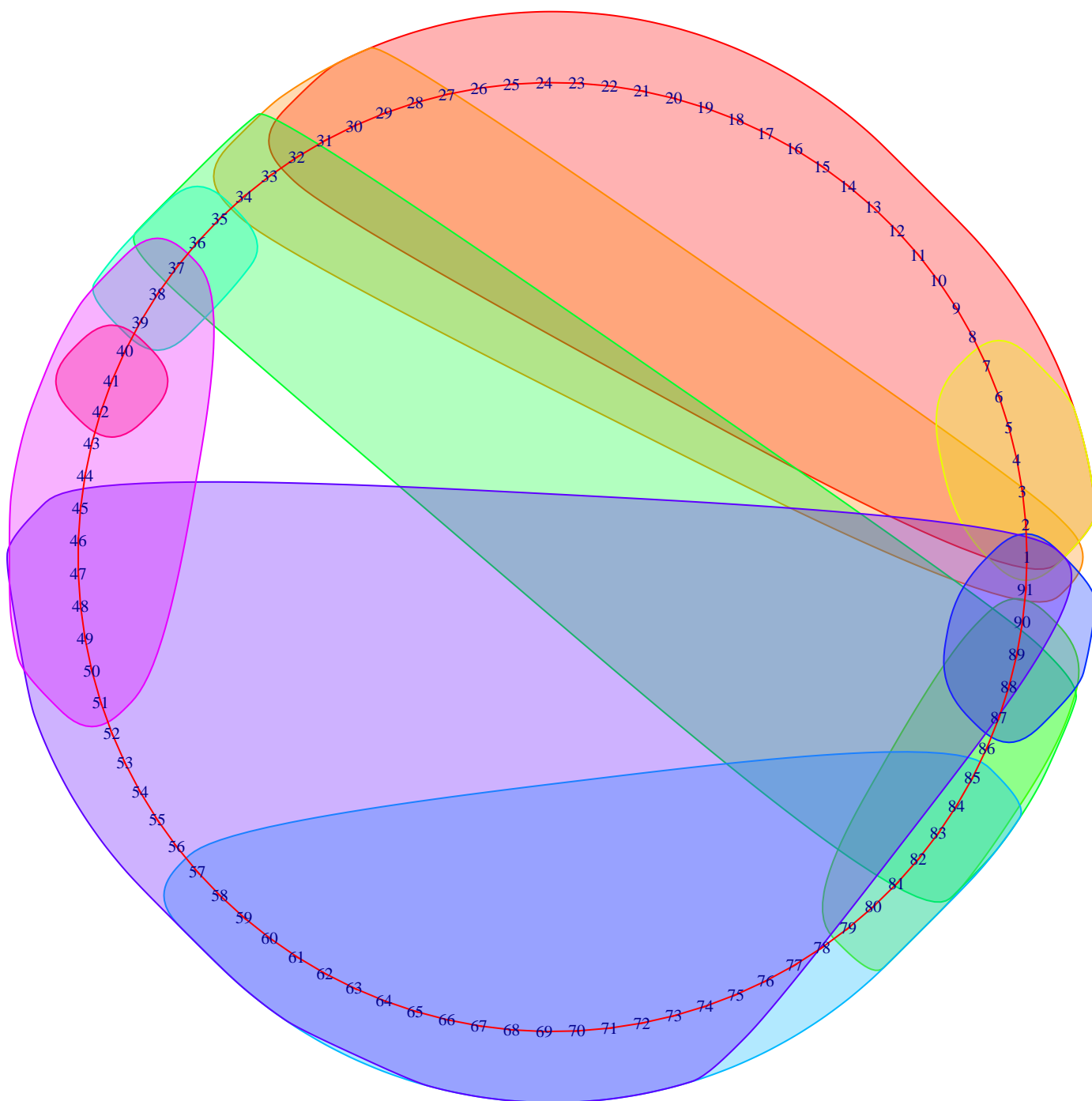

Supplement: Supplementary file 1 [file brainsci-09-00144-s001.zip › Supplementary 2/Mapper_graphs/166438_graph2B.pdf]

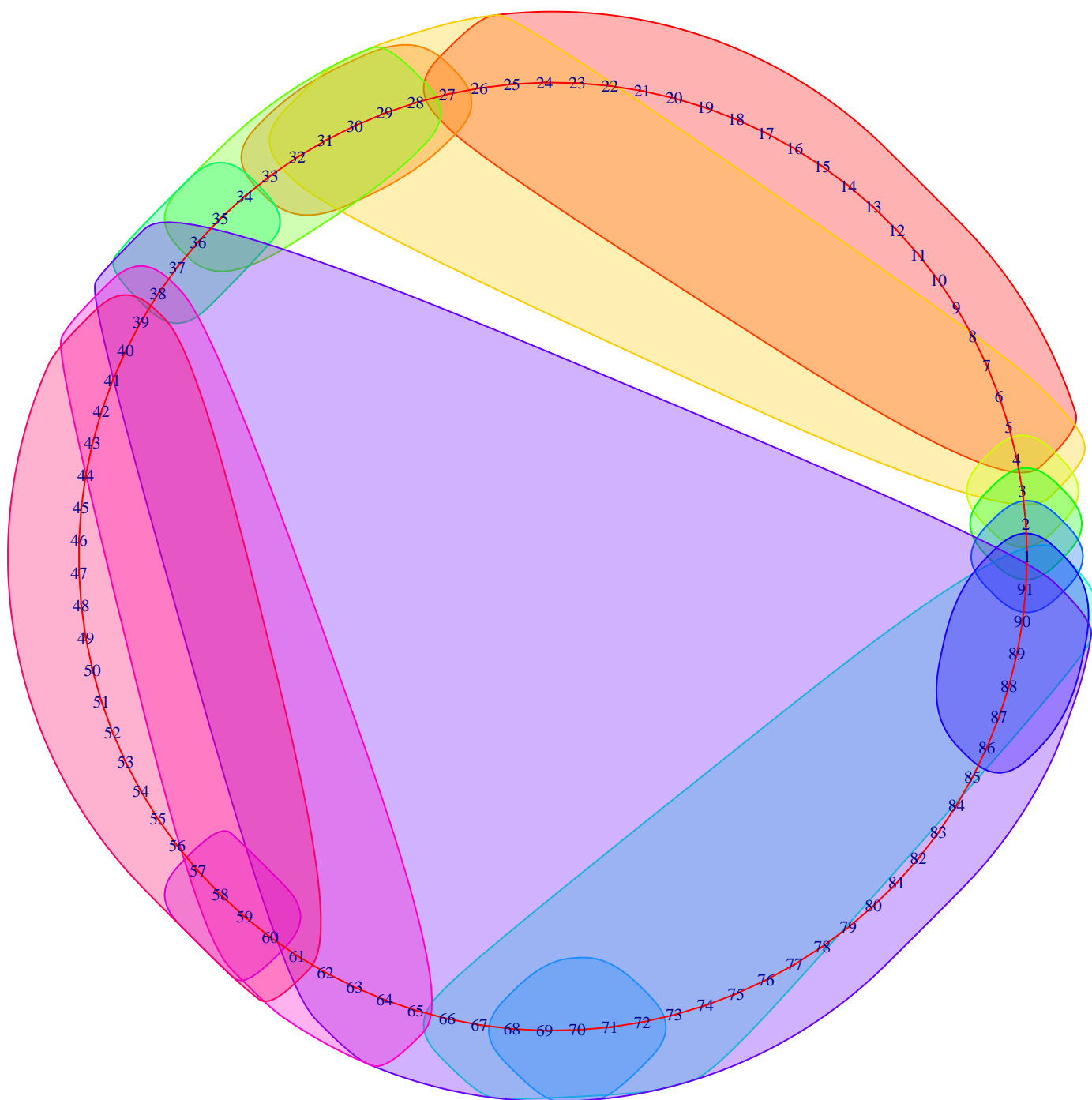

Supplement: Supplementary file 1 [file brainsci-09-00144-s001.zip › Supplementary 2/Mapper_graphs/111514_graph2B.pdf]

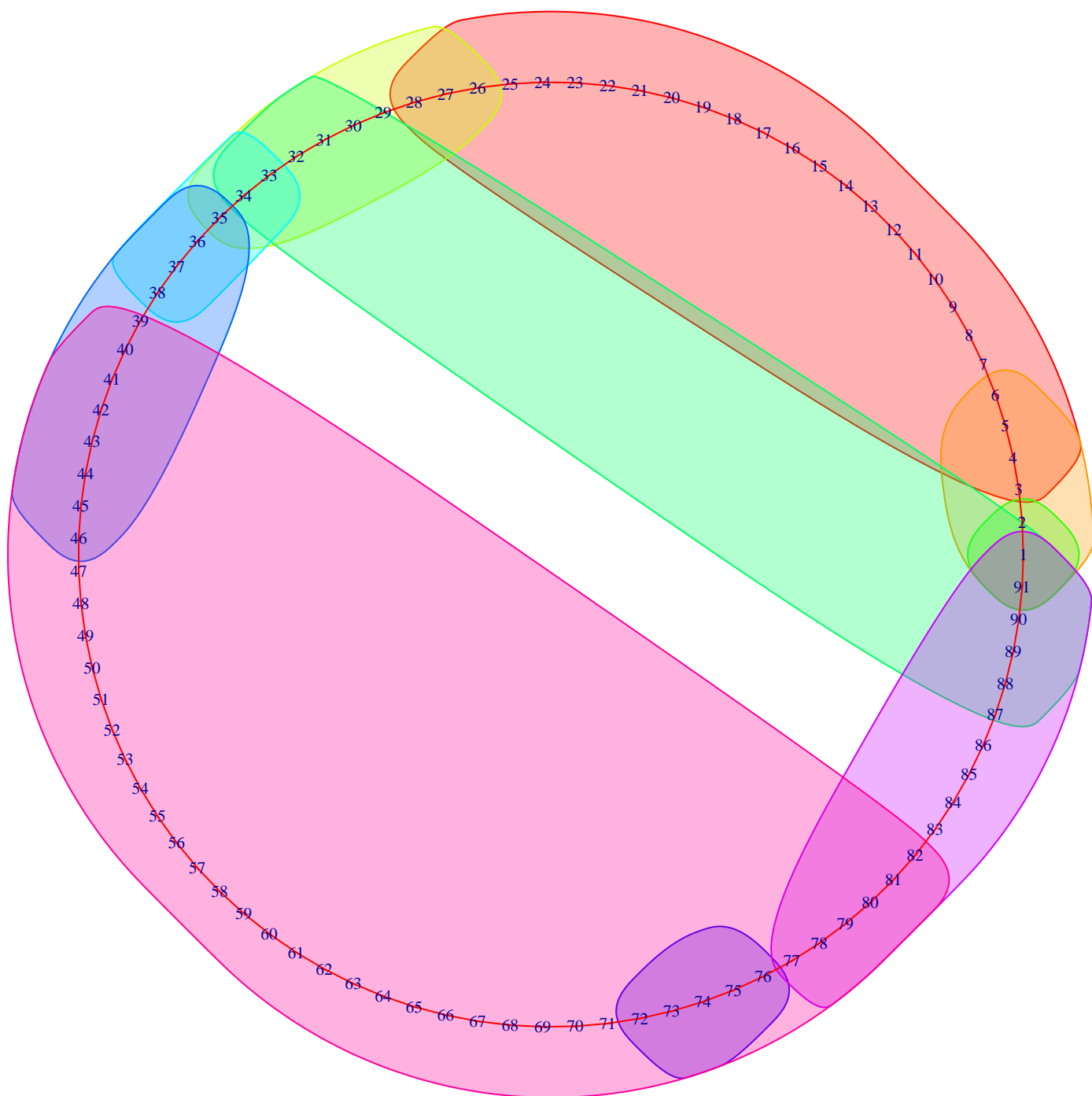

Supplement: Supplementary file 1 [file brainsci-09-00144-s001.zip › Supplementary 2/Mapper_graphs/912447_2B.pdf]

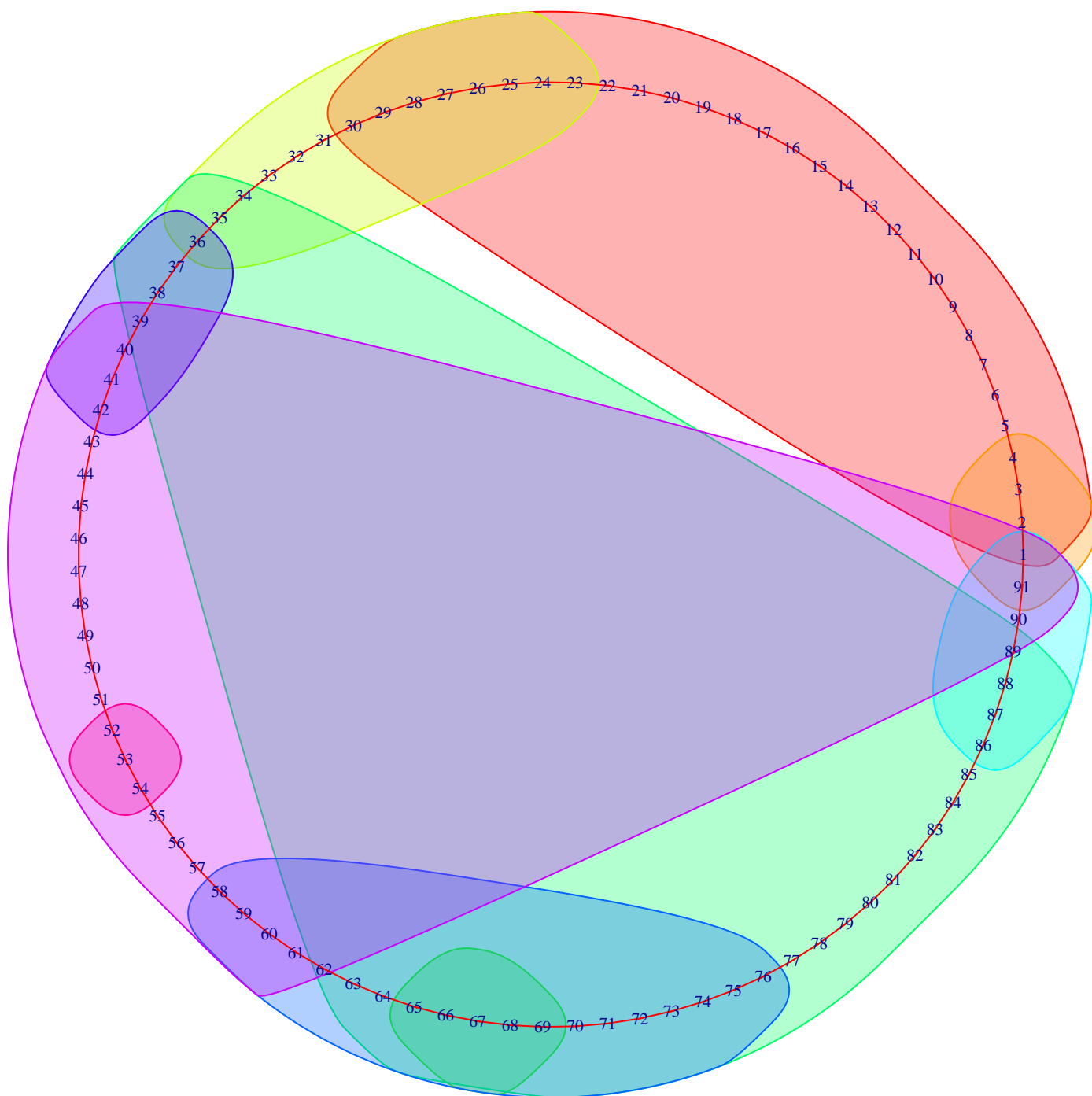

Supplement: Supplementary file 1 [file brainsci-09-00144-s001.zip › Supplementary 2/Mapper_graphs/116726_graph0B.pdf]

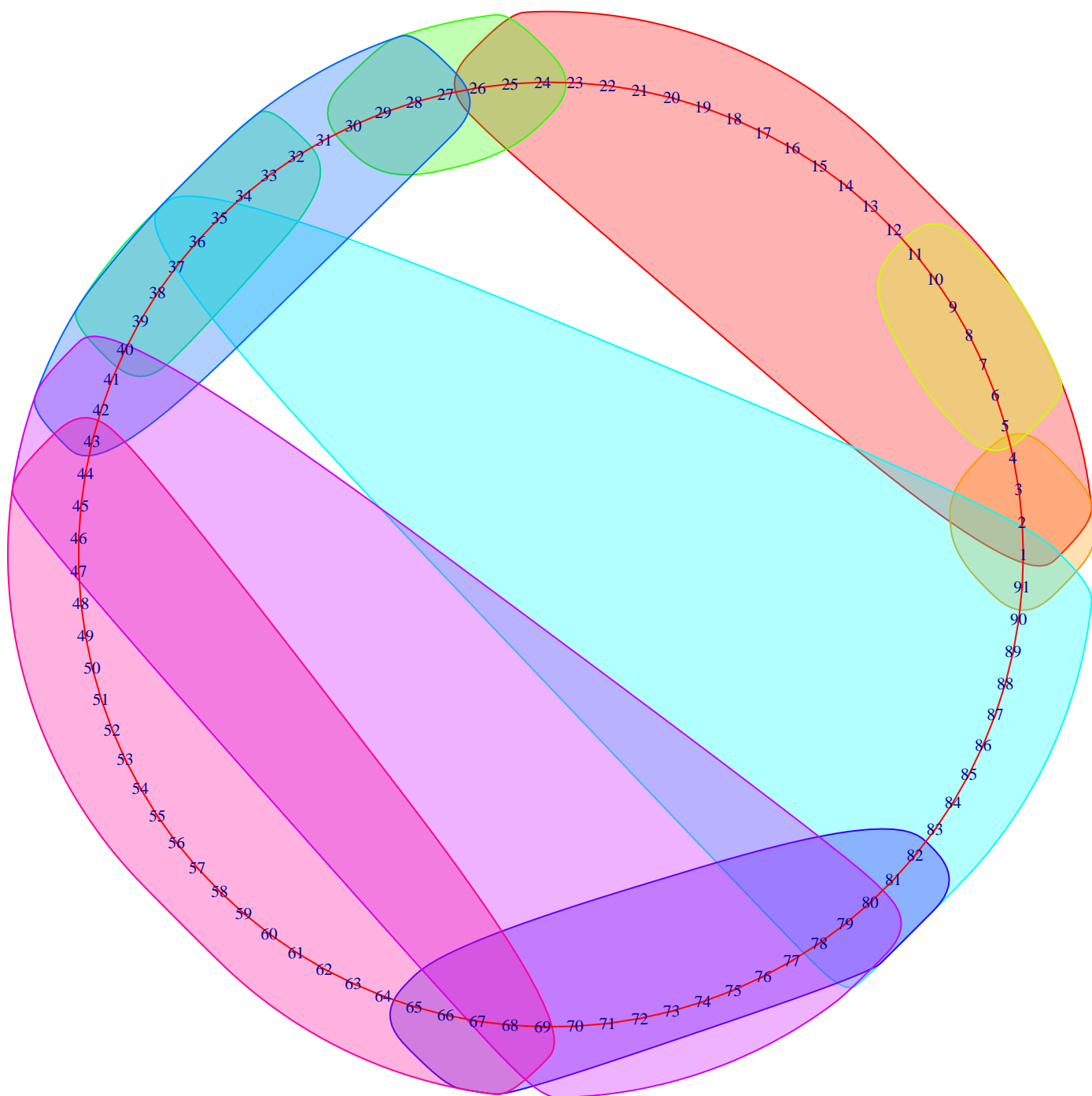

Supplement: Supplementary file 1 [file brainsci-09-00144-s001.zip › Supplementary 2/Mapper_graphs/133019_graph0B.pdf]

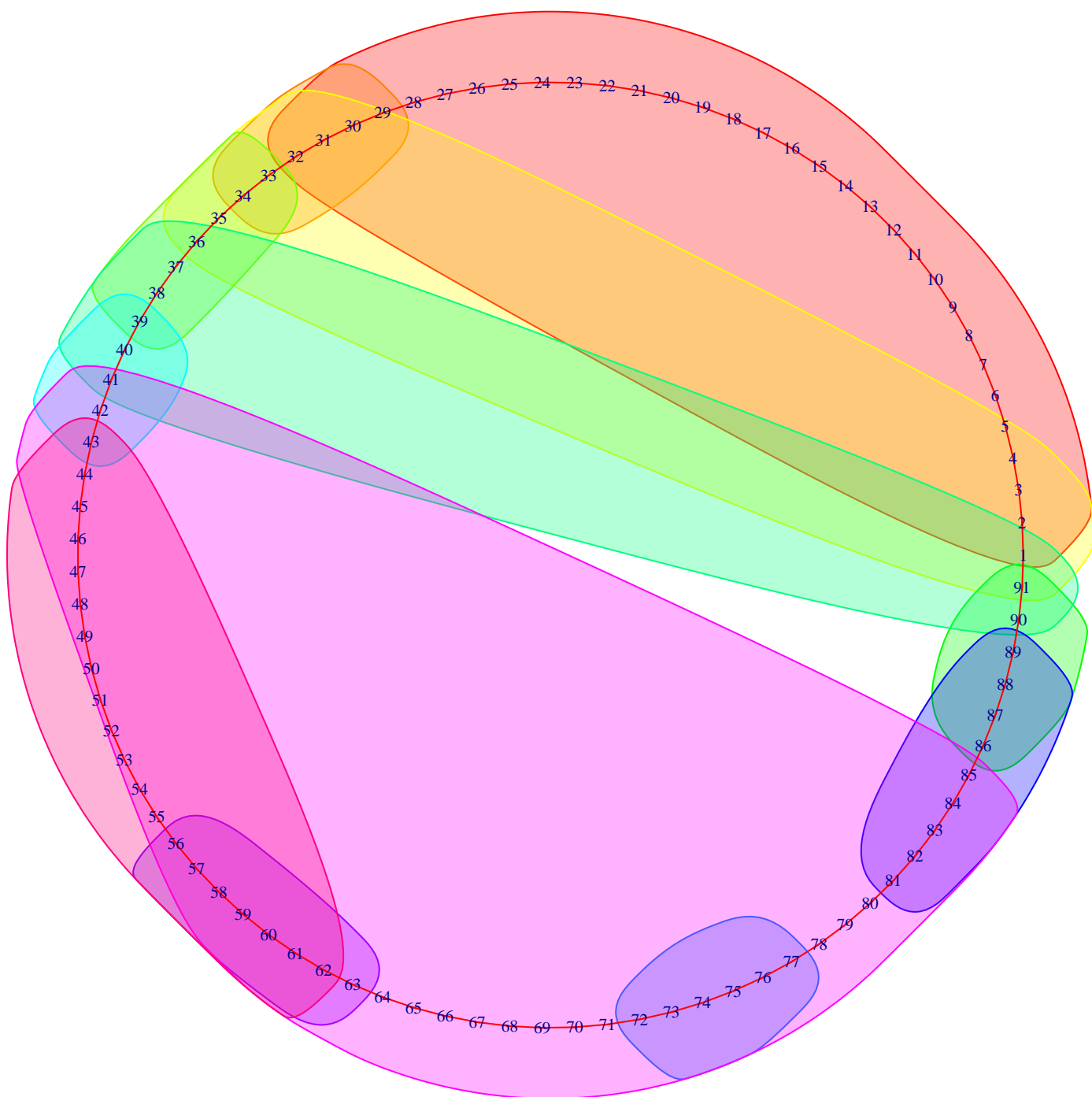

Supplement: Supplementary file 1 [file brainsci-09-00144-s001.zip › Supplementary 2/Mapper_graphs/205119_2B.pdf]
