# Supplementary figures and images for: Uncovering Dynamic Brain Reconfiguration in MEG Working Memory n-Back Task Using Topological Data Analysis (part 1 of 2)
Source: Brain Sci. 2019 Jun 19;9(6):144. doi: 10.3390/brainsci9060144 (PMC6628086; doi:10.3390/brainsci9060144)

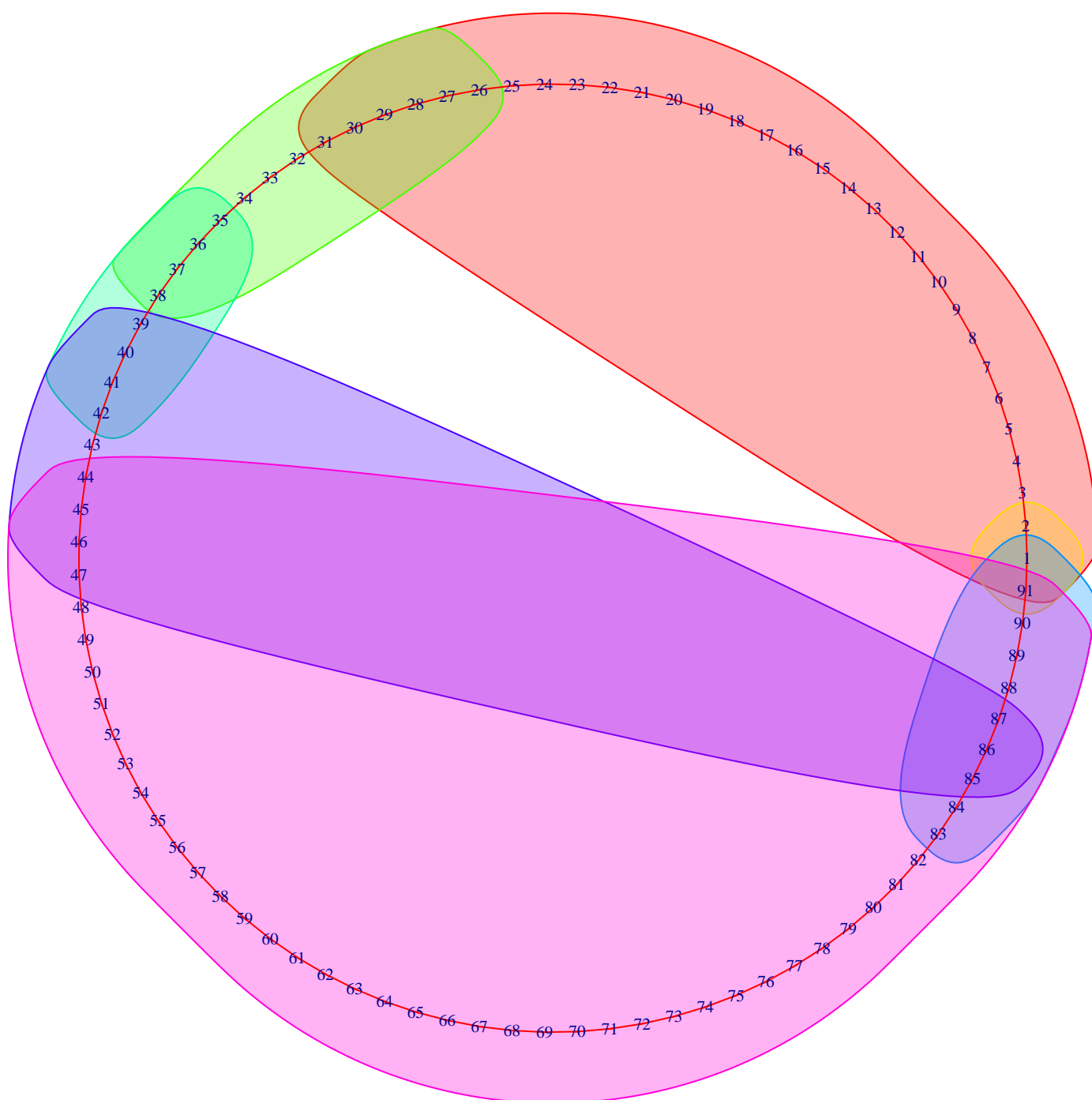

Supplement: Supplementary file 1 [file brainsci-09-00144-s001.zip › Supplementary 2/Mapper_graphs/512835_2B.pdf]

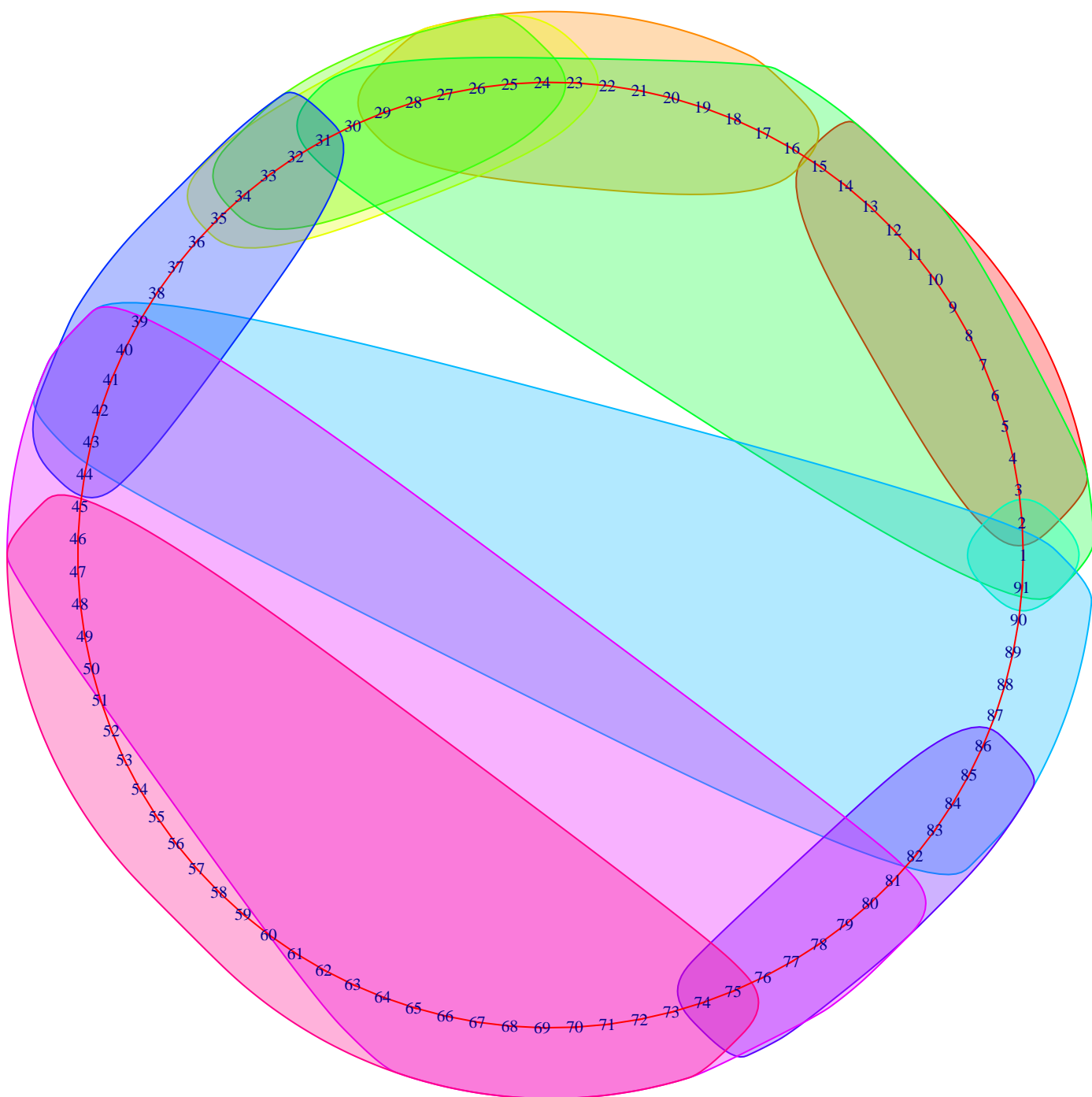

Supplement: Supplementary file 1 [file brainsci-09-00144-s001.zip › Supplementary 2/Mapper_graphs/149741_graph2B.pdf]

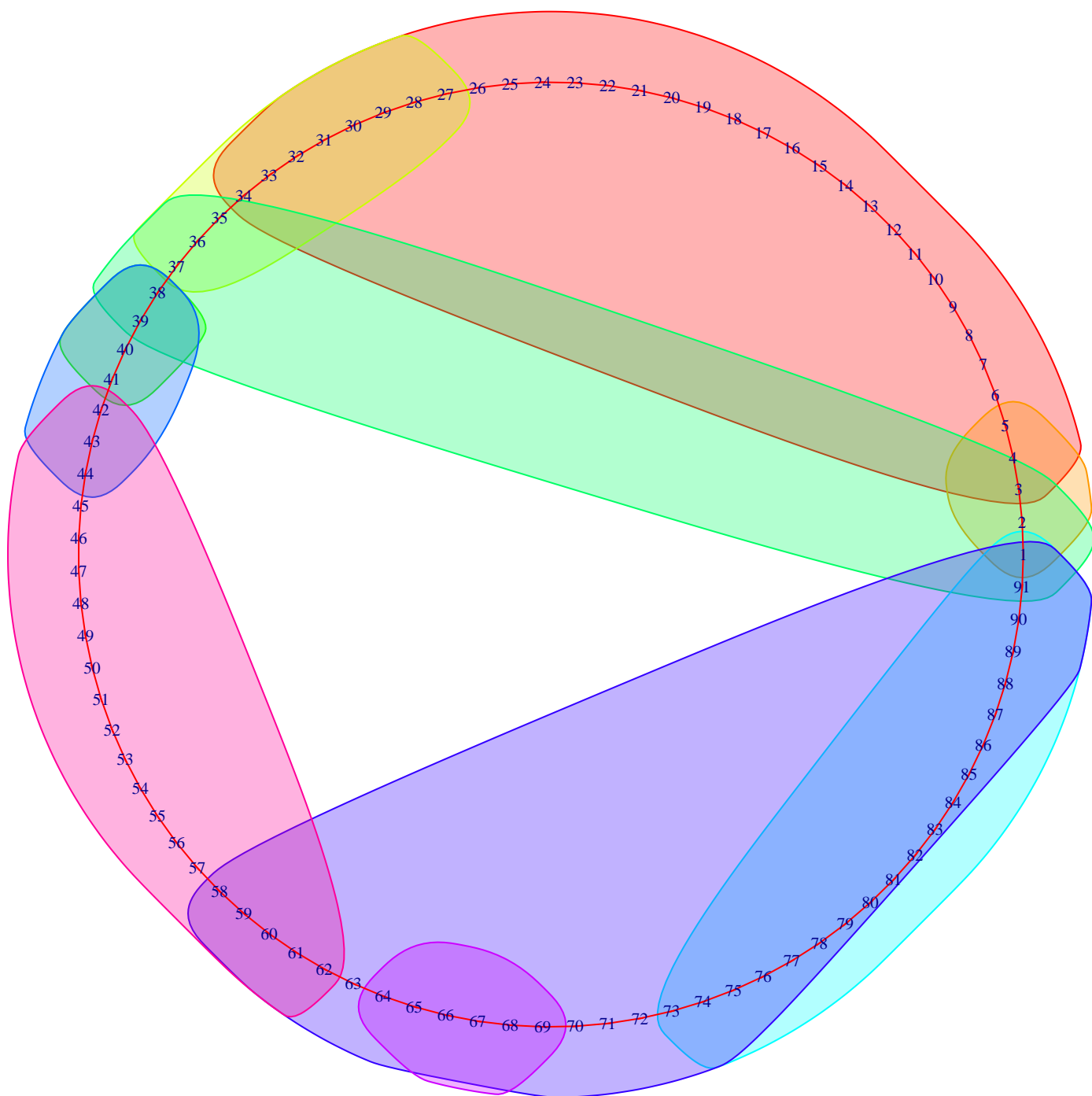

Supplement: Supplementary file 1 [file brainsci-09-00144-s001.zip › Supplementary 2/Mapper_graphs/990366_0B.pdf]

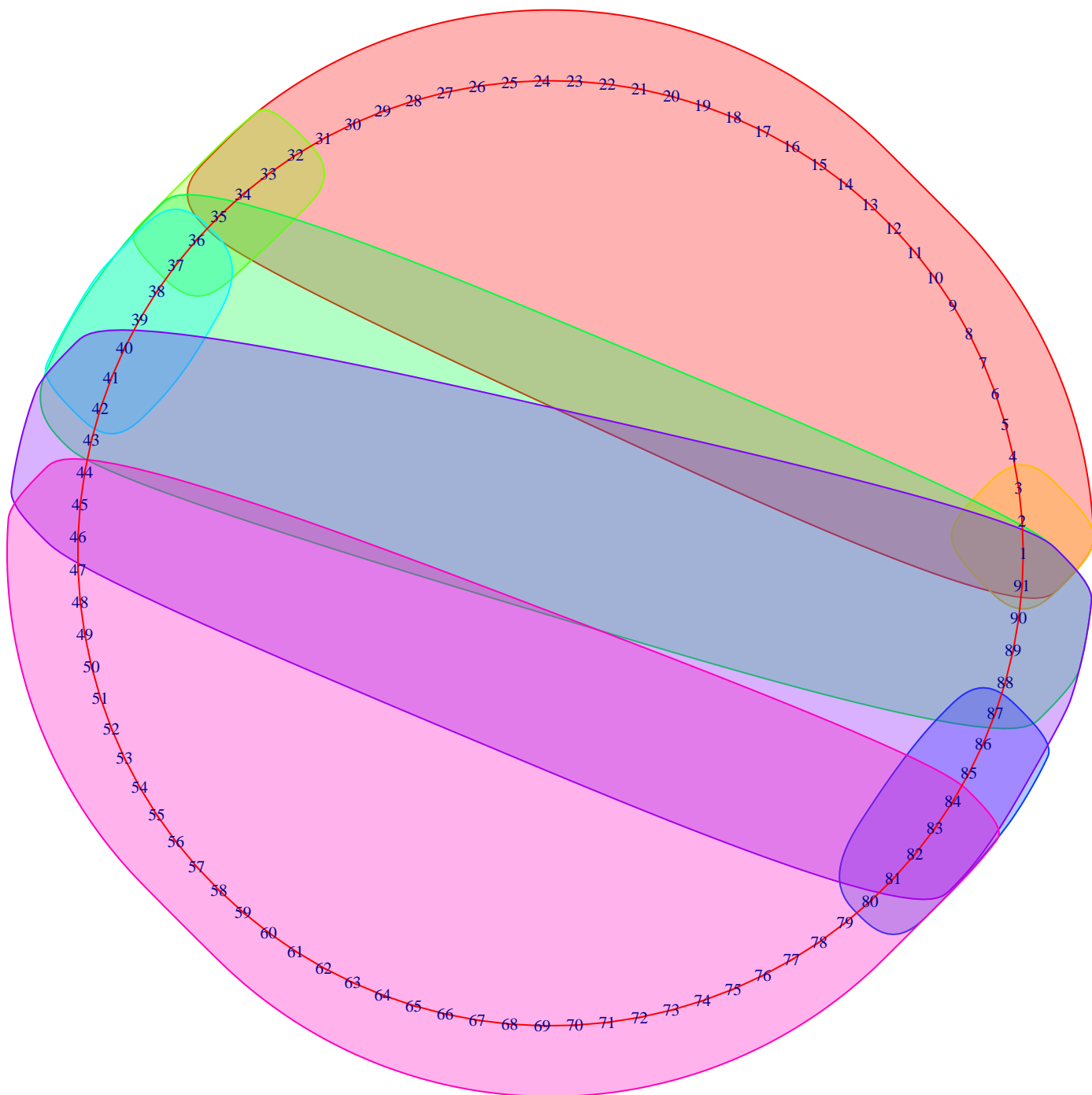

Supplement: Supplementary file 1 [file brainsci-09-00144-s001.zip › Supplementary 2/Mapper_graphs/898176_2B.pdf]

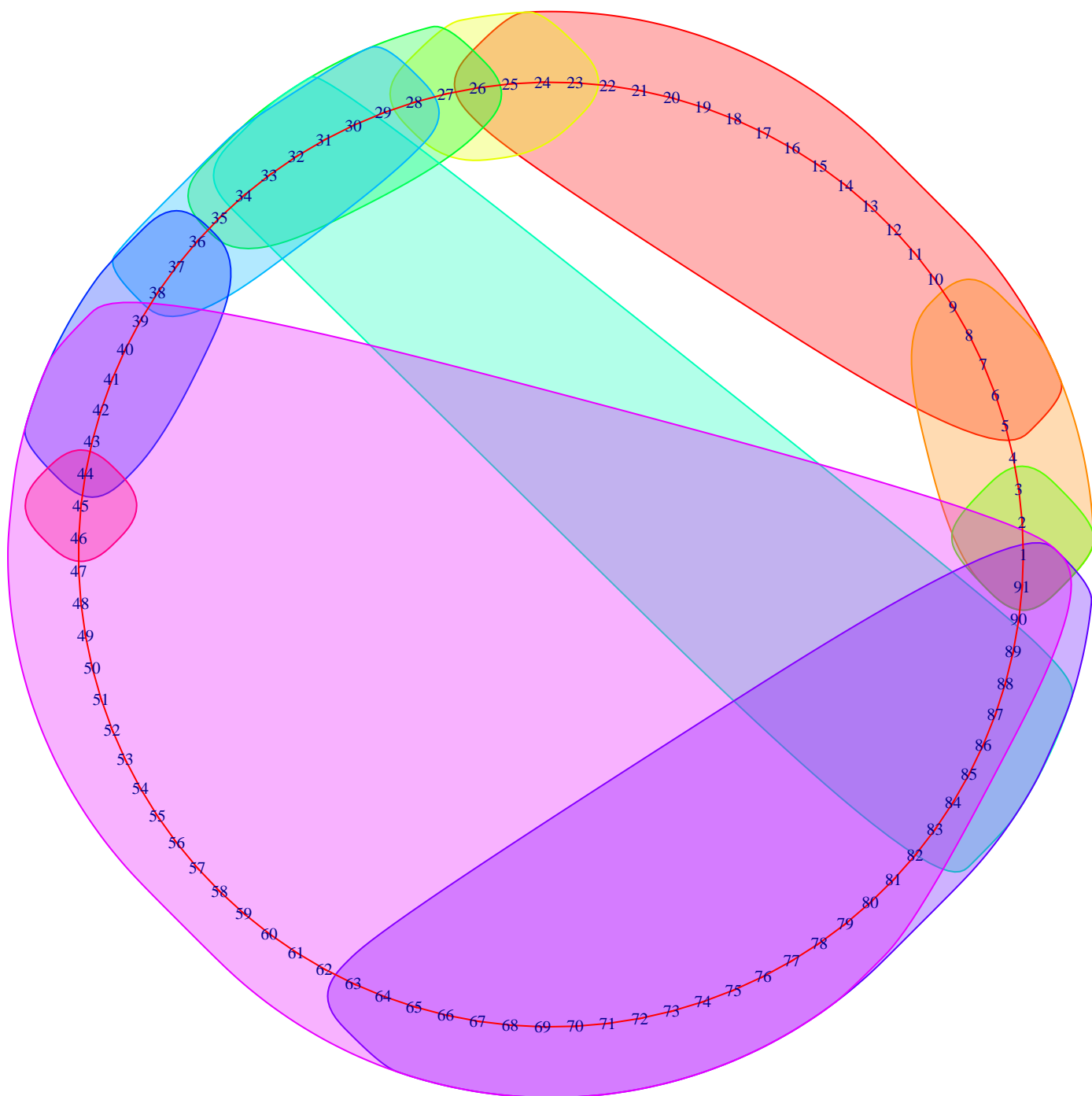

Supplement: Supplementary file 1 [file brainsci-09-00144-s001.zip › Supplementary 2/Mapper_graphs/255639_0B.pdf]

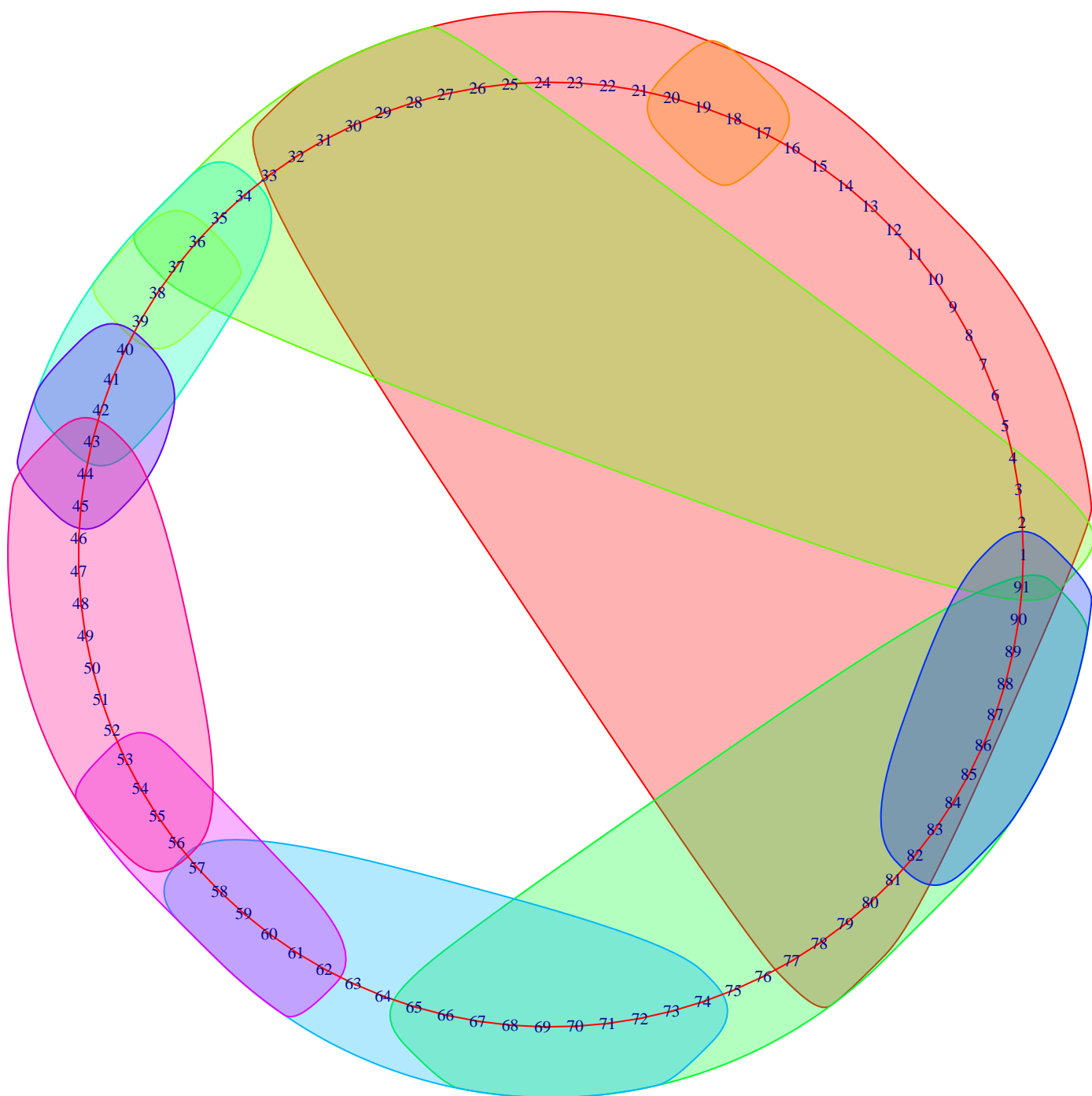

Supplement: Supplementary file 1 [file brainsci-09-00144-s001.zip › Supplementary 2/Mapper_graphs/214524_2B.pdf]

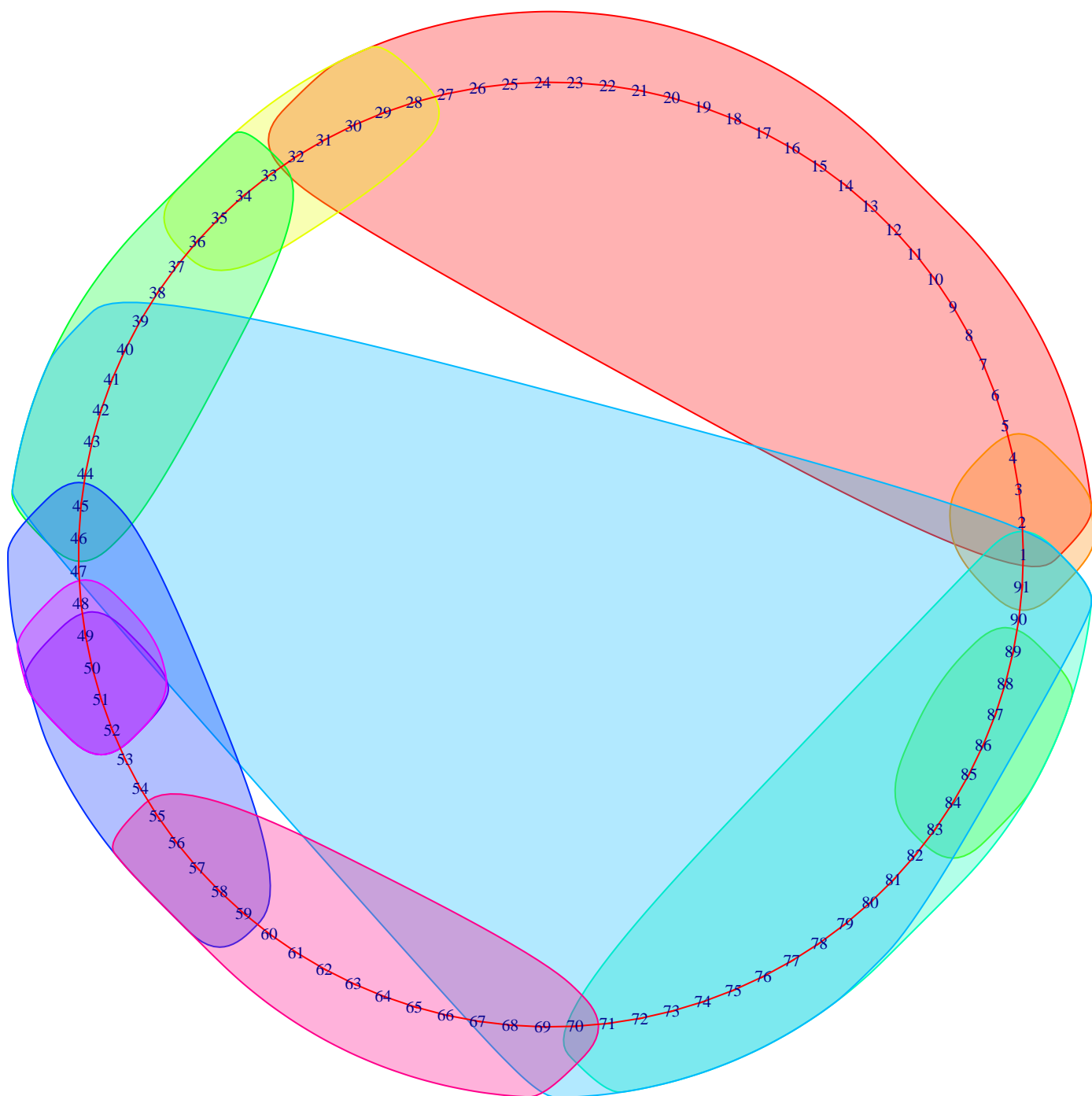

Supplement: Supplementary file 1 [file brainsci-09-00144-s001.zip › Supplementary 2/Mapper_graphs/433839_graph2B.pdf]

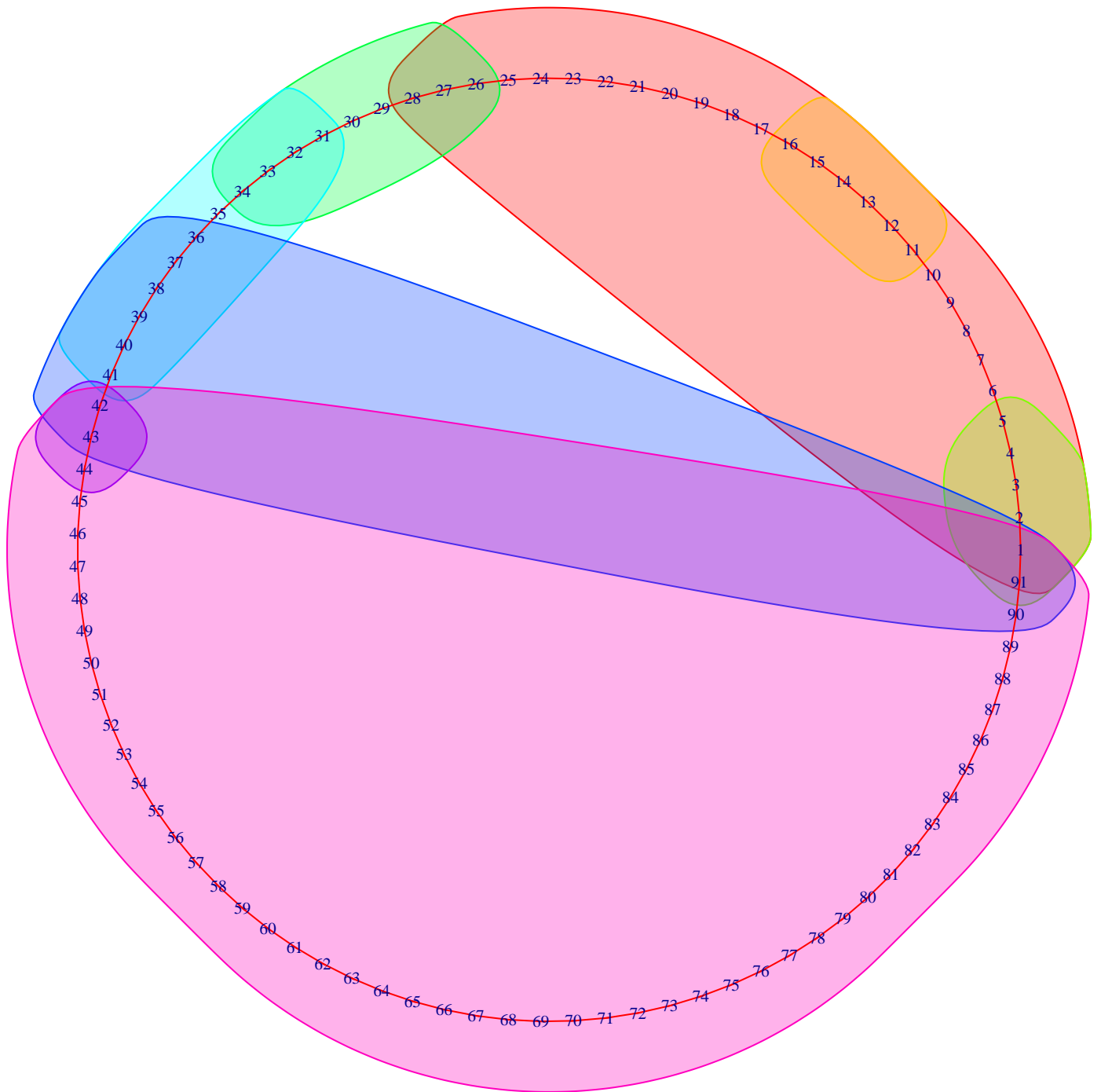

Supplement: Supplementary file 1 [file brainsci-09-00144-s001.zip › Supplementary 2/Mapper_graphs/706040_2B.pdf]

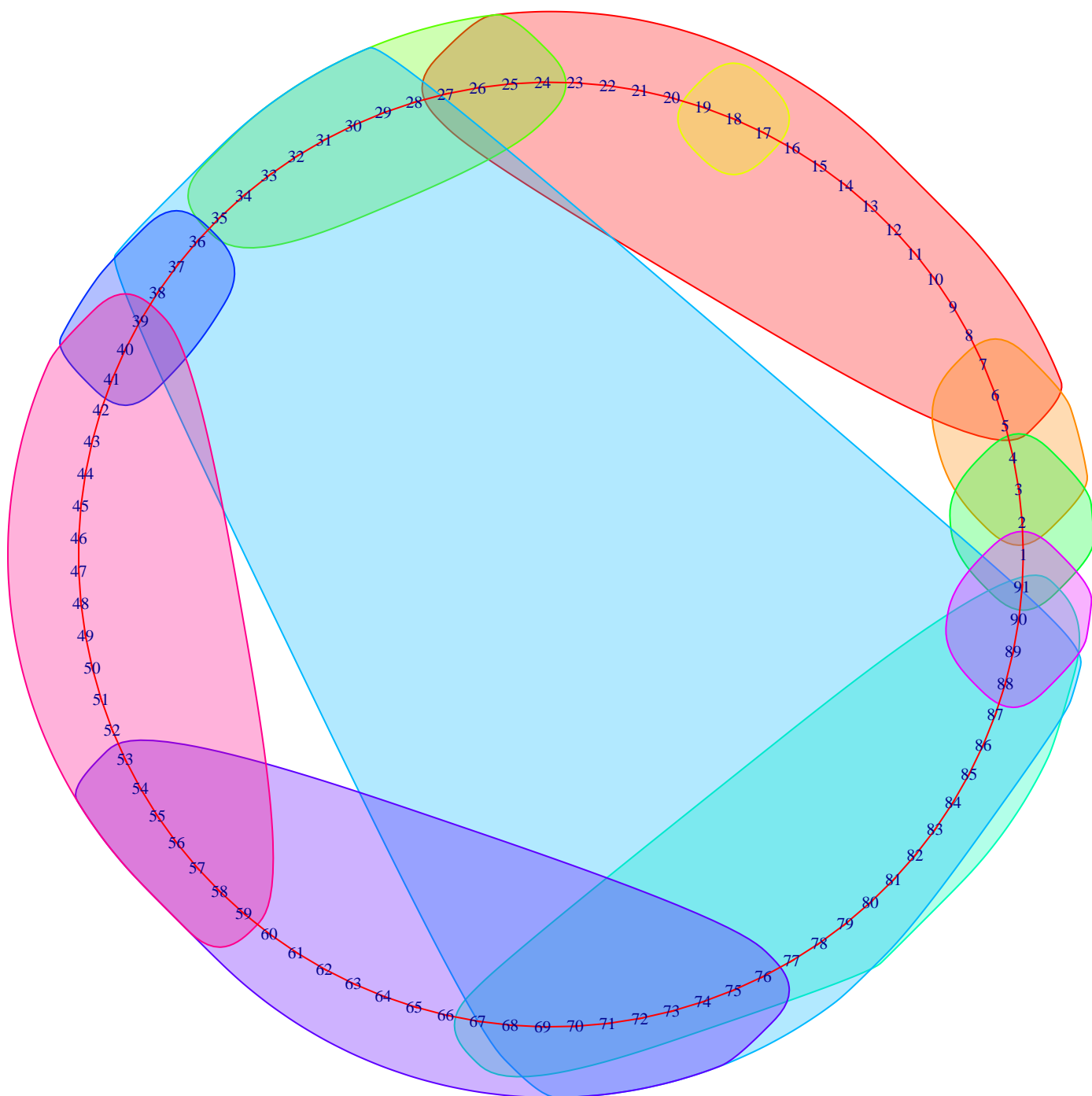

Supplement: Supplementary file 1 [file brainsci-09-00144-s001.zip › Supplementary 2/Mapper_graphs/707749_0B.pdf]

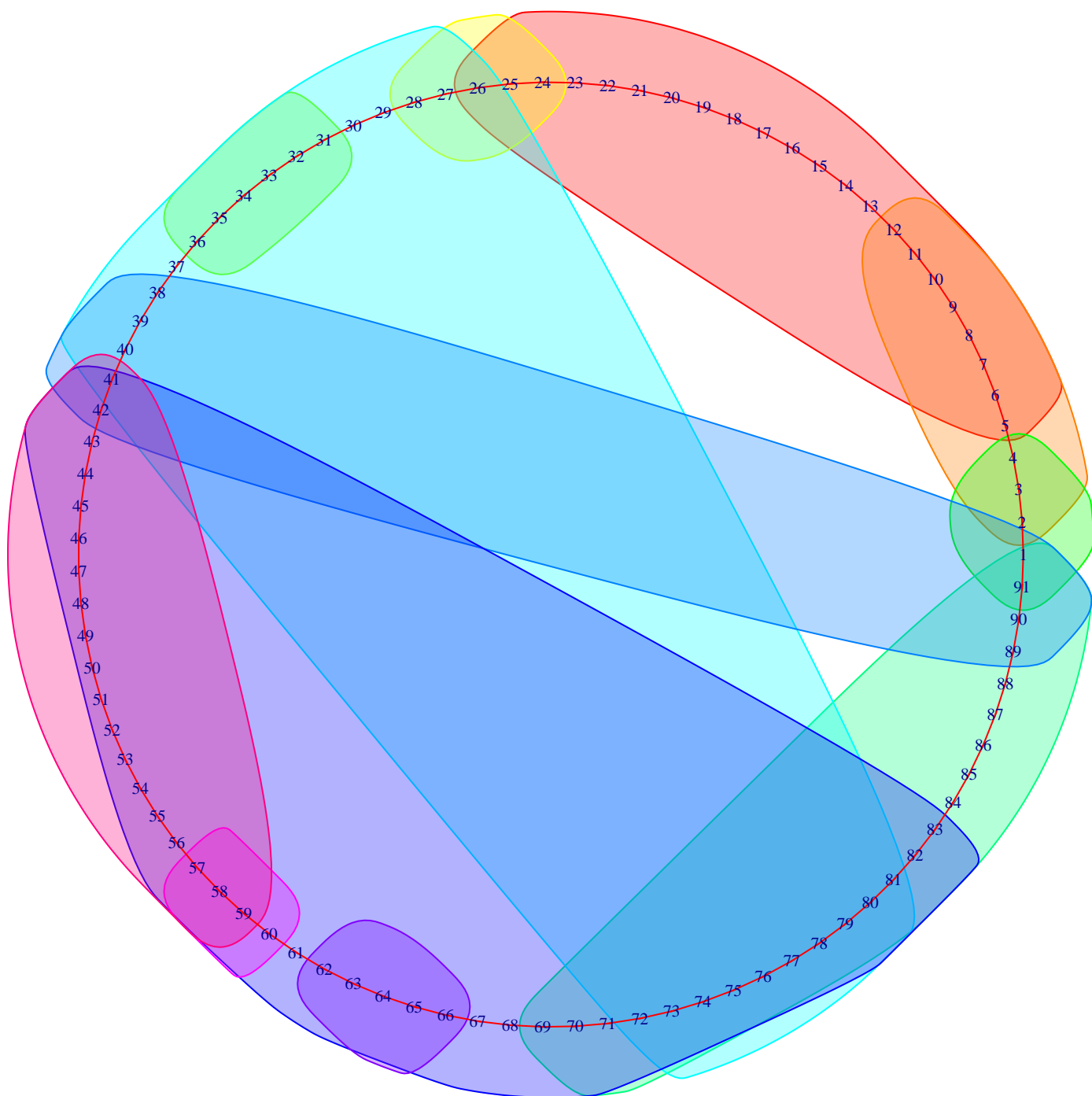

Supplement: Supplementary file 1 [file brainsci-09-00144-s001.zip › Supplementary 2/Mapper_graphs/100307_0B.pdf]

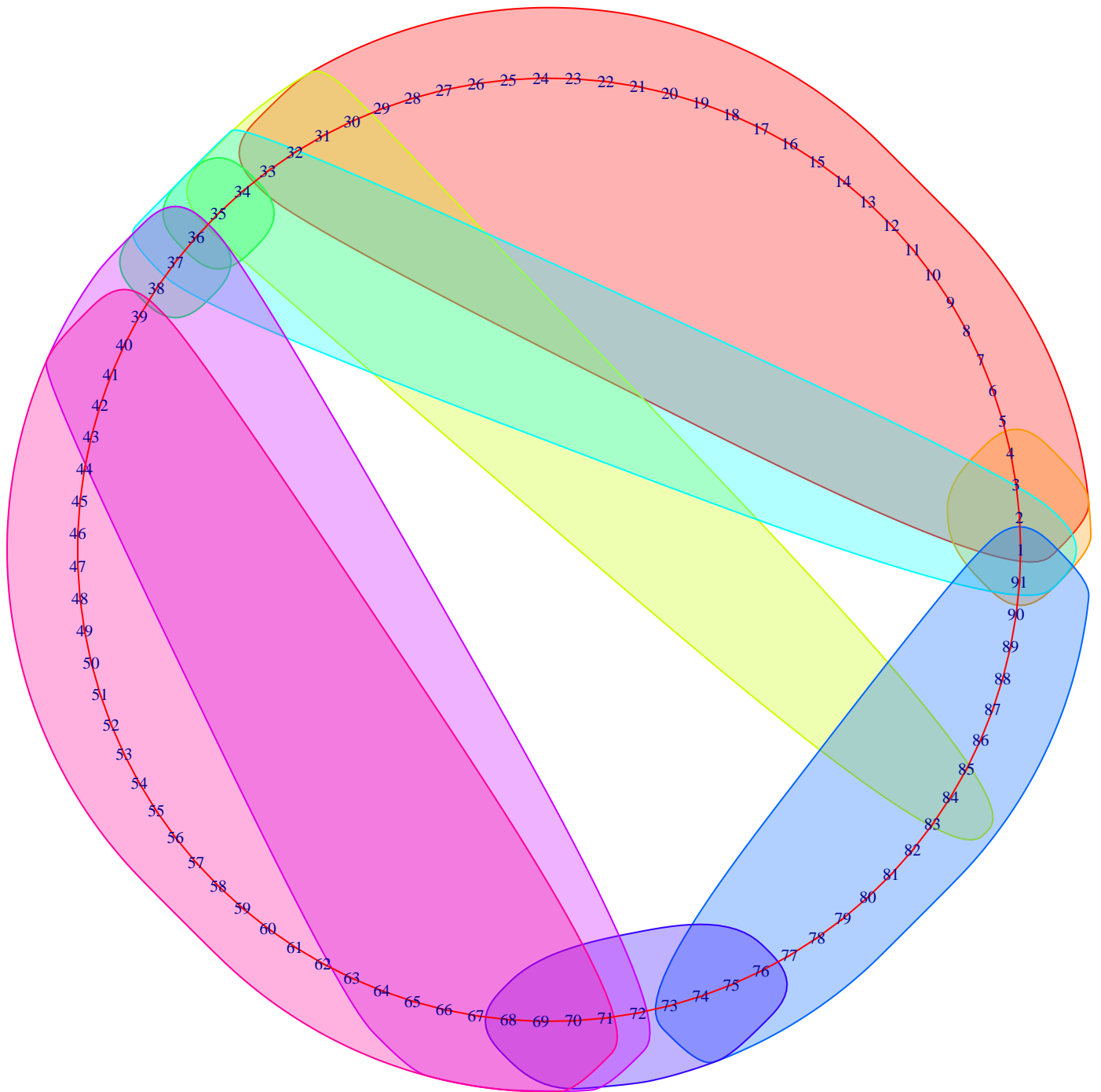

Supplement: Supplementary file 1 [file brainsci-09-00144-s001.zip › Supplementary 2/Mapper_graphs/257845_2B.pdf]

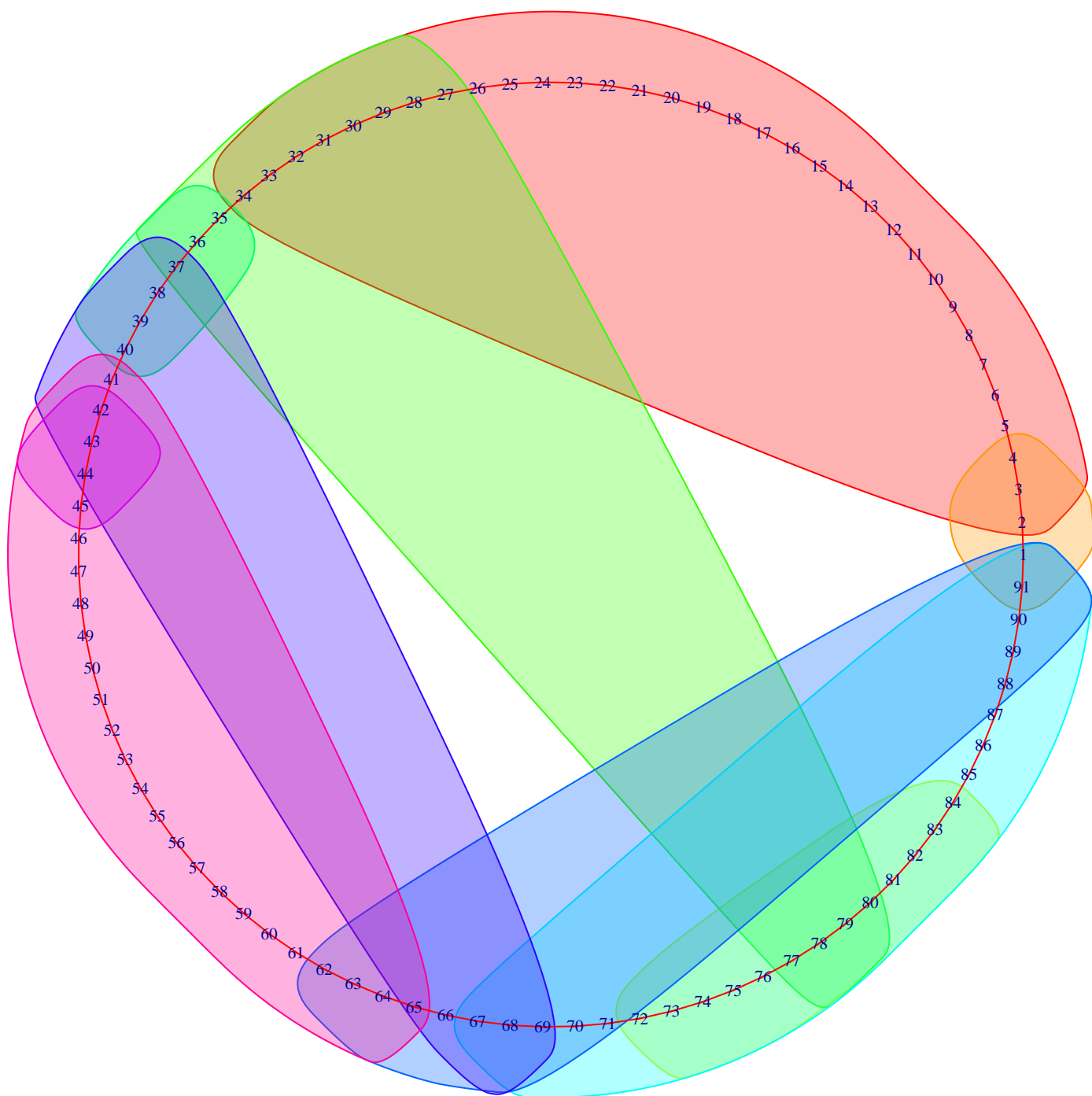

Supplement: Supplementary file 1 [file brainsci-09-00144-s001.zip › Supplementary 2/Mapper_graphs/212318_2B.pdf]

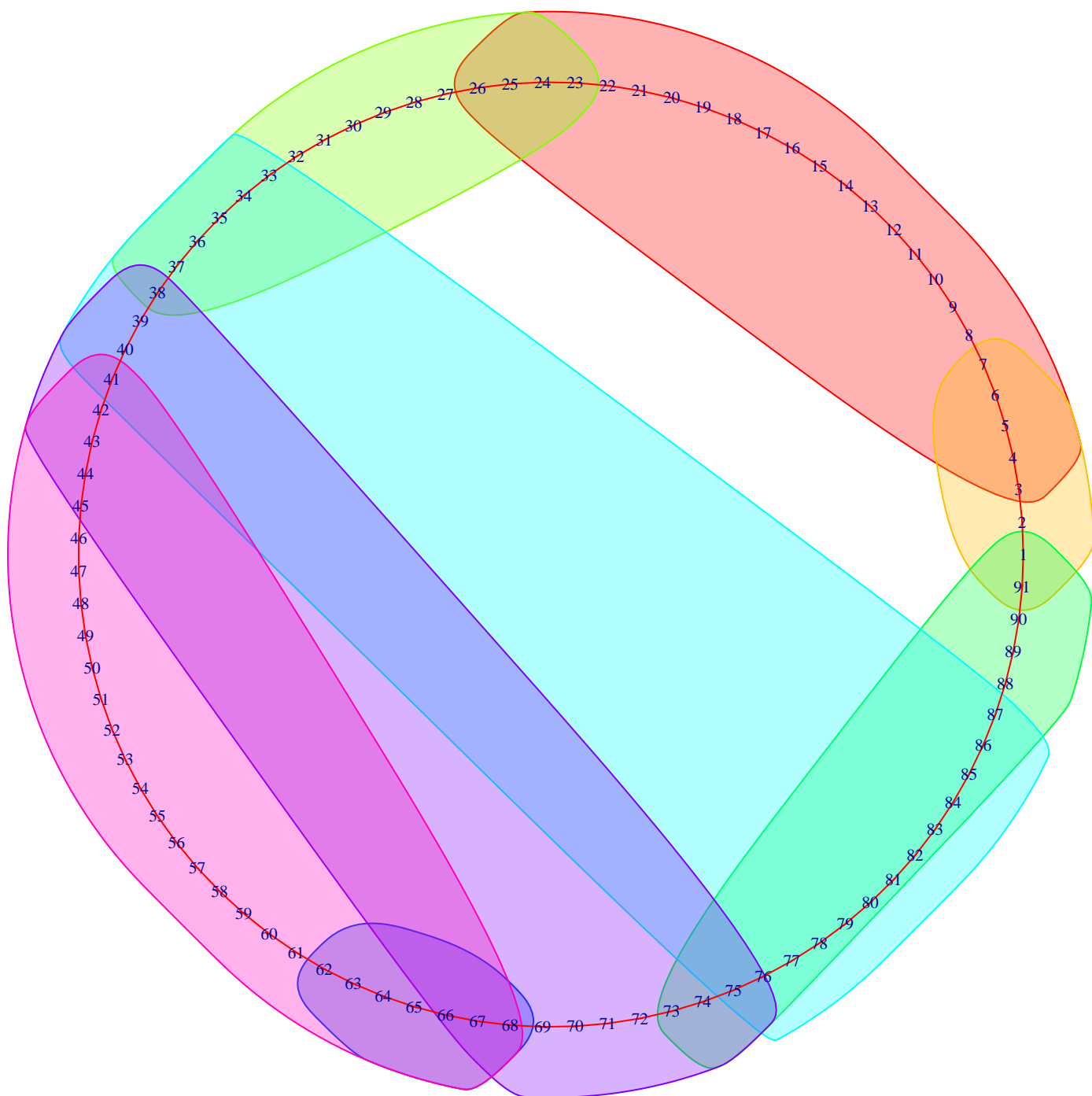

Supplement: Supplementary file 1 [file brainsci-09-00144-s001.zip › Supplementary 2/Mapper_graphs/191437_2B.pdf]

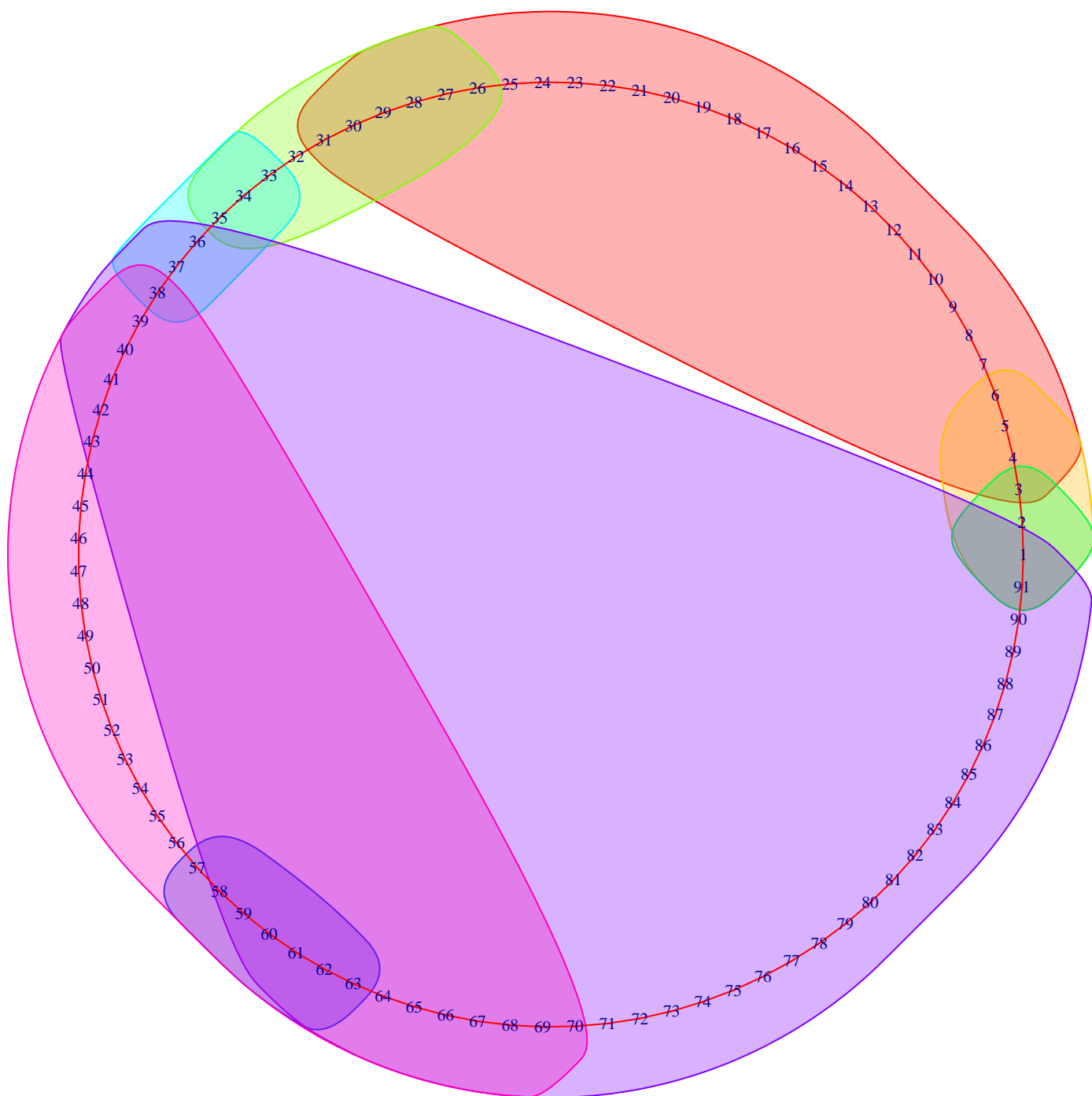

Supplement: Supplementary file 1 [file brainsci-09-00144-s001.zip › Supplementary 2/Mapper_graphs/680957_0B.pdf]

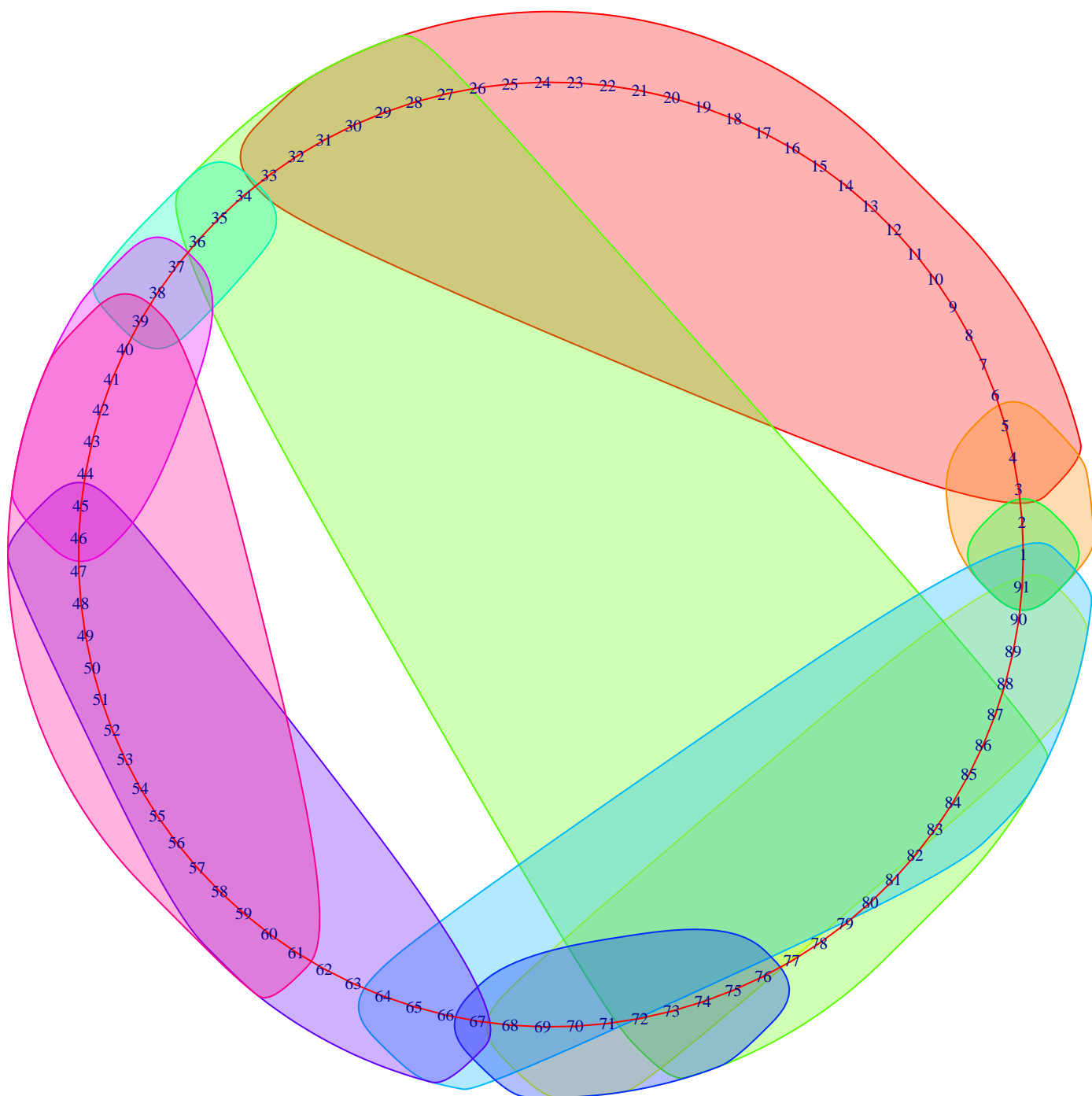

Supplement: Supplementary file 1 [file brainsci-09-00144-s001.zip › Supplementary 2/Mapper_graphs/814649_0B.pdf]

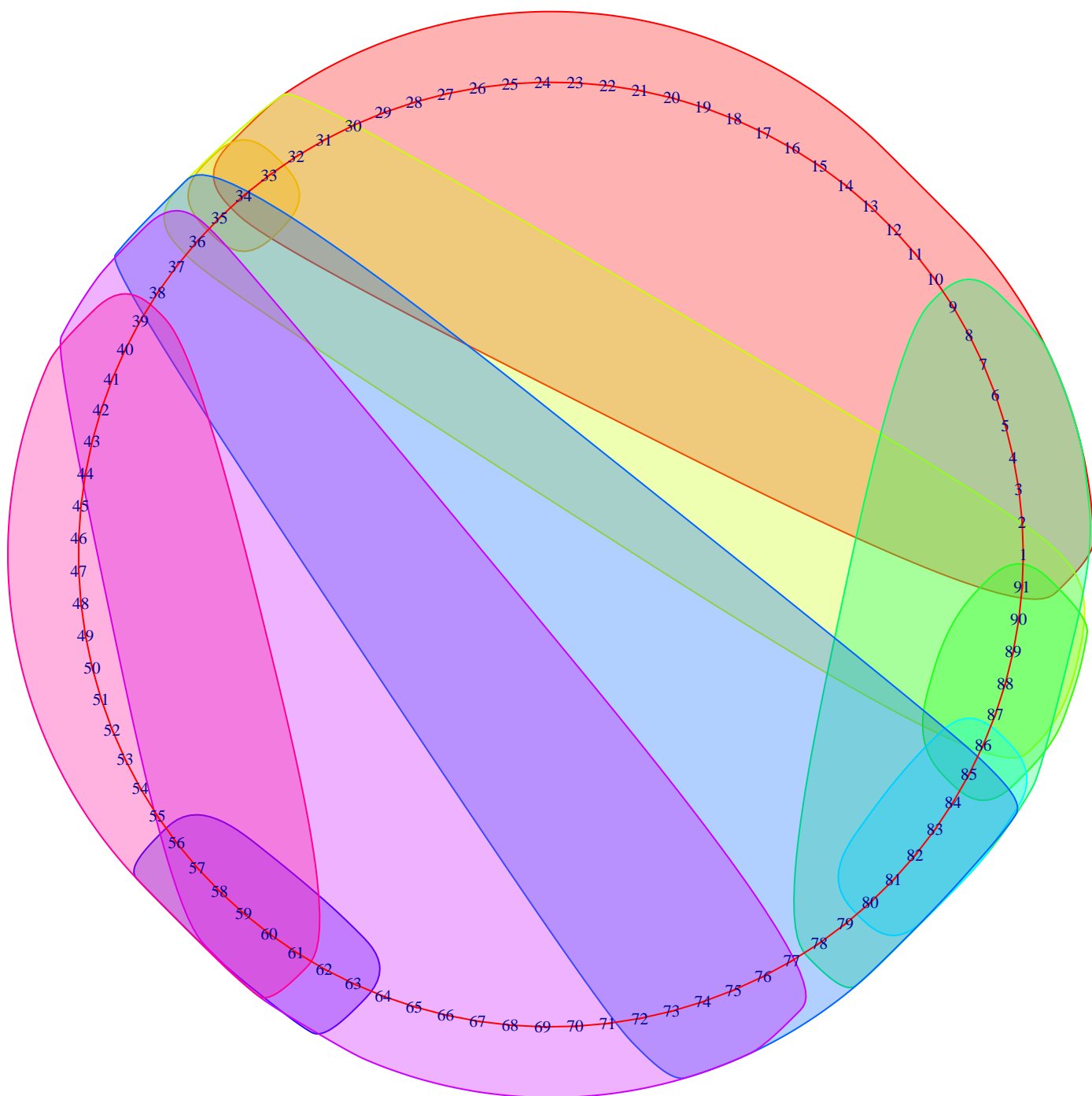

Supplement: Supplementary file 1 [file brainsci-09-00144-s001.zip › Supplementary 2/Mapper_graphs/500222_graph2B.pdf]

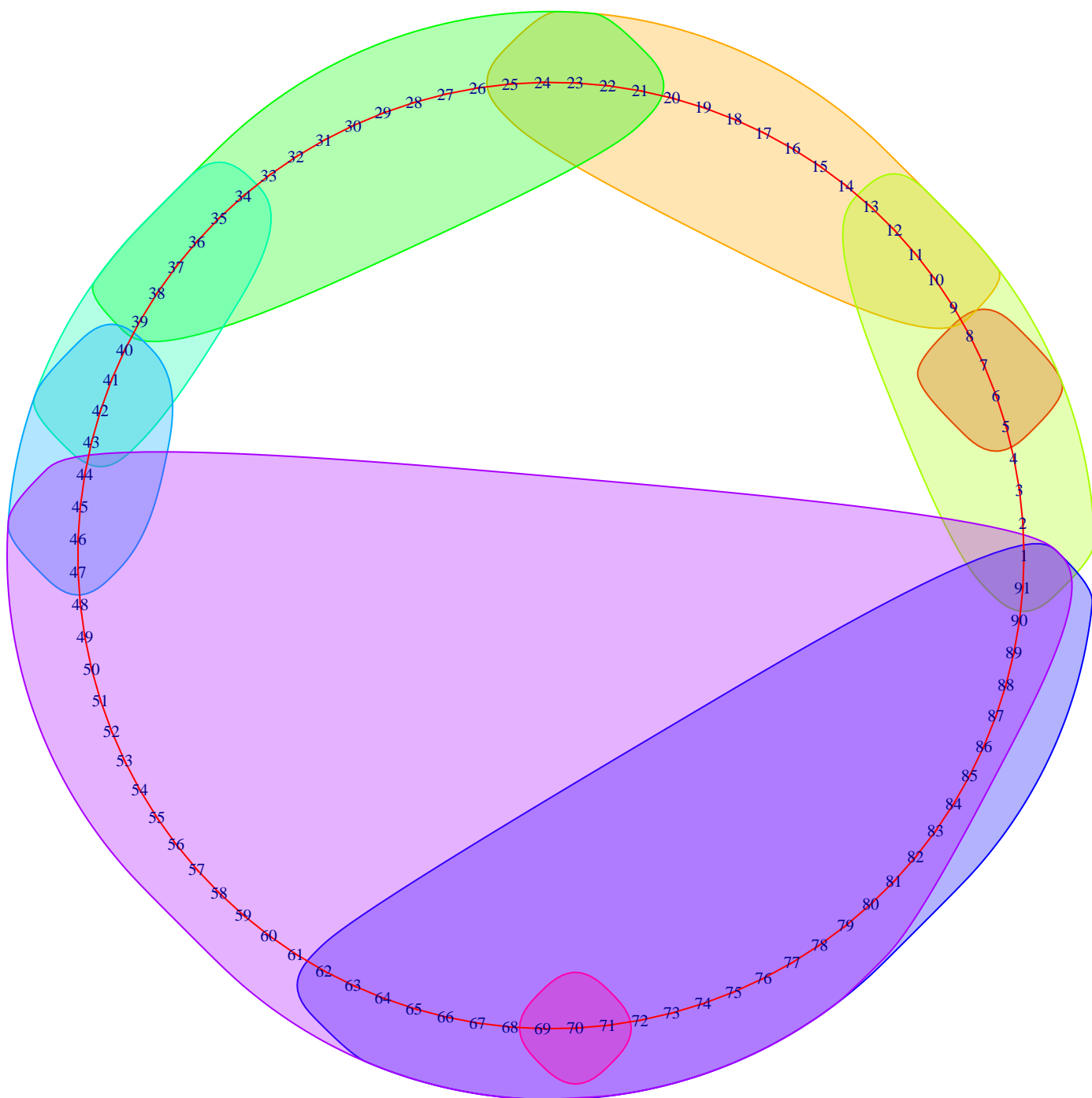

Supplement: Supplementary file 1 [file brainsci-09-00144-s001.zip › Supplementary 2/Mapper_graphs/198653_0B.pdf]

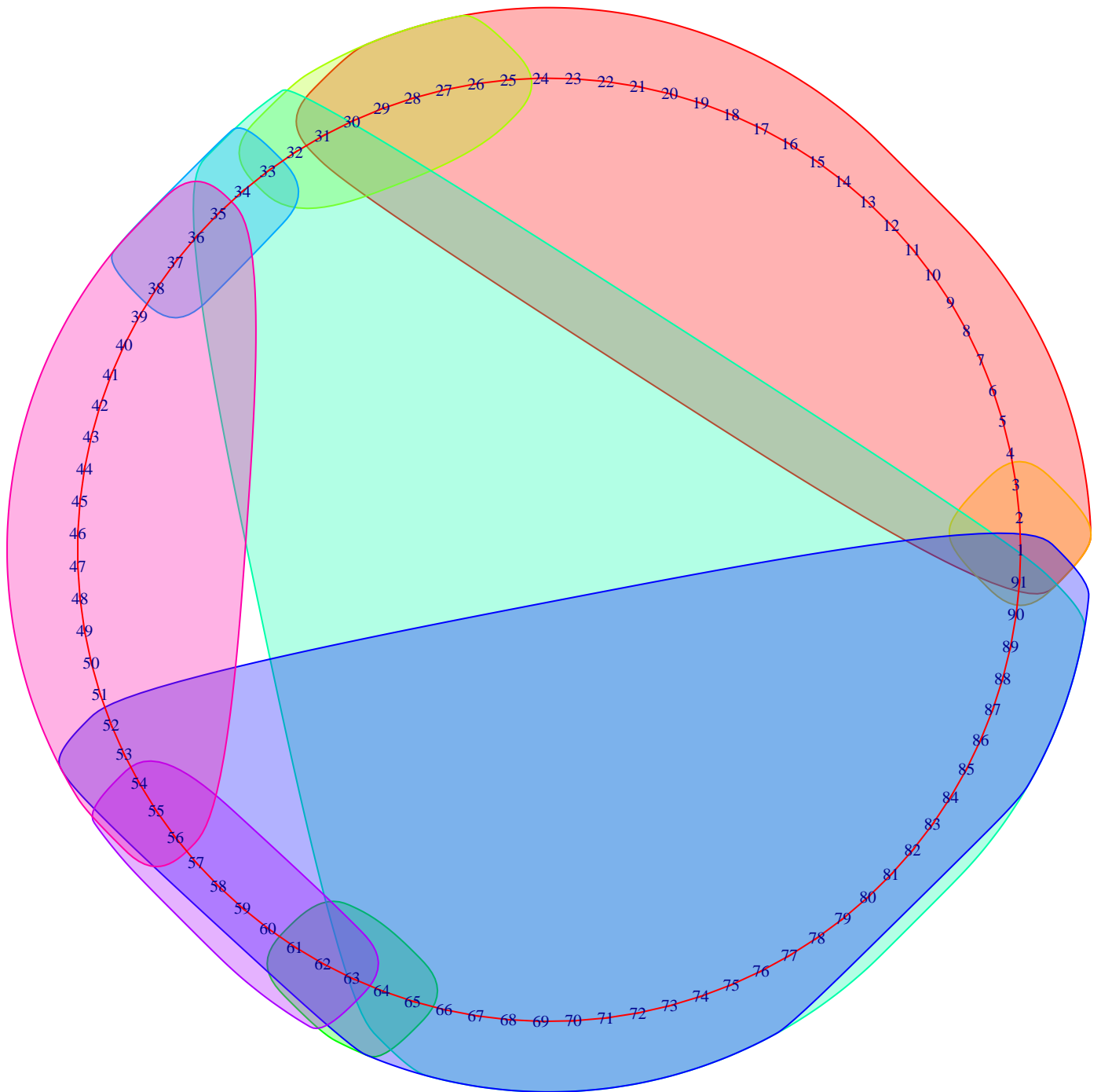

Supplement: Supplementary file 1 [file brainsci-09-00144-s001.zip › Supplementary 2/Mapper_graphs/872764_0B.pdf]

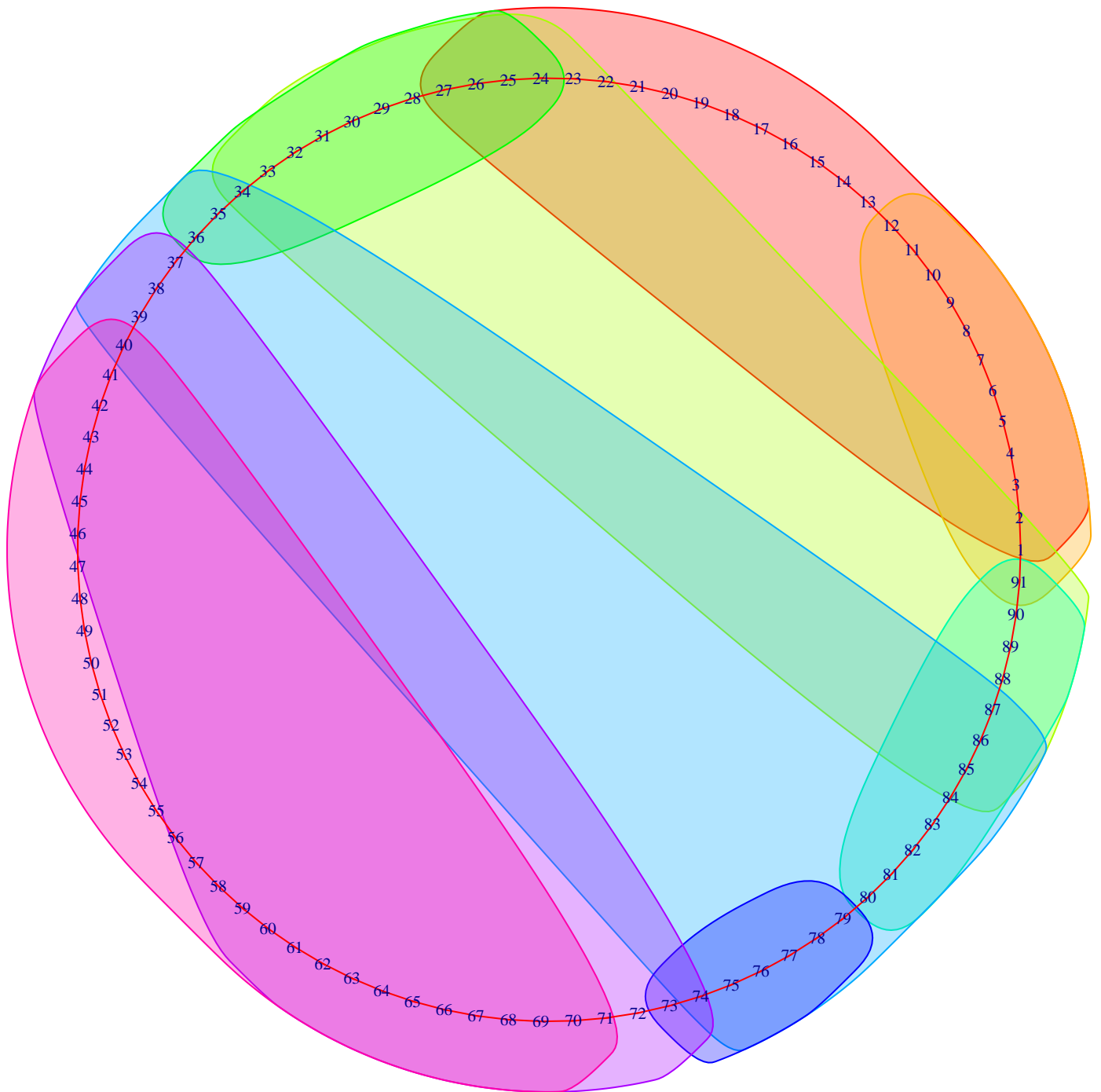

Supplement: Supplementary file 1 [file brainsci-09-00144-s001.zip › Supplementary 2/Mapper_graphs/205119_0B.pdf]

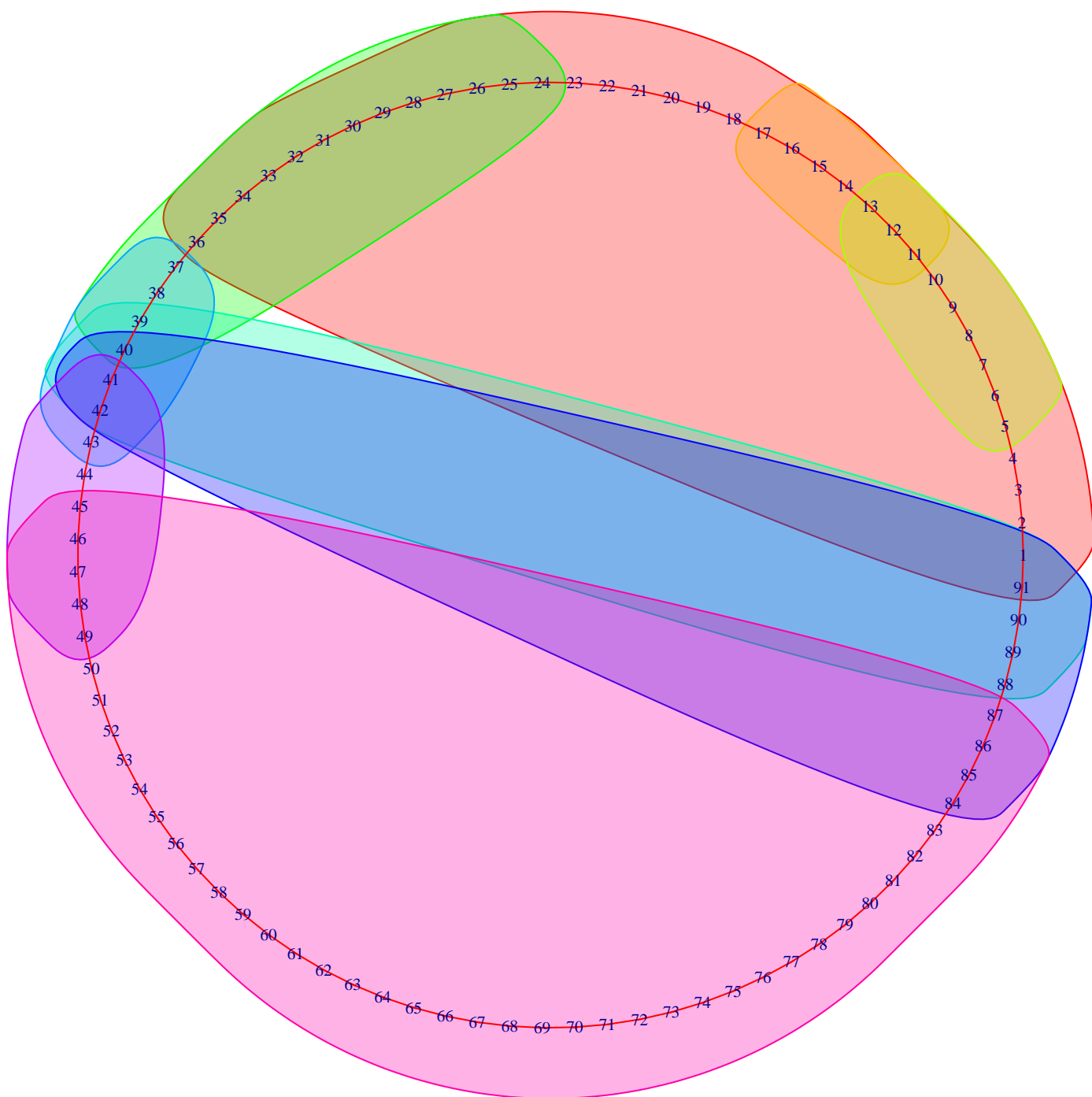

Supplement: Supplementary file 1 [file brainsci-09-00144-s001.zip › Supplementary 2/Mapper_graphs/133019_graph2B.pdf]

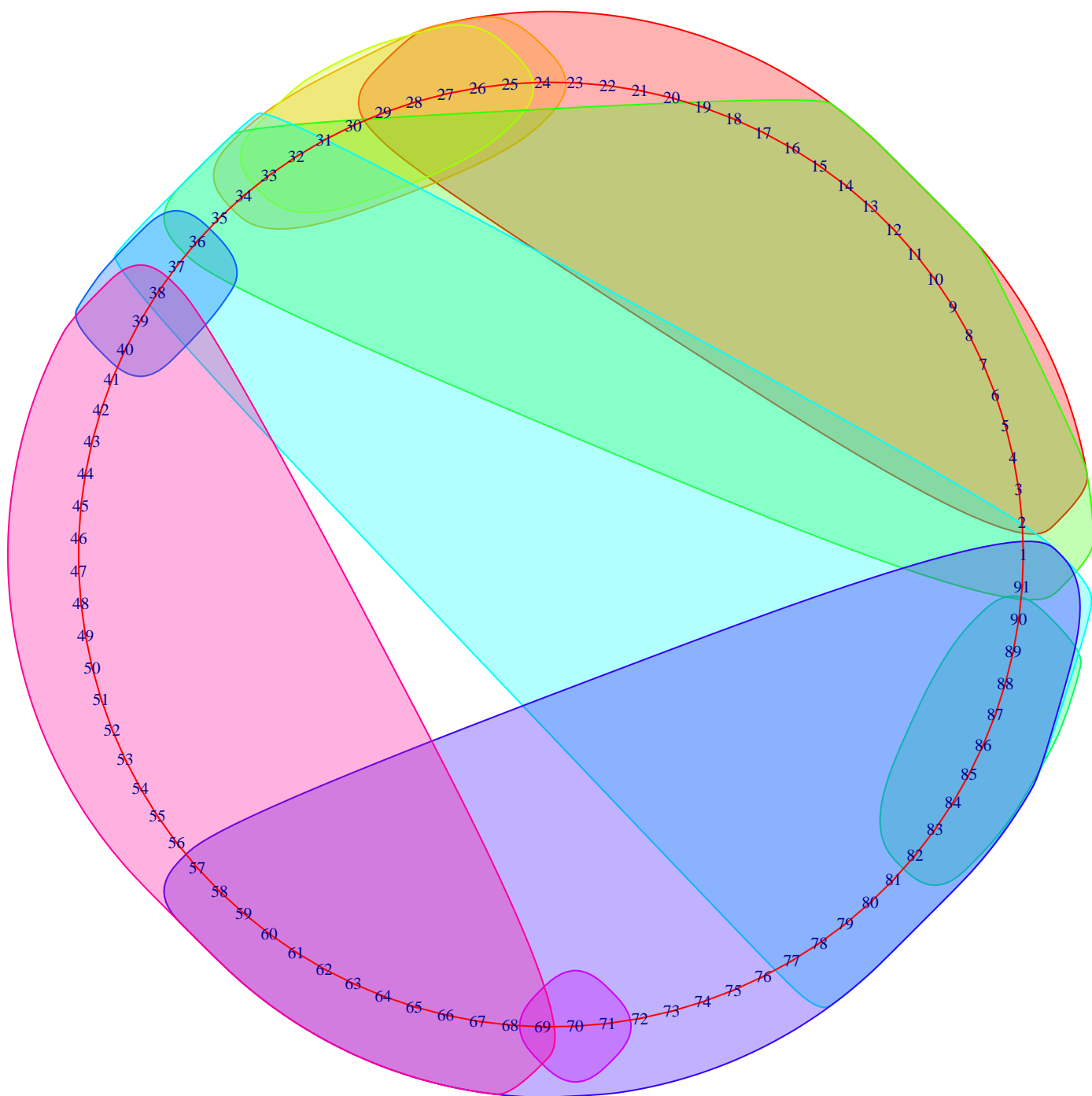

Supplement: Supplementary file 1 [file brainsci-09-00144-s001.zip › Supplementary 2/Mapper_graphs/116726_graph2B.pdf]

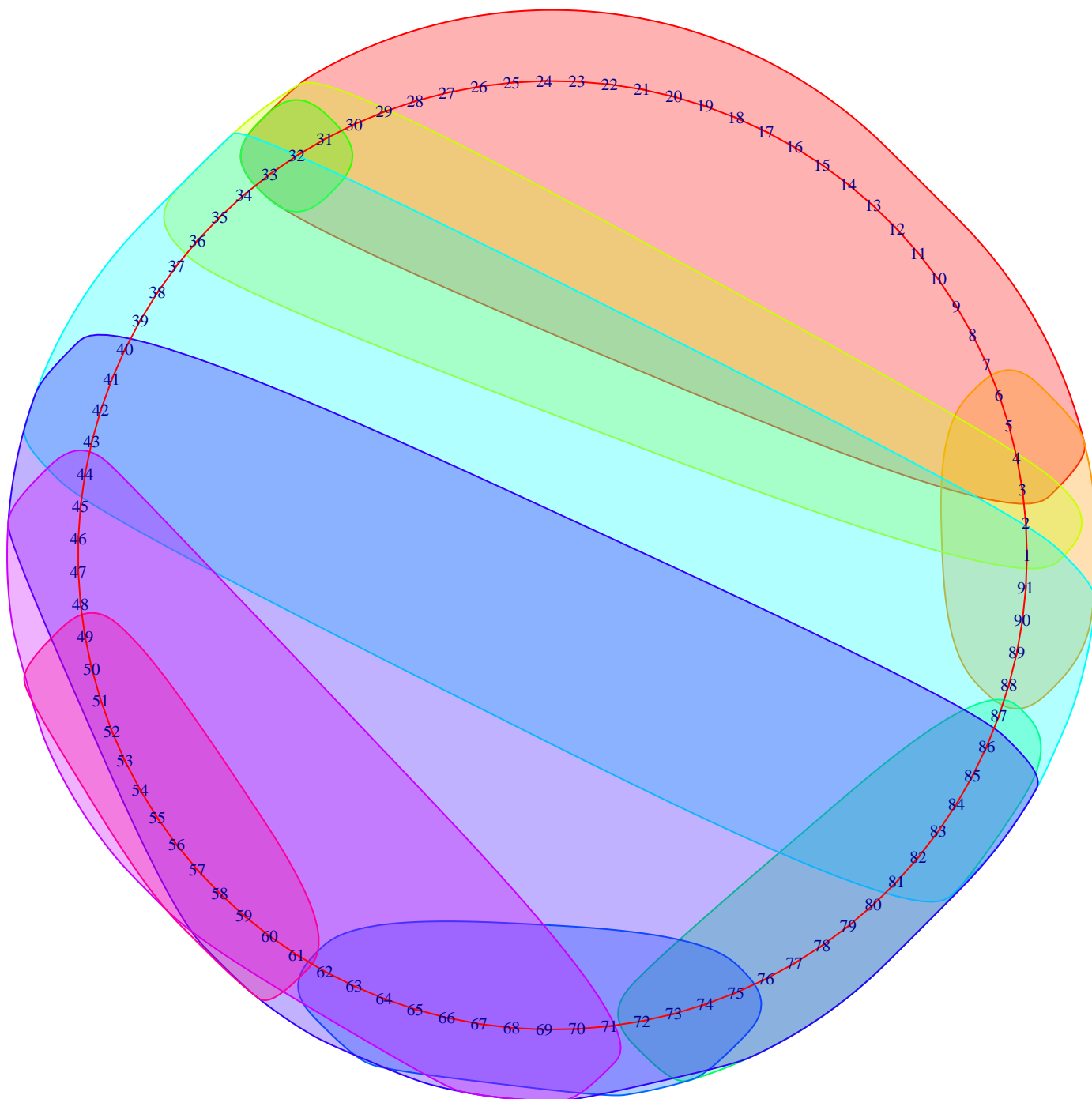

Supplement: Supplementary file 1 [file brainsci-09-00144-s001.zip › Supplementary 2/Mapper_graphs/912447_0B.pdf]

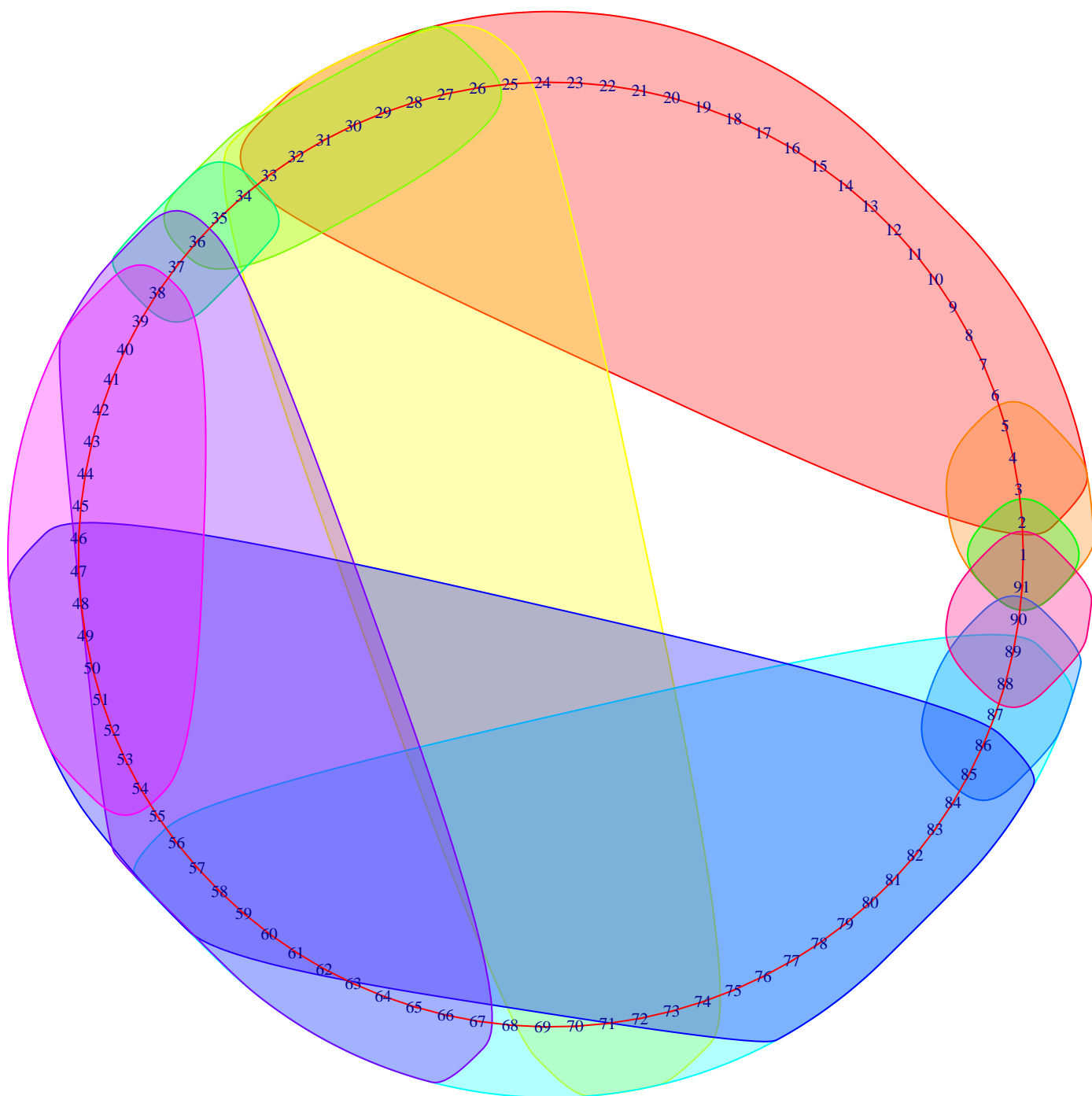

Supplement: Supplementary file 1 [file brainsci-09-00144-s001.zip › Supplementary 2/Mapper_graphs/111514_graph0B.pdf]

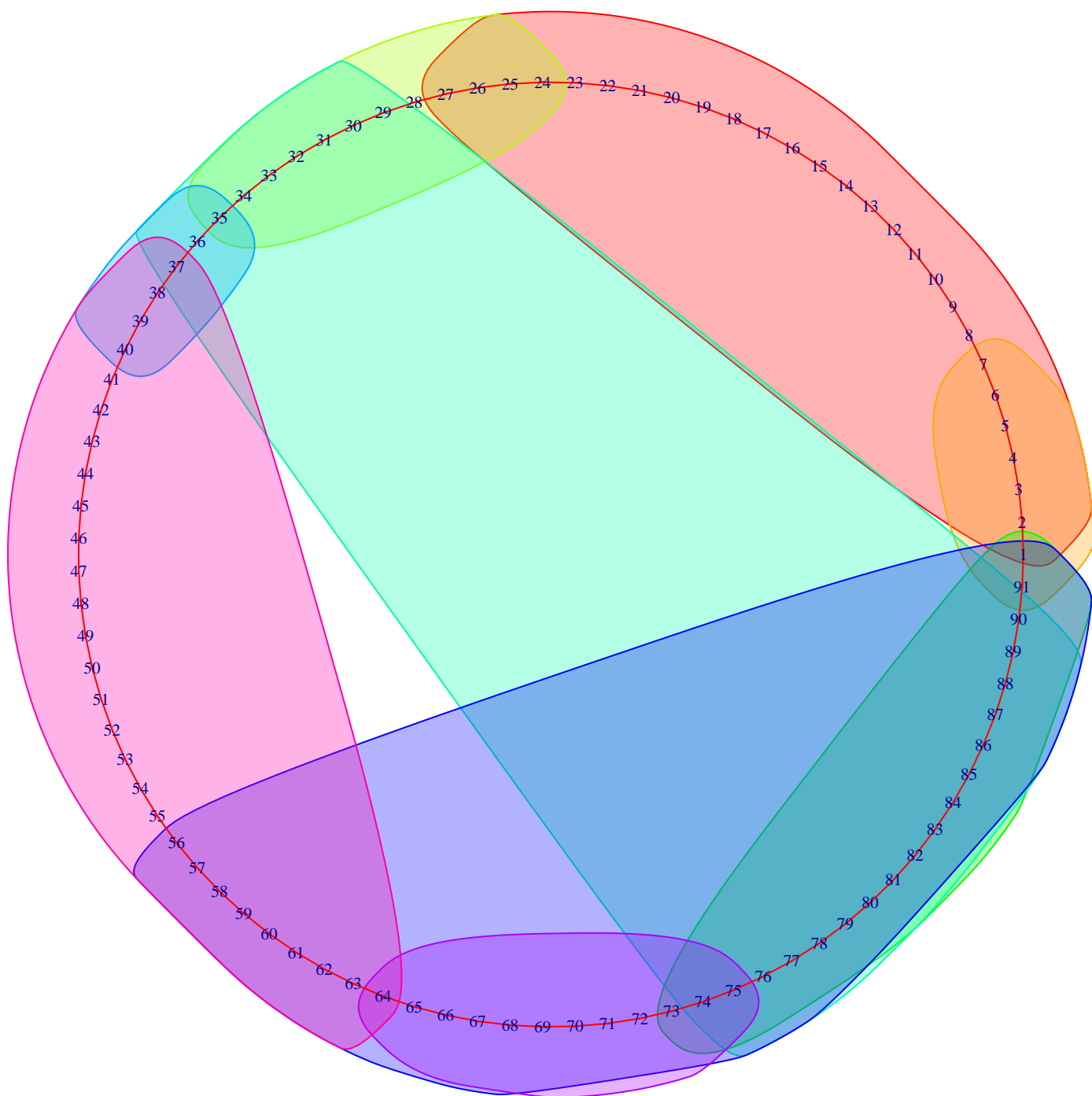

Supplement: Supplementary file 1 [file brainsci-09-00144-s001.zip › Supplementary 2/Mapper_graphs/166438_graph0B.pdf]

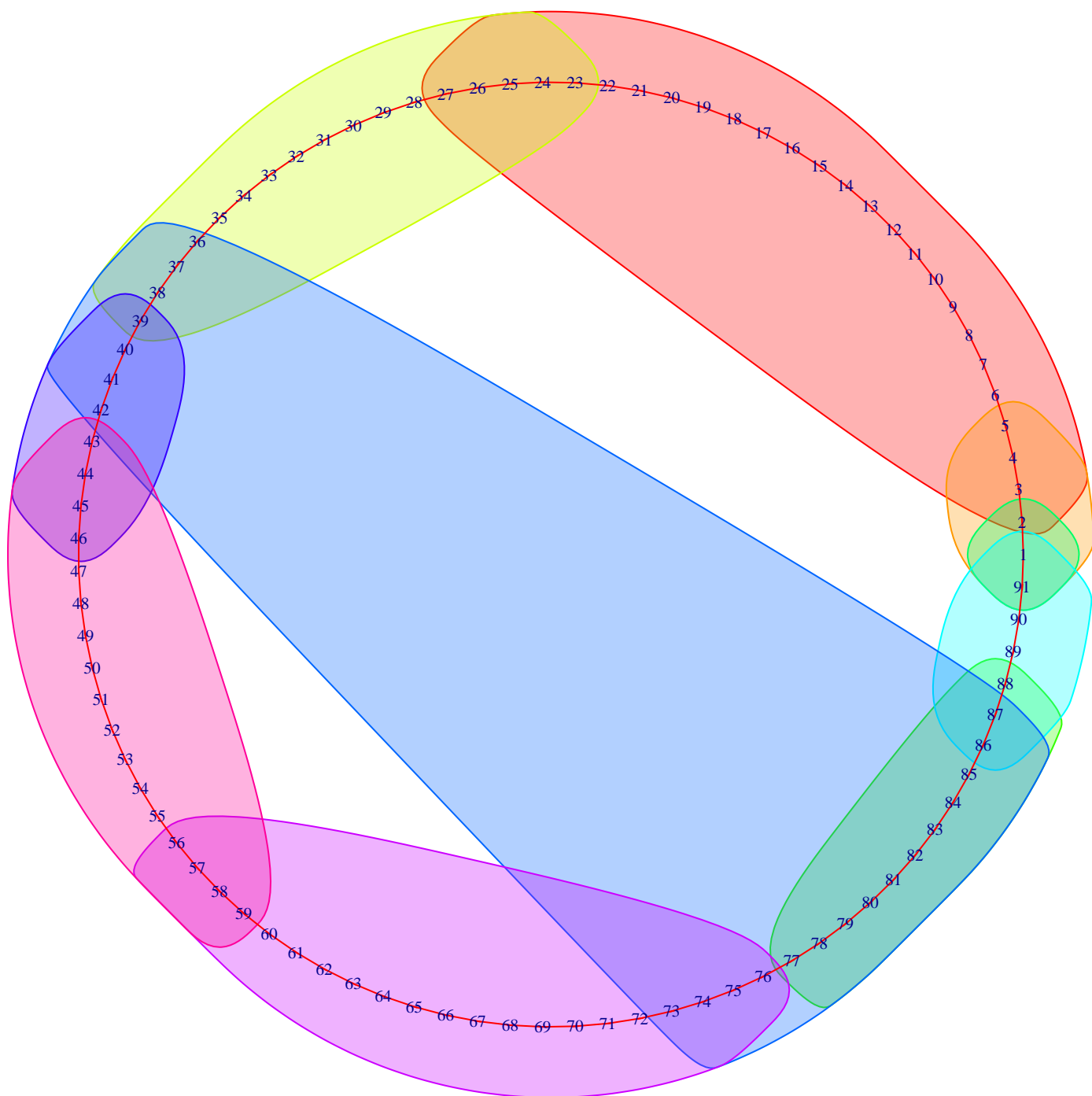

Supplement: Supplementary file 1 [file brainsci-09-00144-s001.zip › Supplementary 2/Mapper_graphs/353740_graph2B.pdf]

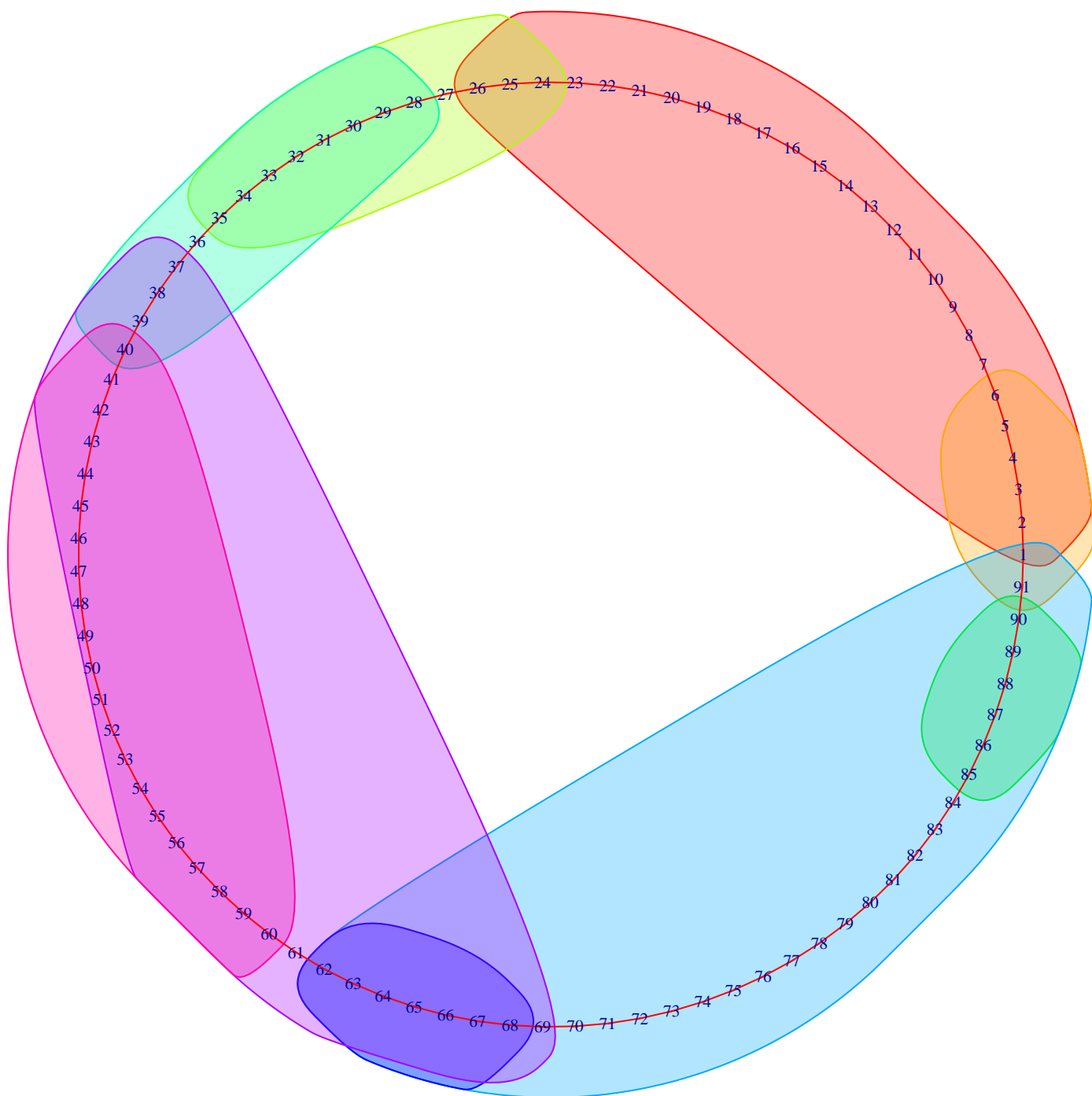

Supplement: Supplementary file 1 [file brainsci-09-00144-s001.zip › Supplementary 2/Mapper_graphs/715950_0B.pdf]

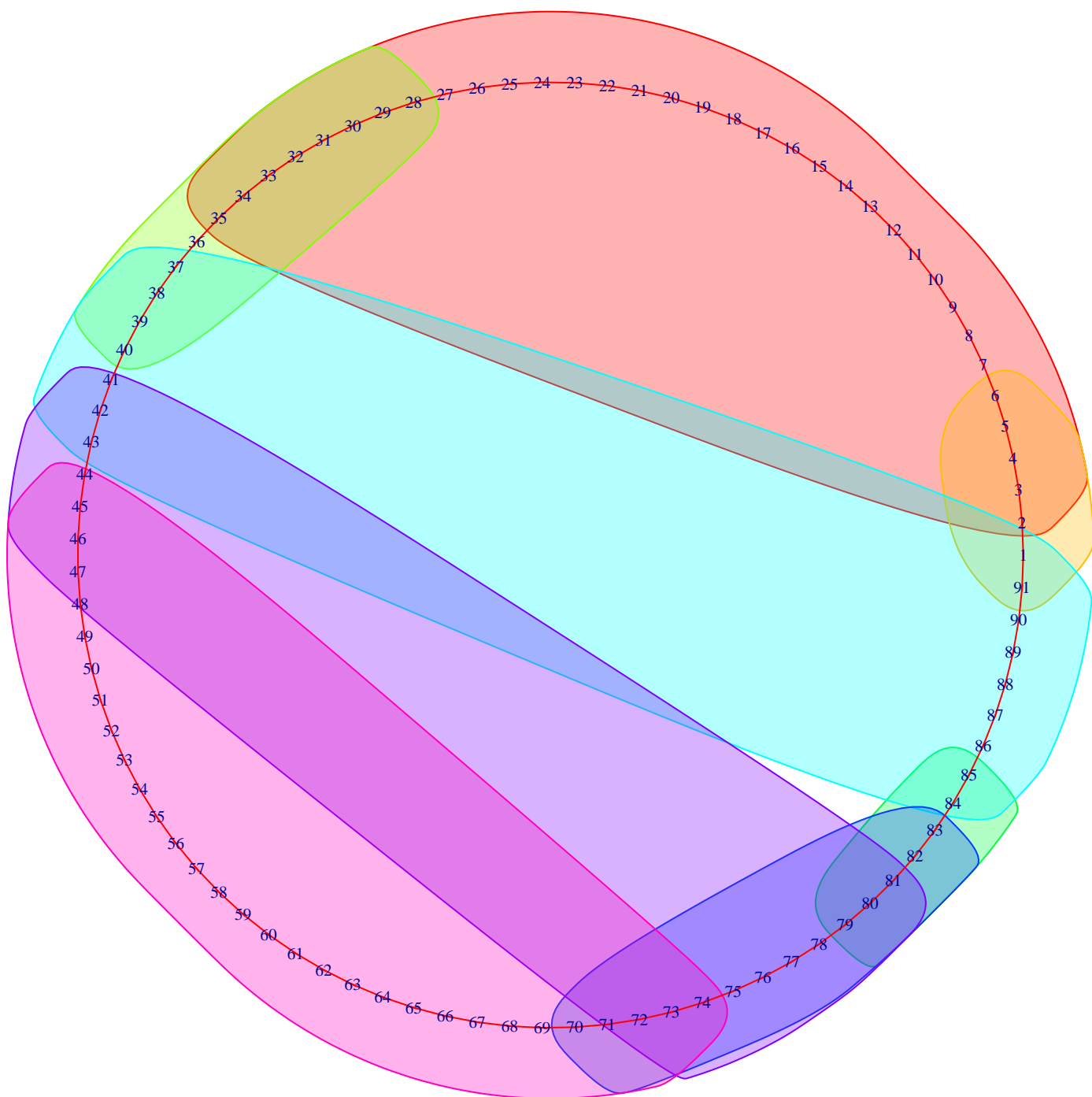

Supplement: Supplementary file 1 [file brainsci-09-00144-s001.zip › Supplementary 2/Mapper_graphs/725751_2B.pdf]

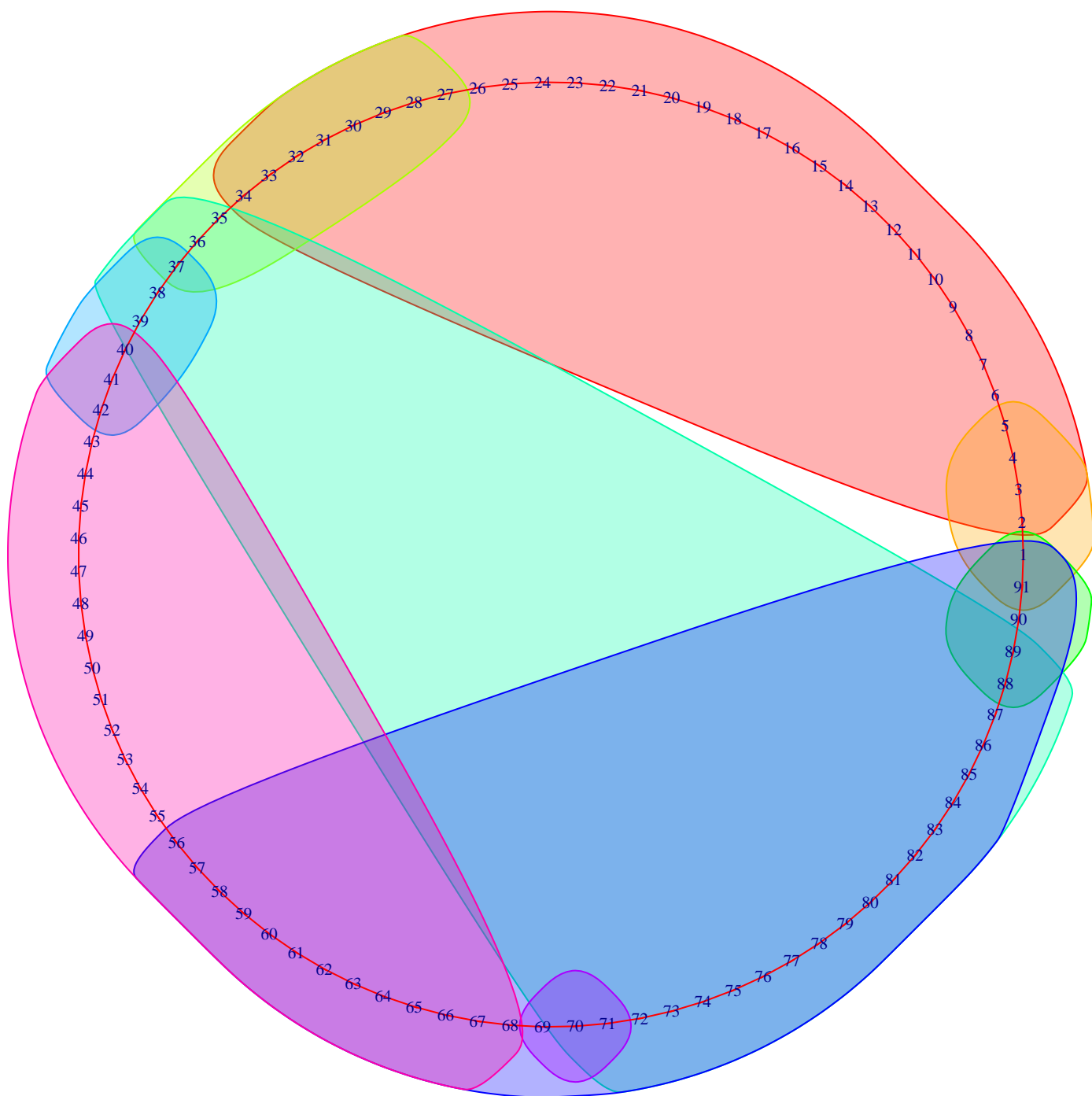

Supplement: Supplementary file 1 [file brainsci-09-00144-s001.zip › Supplementary 2/Mapper_graphs/156334_graph2B.pdf]

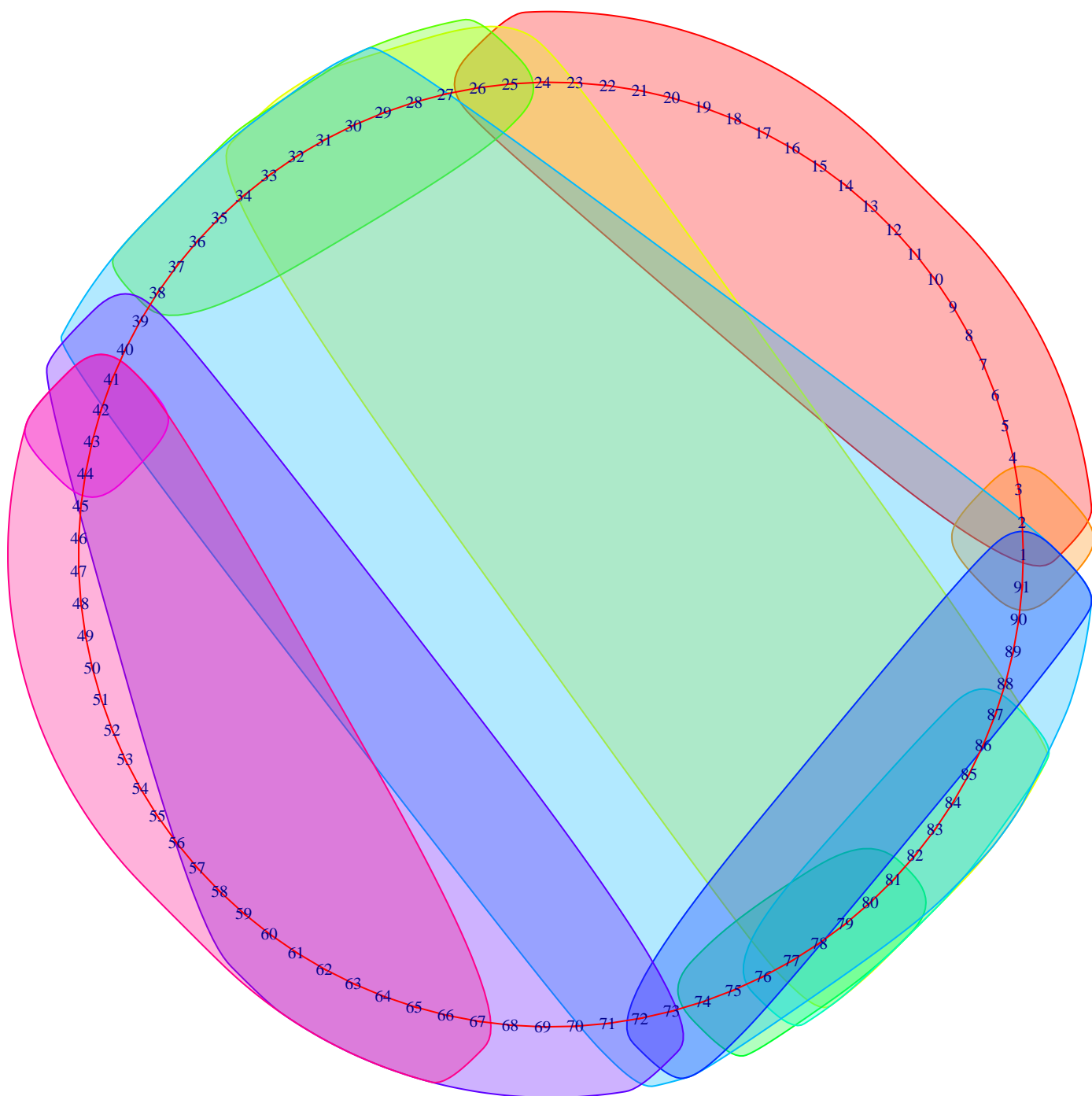

Supplement: Supplementary file 1 [file brainsci-09-00144-s001.zip › Supplementary 2/Mapper_graphs/735148_2B.pdf]

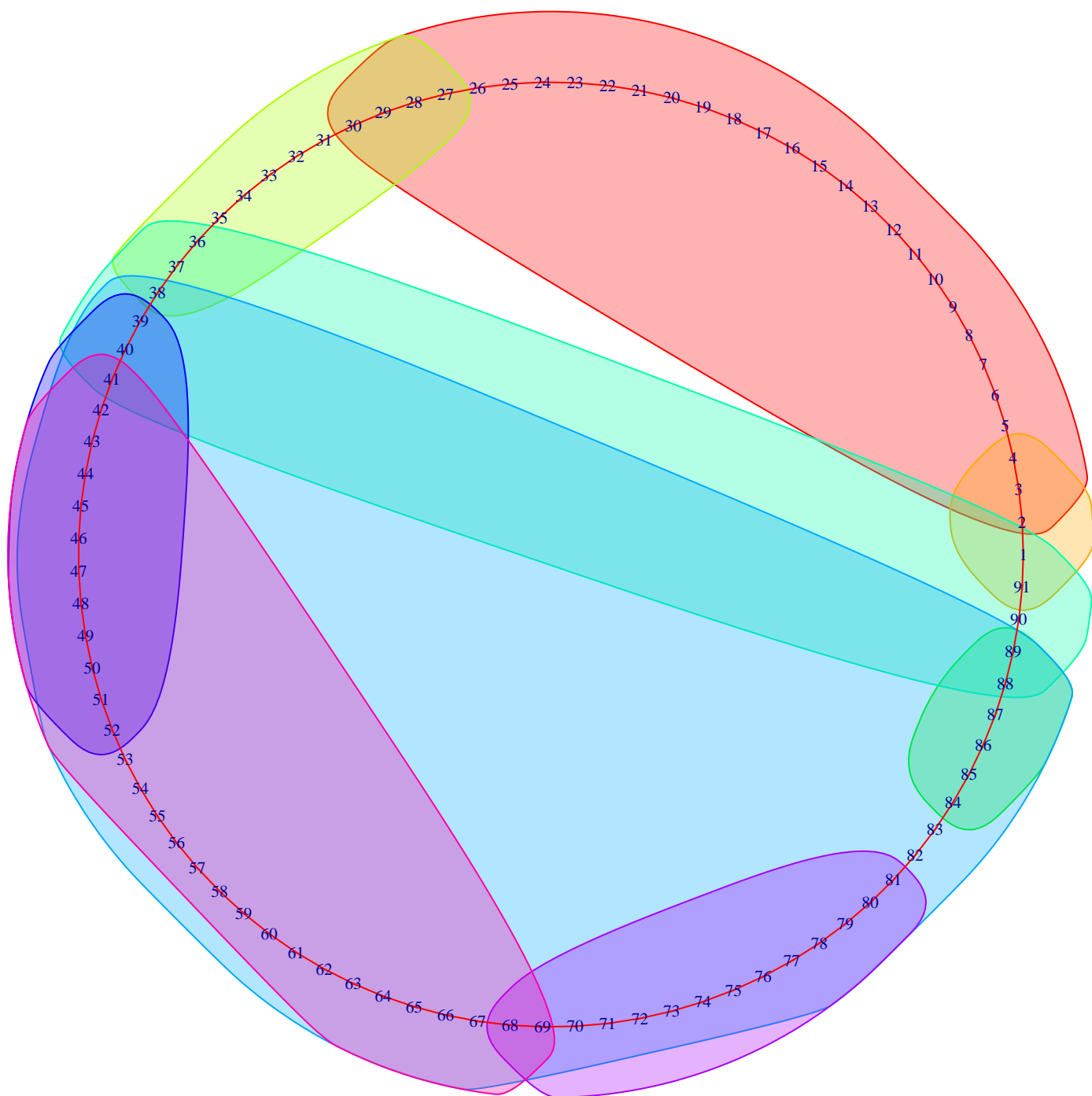

Supplement: Supplementary file 1 [file brainsci-09-00144-s001.zip › Supplementary 2/Mapper_graphs/783462_2B.pdf]

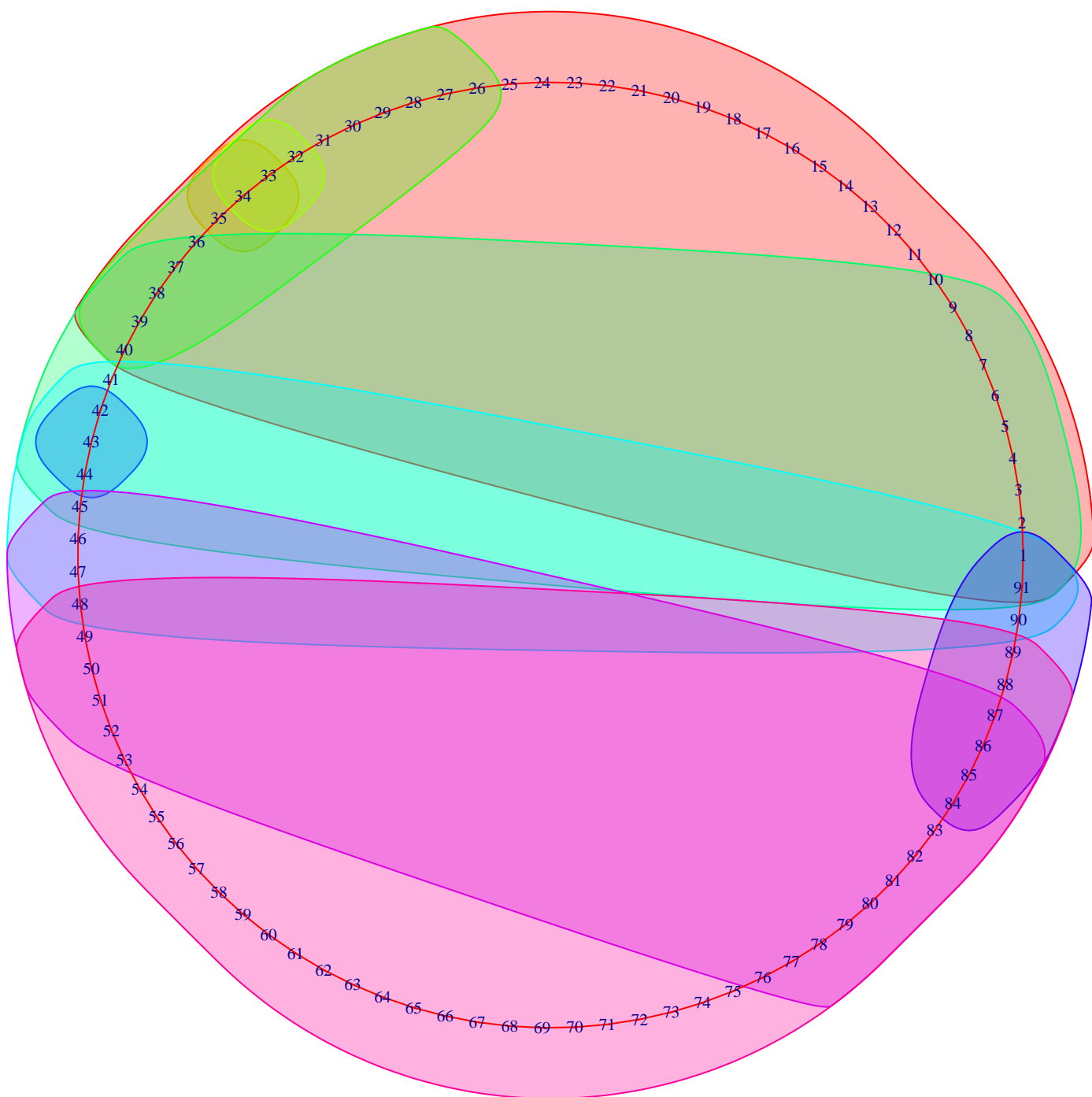

Supplement: Supplementary file 1 [file brainsci-09-00144-s001.zip › Supplementary 2/Mapper_graphs/660951_2B.pdf]

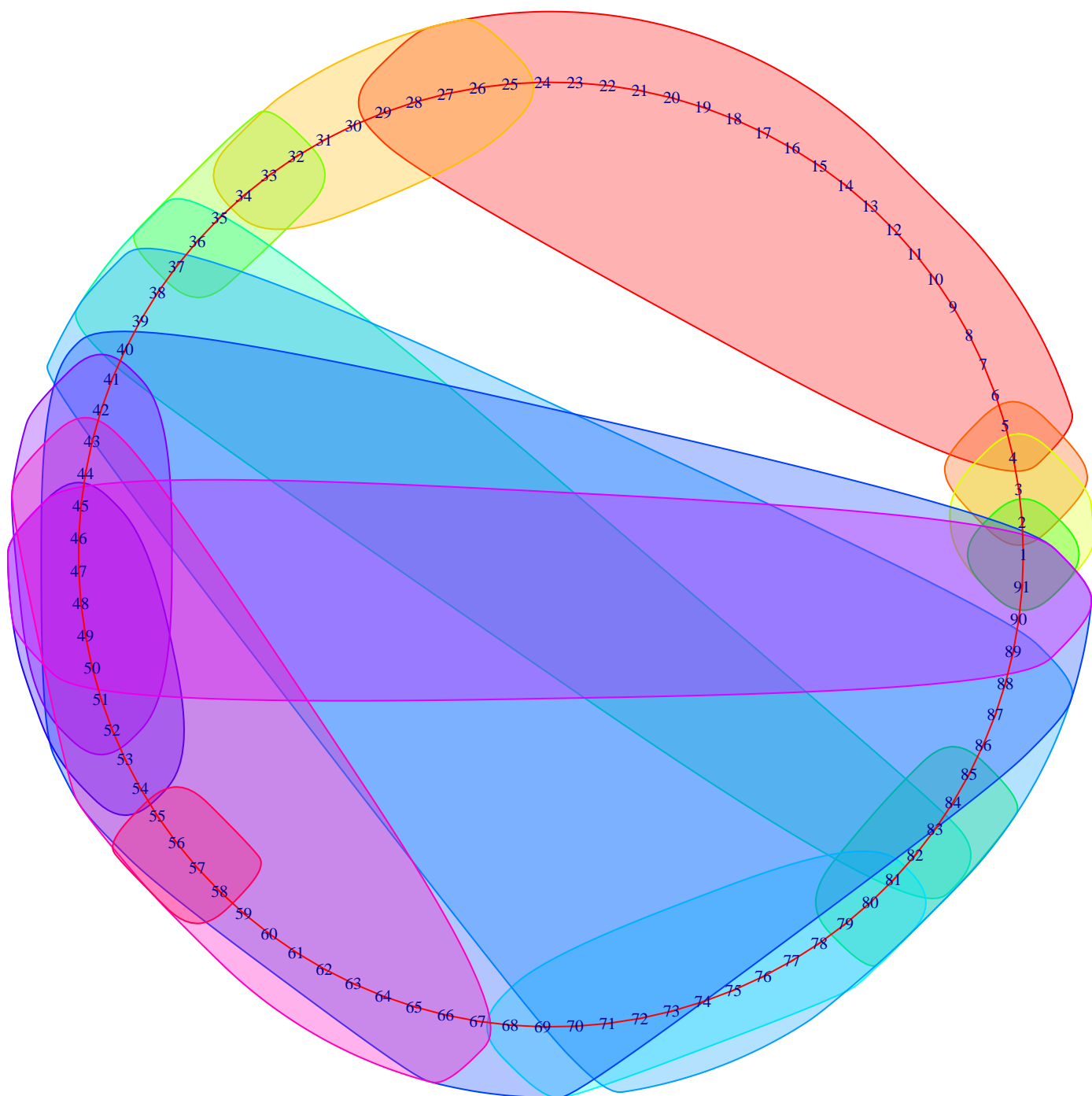

Supplement: Supplementary file 1 [file brainsci-09-00144-s001.zip › Supplementary 2/Mapper_graphs/146129_graph2B.pdf]

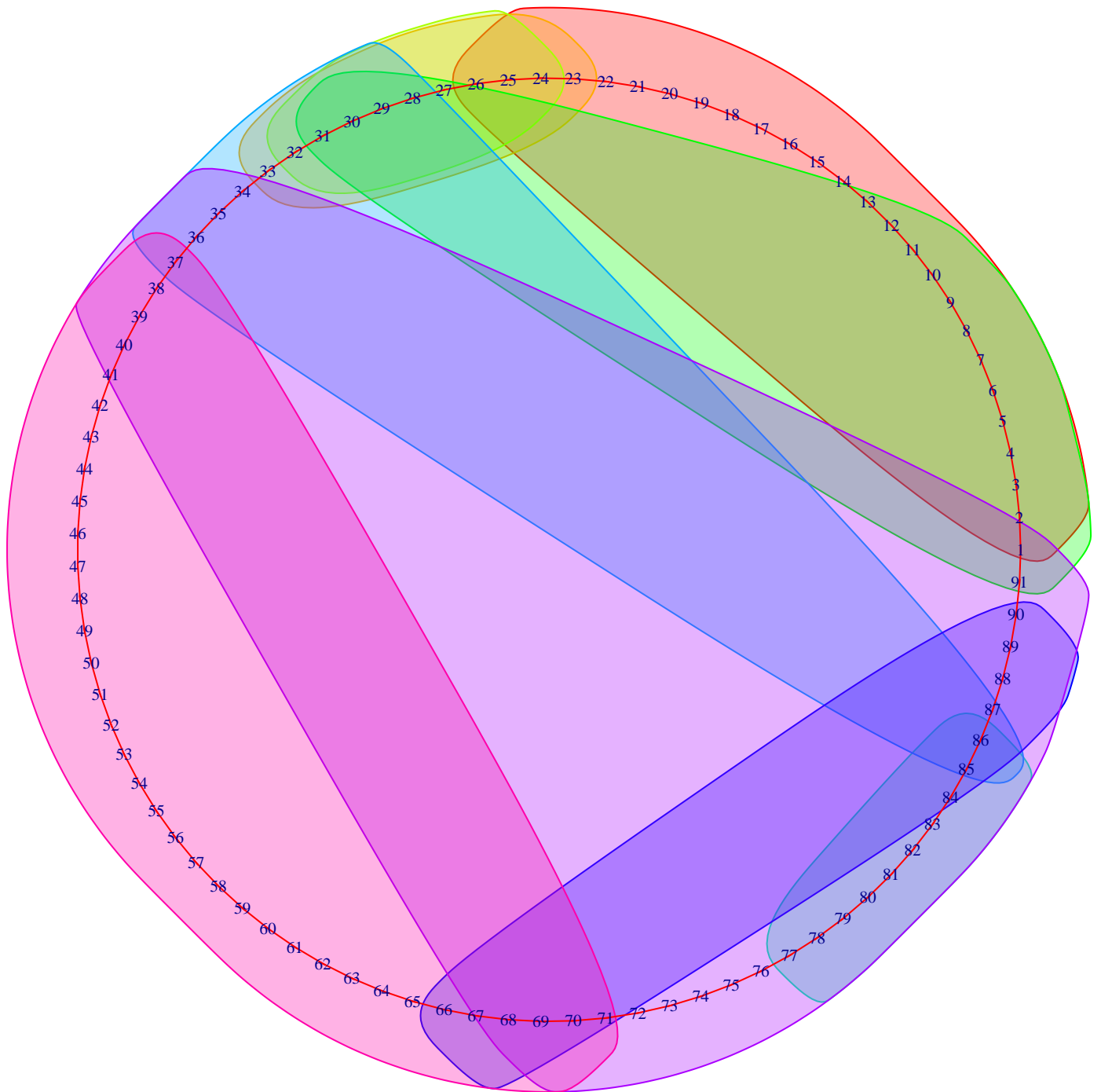

Supplement: Supplementary file 1 [file brainsci-09-00144-s001.zip › Supplementary 2/Mapper_graphs/108323_graph2B.pdf]

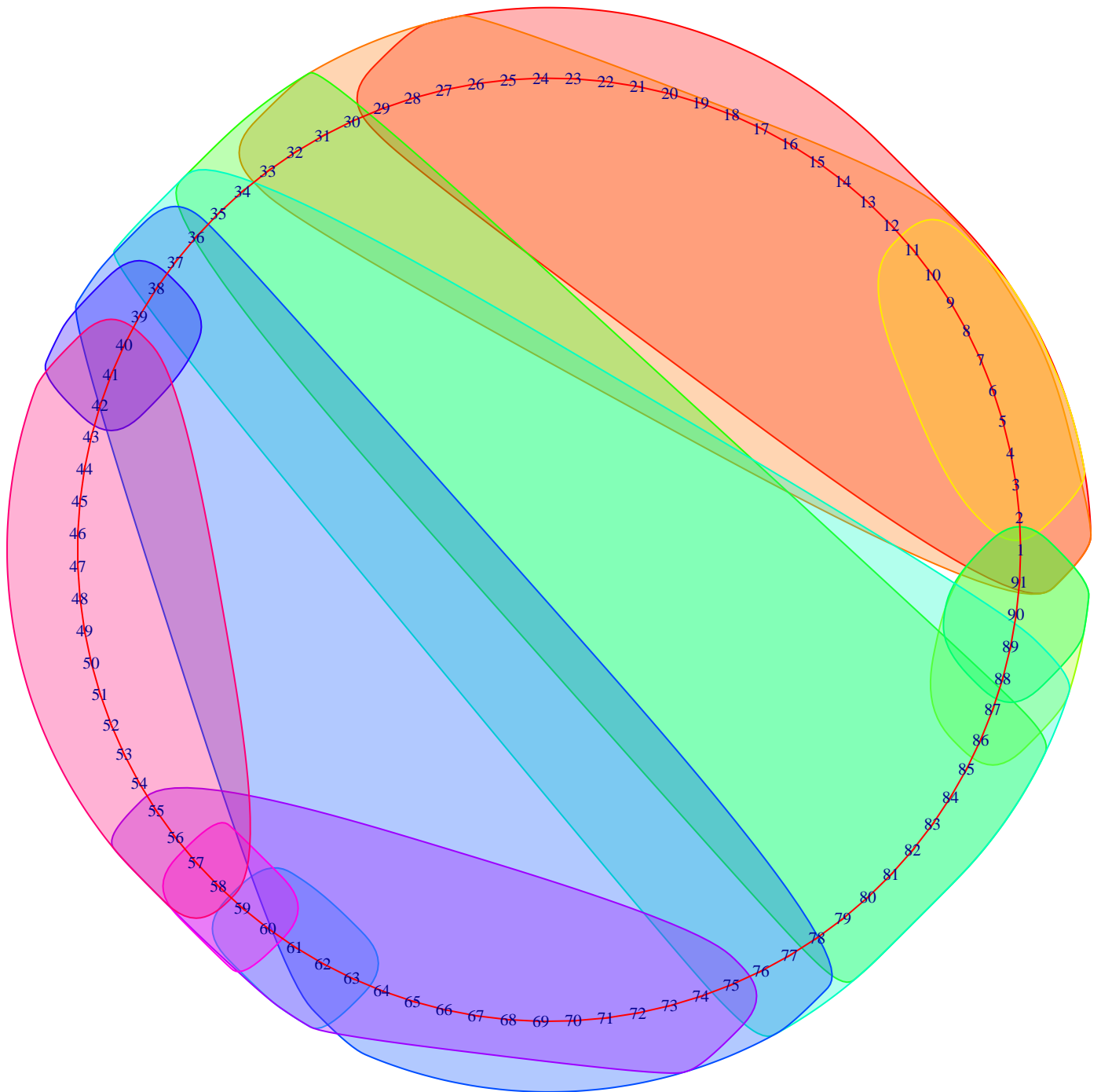

Supplement: Supplementary file 1 [file brainsci-09-00144-s001.zip › Supplementary 2/Mapper_graphs/185442_2B.pdf]

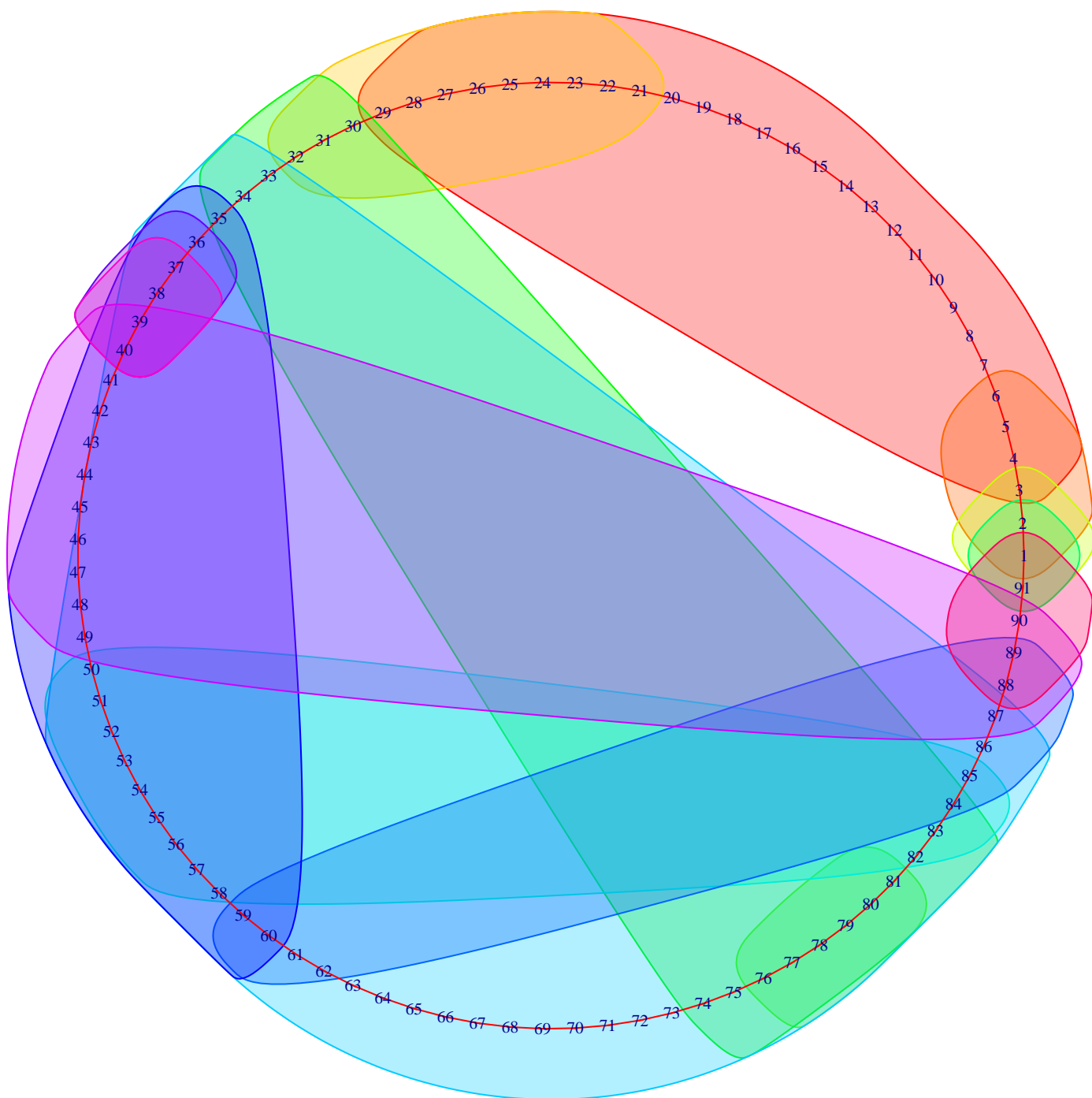

Supplement: Supplementary file 1 [file brainsci-09-00144-s001.zip › Supplementary 2/Mapper_graphs/140117_graph2B.pdf]

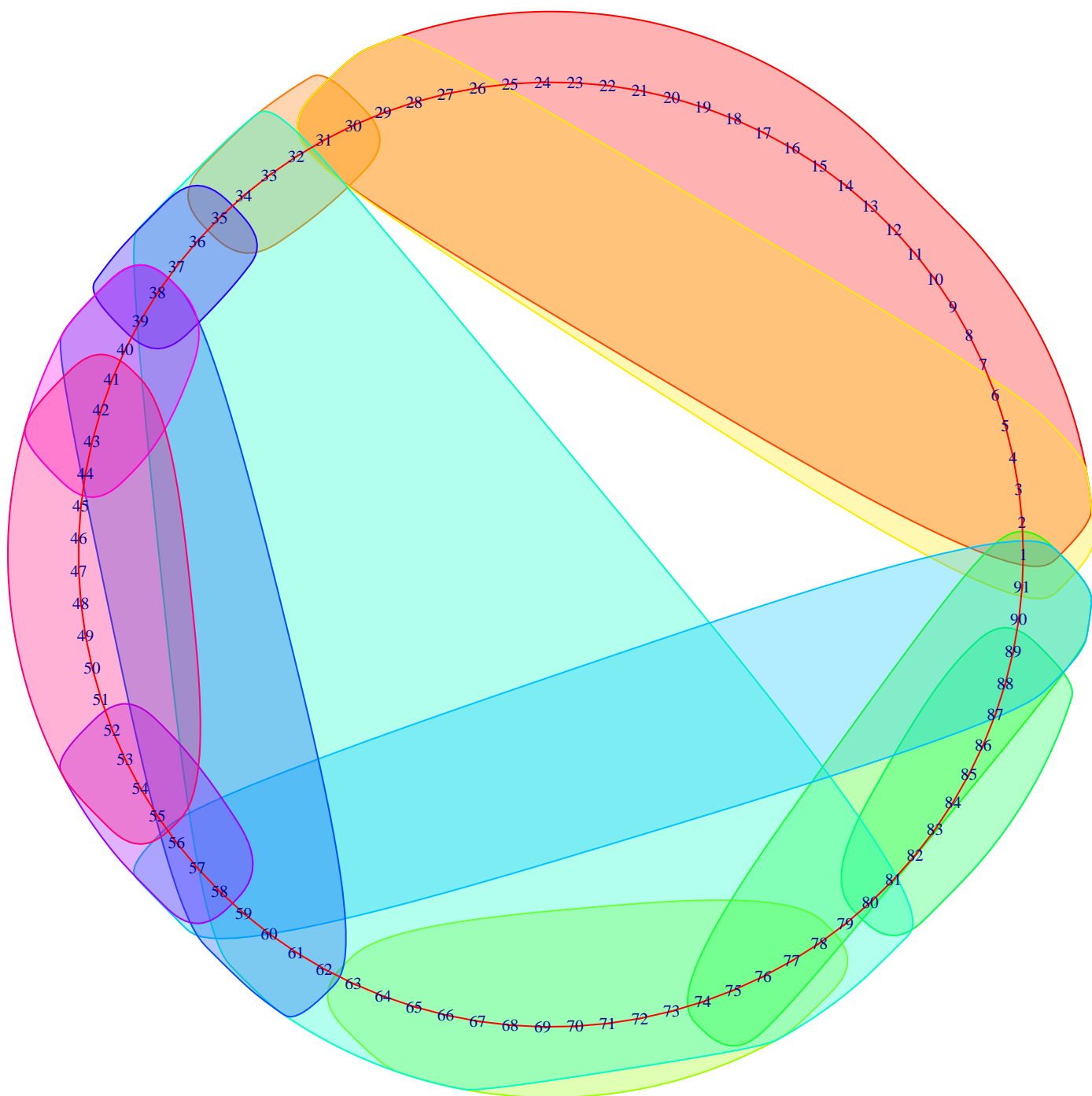

Supplement: Supplementary file 1 [file brainsci-09-00144-s001.zip › Supplementary 2/Mapper_graphs/352738_0B.pdf]

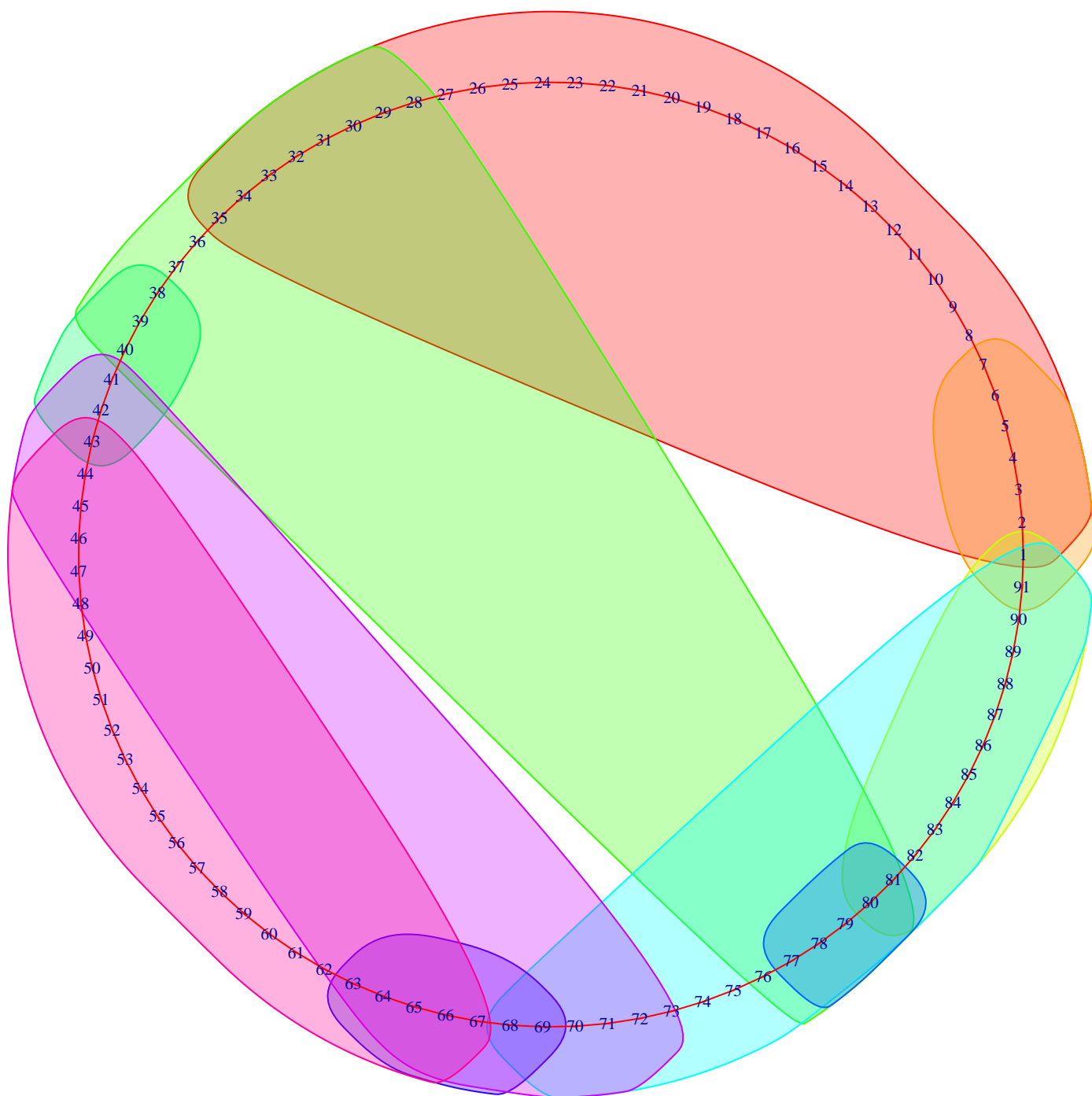

Supplement: Supplementary file 1 [file brainsci-09-00144-s001.zip › Supplementary 2/Mapper_graphs/283543_0B.pdf]

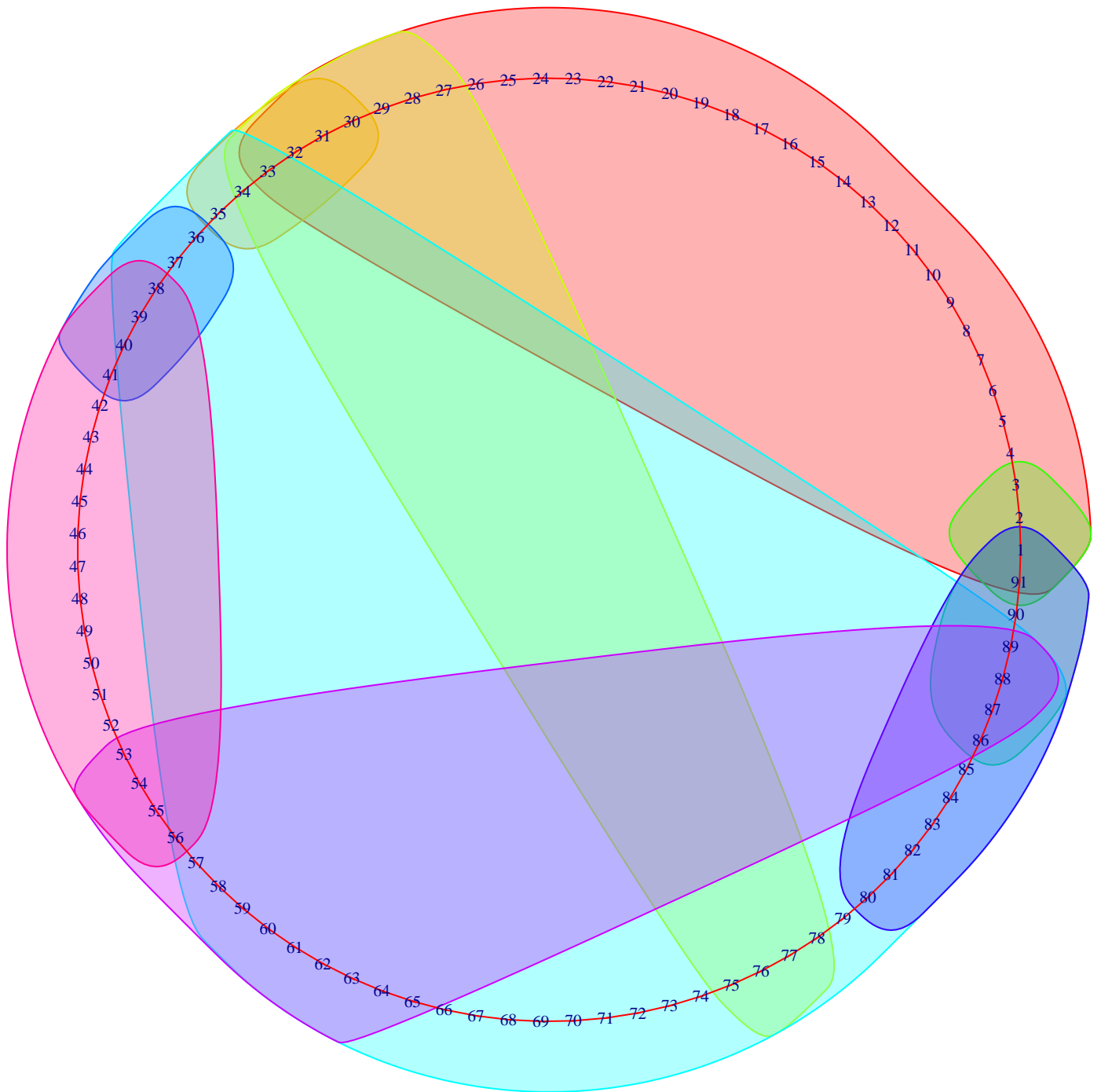

Supplement: Supplementary file 1 [file brainsci-09-00144-s001.zip › Supplementary 2/Mapper_graphs/191033_0B.pdf]

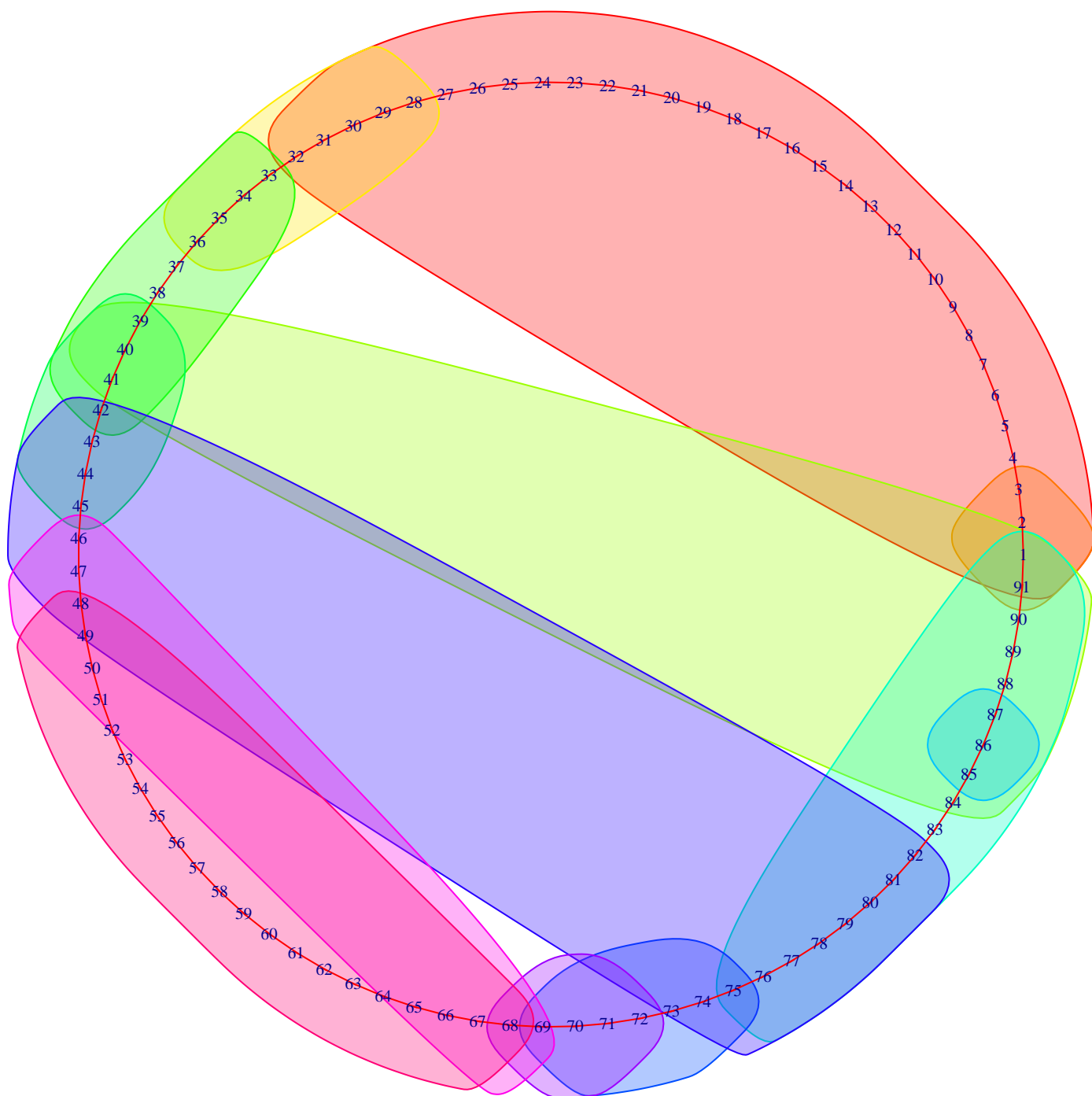

Supplement: Supplementary file 1 [file brainsci-09-00144-s001.zip › Supplementary 2/Mapper_graphs/175237_graph2B.pdf]

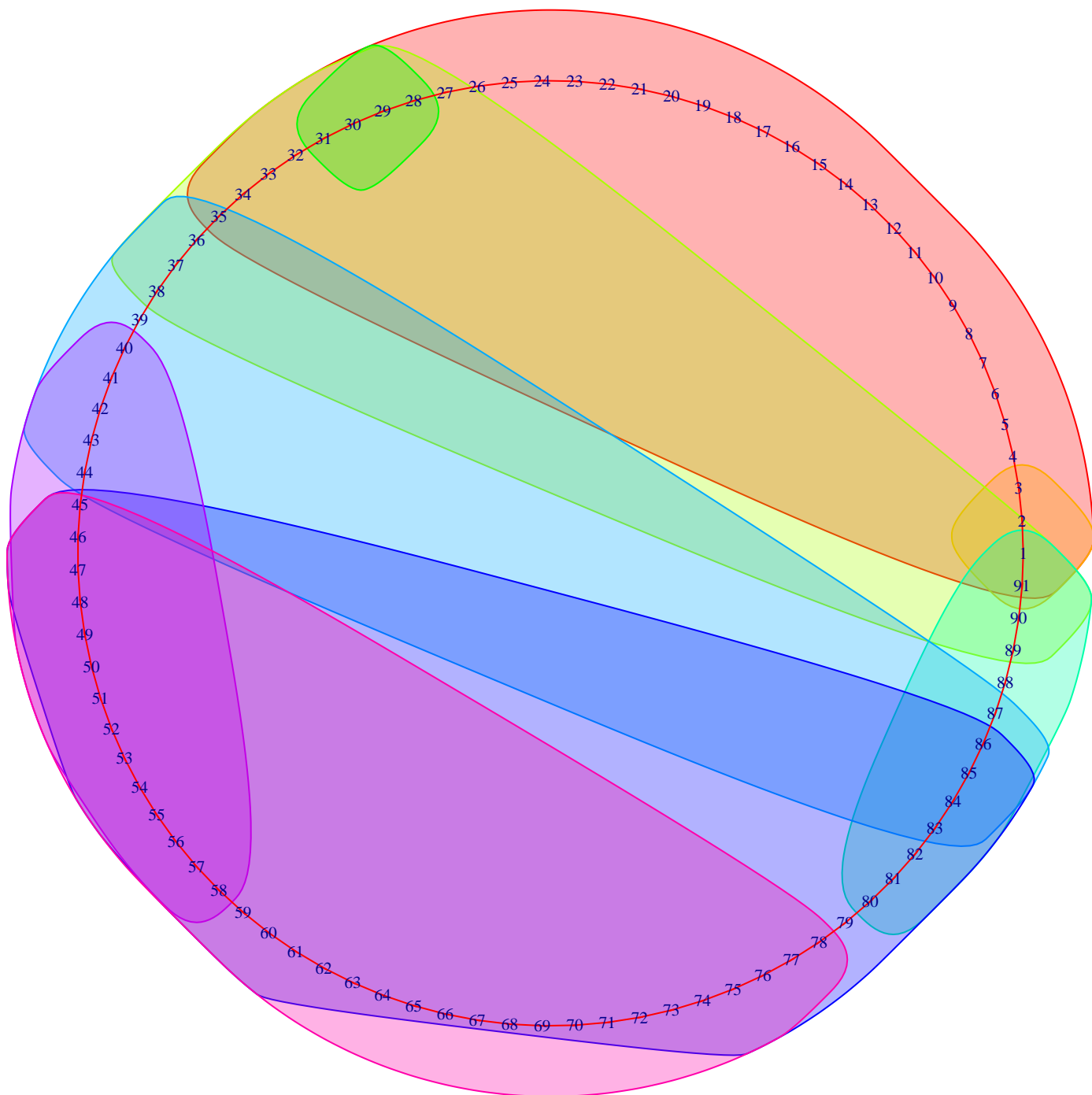

Supplement: Supplementary file 1 [file brainsci-09-00144-s001.zip › Supplementary 2/Mapper_graphs/204521_2B.pdf]

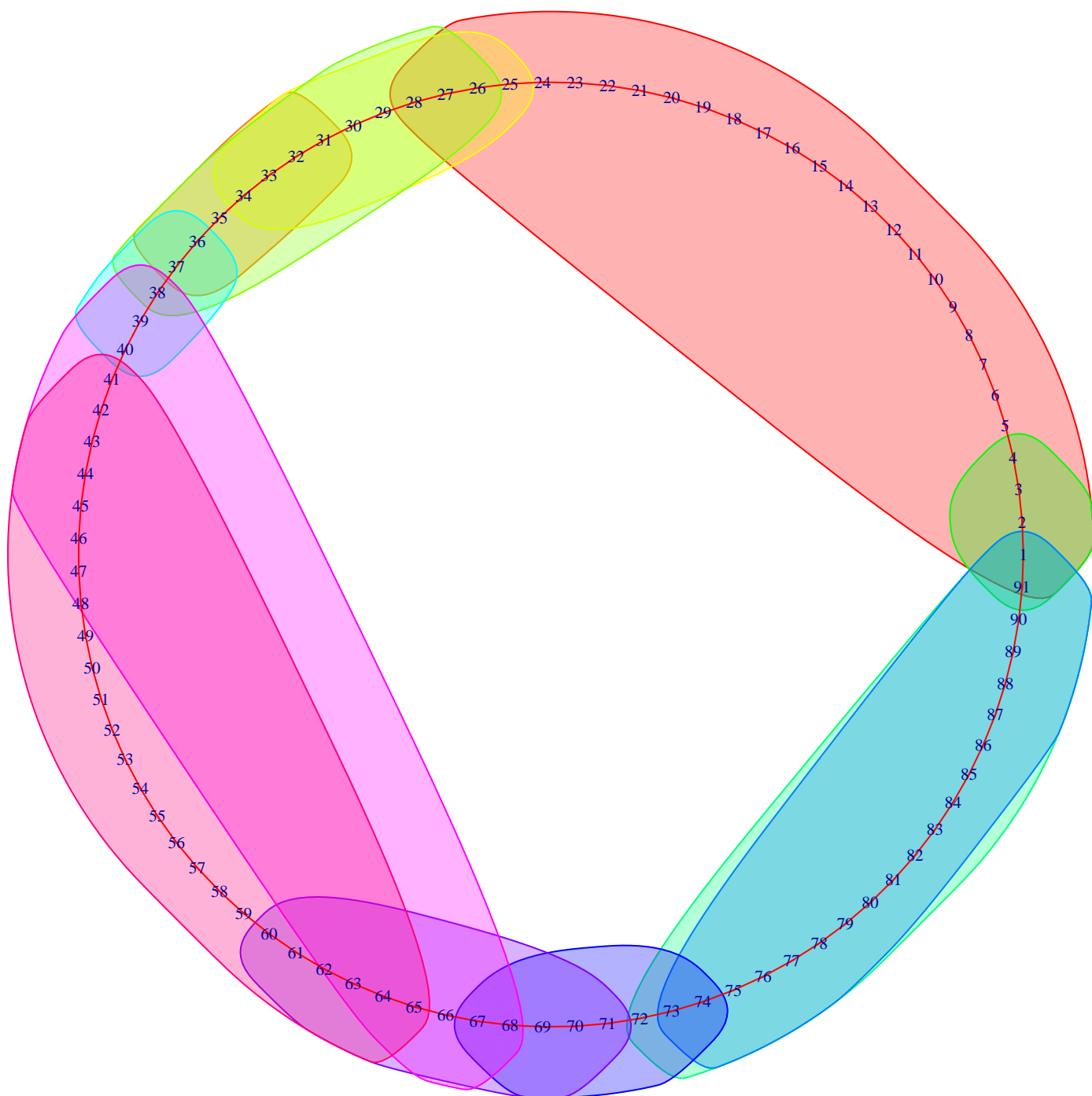

Supplement: Supplementary file 1 [file brainsci-09-00144-s001.zip › Supplementary 2/Mapper_graphs/175540_graph0B.pdf]

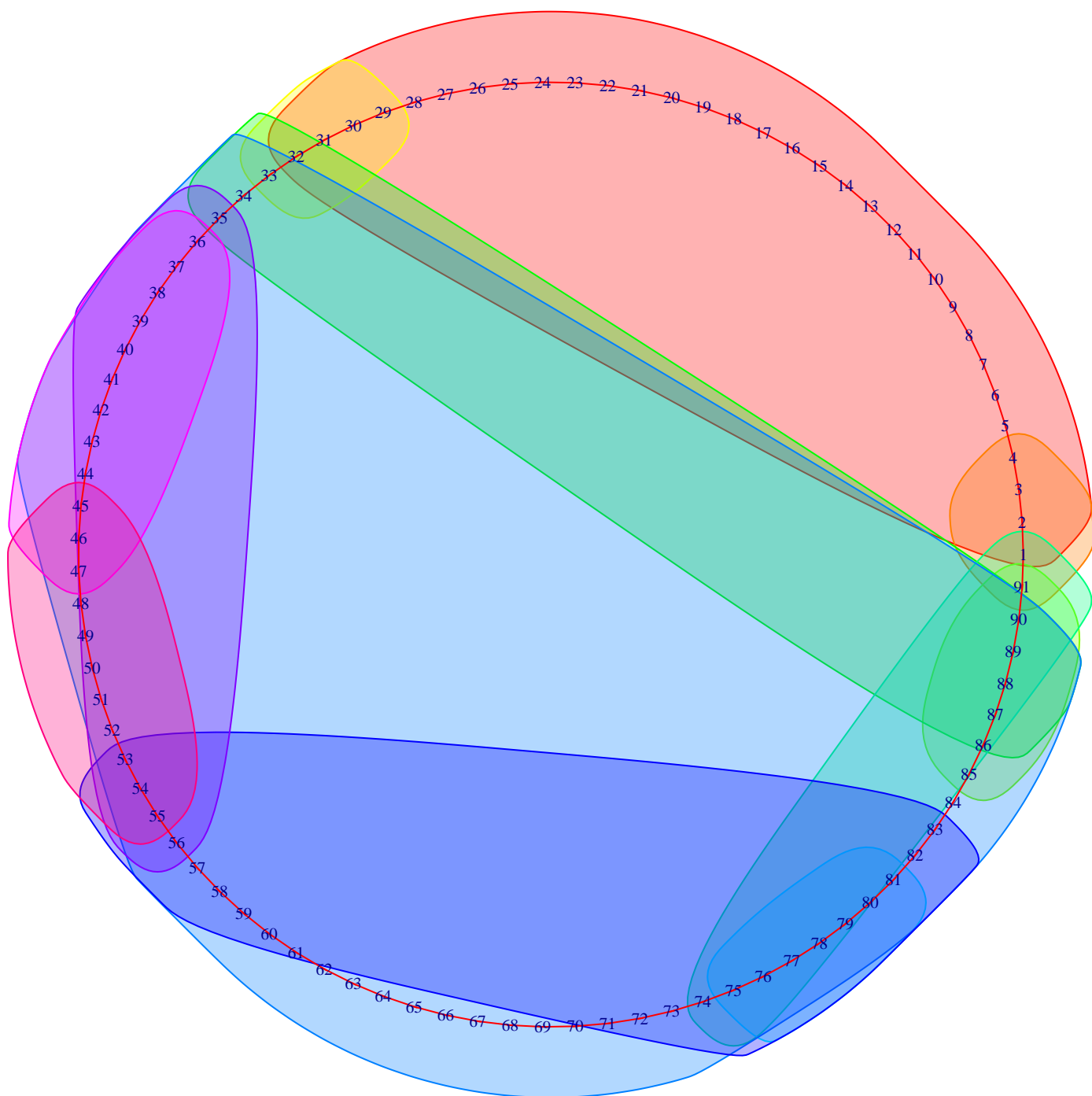

Supplement: Supplementary file 1 [file brainsci-09-00144-s001.zip › Supplementary 2/Mapper_graphs/192641_0B.pdf]

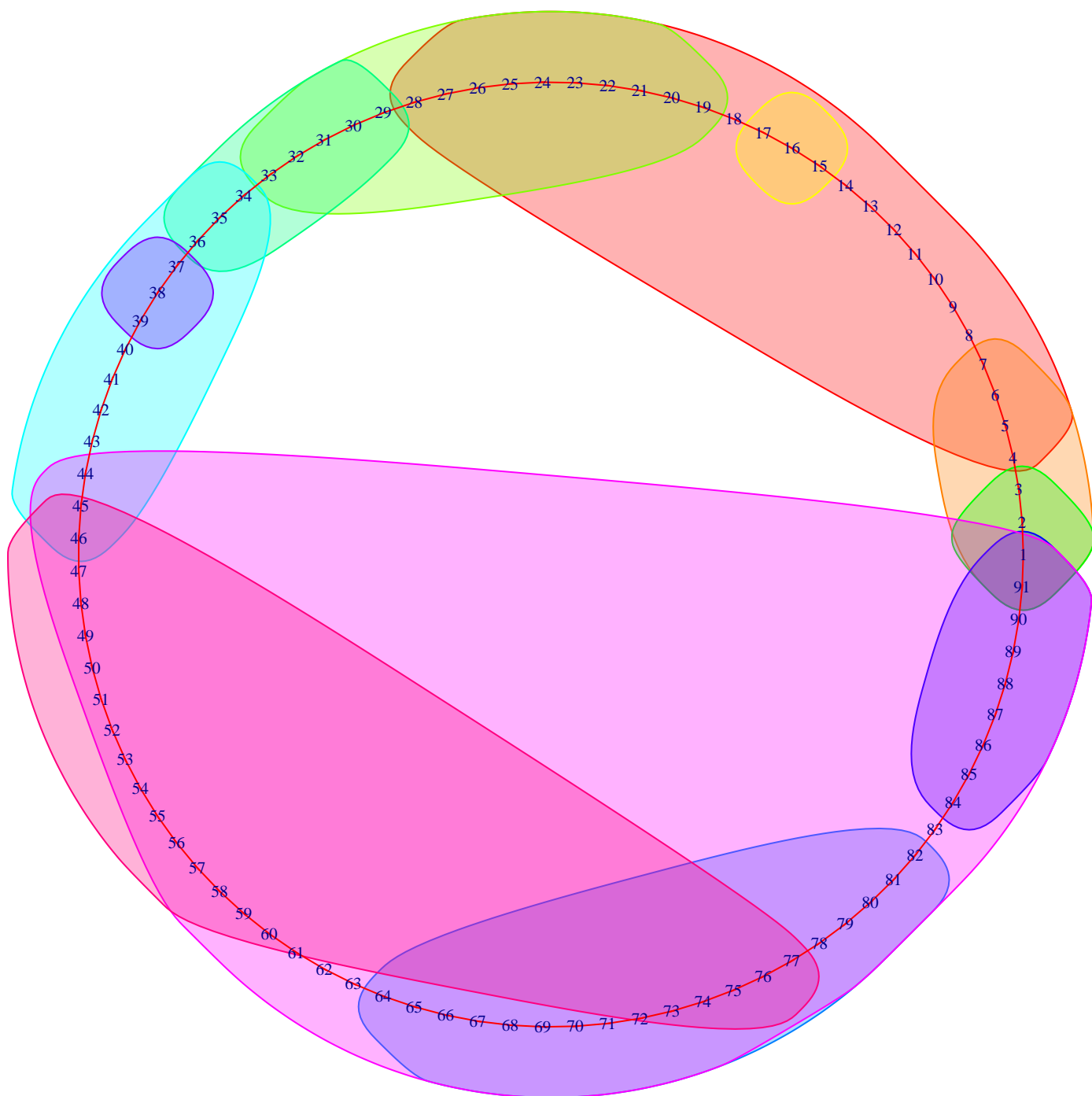

Supplement: Supplementary file 1 [file brainsci-09-00144-s001.zip › Supplementary 2/Mapper_graphs/112920_graph2B.pdf]

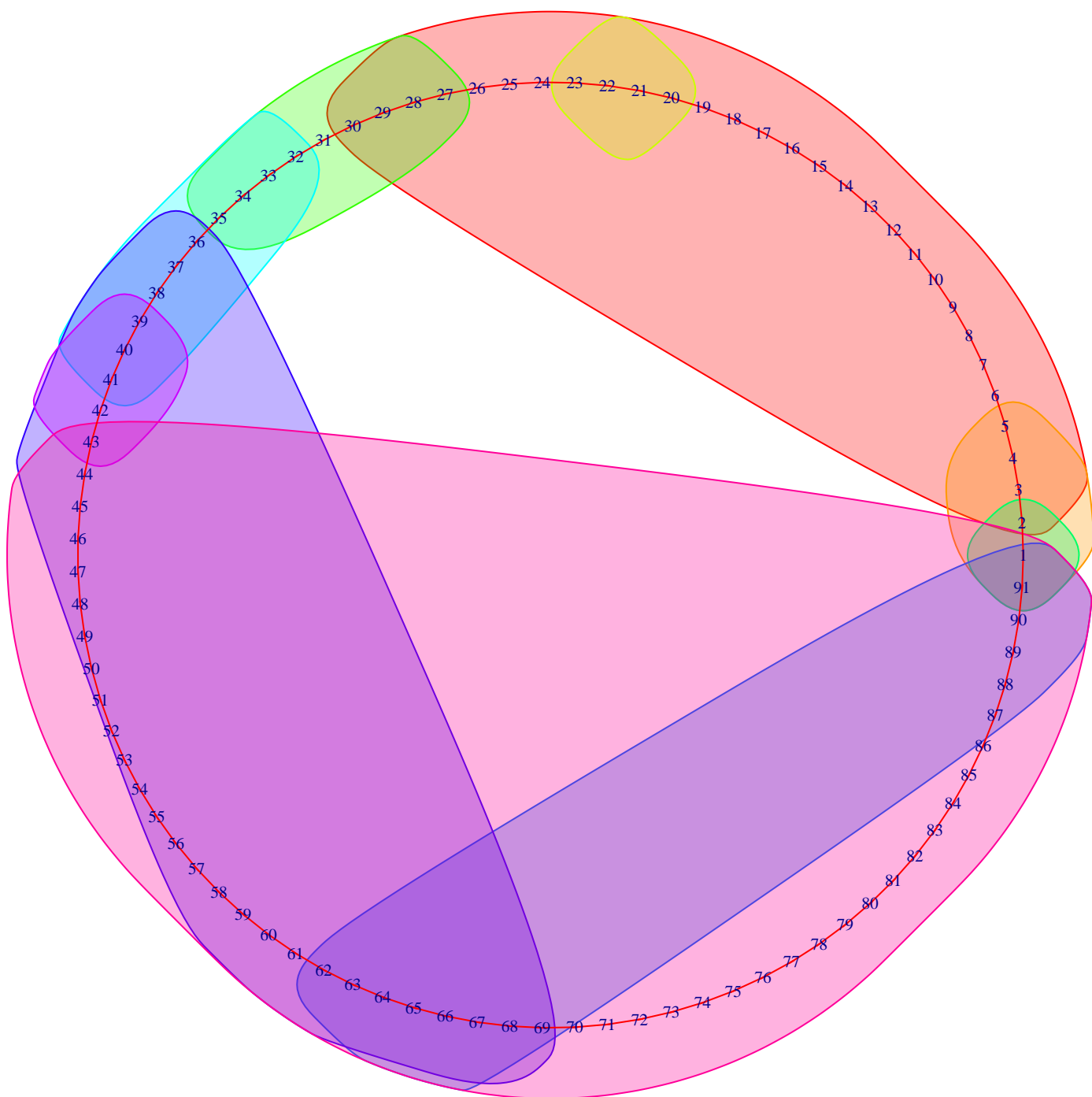

Supplement: Supplementary file 1 [file brainsci-09-00144-s001.zip › Supplementary 2/Mapper_graphs/172029_graph2B.pdf]

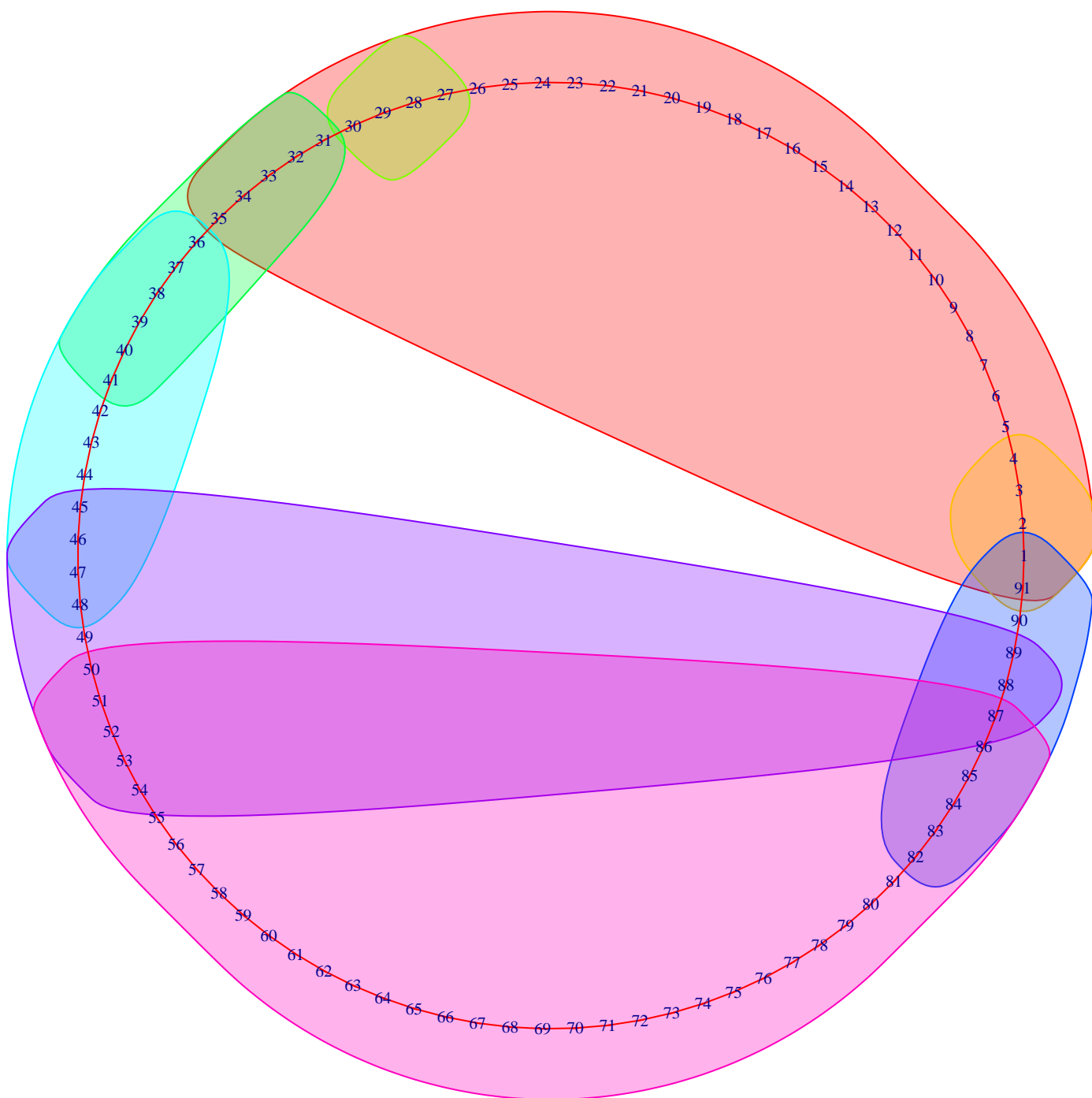

Supplement: Supplementary file 1 [file brainsci-09-00144-s001.zip › Supplementary 2/Mapper_graphs/250427_0B.pdf]

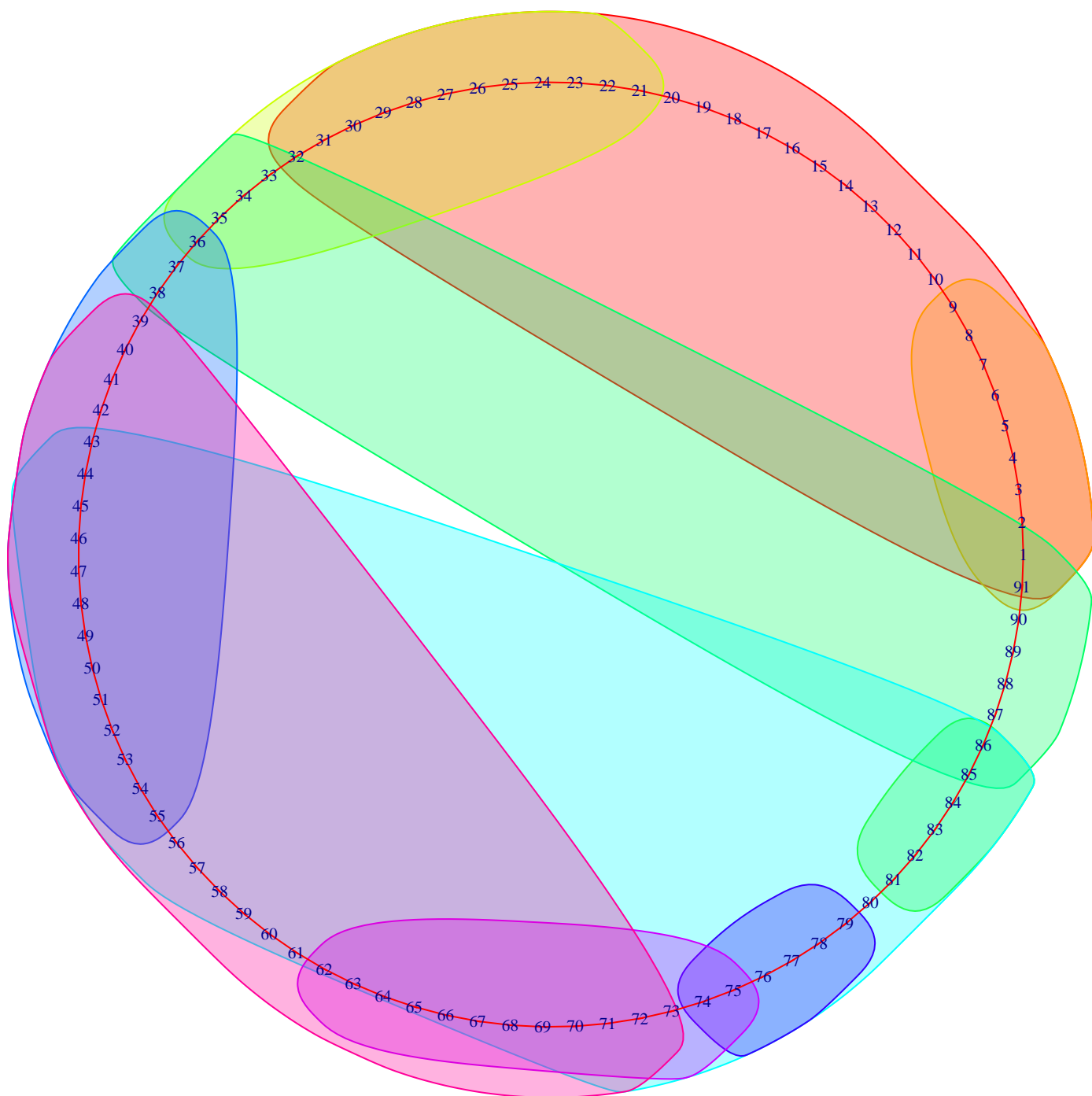

Supplement: Supplementary file 1 [file brainsci-09-00144-s001.zip › Supplementary 2/Mapper_graphs/104012_graph2B.pdf]

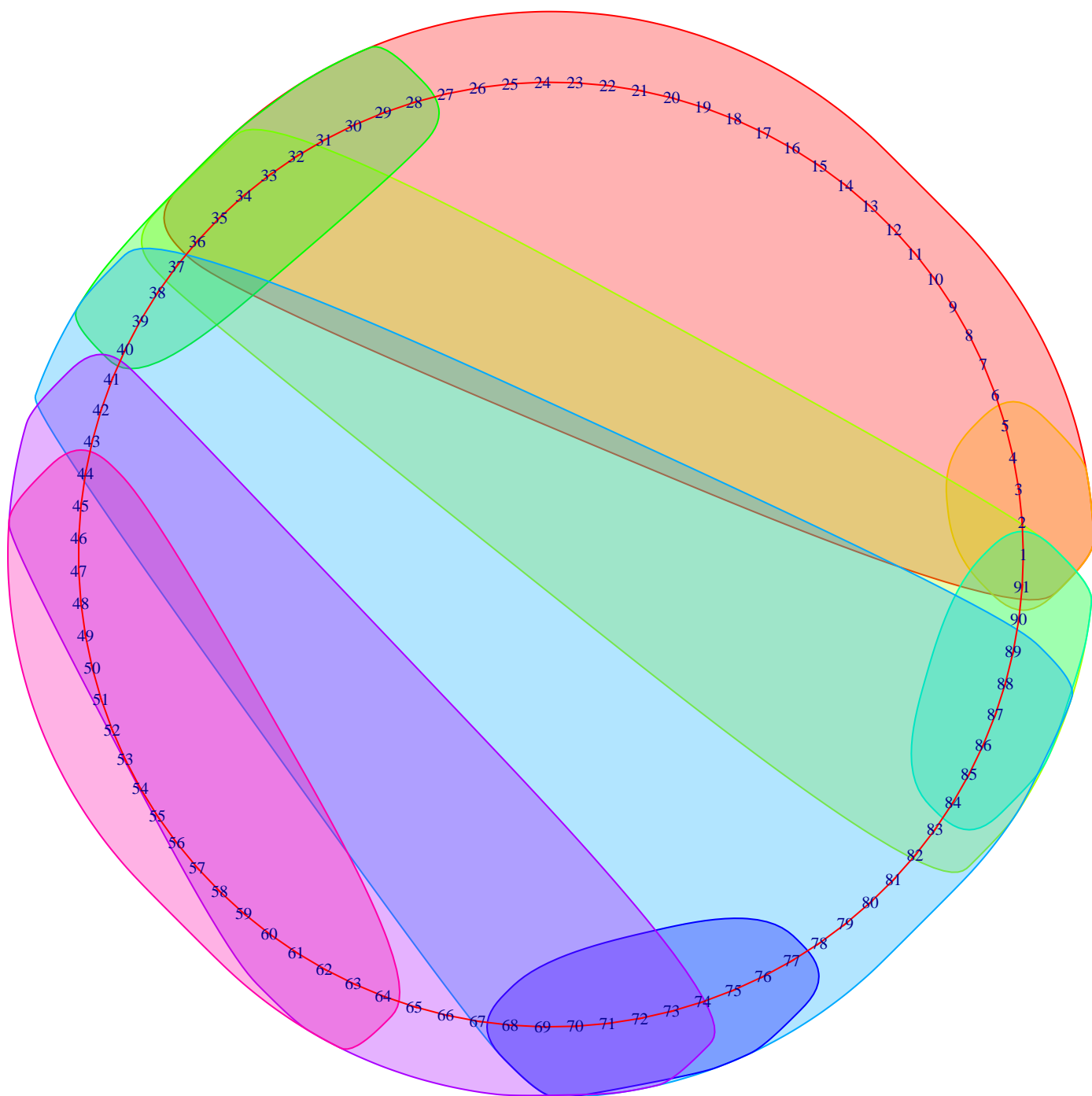

Supplement: Supplementary file 1 [file brainsci-09-00144-s001.zip › Supplementary 2/Mapper_graphs/195041_0B.pdf]

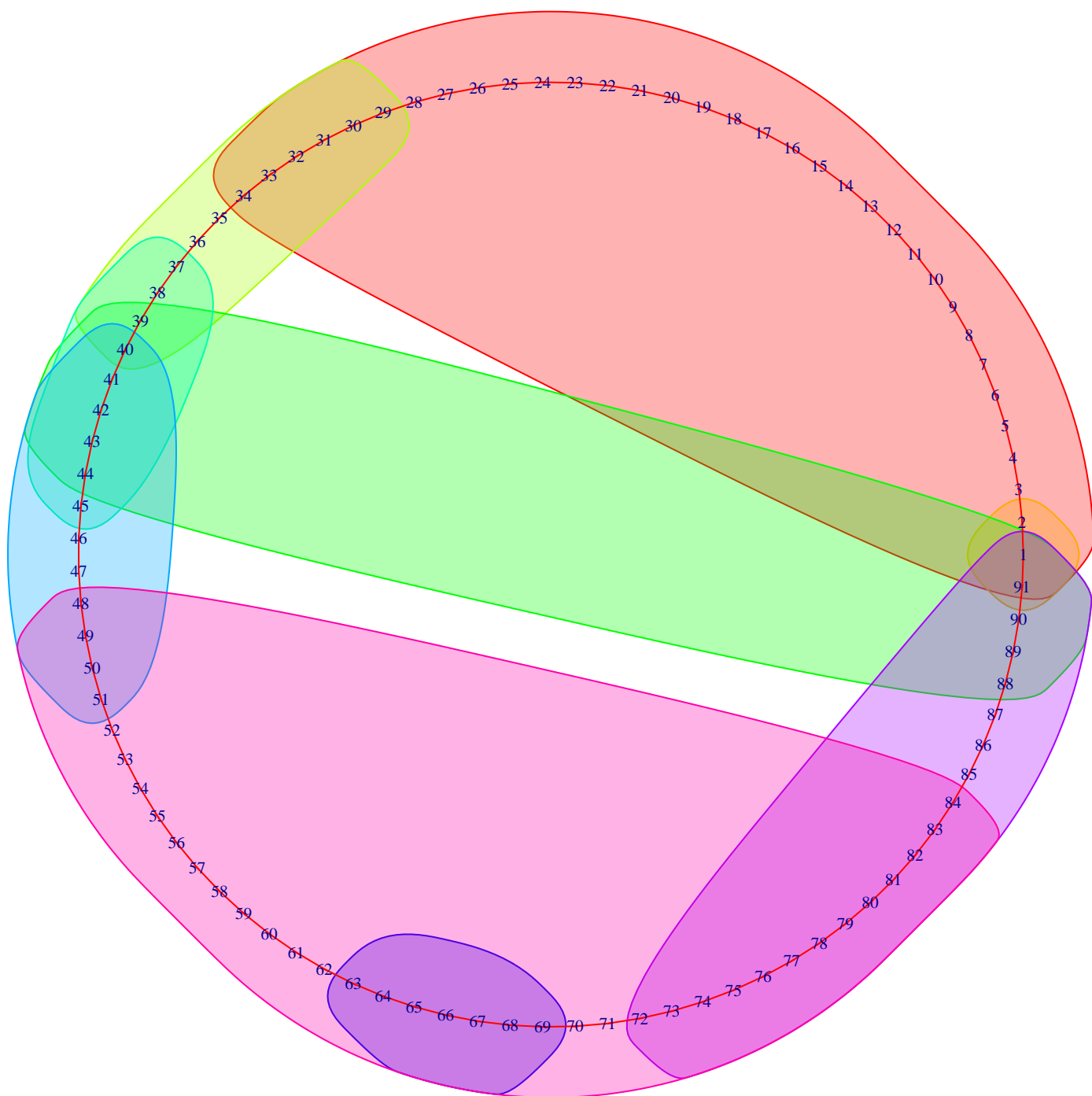

Supplement: Supplementary file 1 [file brainsci-09-00144-s001.zip › Supplementary 2/Mapper_graphs/191841_0B.pdf]

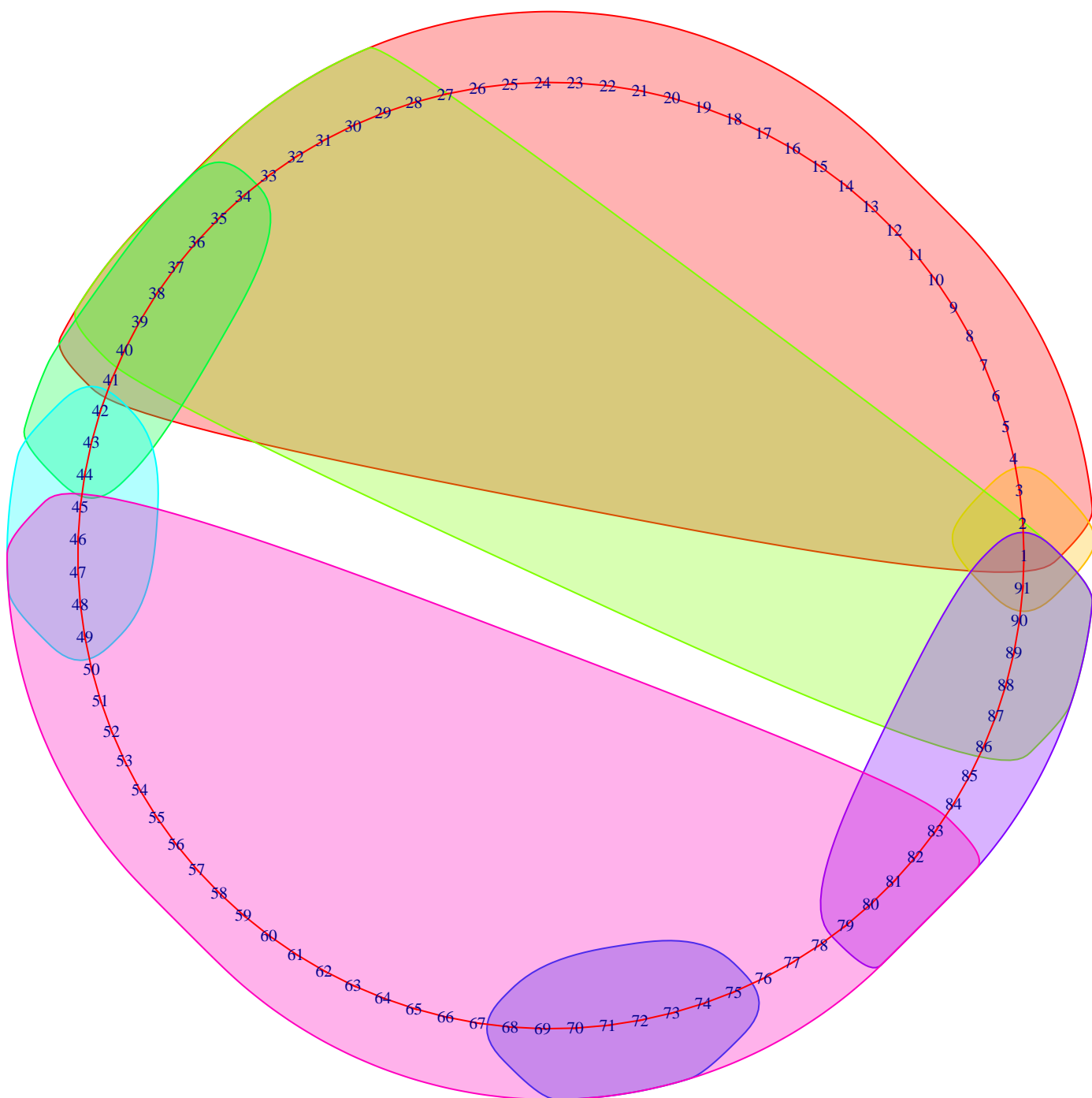

Supplement: Supplementary file 1 [file brainsci-09-00144-s001.zip › Supplementary 2/Mapper_graphs/105923_graph2B.pdf]

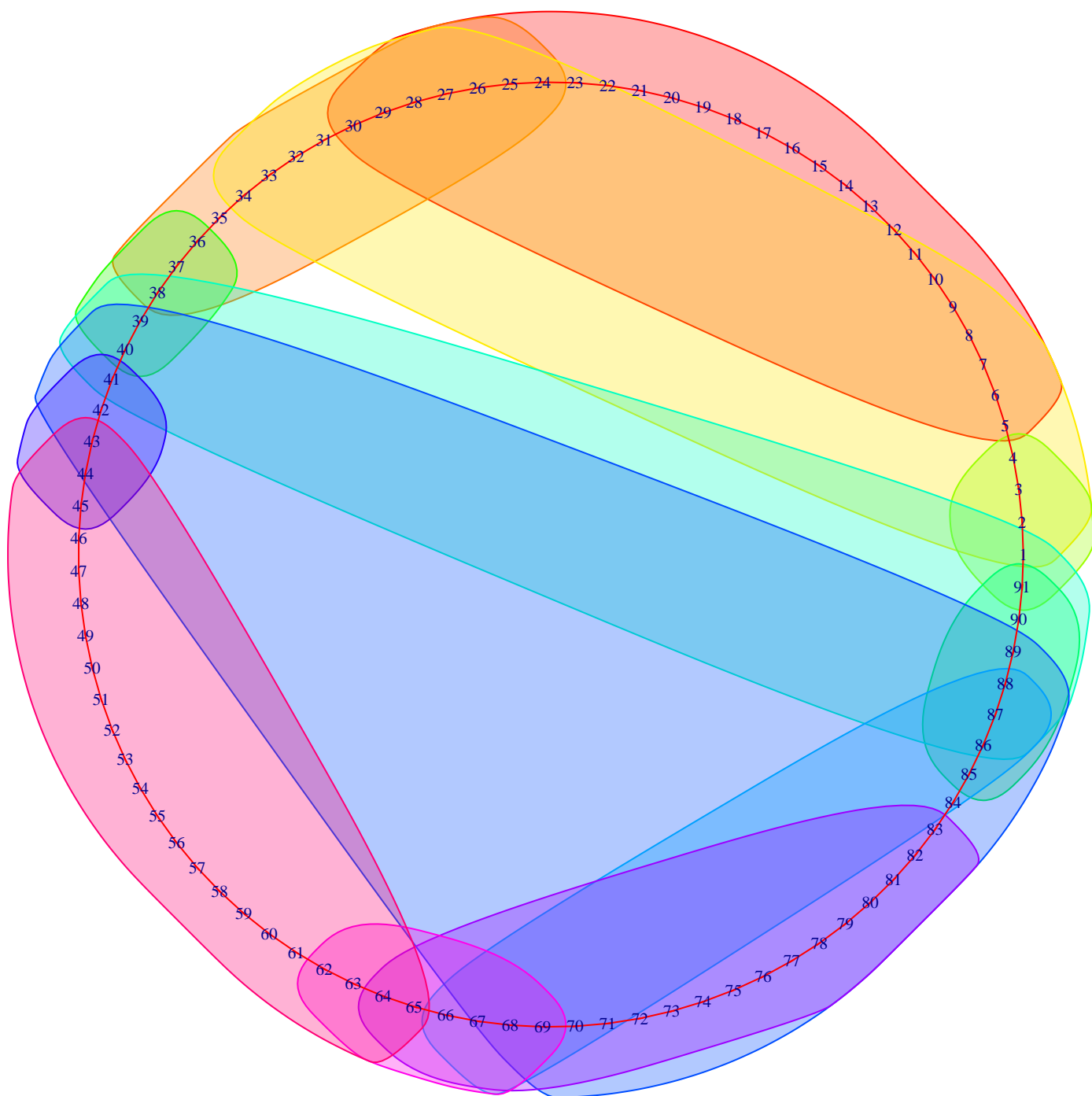

Supplement: Supplementary file 1 [file brainsci-09-00144-s001.zip › Supplementary 2/Mapper_graphs/182840_2B.pdf]

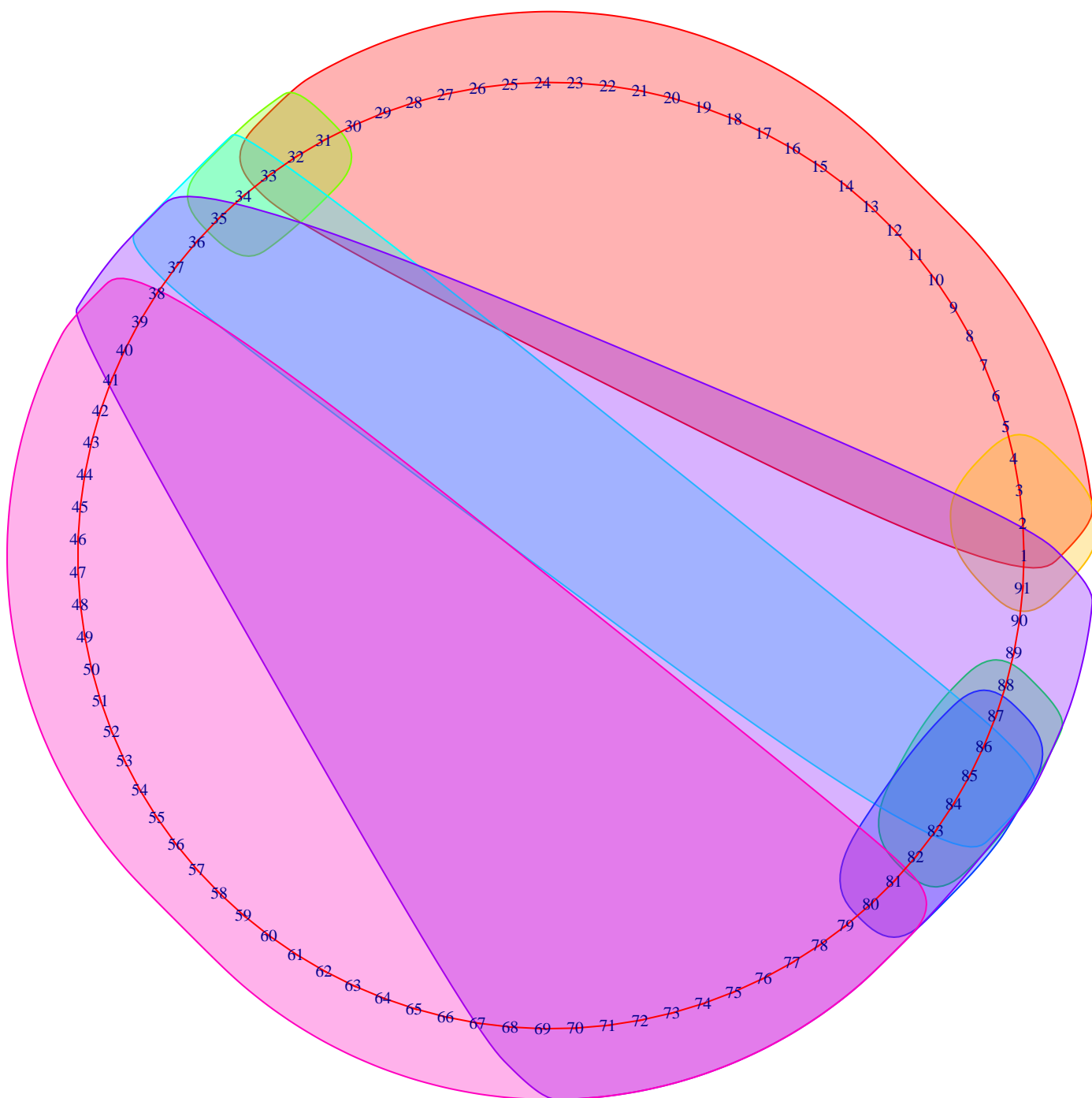

Supplement: Supplementary file 1 [file brainsci-09-00144-s001.zip › Supplementary 2/Mapper_graphs/877168_2B.pdf]

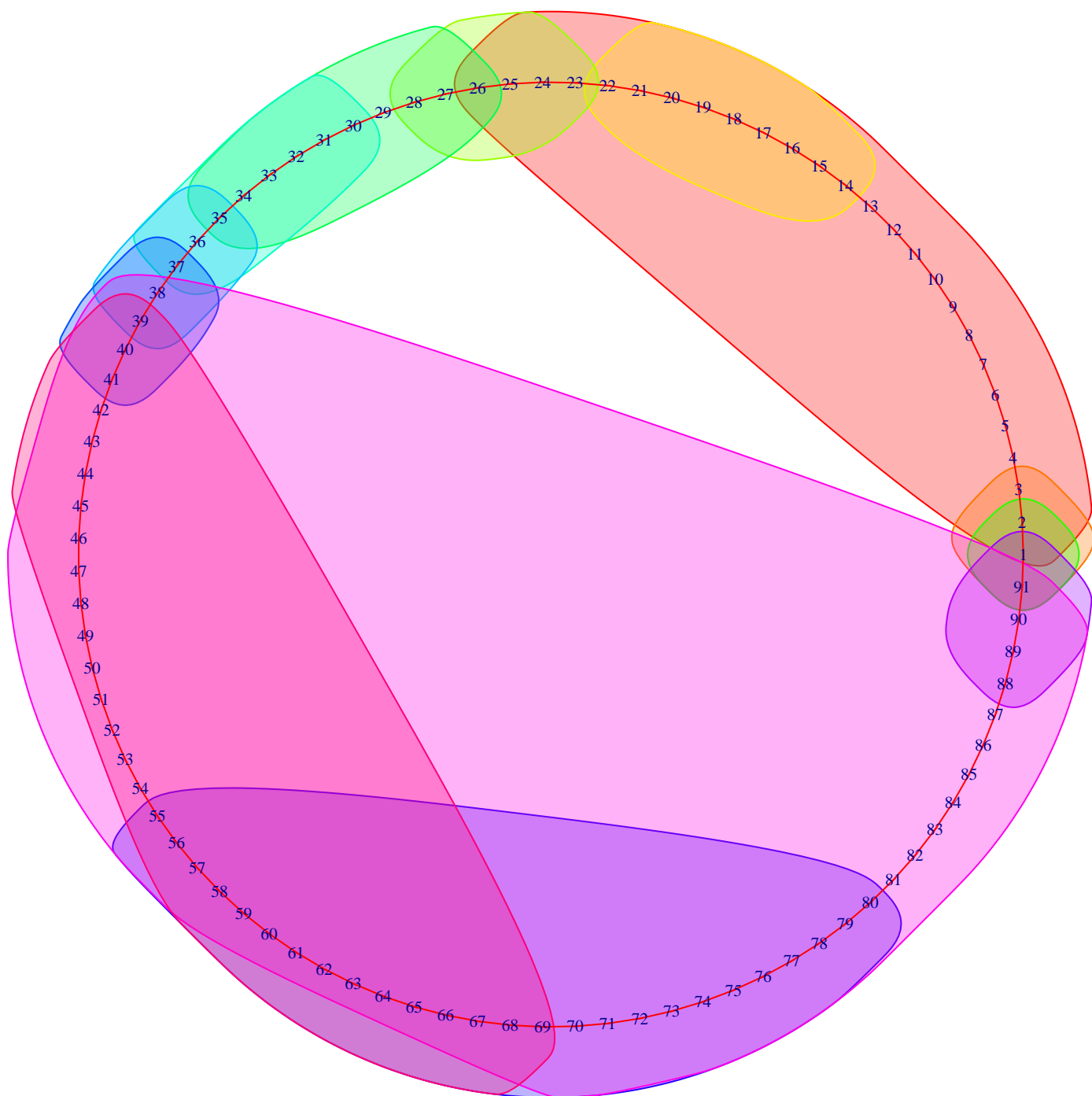

Supplement: Supplementary file 1 [file brainsci-09-00144-s001.zip › Supplementary 2/Mapper_graphs/113922_graph0B.pdf]

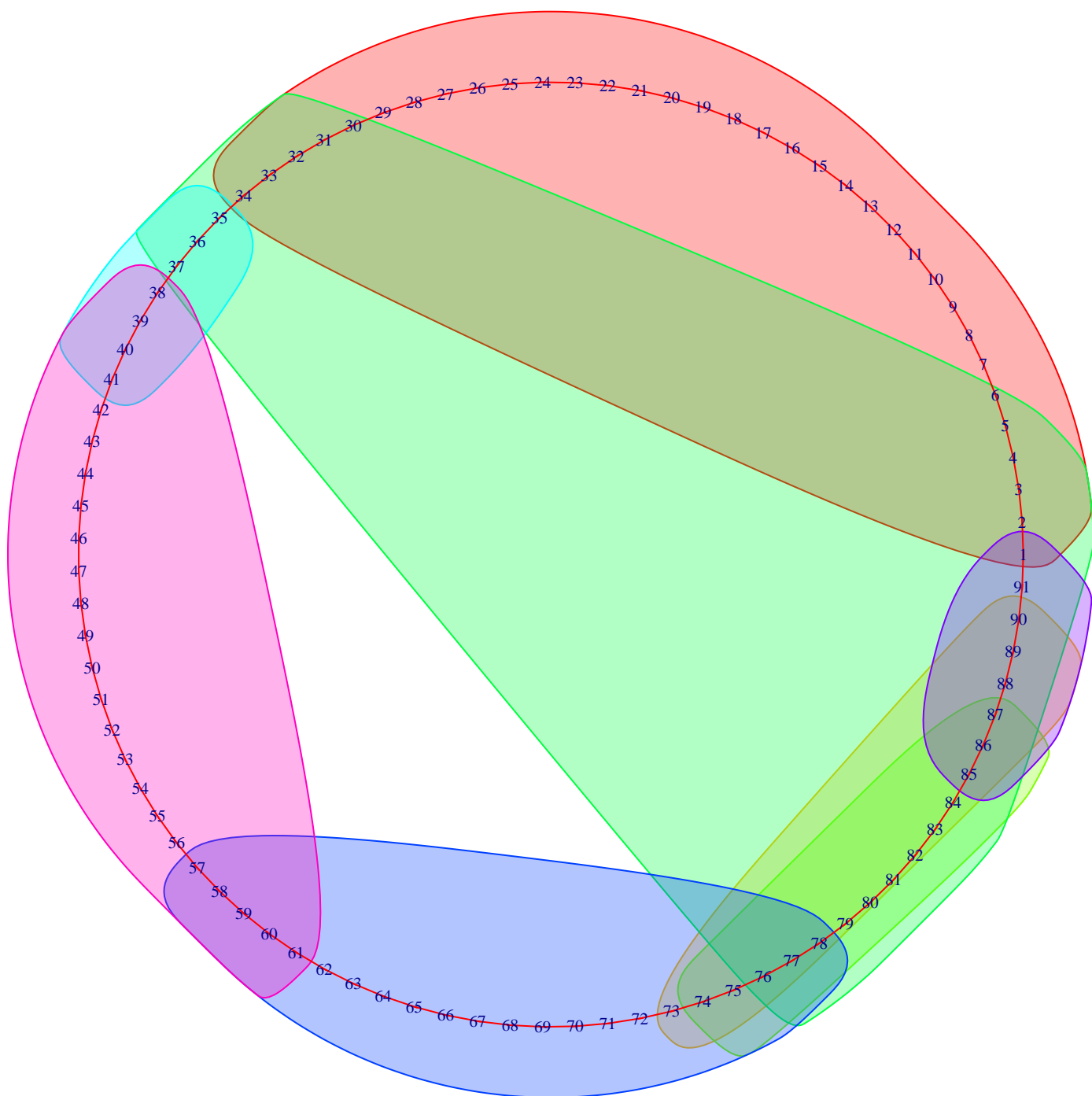

Supplement: Supplementary file 1 [file brainsci-09-00144-s001.zip › Supplementary 2/Mapper_graphs/248339_0B.pdf]

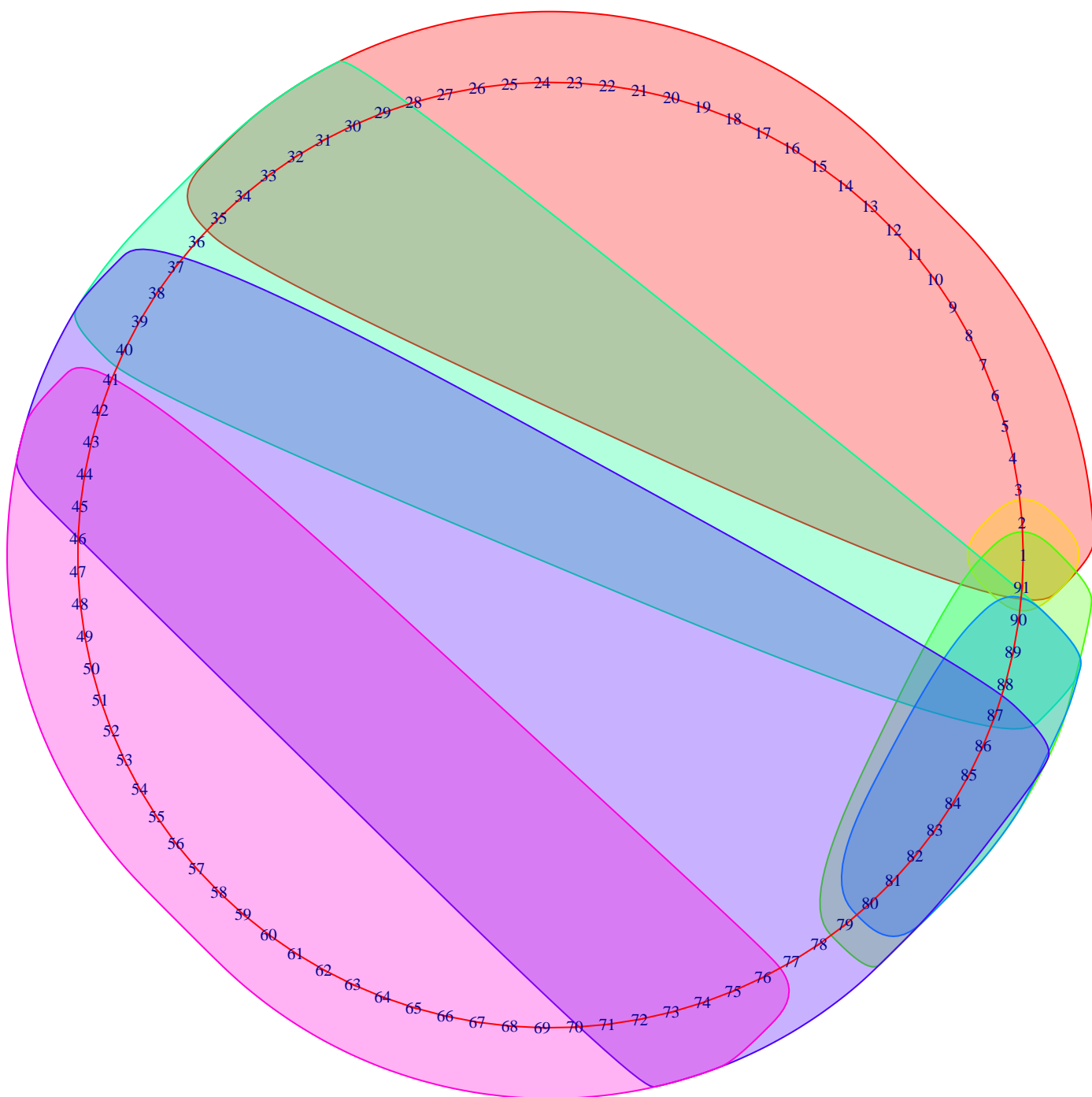

Supplement: Supplementary file 1 [file brainsci-09-00144-s001.zip › Supplementary 2/Mapper_graphs/109123_graph0B.pdf]

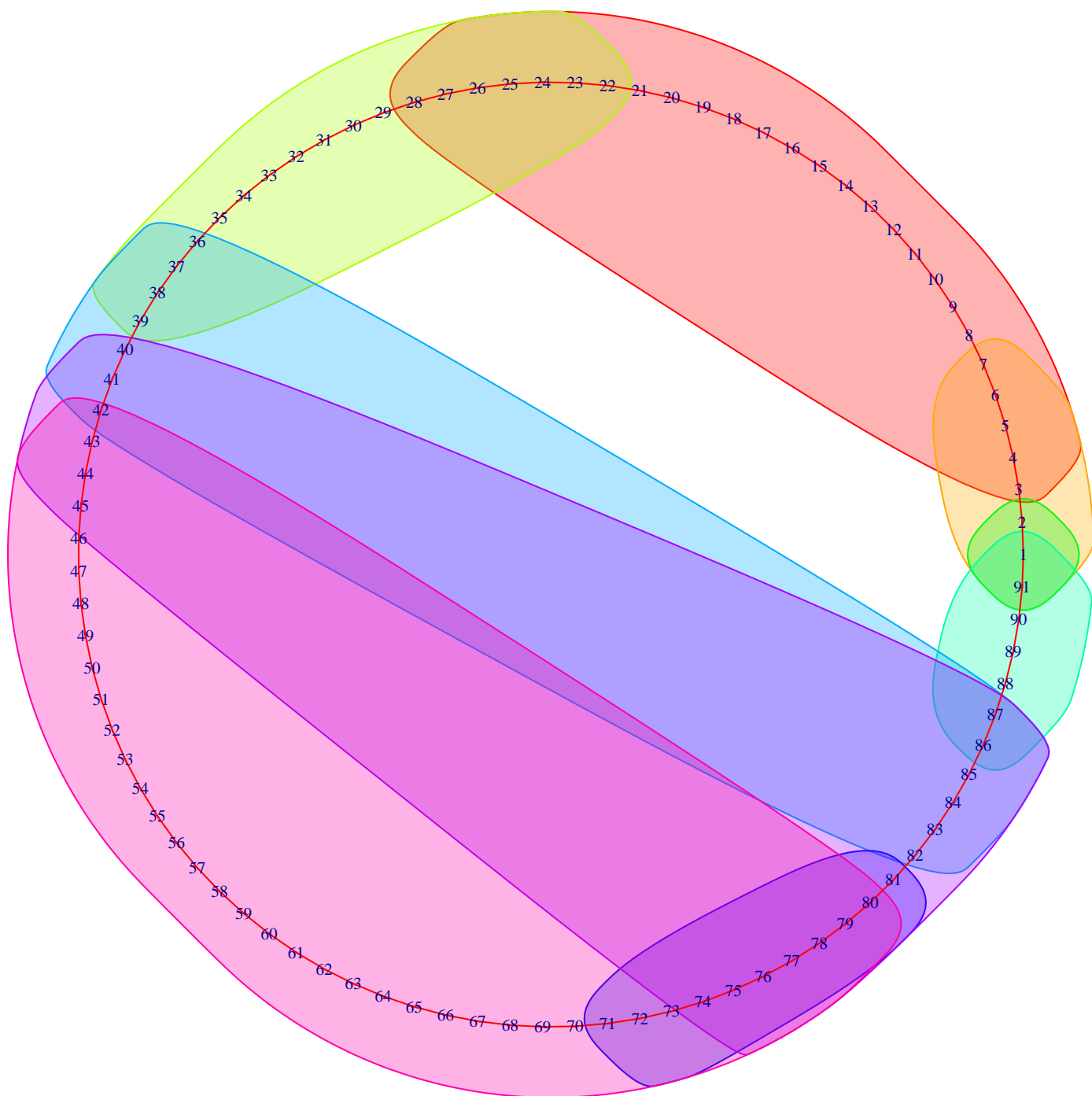

Supplement: Supplementary file 1 [file brainsci-09-00144-s001.zip › Supplementary 2/Mapper_graphs/581450_2B.pdf]

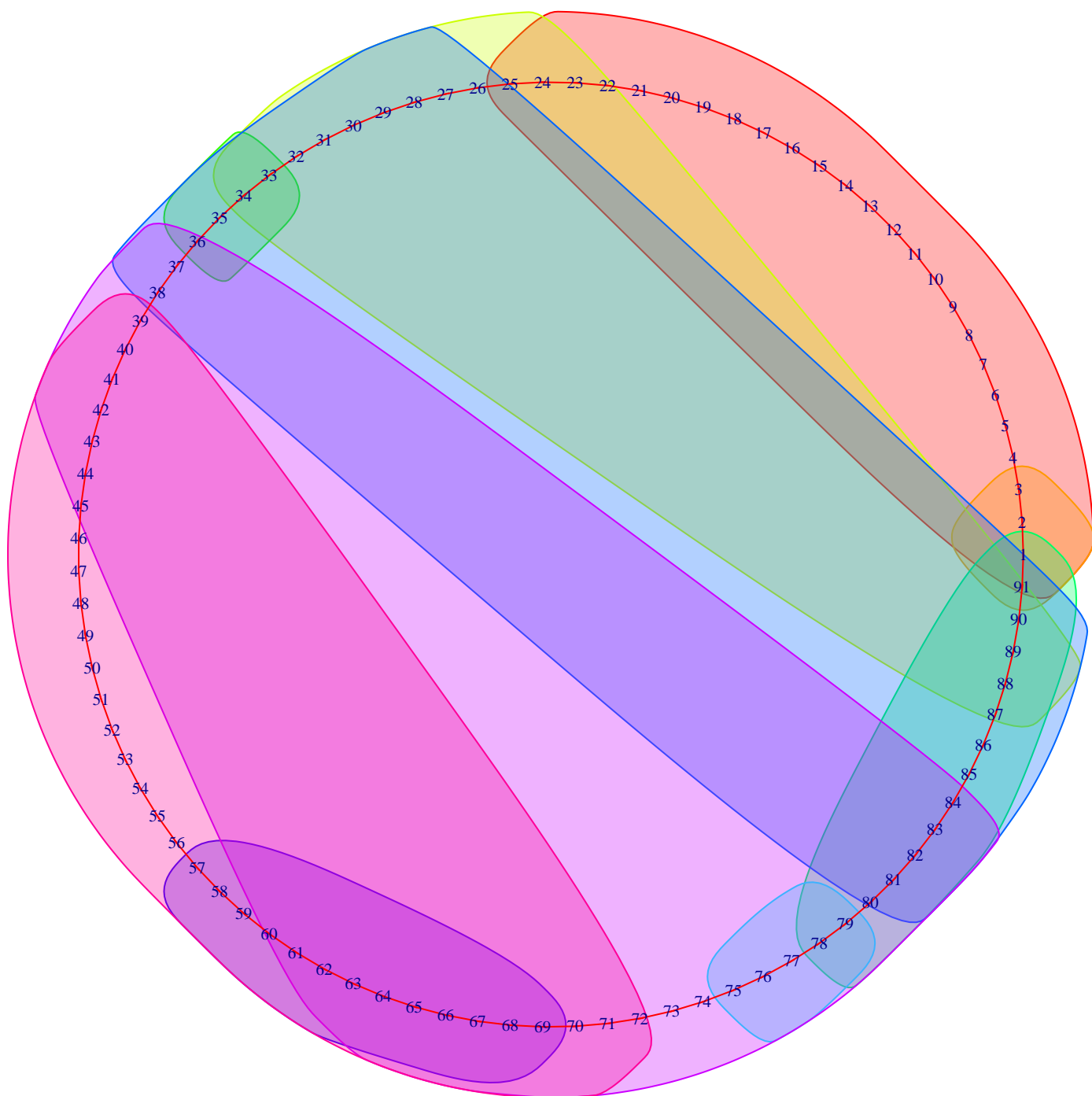

Supplement: Supplementary file 1 [file brainsci-09-00144-s001.zip › Supplementary 2/Mapper_graphs/162026_graph2B.pdf]

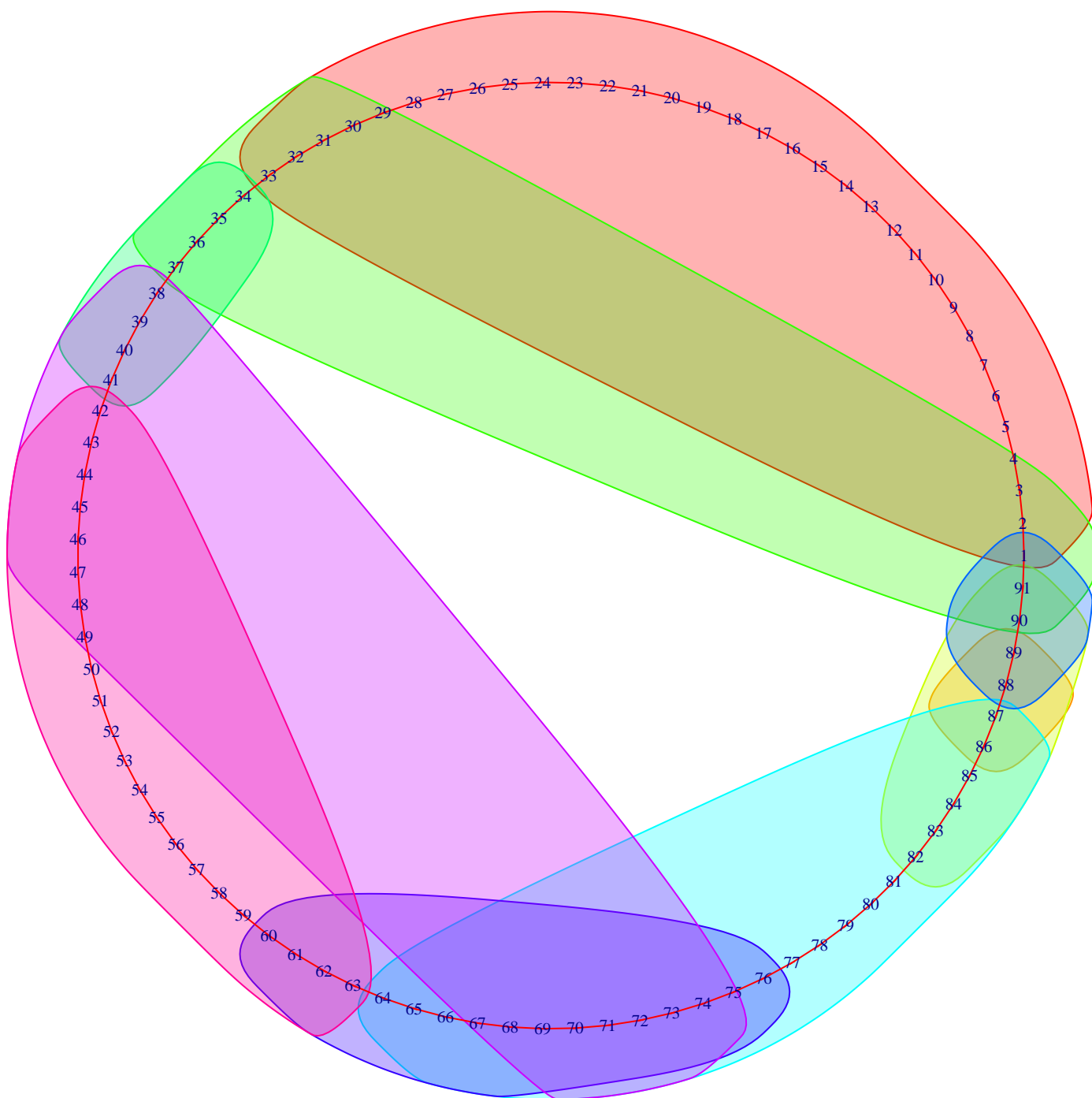

Supplement: Supplementary file 1 [file brainsci-09-00144-s001.zip › Supplementary 2/Mapper_graphs/223929_2B.pdf]

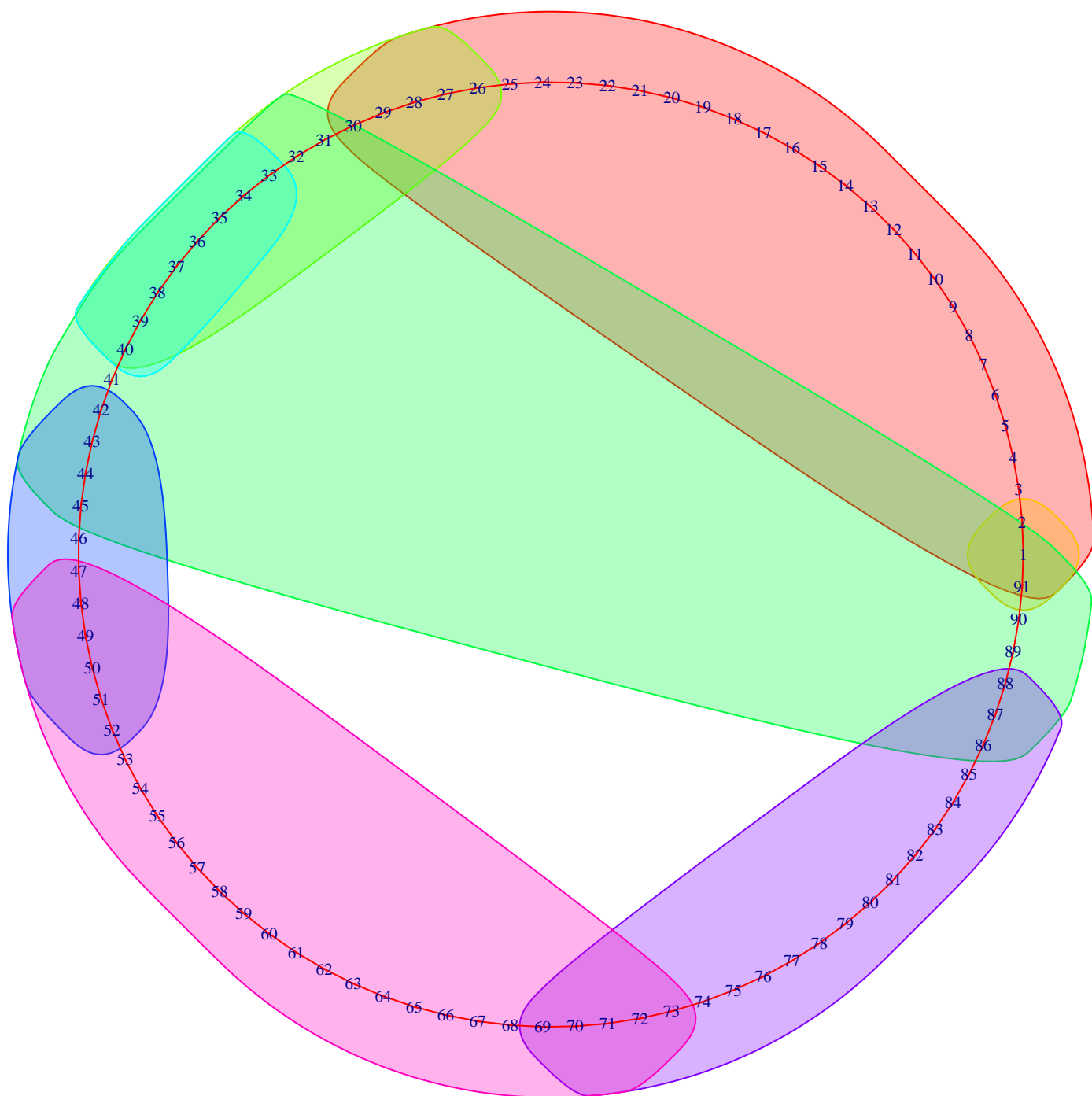

Supplement: Supplementary file 1 [file brainsci-09-00144-s001.zip › Supplementary 2/Mapper_graphs/151526_graph2B.pdf]

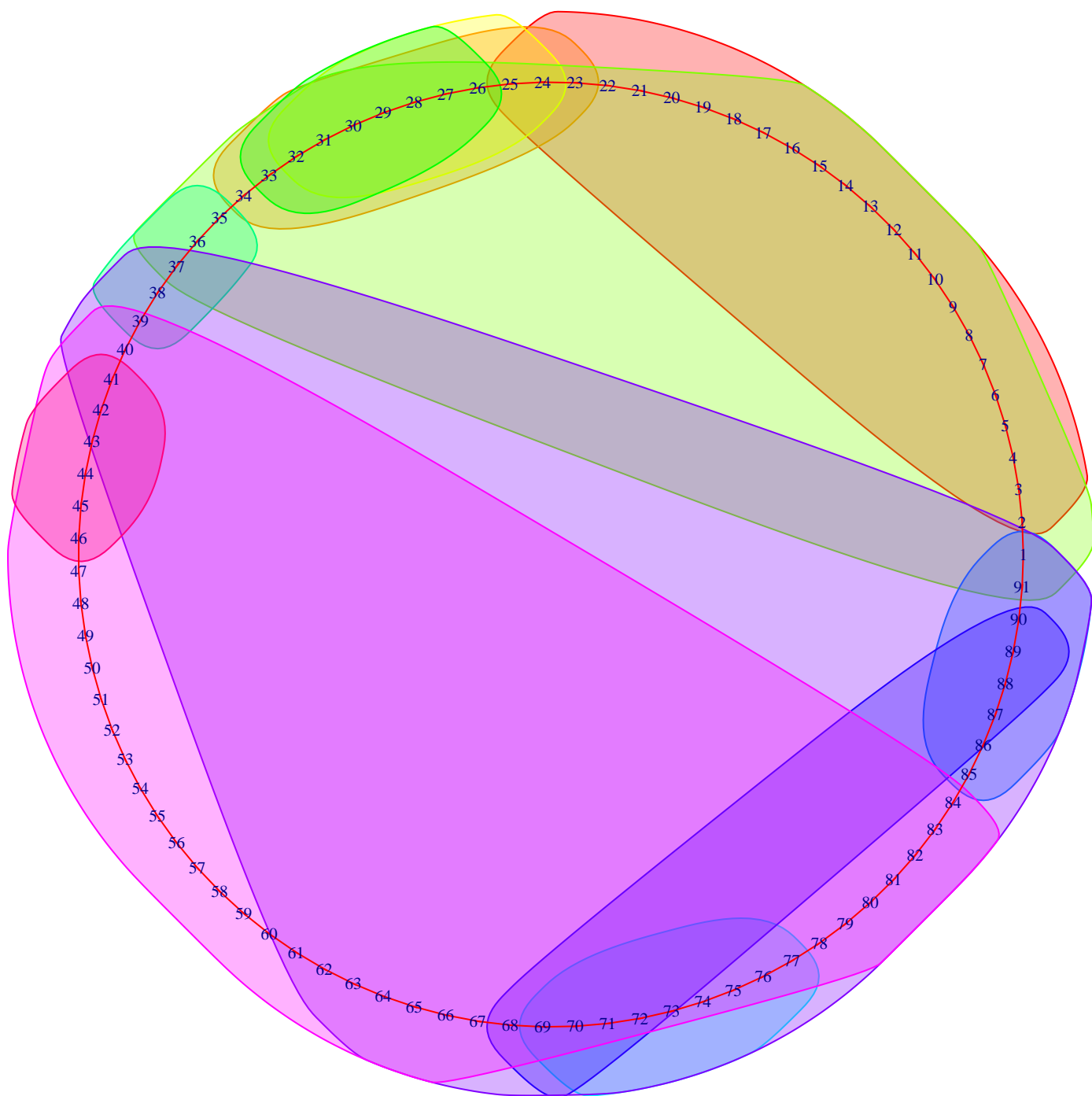

Supplement: Supplementary file 1 [file brainsci-09-00144-s001.zip › Supplementary 2/Mapper_graphs/568963_0B.pdf]

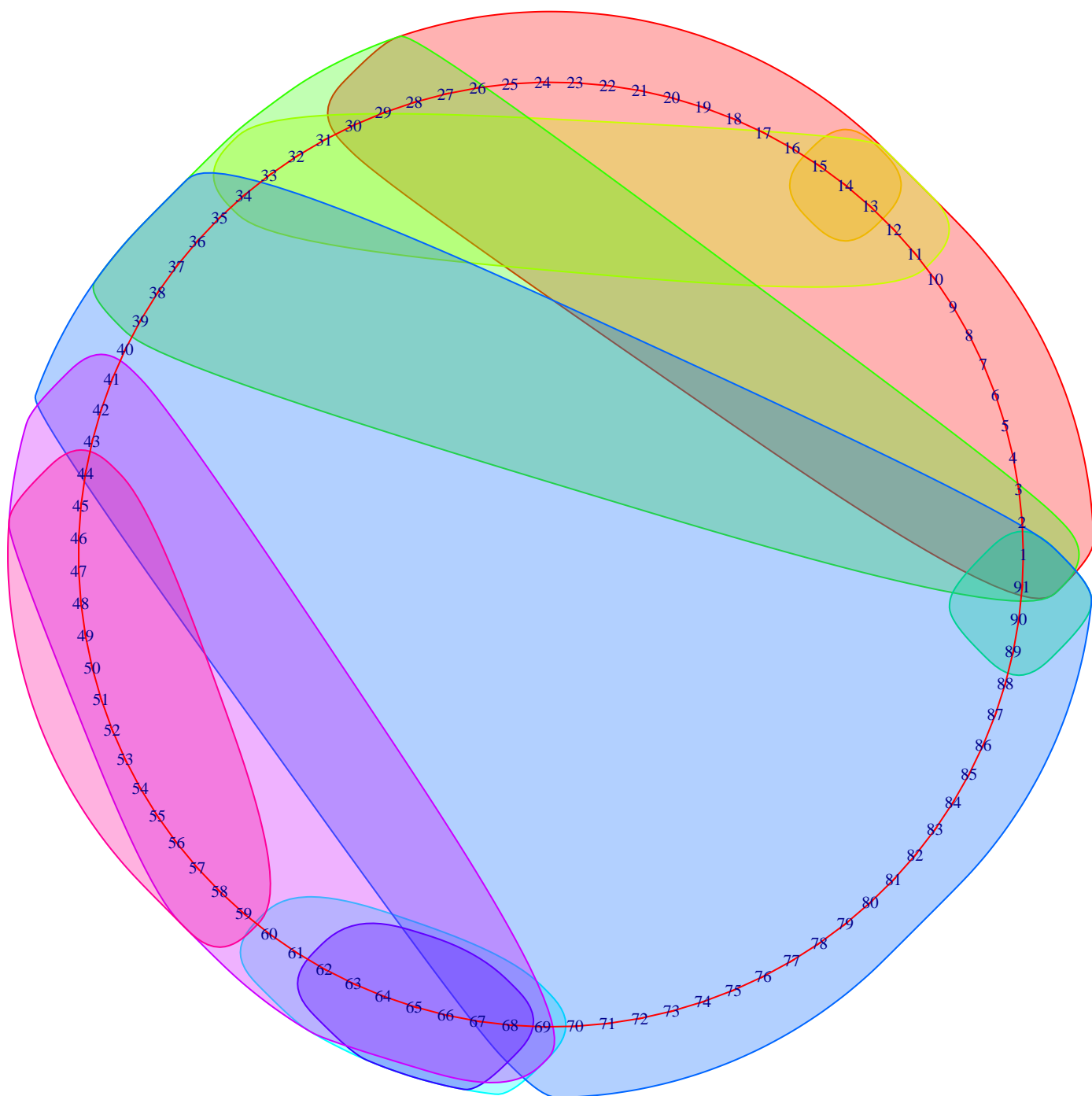

Supplement: Supplementary file 1 [file brainsci-09-00144-s001.zip › Supplementary 2/Mapper_graphs/164636_graph0B.pdf]

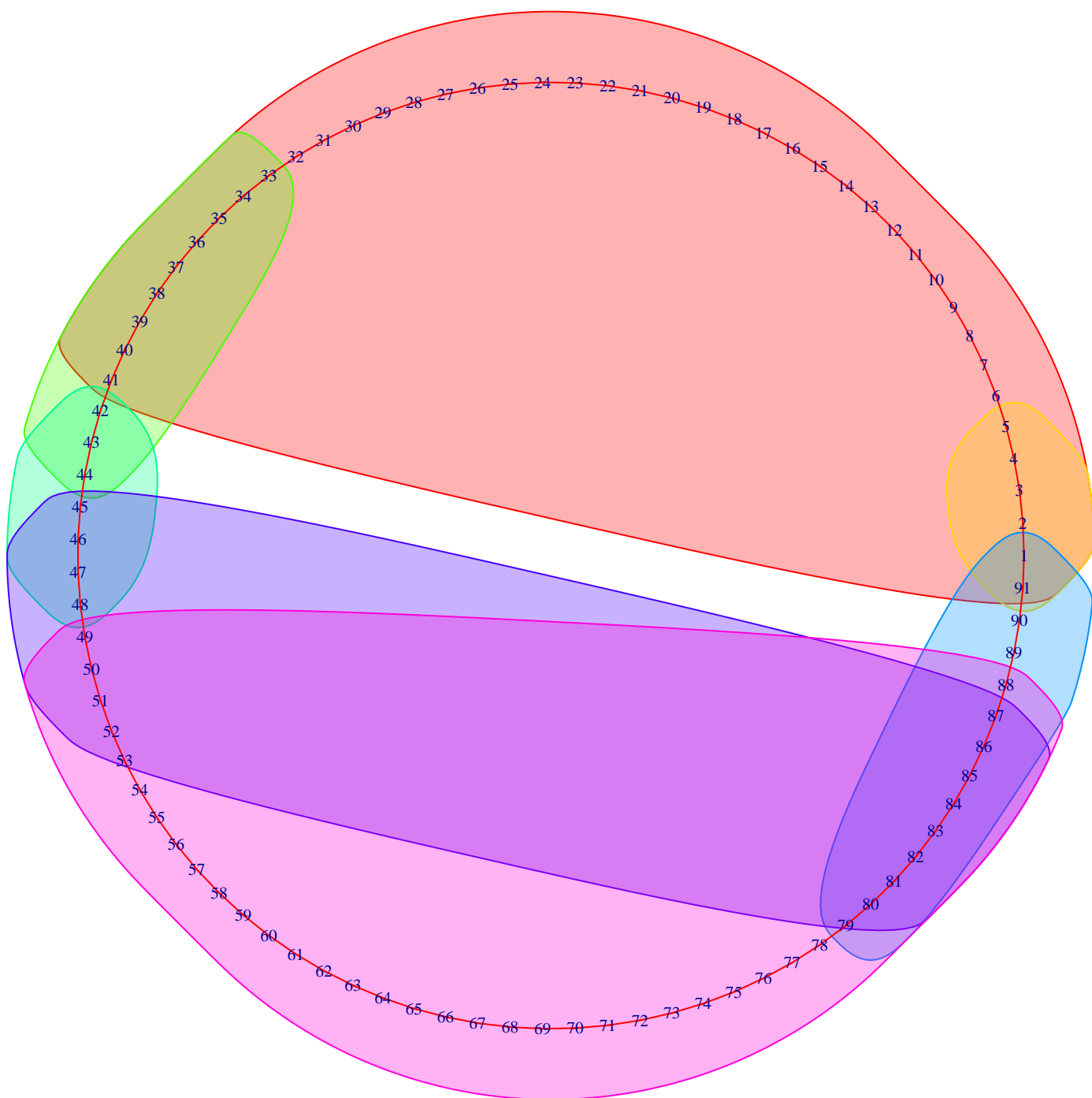

Supplement: Supplementary file 1 [file brainsci-09-00144-s001.zip › Supplementary 2/Mapper_graphs/125525_graph2B.pdf]

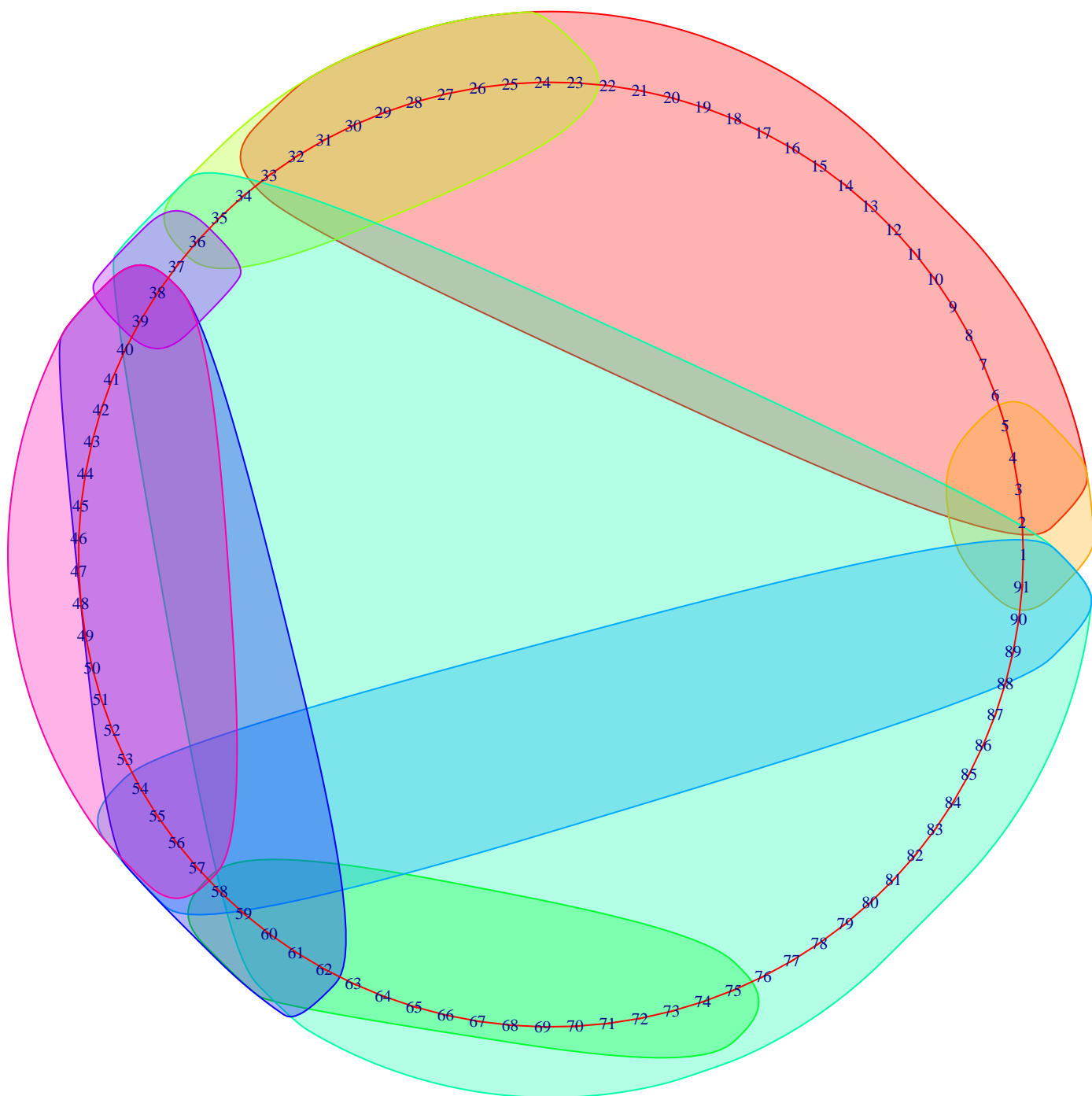

Supplement: Supplementary file 1 [file brainsci-09-00144-s001.zip › Supplementary 2/Mapper_graphs/189349_0B.pdf]

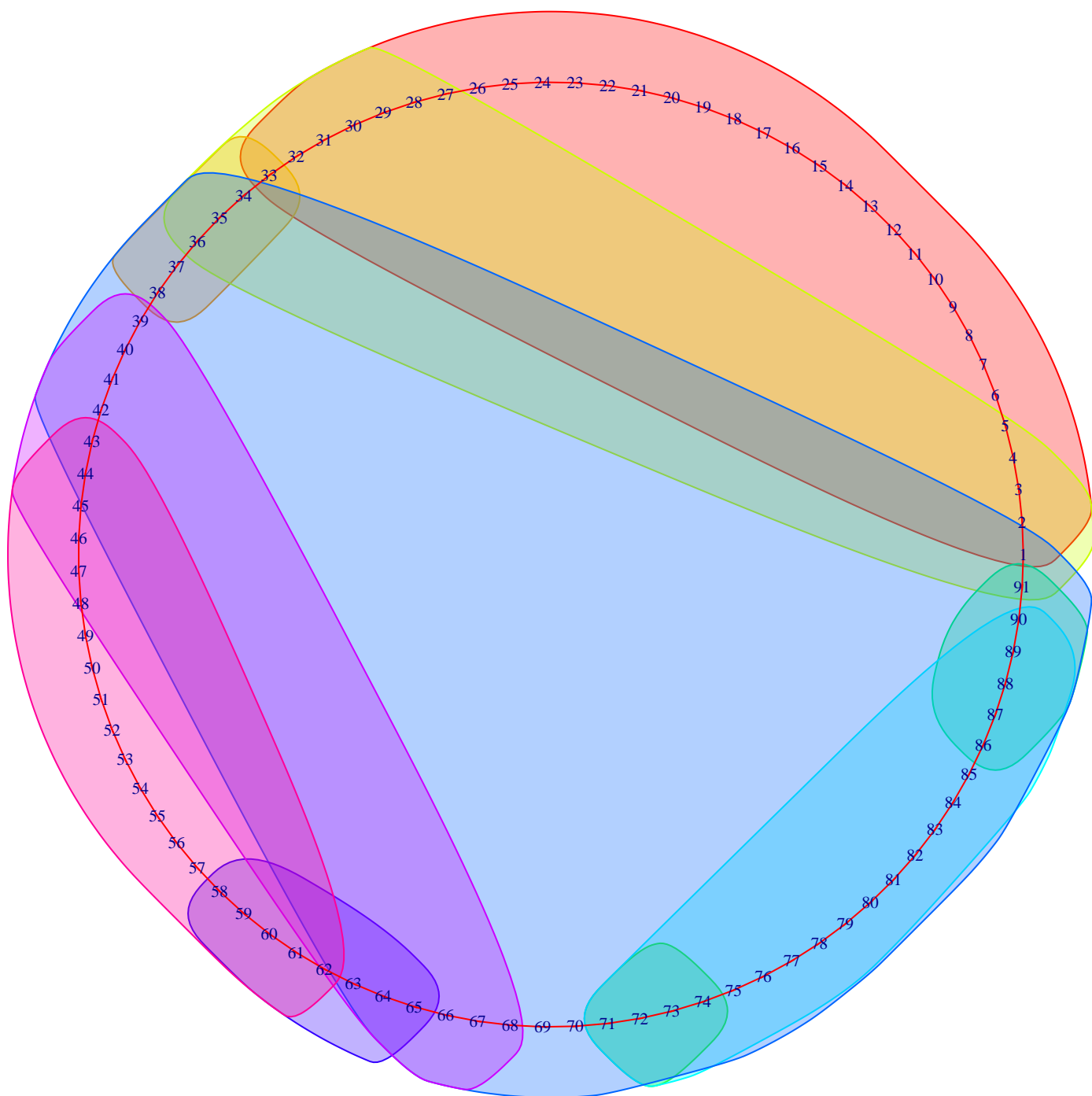

Supplement: Supplementary file 1 [file brainsci-09-00144-s001.zip › Supplementary 2/Mapper_graphs/825048_0B.pdf]

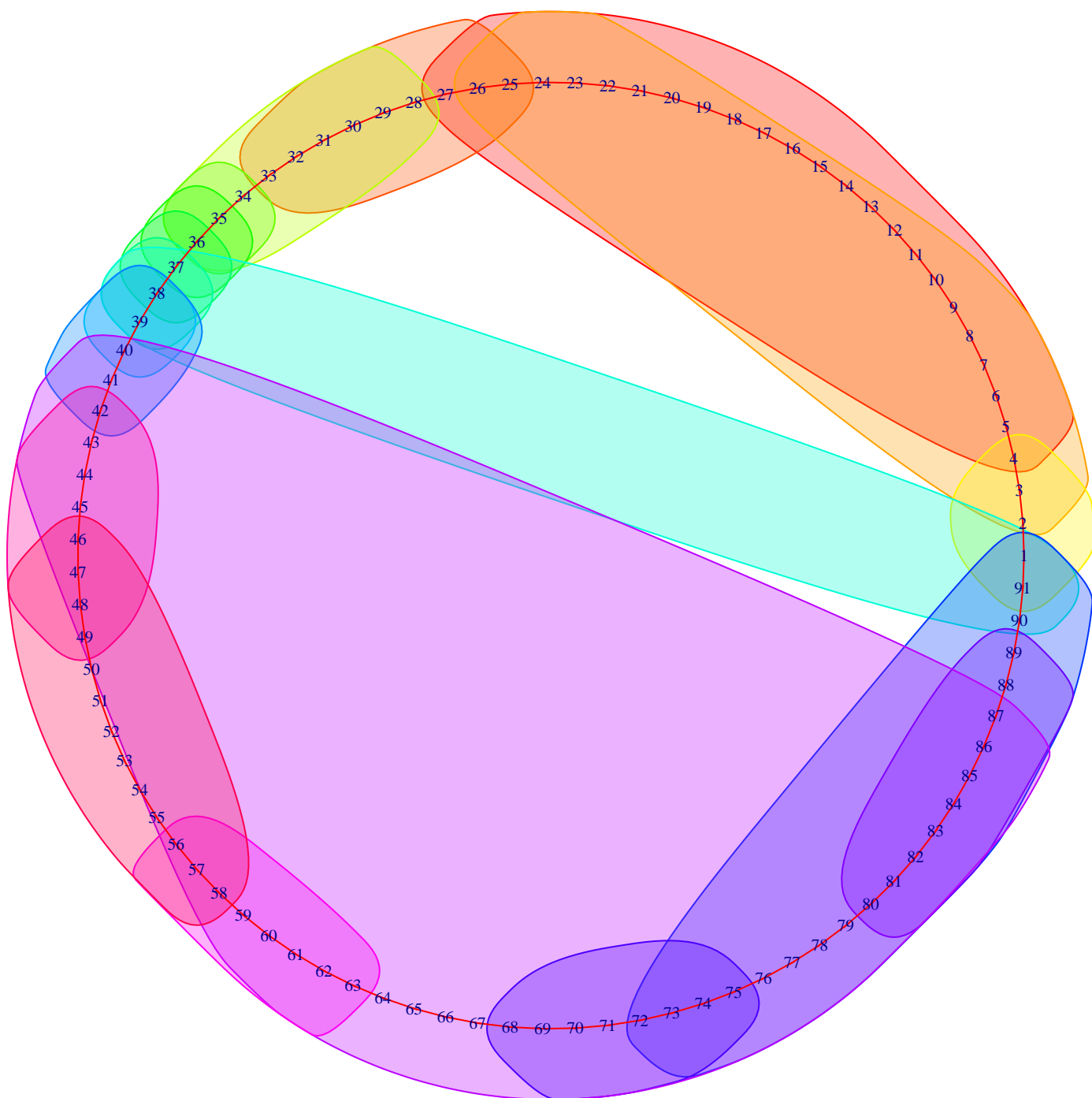

Supplement: Supplementary file 1 [file brainsci-09-00144-s001.zip › Supplementary 2/Mapper_graphs/162935_graph2B.pdf]

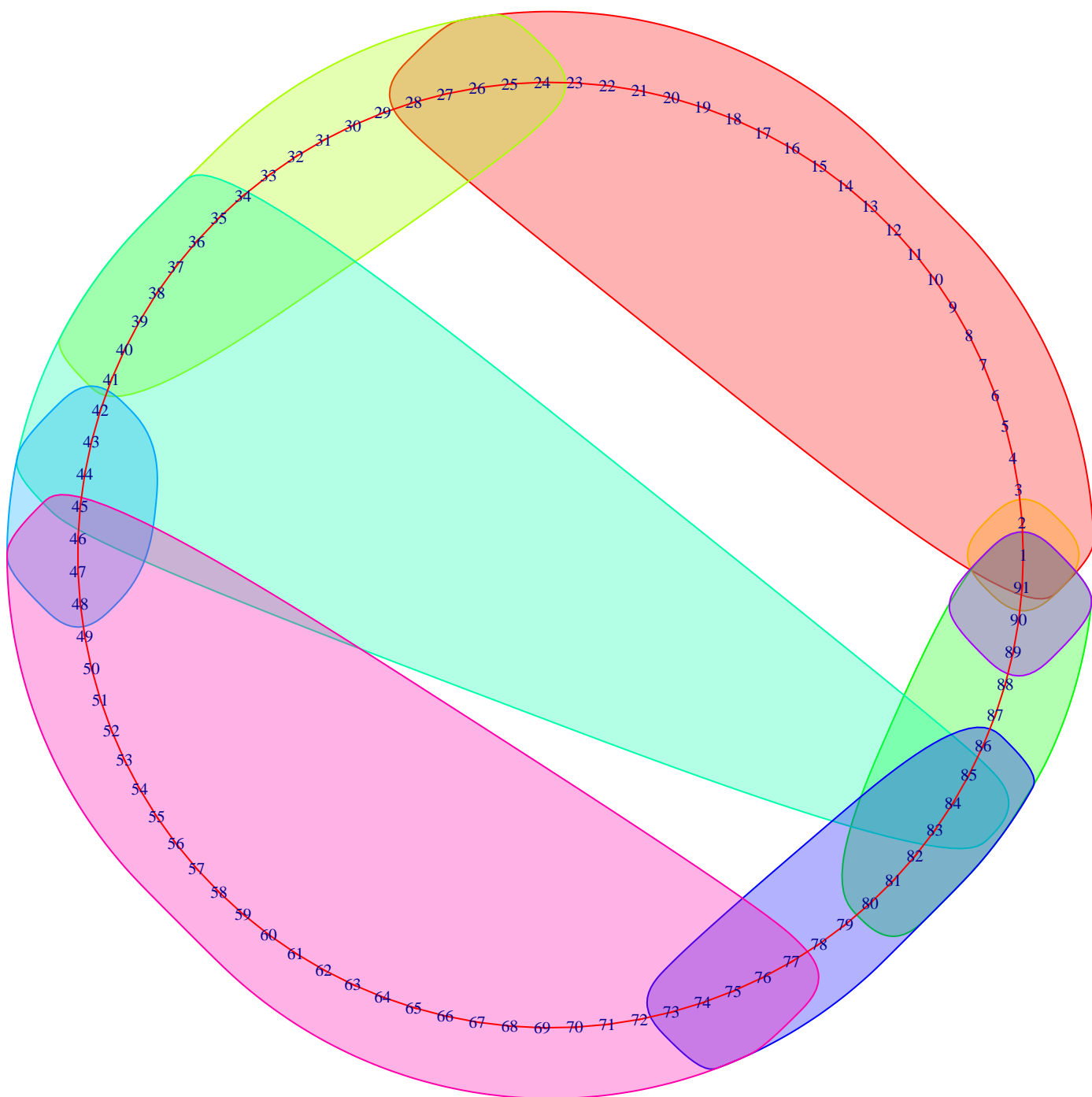

Supplement: Supplementary file 1 [file brainsci-09-00144-s001.zip › Supplementary 2/Mapper_graphs/662551_0B.pdf]

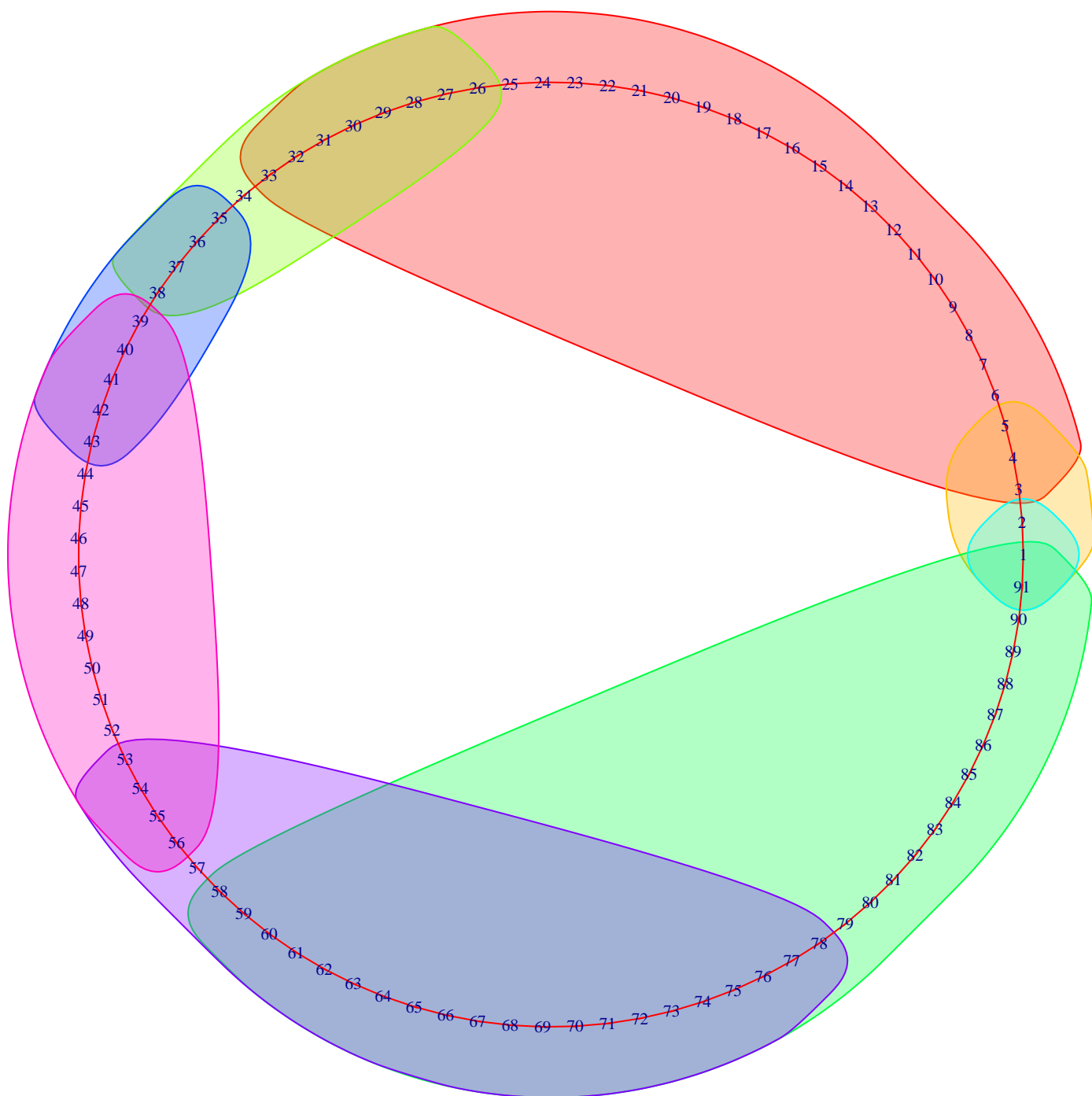

Supplement: Supplementary file 1 [file brainsci-09-00144-s001.zip › Supplementary 2/Mapper_graphs/601127_0B.pdf]

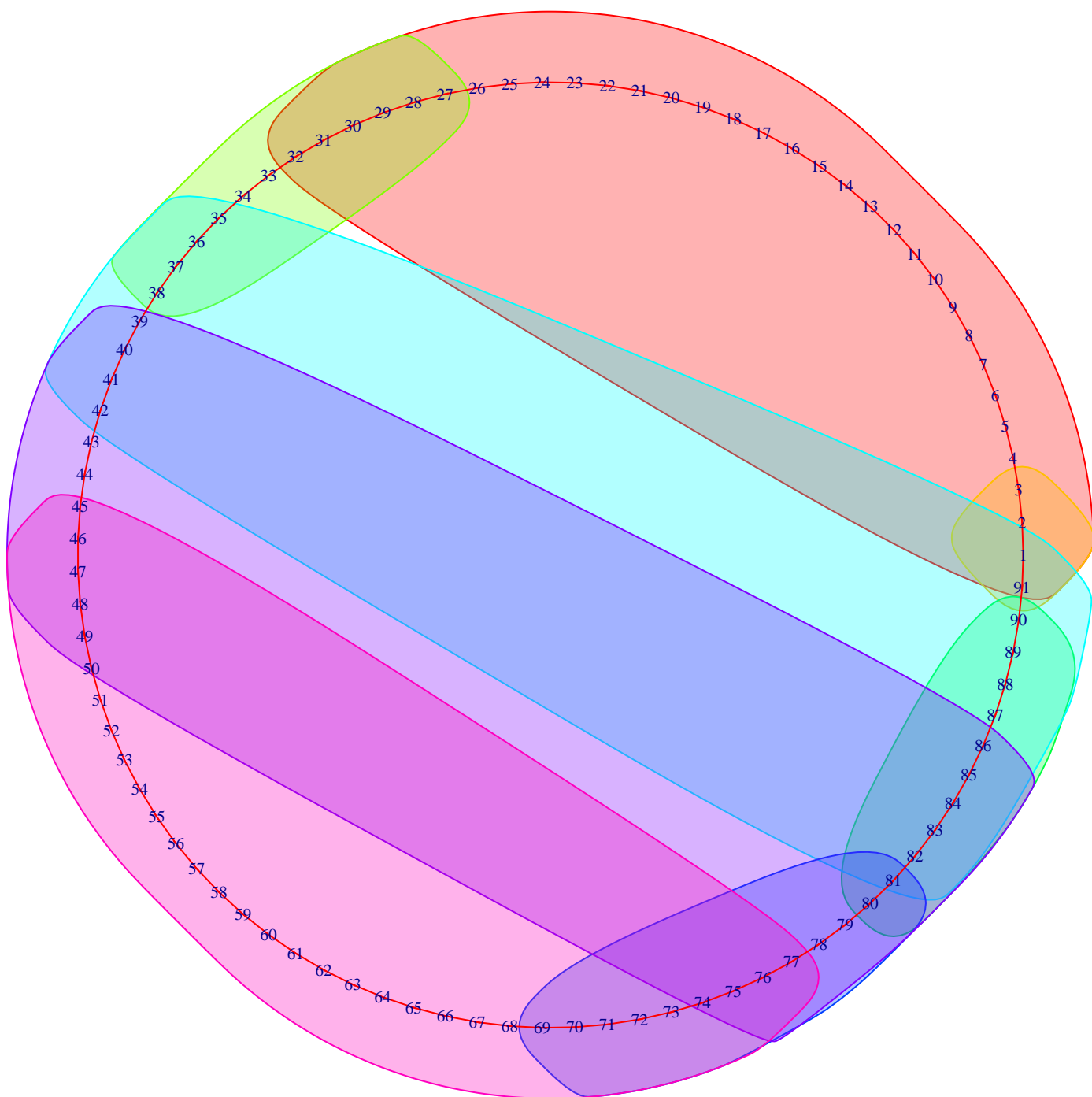

Supplement: Supplementary file 1 [file brainsci-09-00144-s001.zip › Supplementary 2/Mapper_graphs/177746_graph2B.pdf]

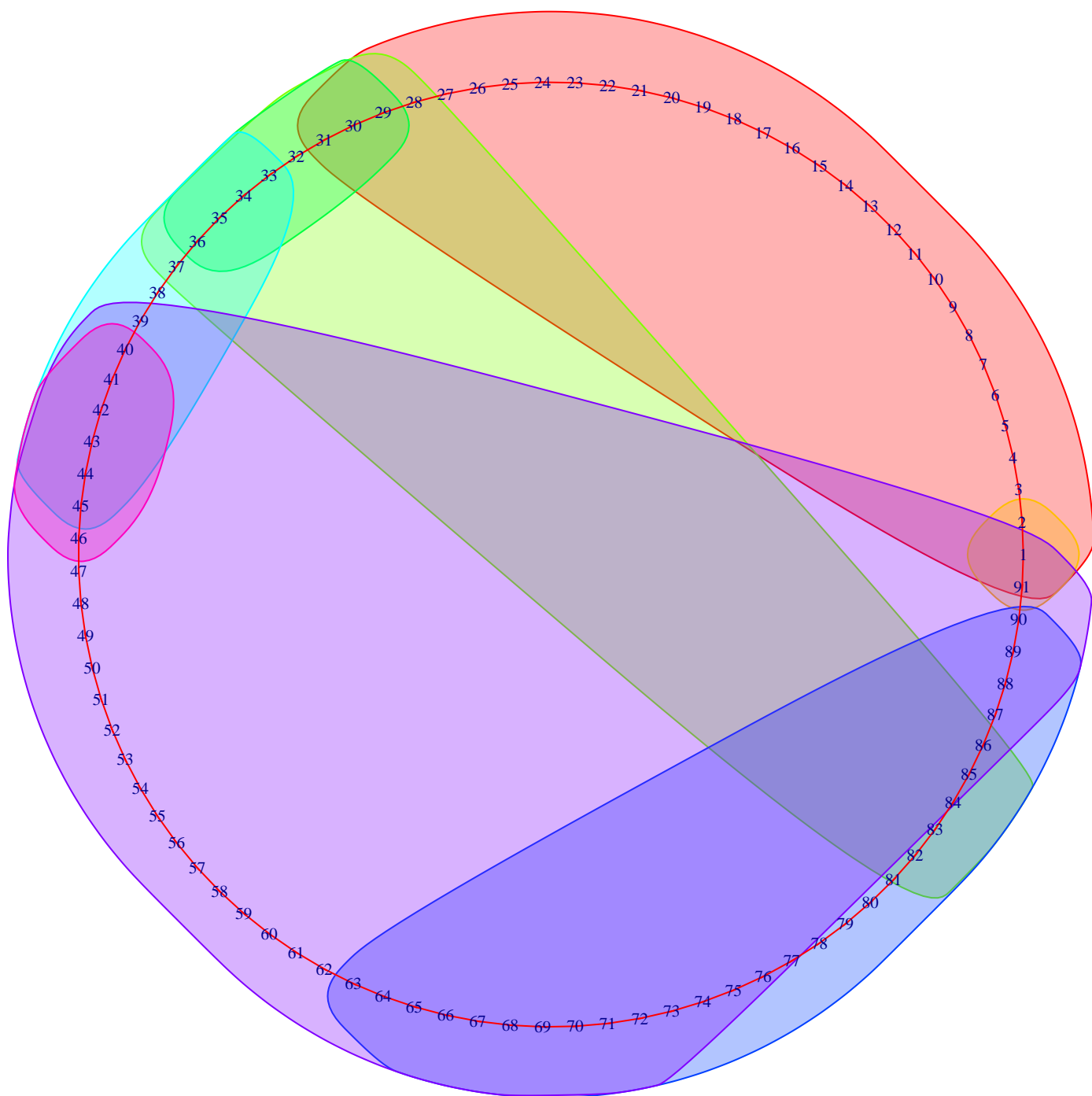

Supplement: Supplementary file 1 [file brainsci-09-00144-s001.zip › Supplementary 2/Mapper_graphs/917255_2B.pdf]

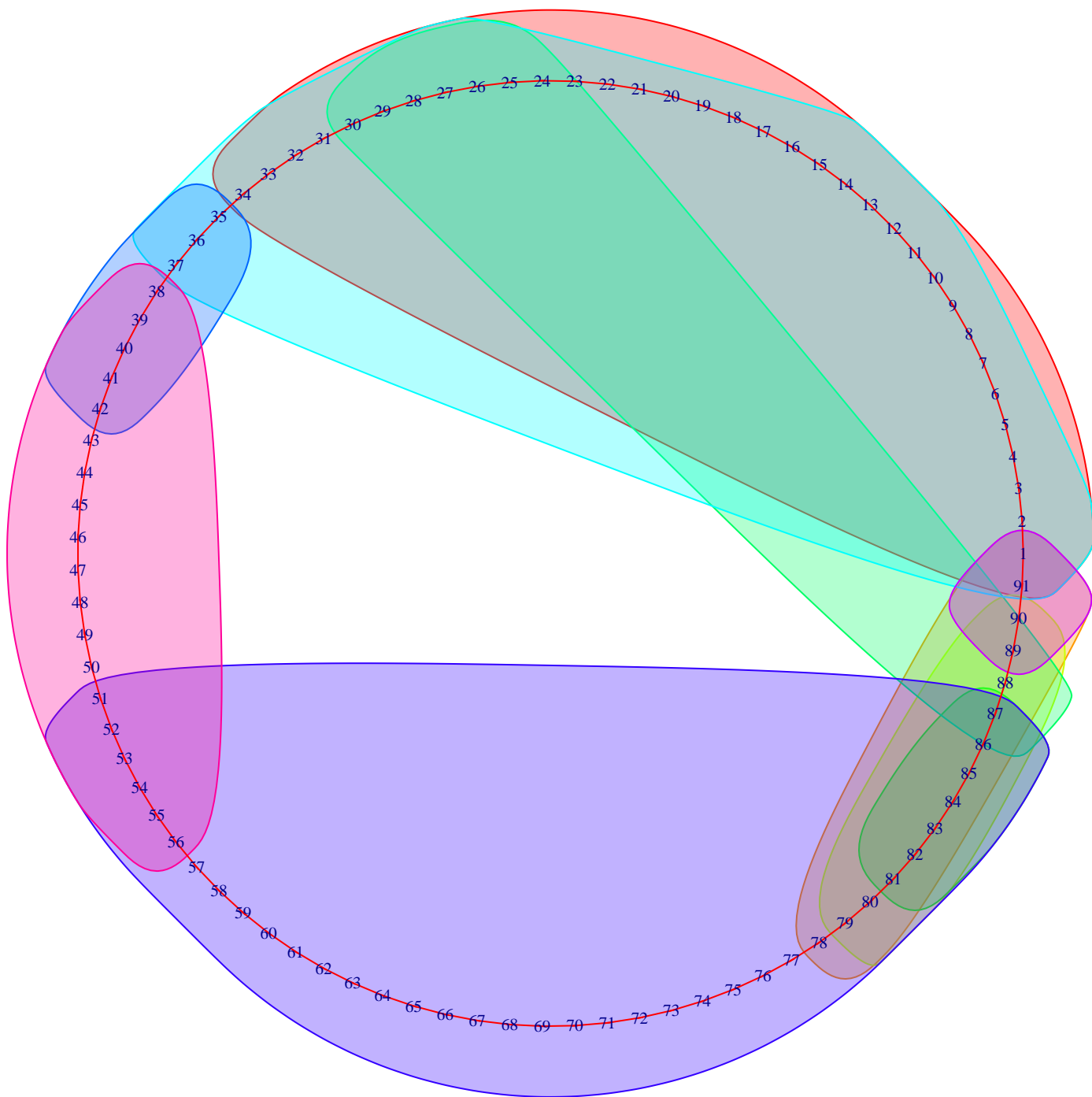

Supplement: Supplementary file 1 [file brainsci-09-00144-s001.zip › Supplementary 2/Mapper_graphs/106521_graph2B.pdf]

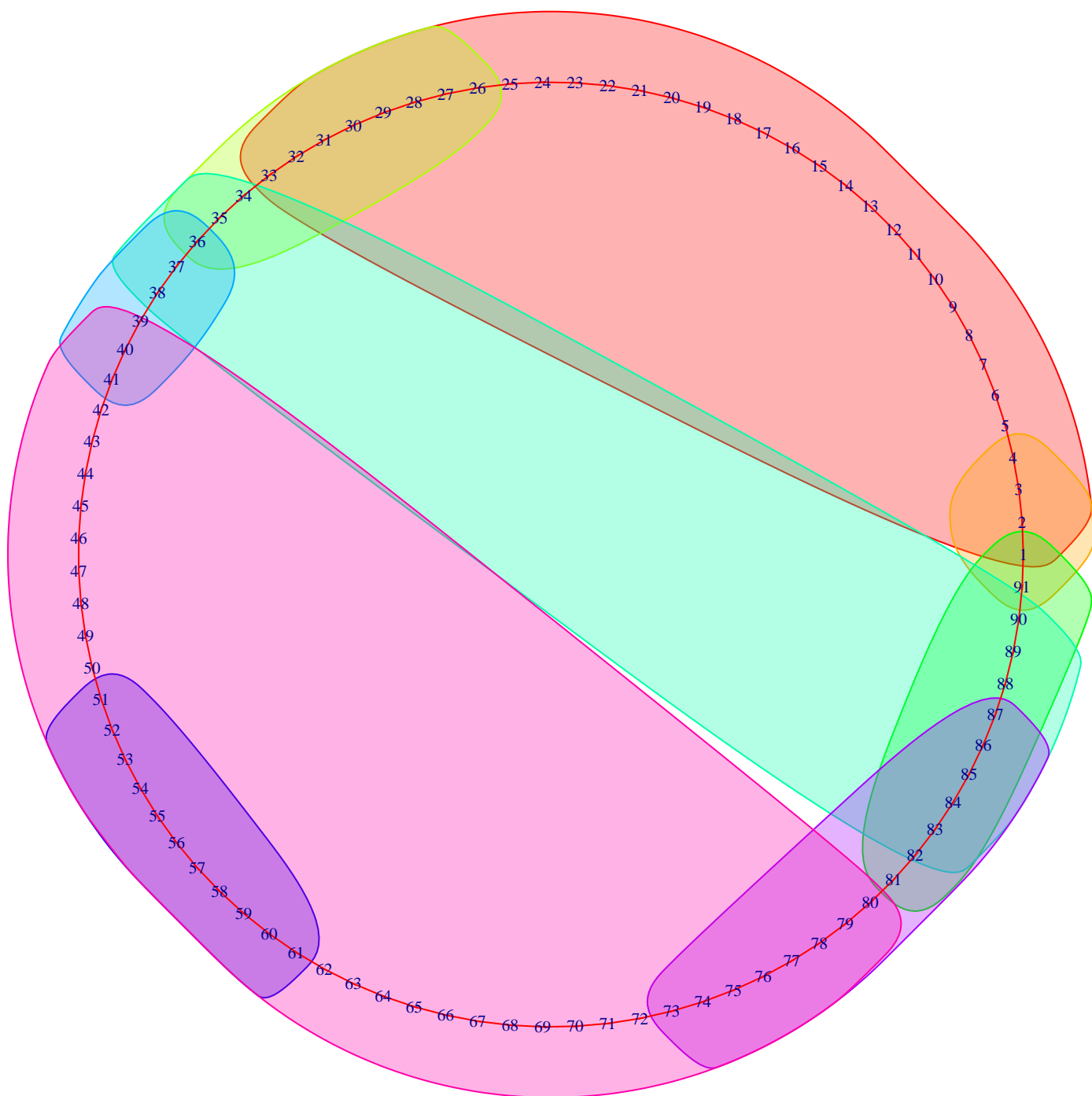

Supplement: Supplementary file 1 [file brainsci-09-00144-s001.zip › Supplementary 2/Mapper_graphs/158136_graph2B.pdf]

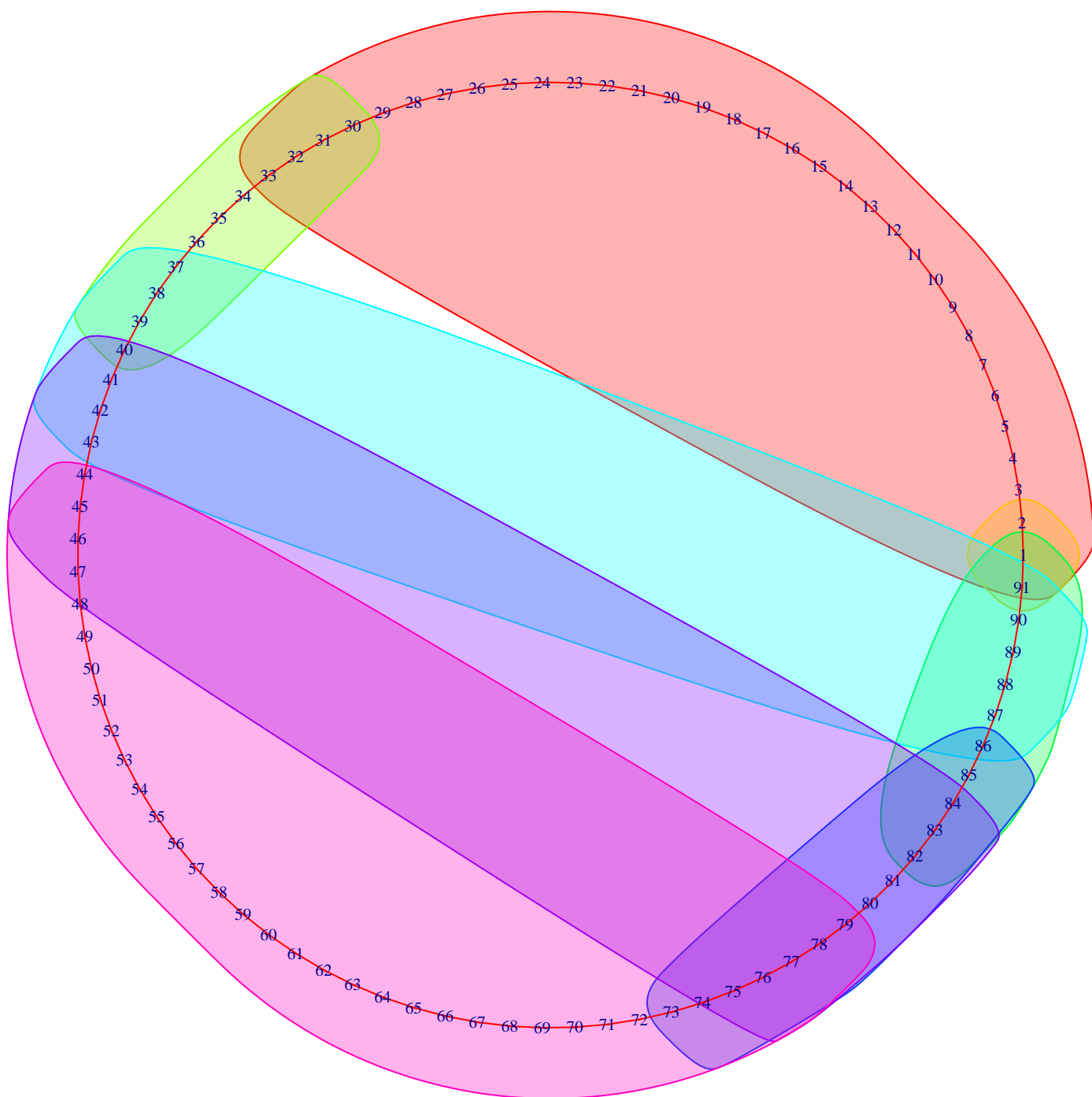

Supplement: Supplementary file 1 [file brainsci-09-00144-s001.zip › Supplementary 2/Mapper_graphs/283543_2B.pdf]

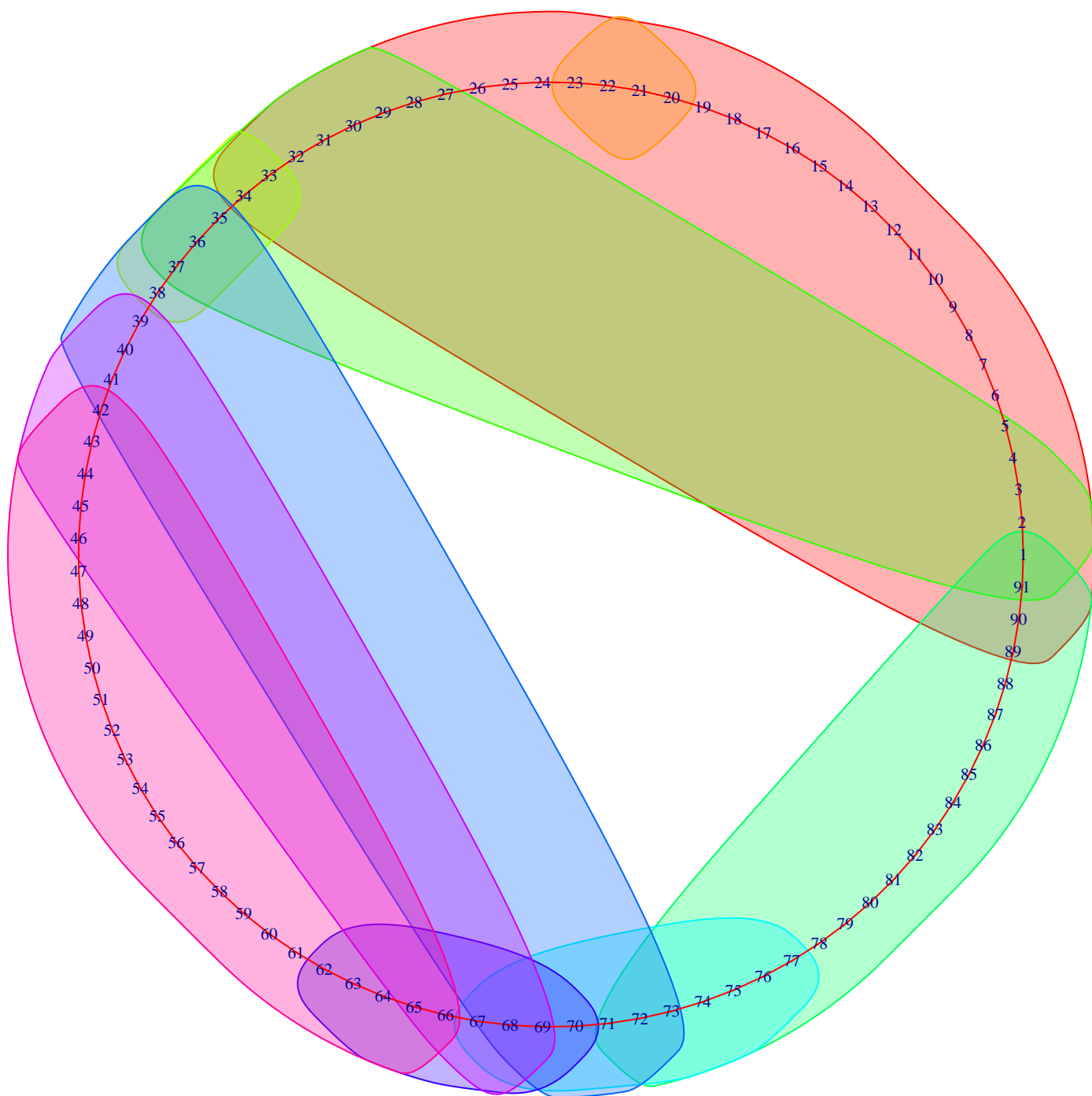

Supplement: Supplementary file 1 [file brainsci-09-00144-s001.zip › Supplementary 2/Mapper_graphs/352738_2B.pdf]

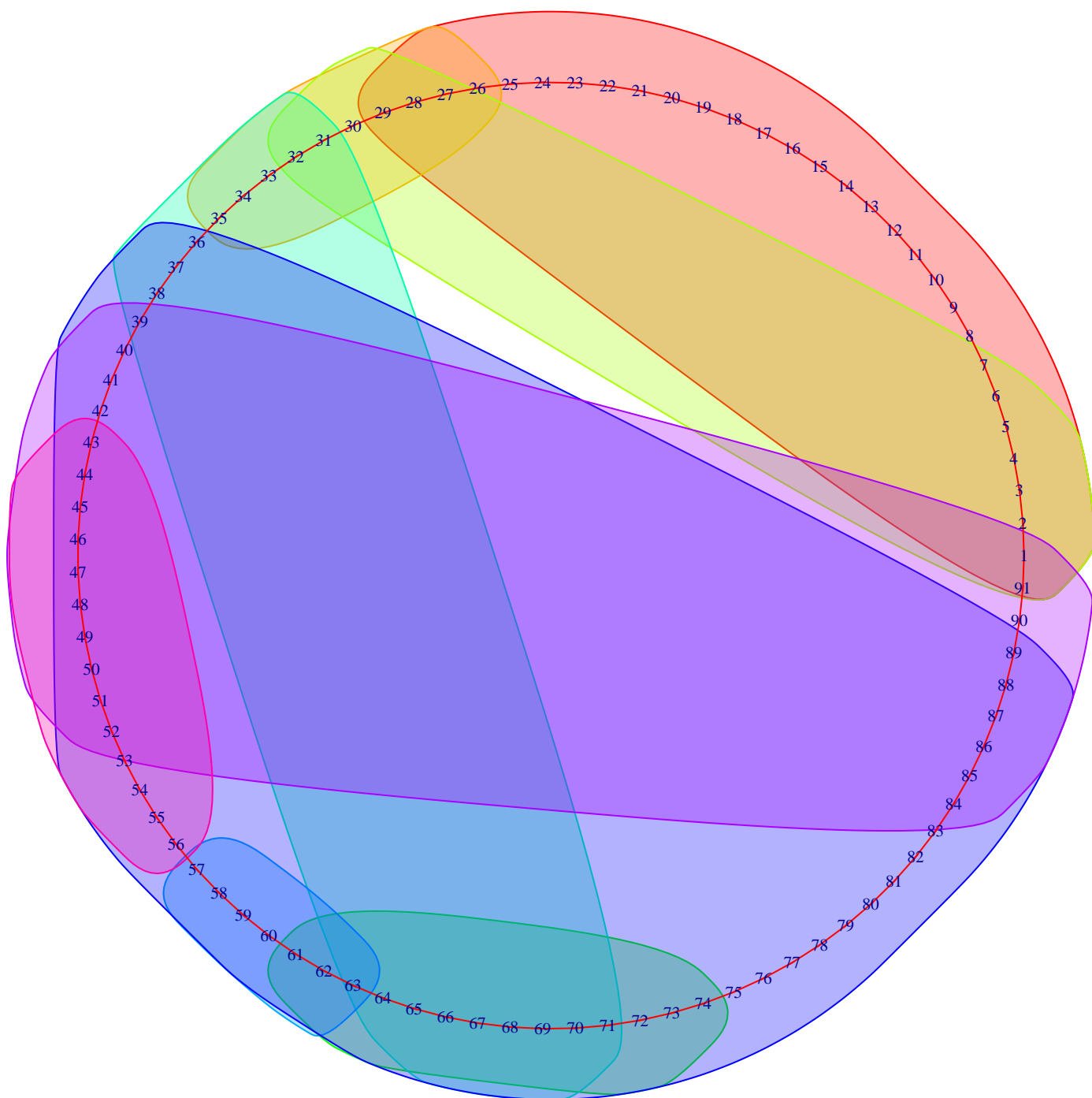

Supplement: Supplementary file 1 [file brainsci-09-00144-s001.zip › Supplementary 2/Mapper_graphs/185442_0B.pdf]

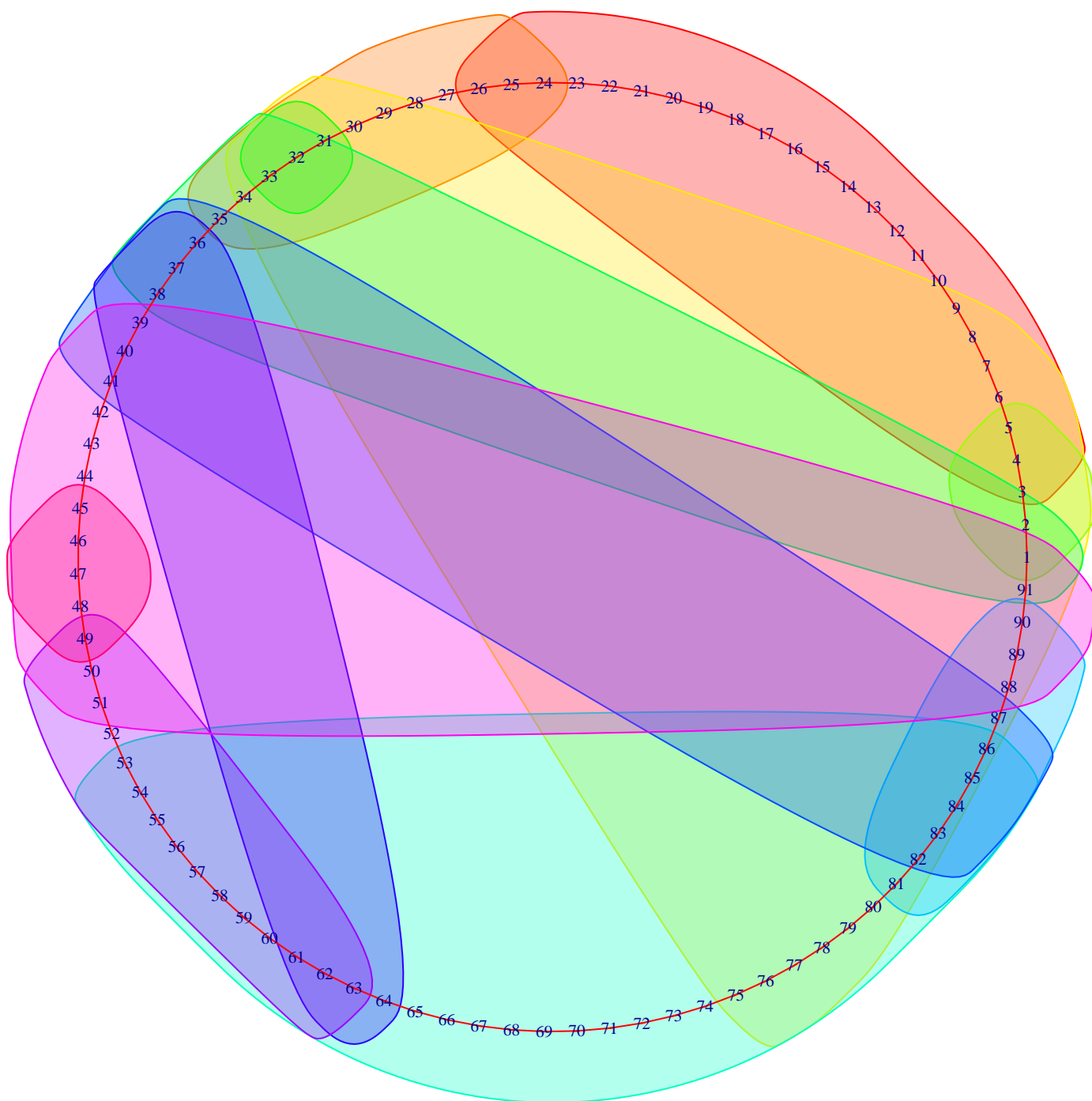

Supplement: Supplementary file 1 [file brainsci-09-00144-s001.zip › Supplementary 2/Mapper_graphs/140117_graph0B.pdf]

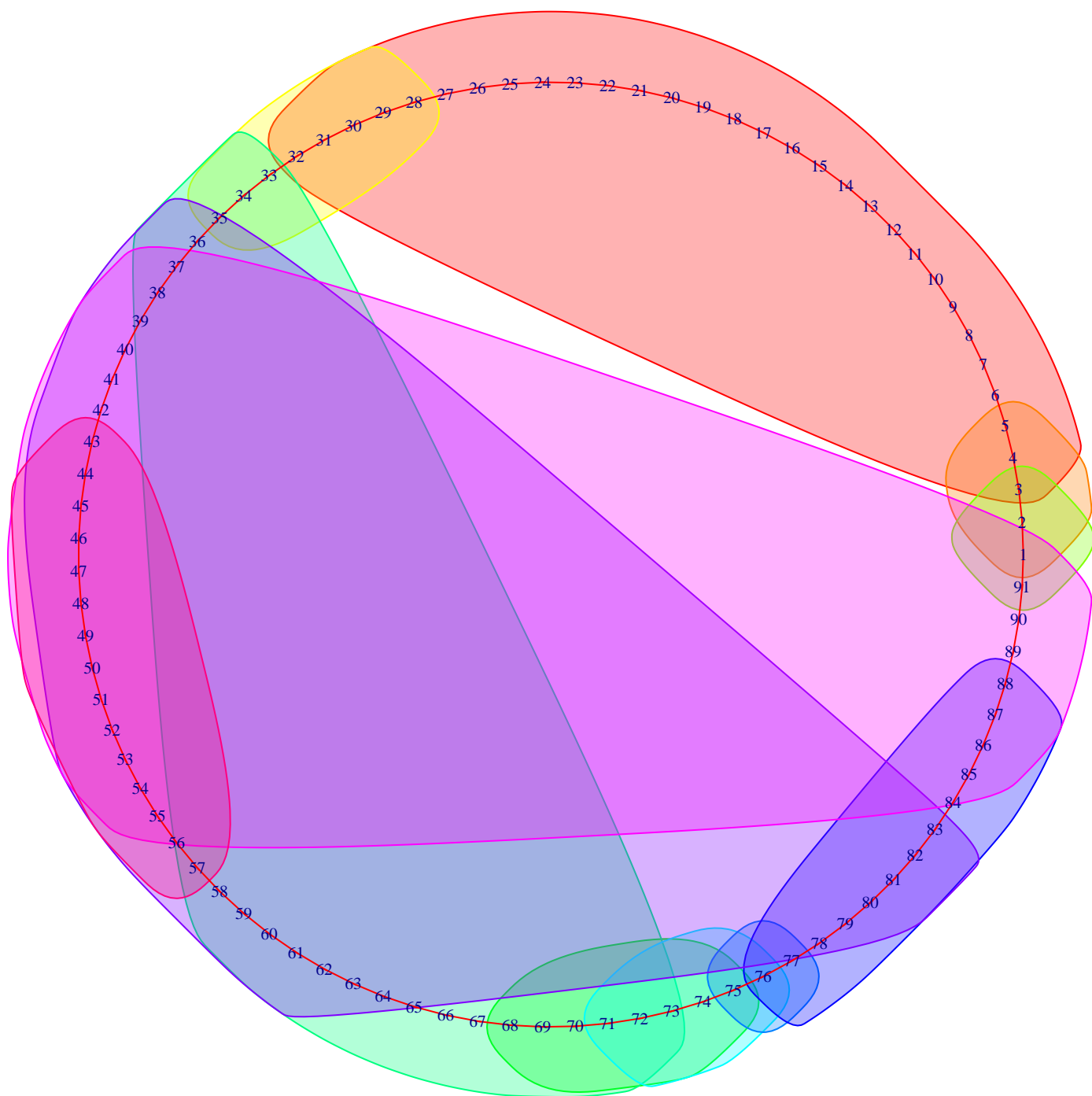

Supplement: Supplementary file 1 [file brainsci-09-00144-s001.zip › Supplementary 2/Mapper_graphs/108323_graph0B.pdf]

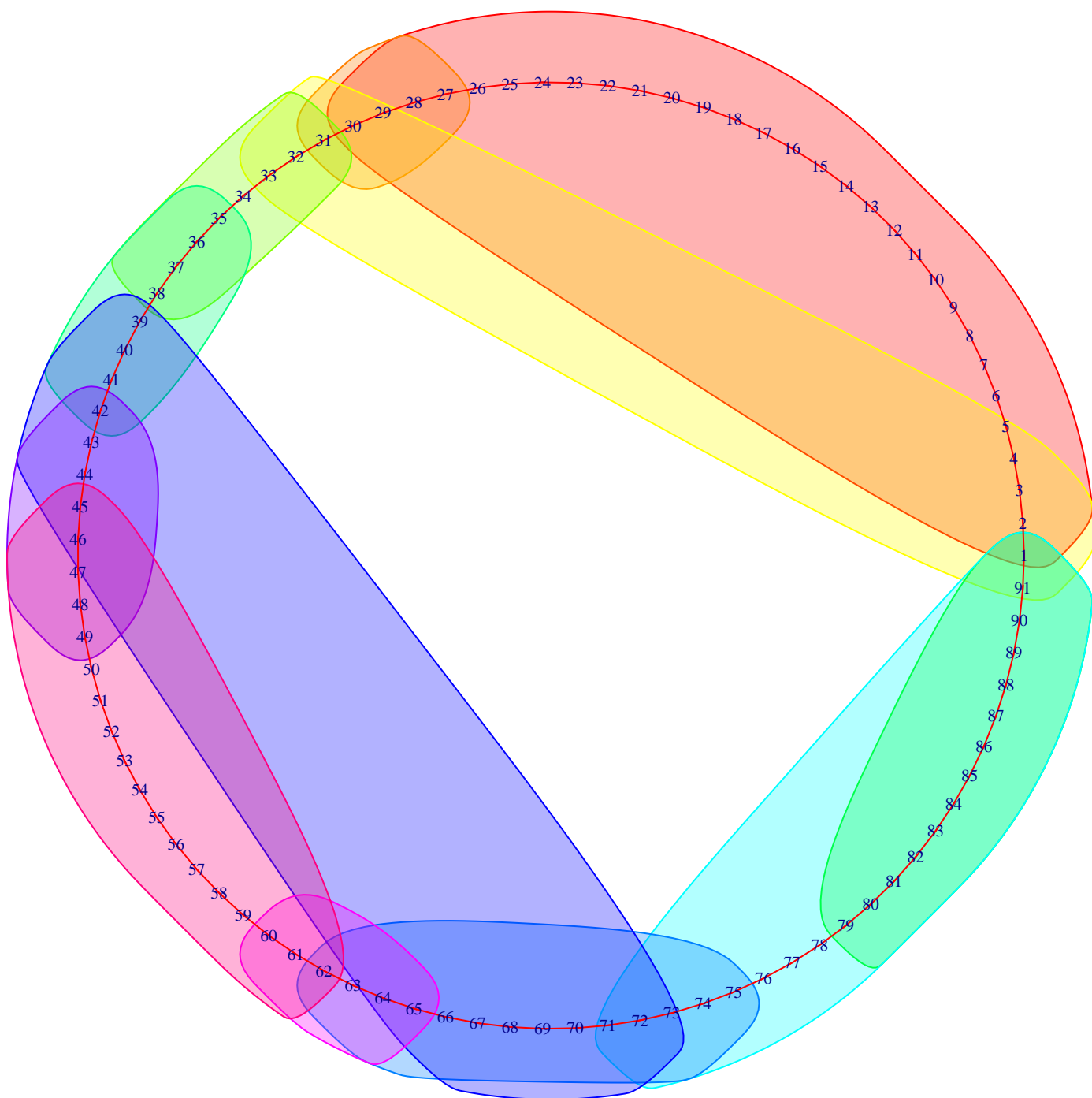

Supplement: Supplementary file 1 [file brainsci-09-00144-s001.zip › Supplementary 2/Mapper_graphs/146129_graph0B.pdf]

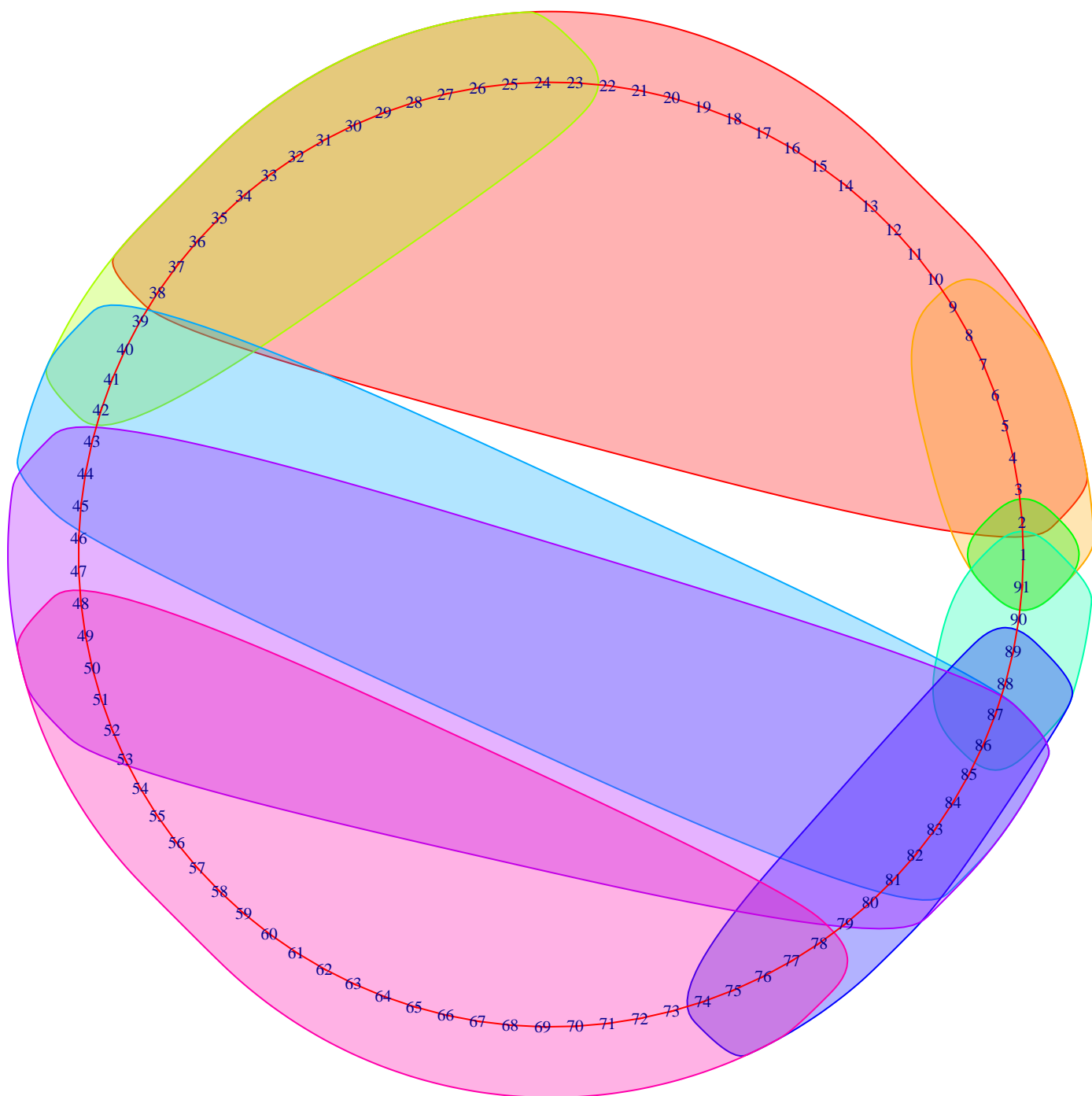

Supplement: Supplementary file 1 [file brainsci-09-00144-s001.zip › Supplementary 2/Mapper_graphs/660951_0B.pdf]

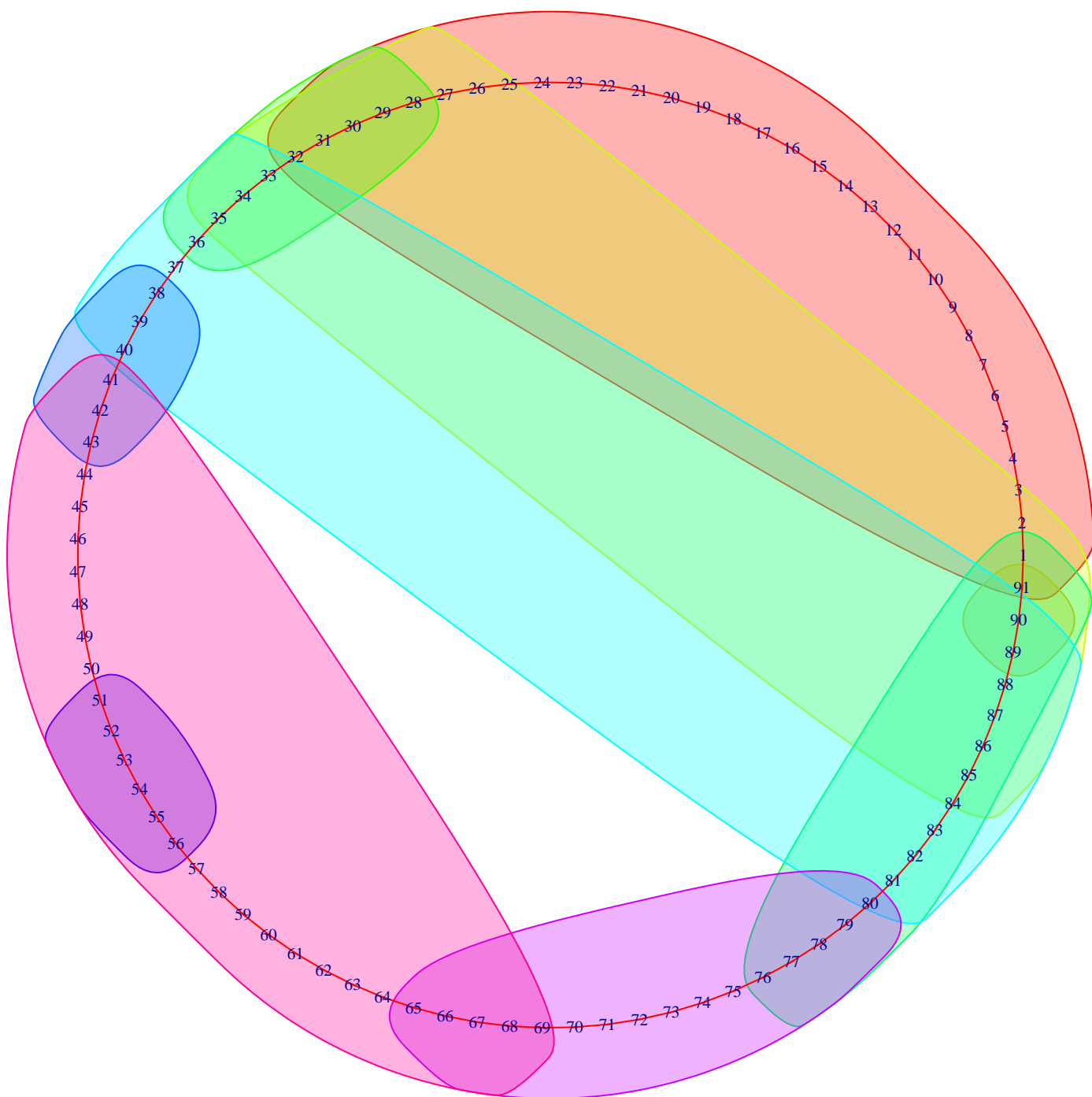

Supplement: Supplementary file 1 [file brainsci-09-00144-s001.zip › Supplementary 2/Mapper_graphs/783462_0B.pdf]

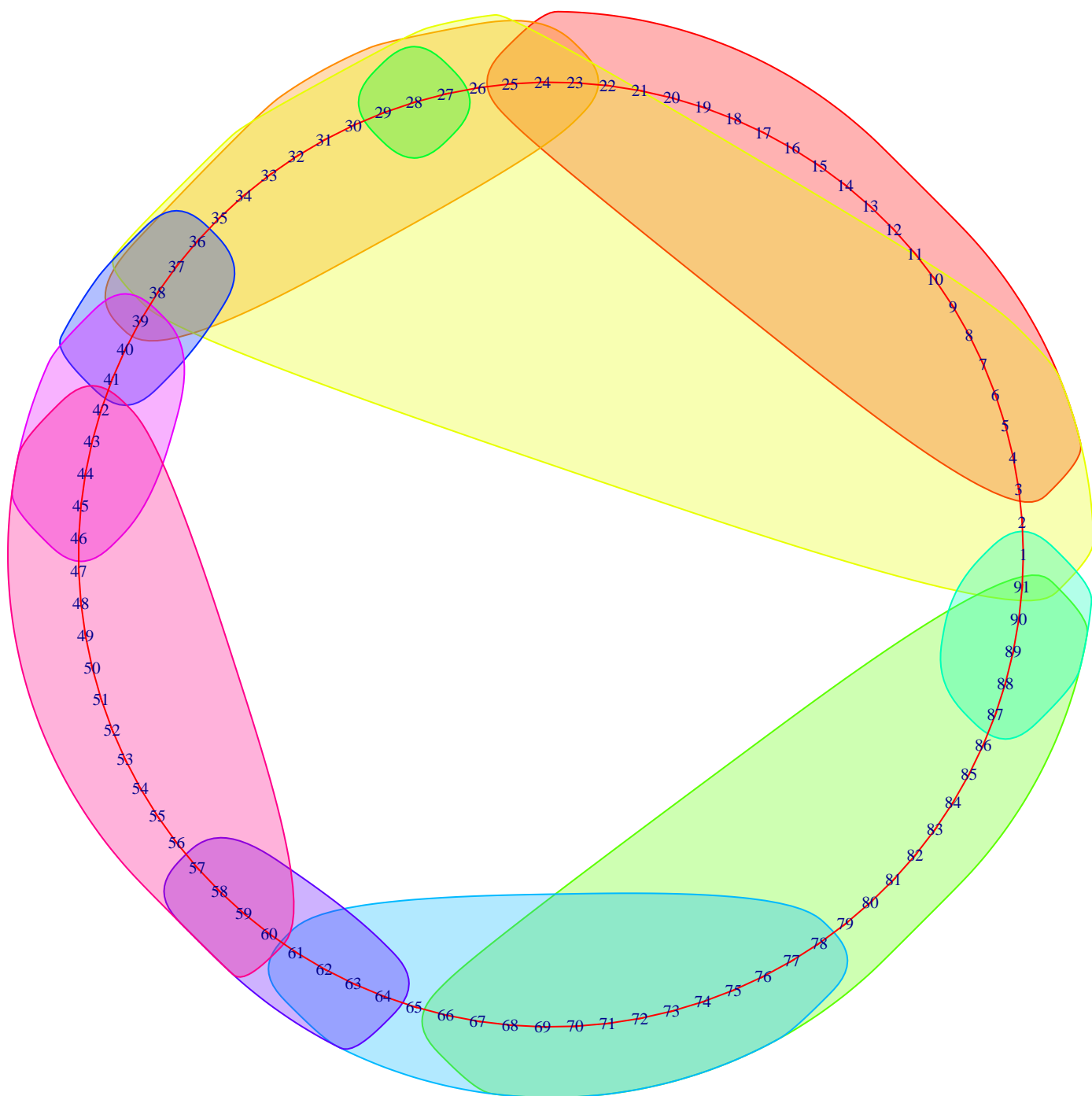

Supplement: Supplementary file 1 [file brainsci-09-00144-s001.zip › Supplementary 2/Mapper_graphs/735148_0B.pdf]

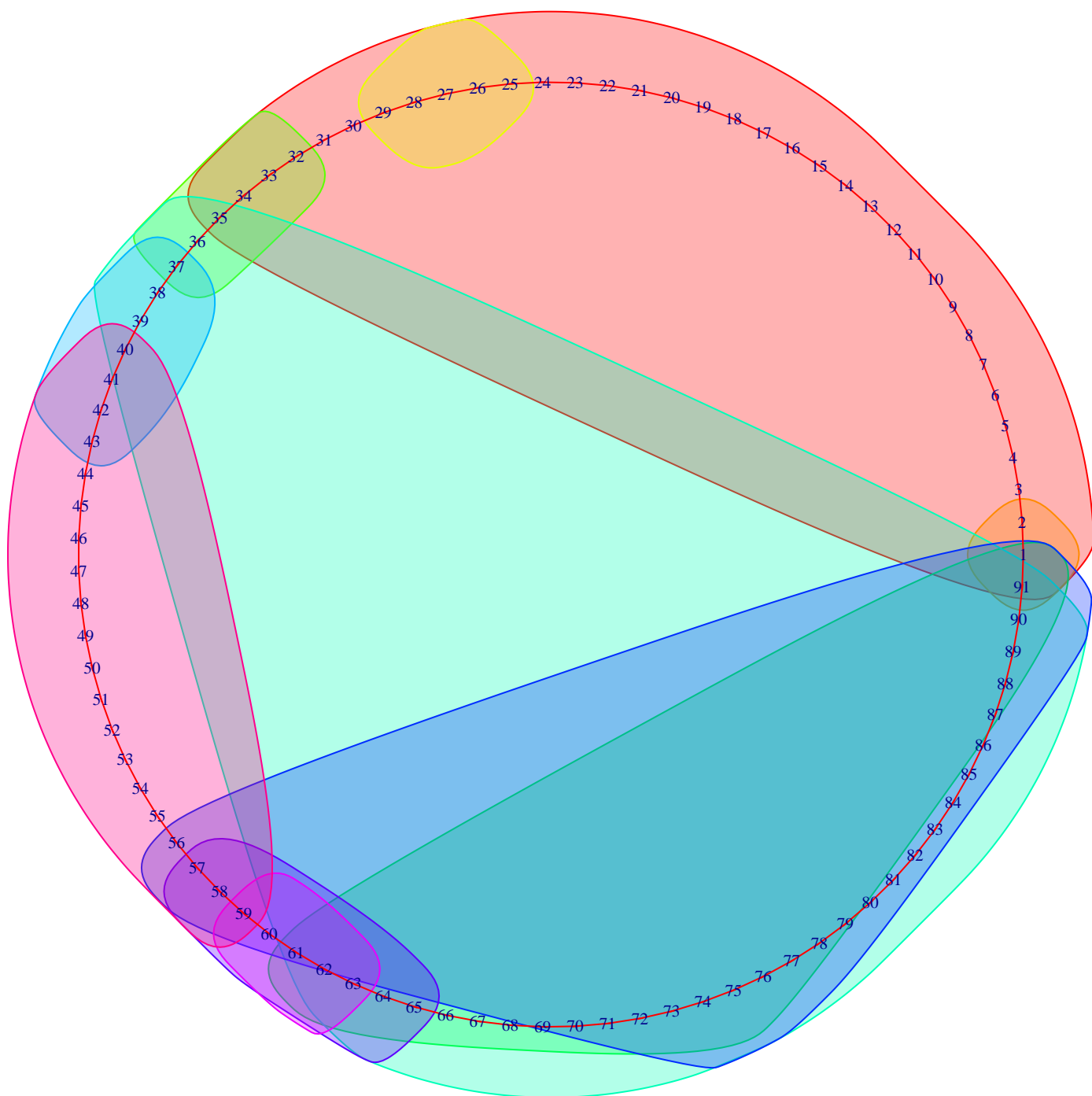

Supplement: Supplementary file 1 [file brainsci-09-00144-s001.zip › Supplementary 2/Mapper_graphs/156334_graph0B.pdf]

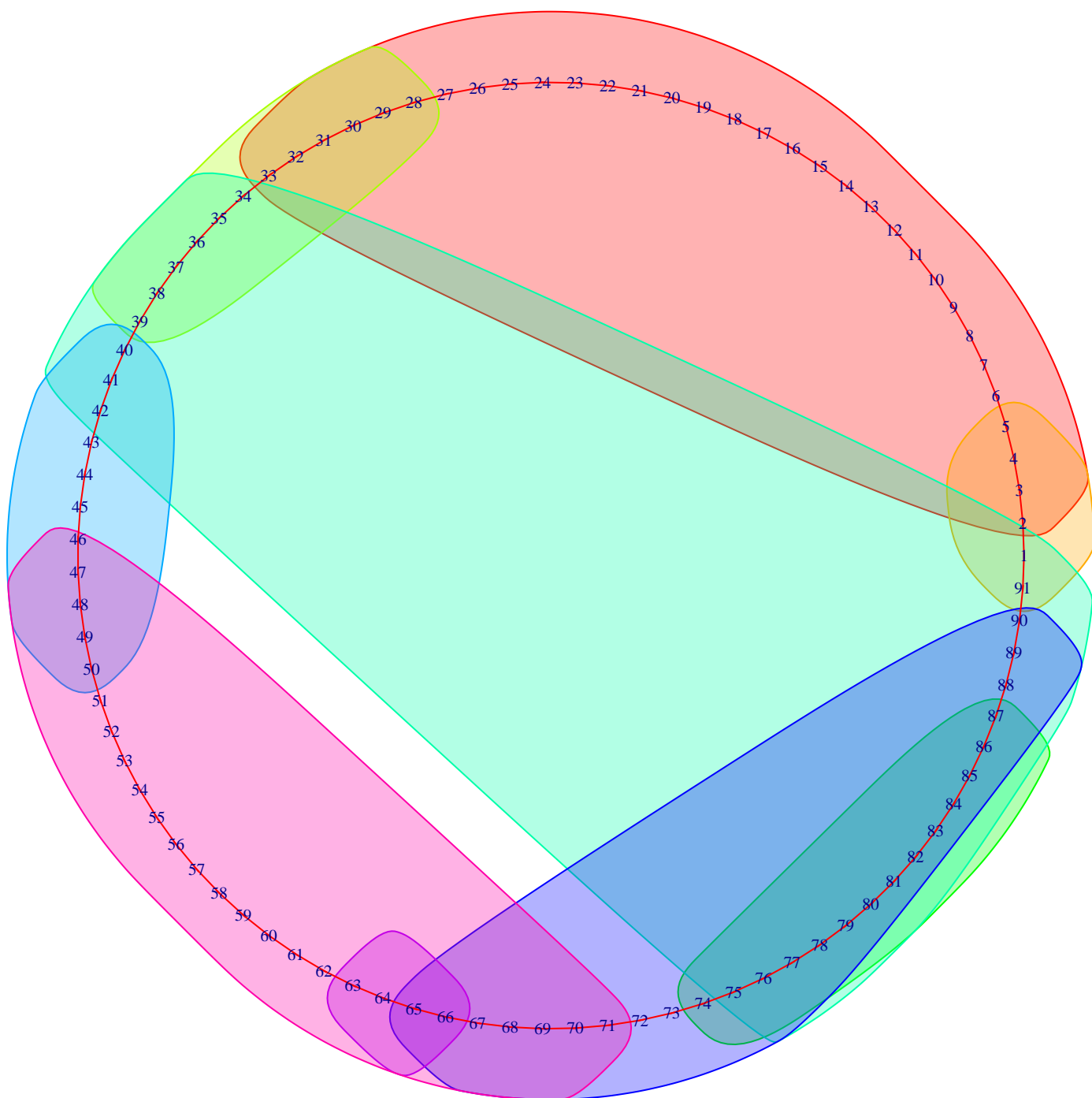

Supplement: Supplementary file 1 [file brainsci-09-00144-s001.zip › Supplementary 2/Mapper_graphs/725751_0B.pdf]

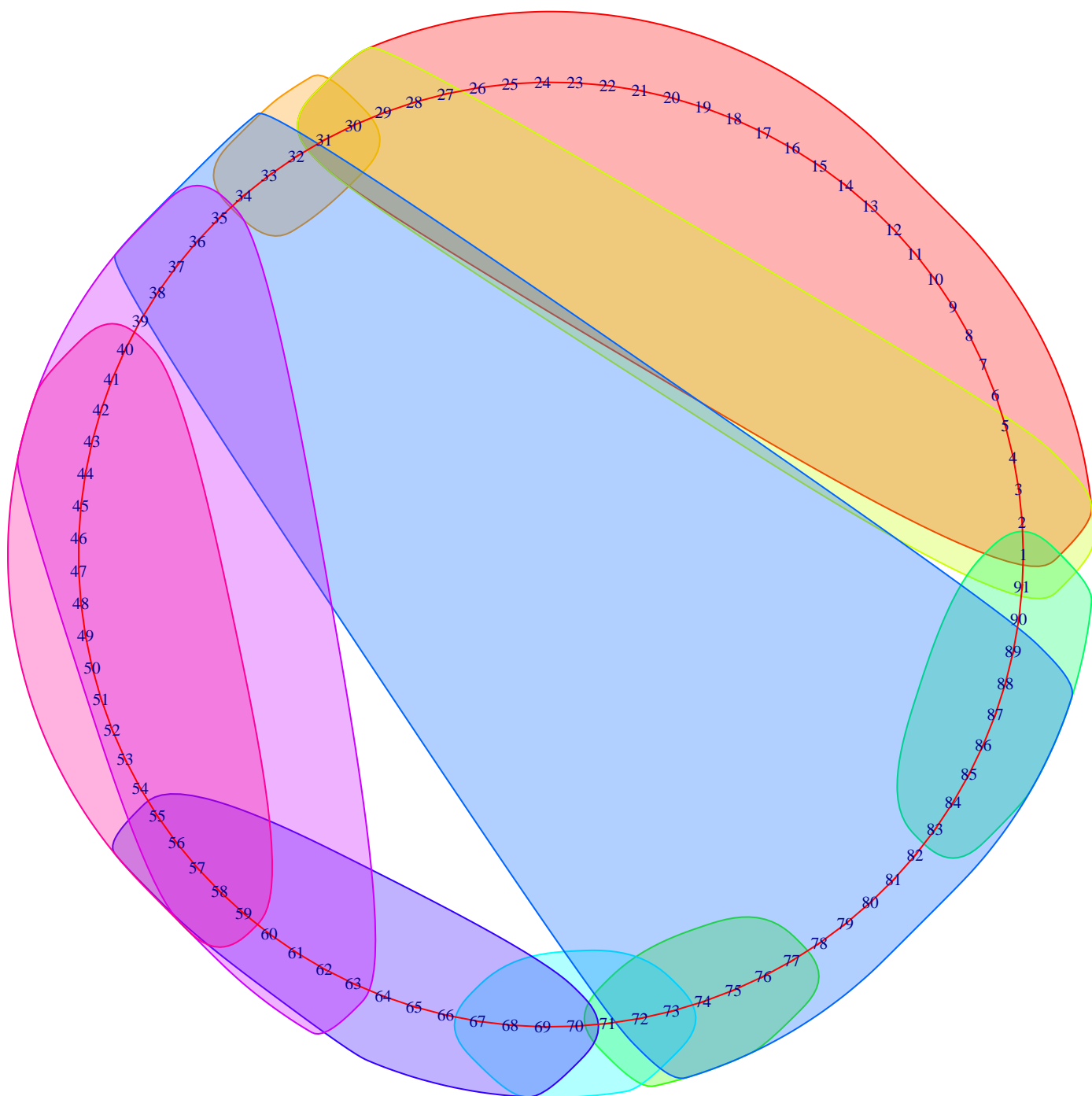

Supplement: Supplementary file 1 [file brainsci-09-00144-s001.zip › Supplementary 2/Mapper_graphs/667056_0B.pdf]

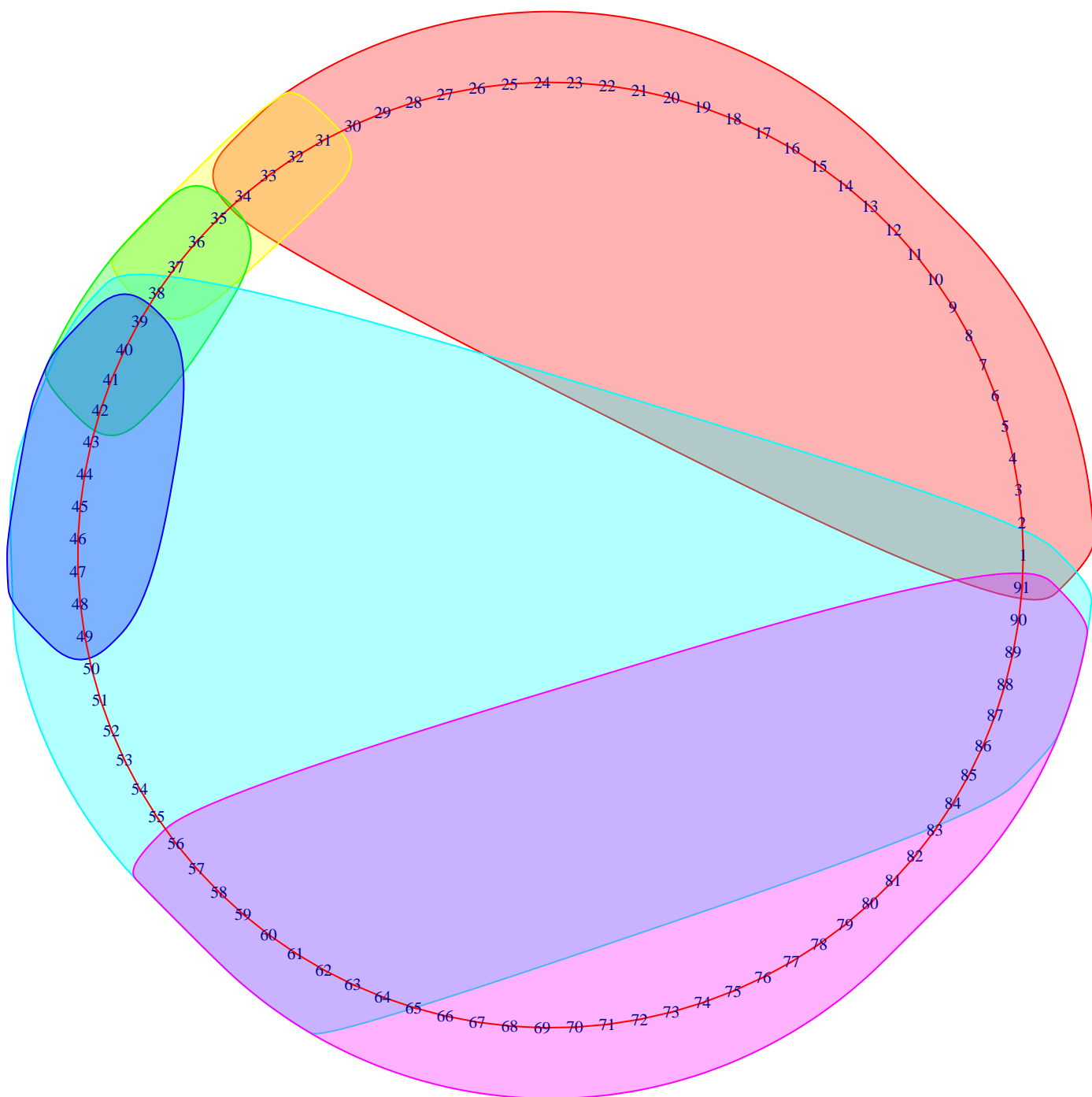

Supplement: Supplementary file 1 [file brainsci-09-00144-s001.zip › Supplementary 2/Mapper_graphs/191841_2B.pdf]

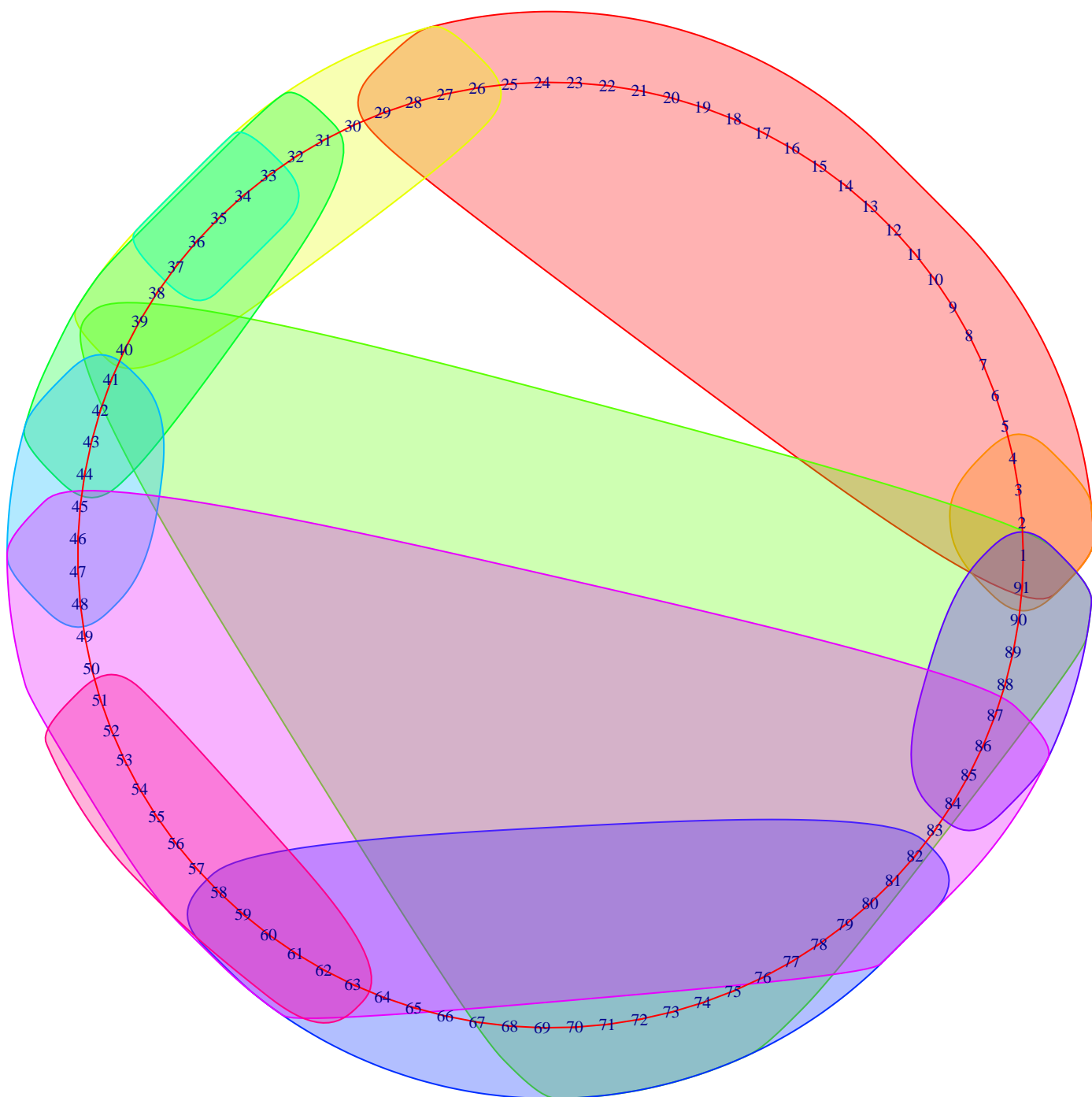

Supplement: Supplementary file 1 [file brainsci-09-00144-s001.zip › Supplementary 2/Mapper_graphs/105923_graph0B.pdf]

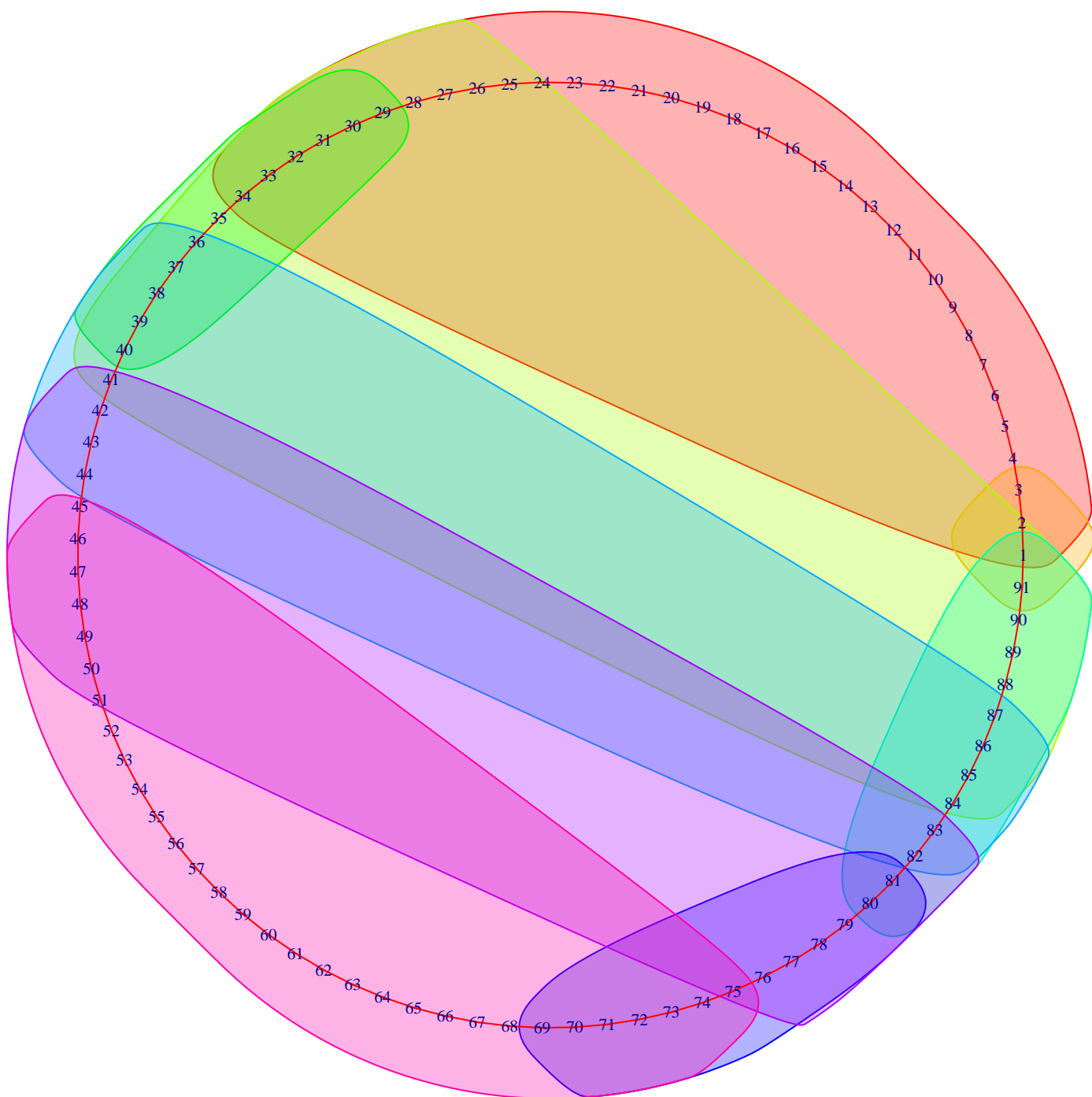

Supplement: Supplementary file 1 [file brainsci-09-00144-s001.zip › Supplementary 2/Mapper_graphs/195041_2B.pdf]

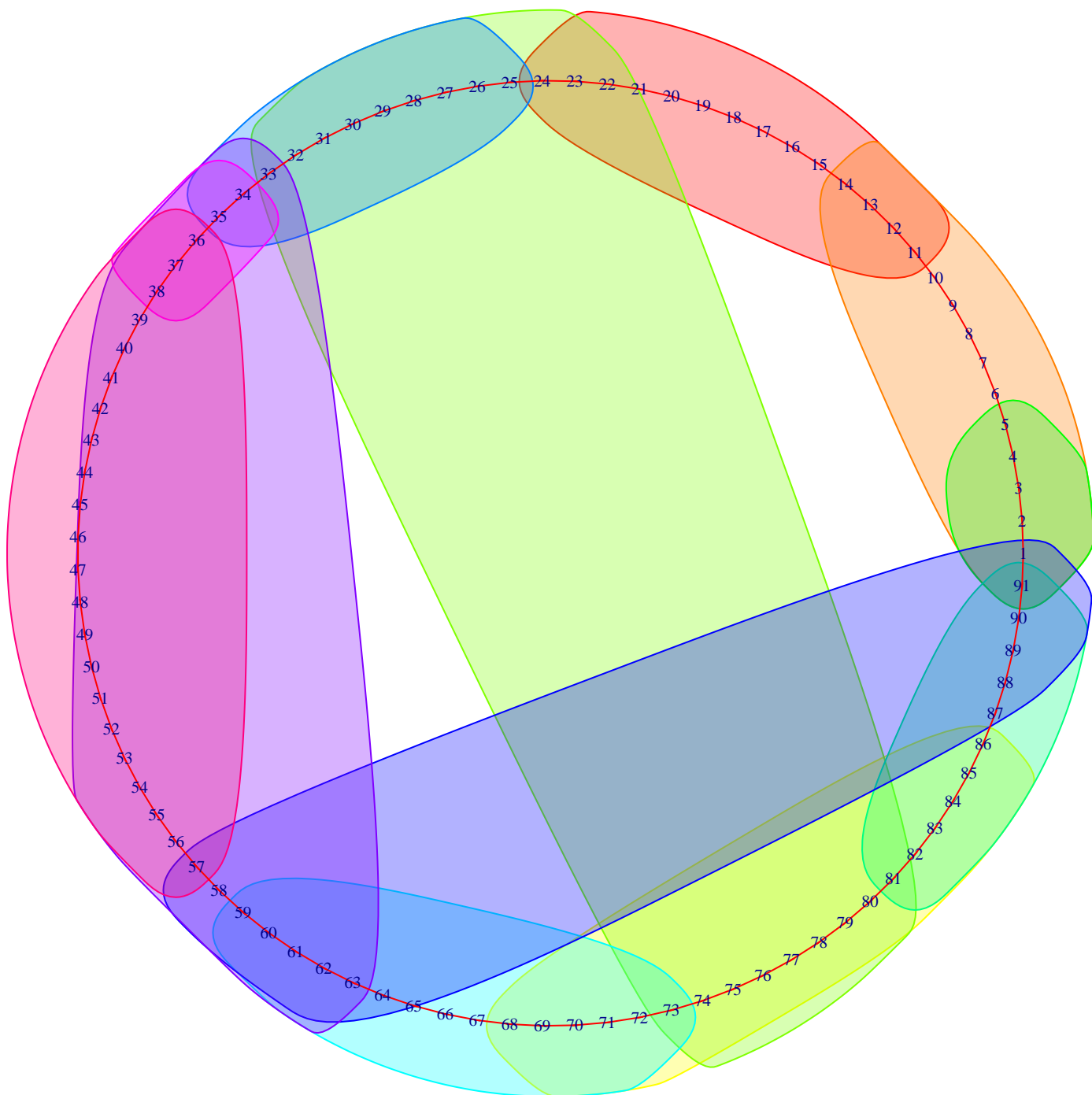

Supplement: Supplementary file 1 [file brainsci-09-00144-s001.zip › Supplementary 2/Mapper_graphs/406836_graph2B.pdf]

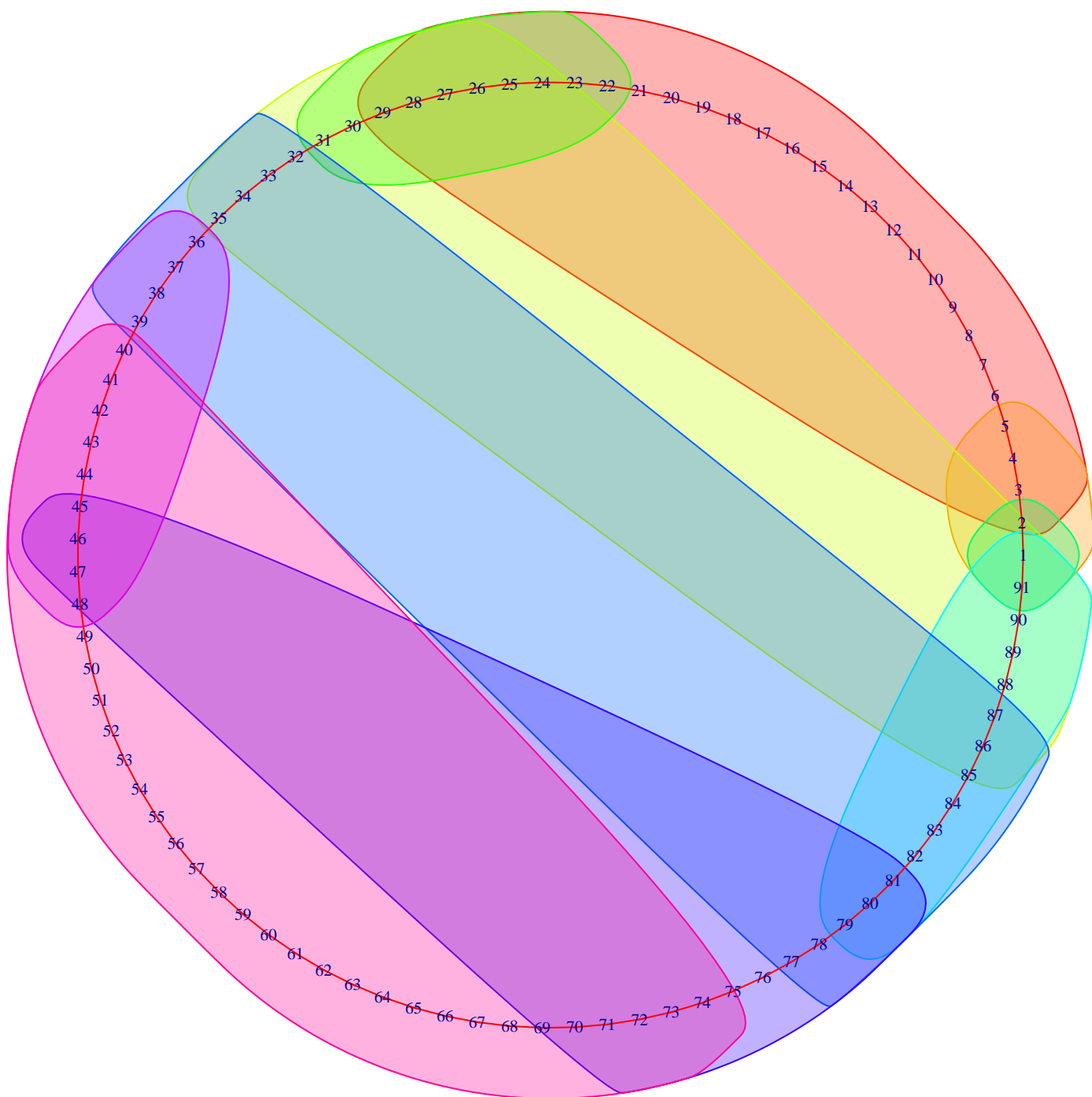

Supplement: Supplementary file 1 [file brainsci-09-00144-s001.zip › Supplementary 2/Mapper_graphs/104012_graph0B.pdf]

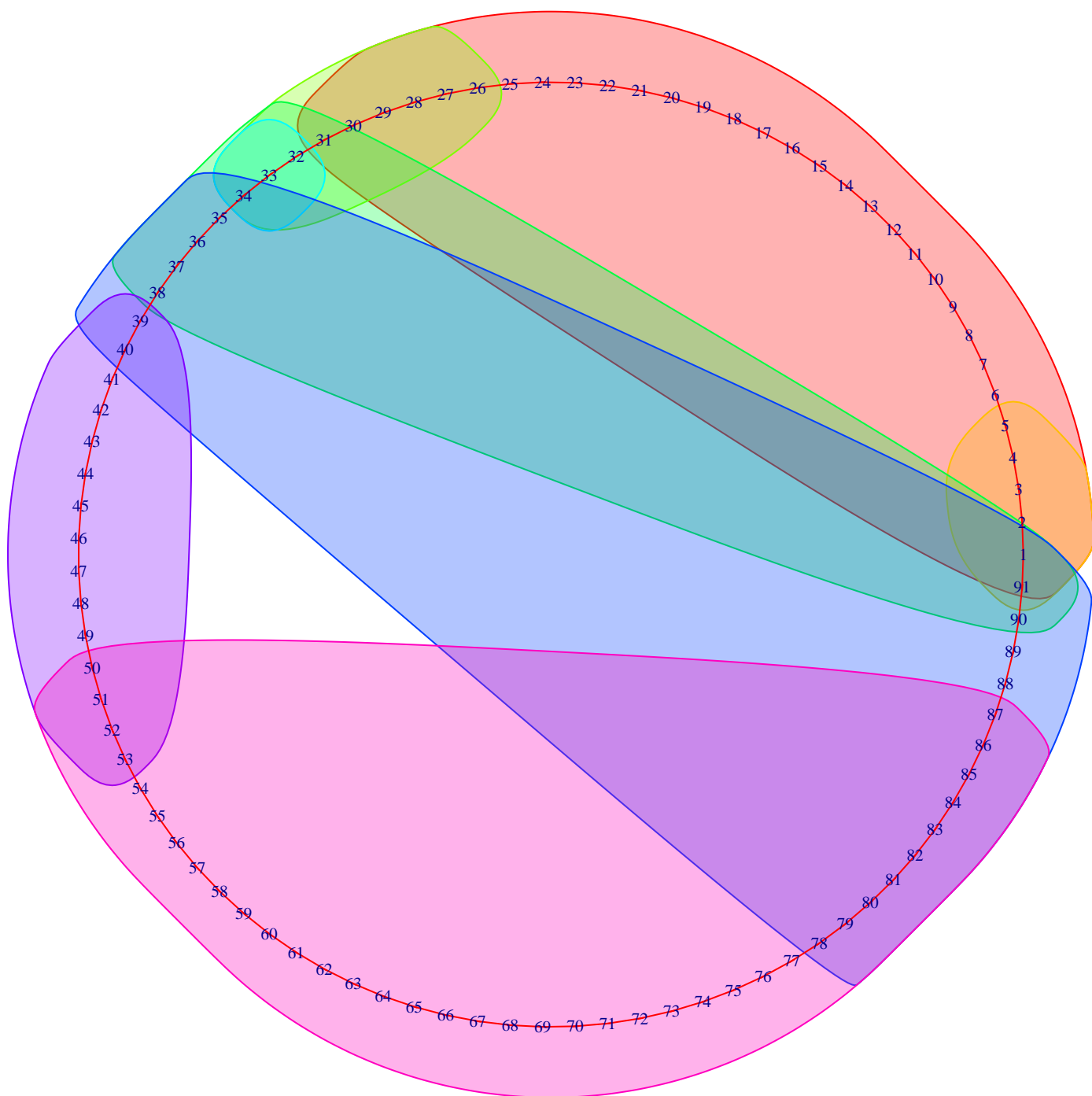

Supplement: Supplementary file 1 [file brainsci-09-00144-s001.zip › Supplementary 2/Mapper_graphs/250427_2B.pdf]

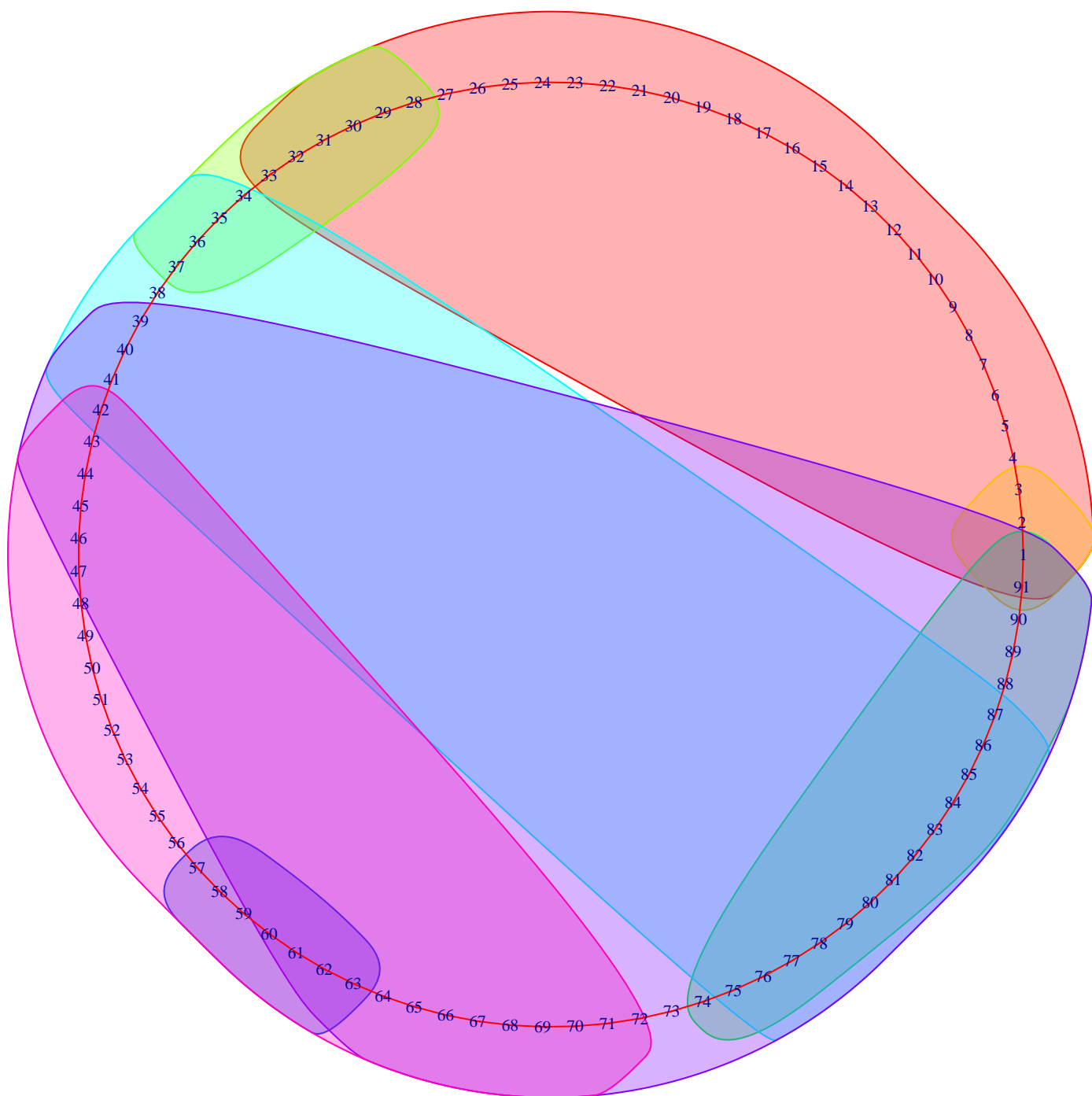

Supplement: Supplementary file 1 [file brainsci-09-00144-s001.zip › Supplementary 2/Mapper_graphs/172029_graph0B.pdf]

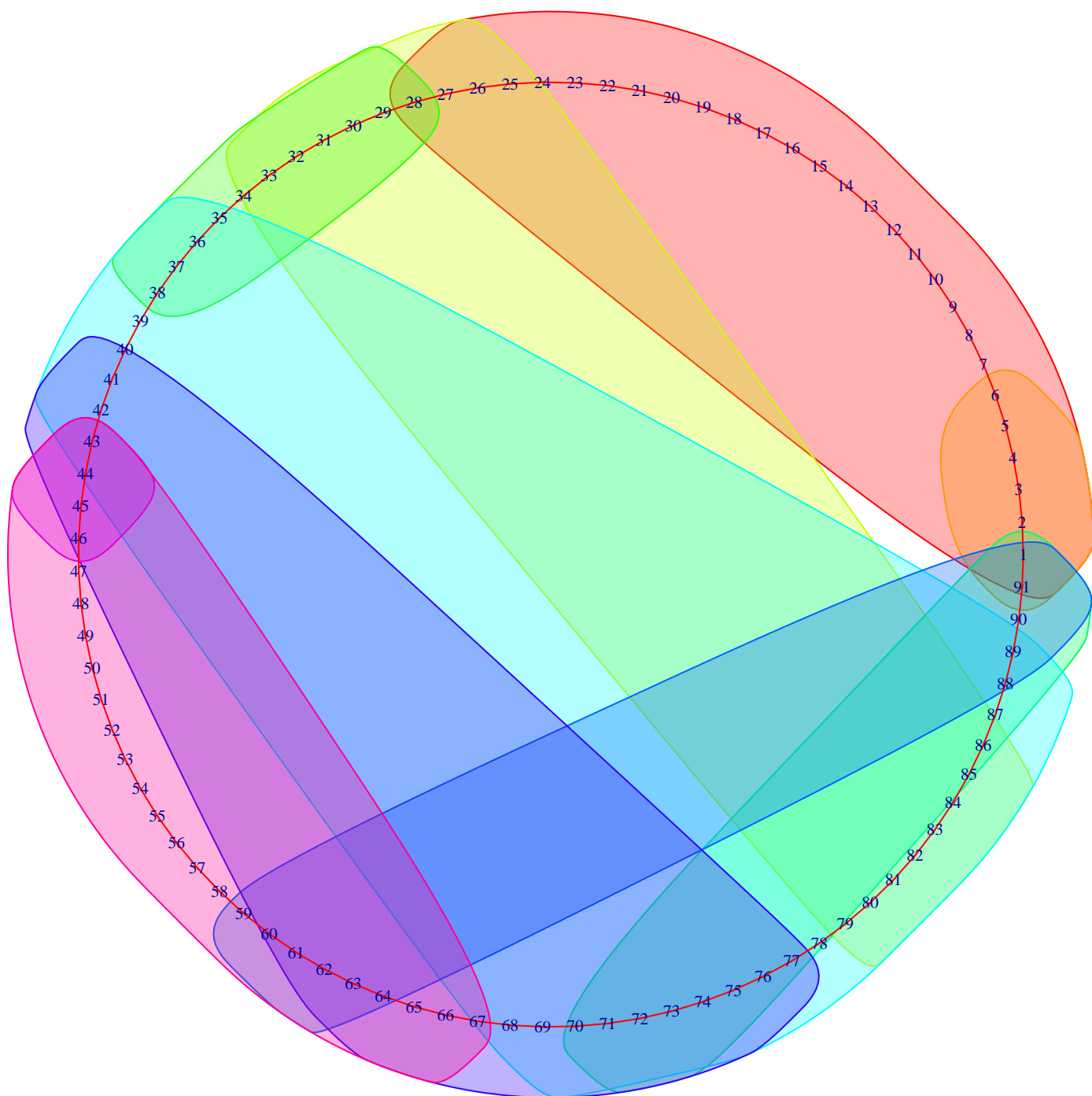

Supplement: Supplementary file 1 [file brainsci-09-00144-s001.zip › Supplementary 2/Mapper_graphs/112920_graph0B.pdf]

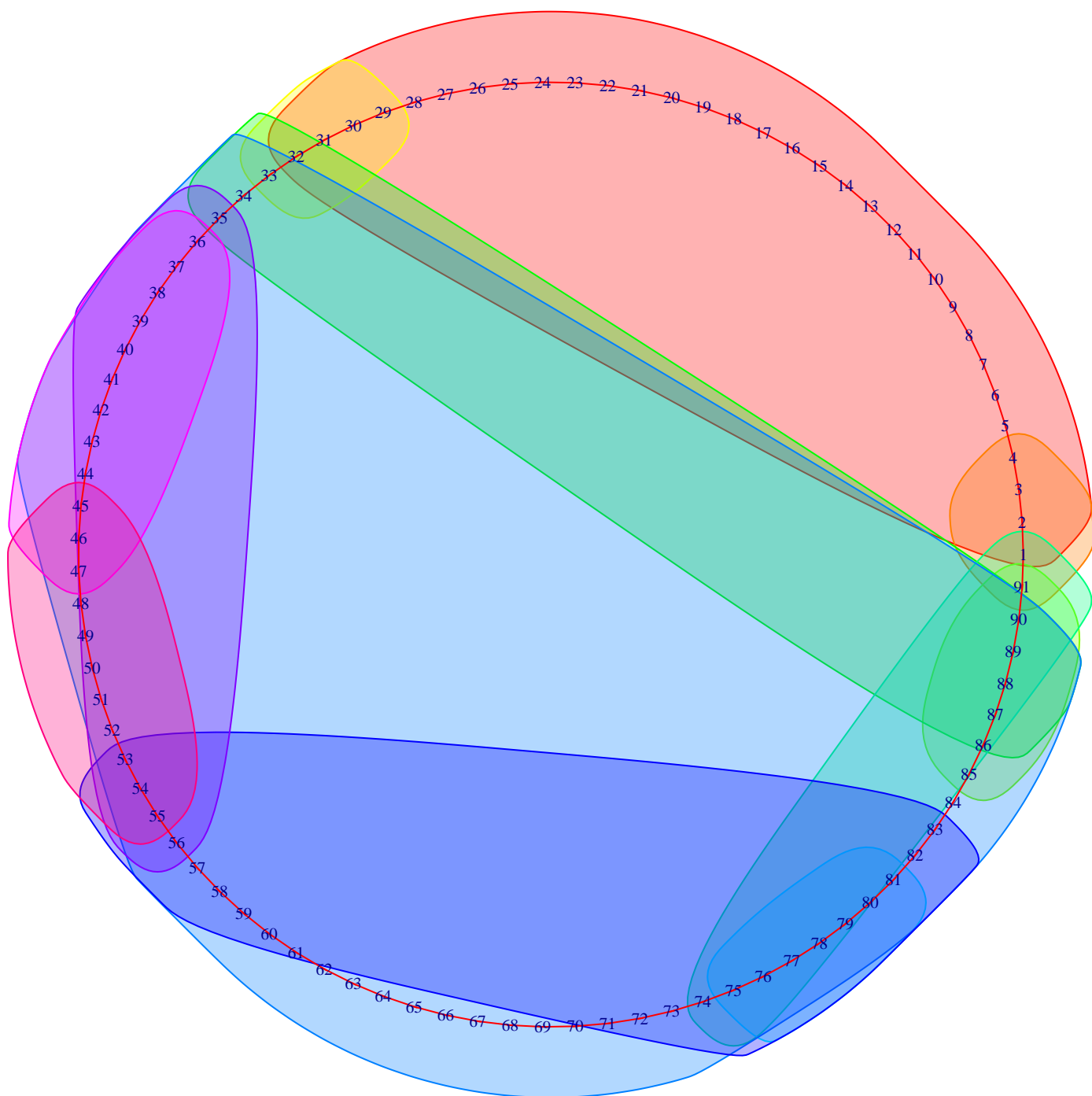

Supplement: Supplementary file 1 [file brainsci-09-00144-s001.zip › Supplementary 2/Mapper_graphs/192641_2B.pdf]

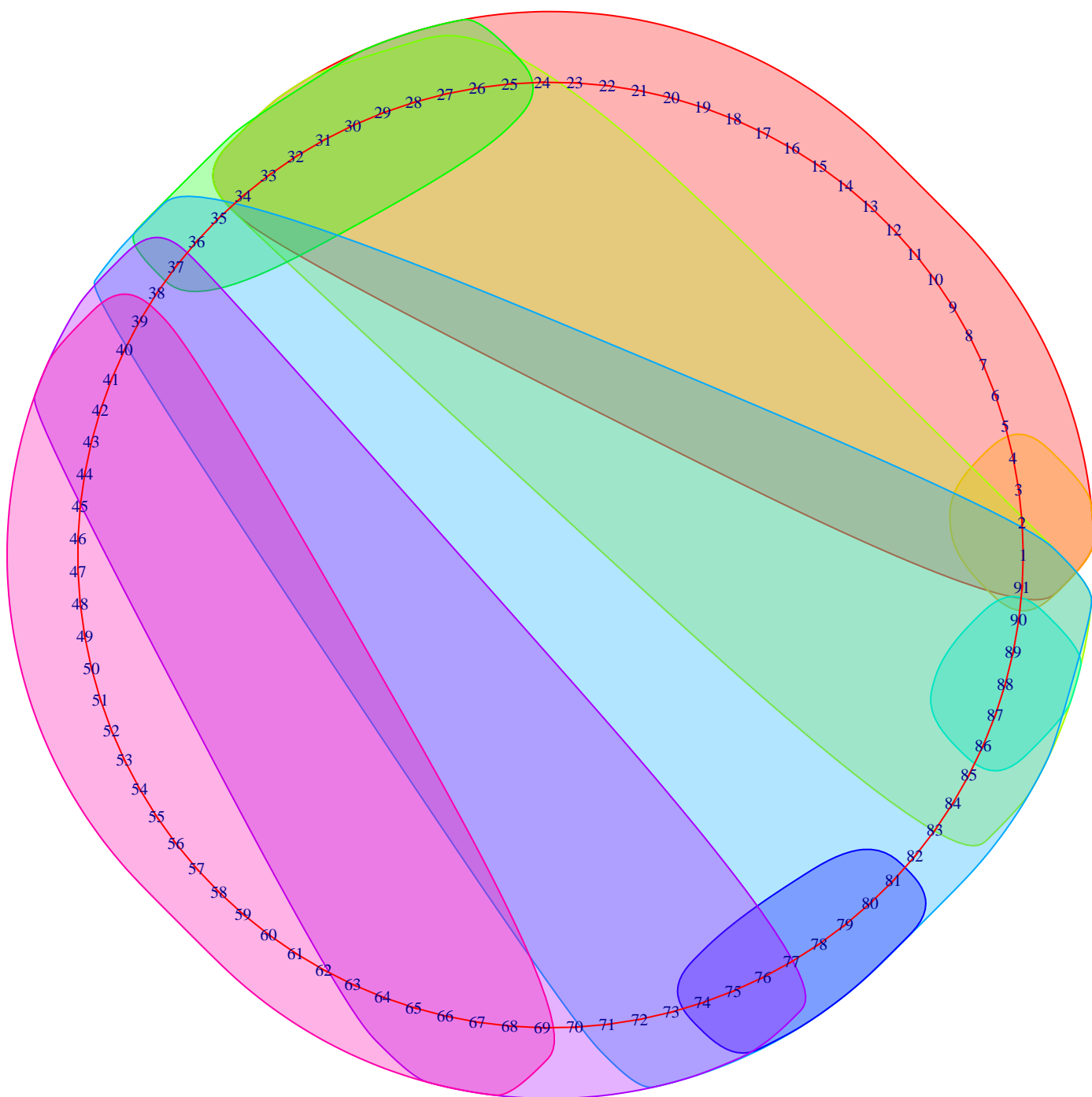

Supplement: Supplementary file 1 [file brainsci-09-00144-s001.zip › Supplementary 2/Mapper_graphs/175540_graph2B.pdf]

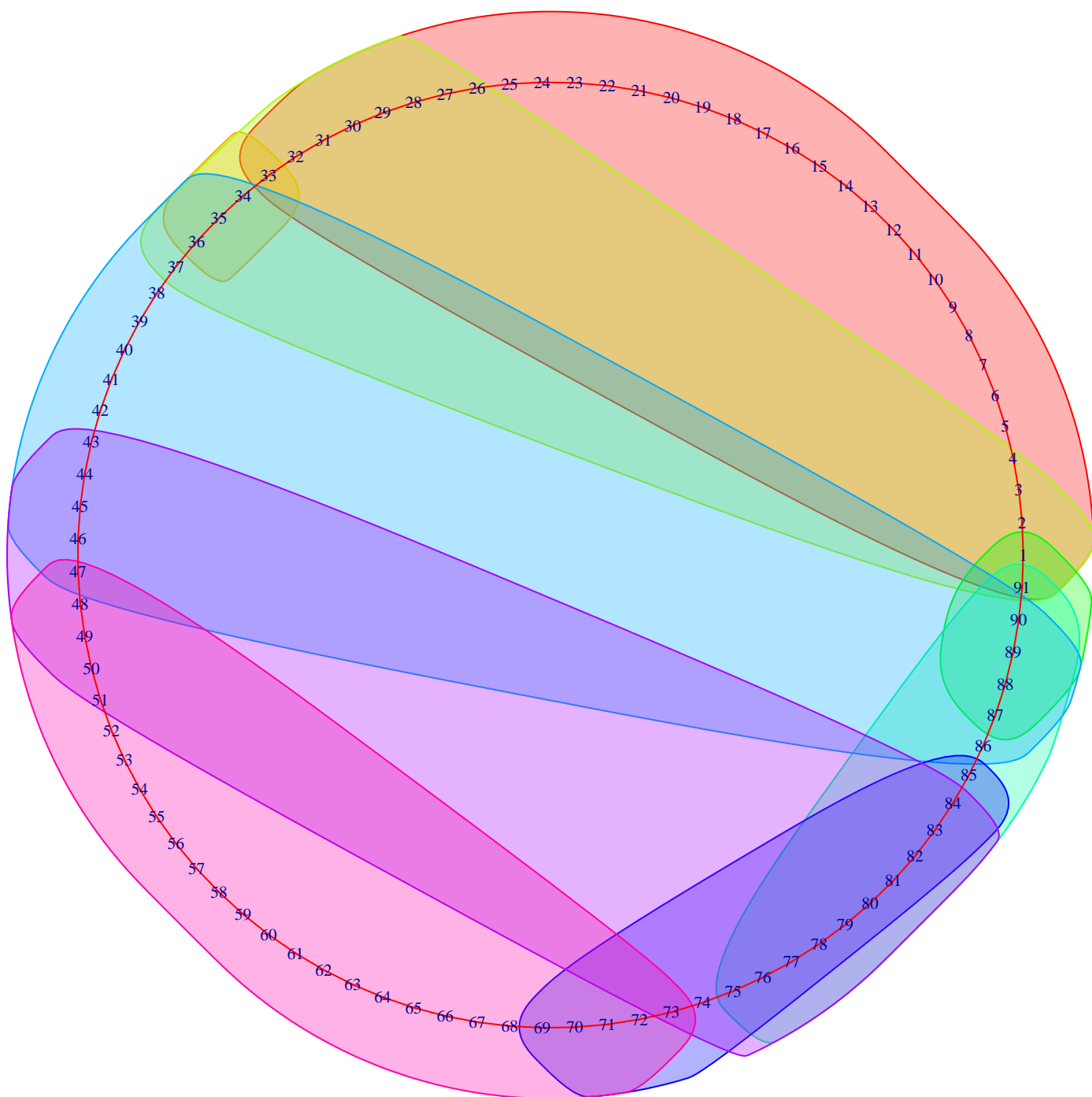

Supplement: Supplementary file 1 [file brainsci-09-00144-s001.zip › Supplementary 2/Mapper_graphs/204521_0B.pdf]

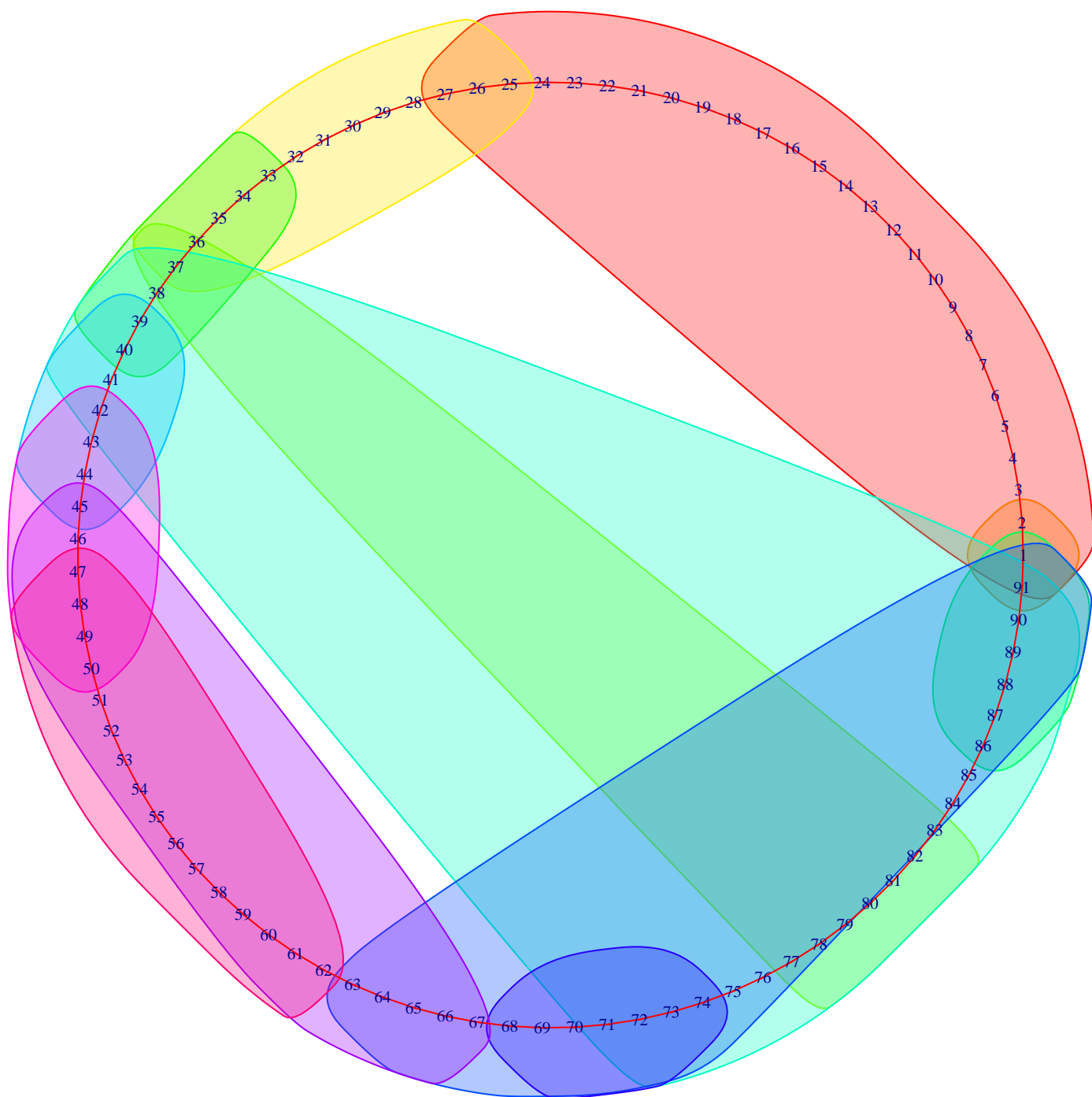

Supplement: Supplementary file 1 [file brainsci-09-00144-s001.zip › Supplementary 2/Mapper_graphs/175237_graph0B.pdf]

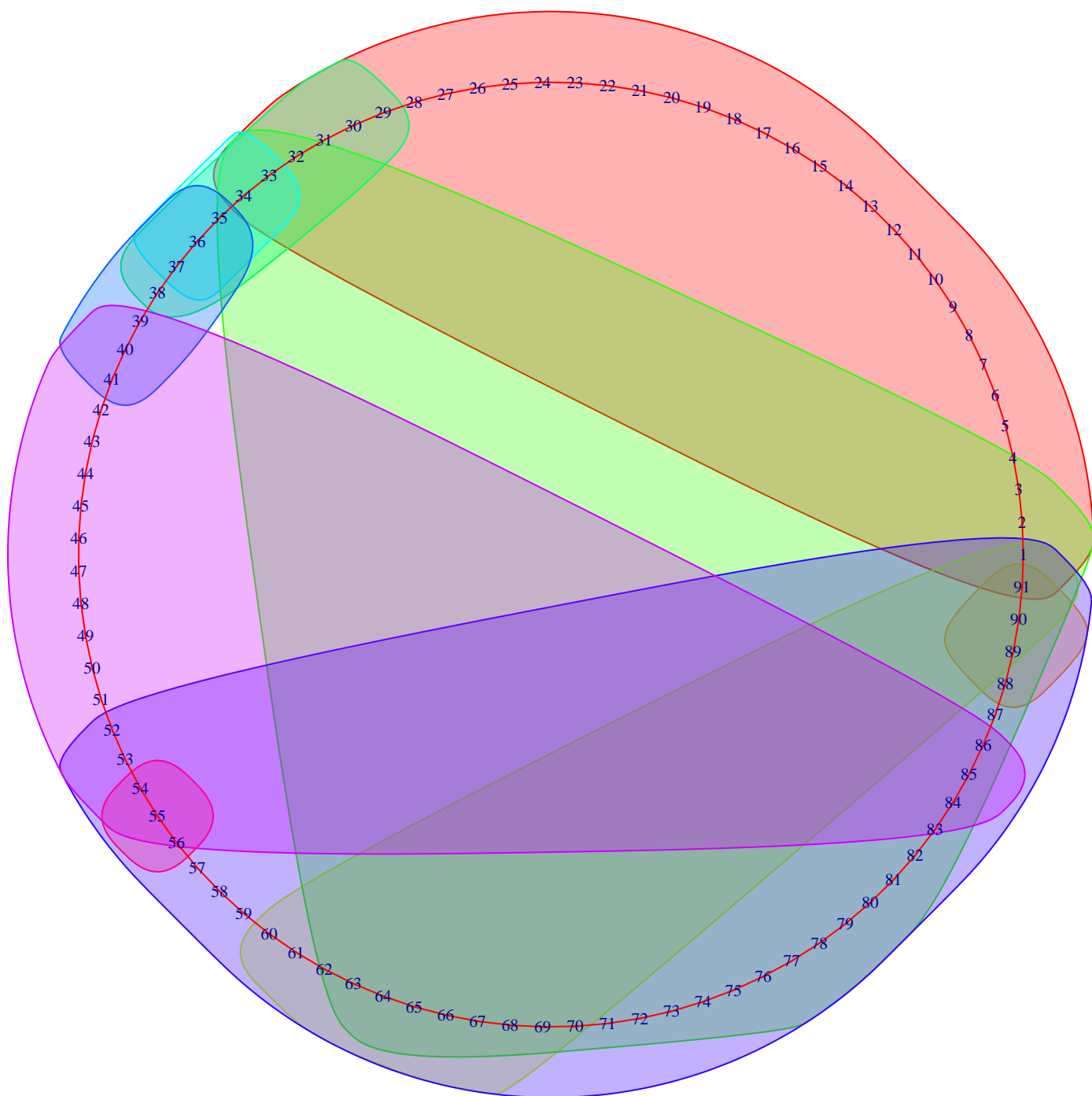

Supplement: Supplementary file 1 [file brainsci-09-00144-s001.zip › Supplementary 2/Mapper_graphs/191033_2B.pdf]

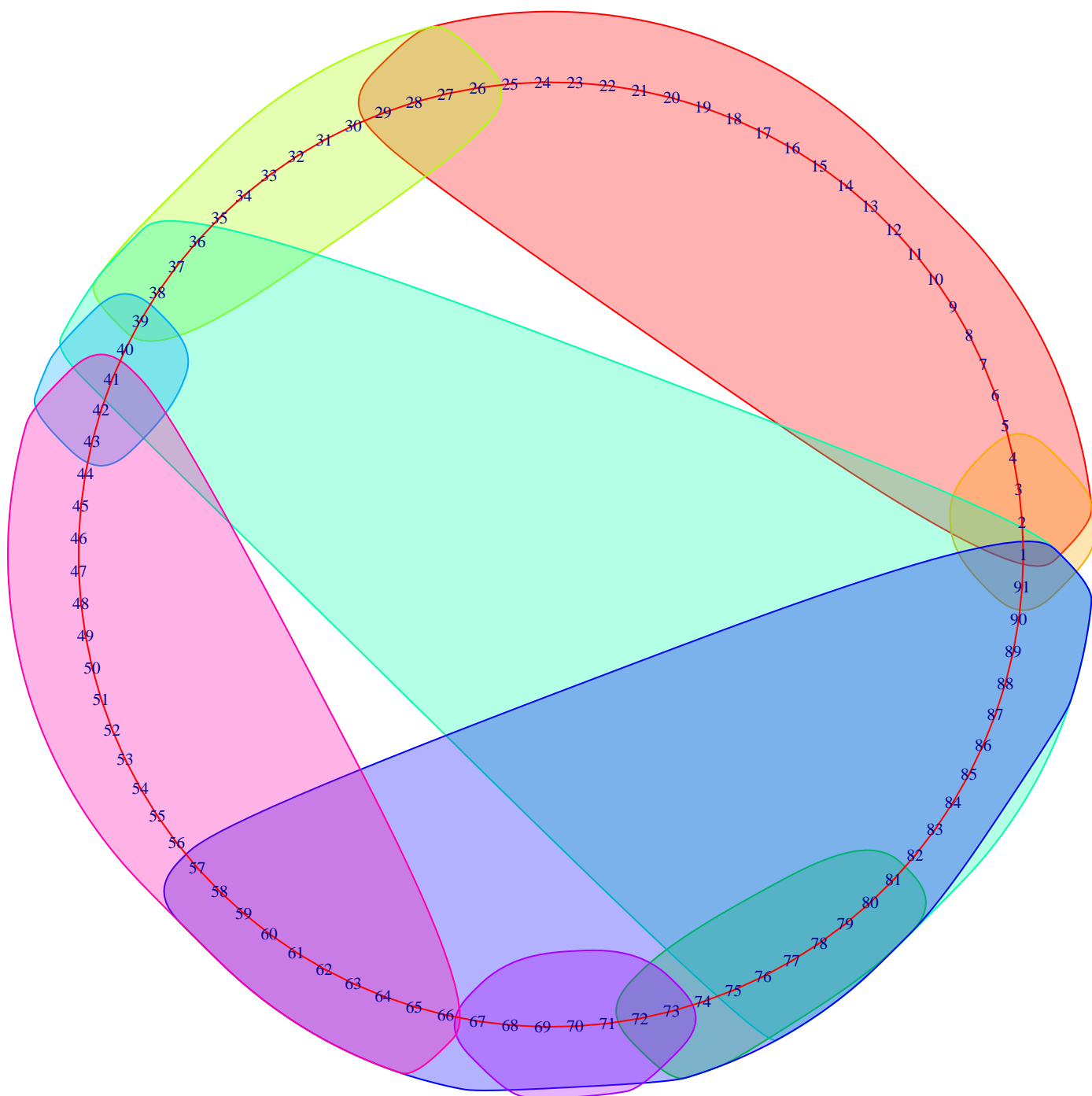

Supplement: Supplementary file 1 [file brainsci-09-00144-s001.zip › Supplementary 2/Mapper_graphs/568963_2B.pdf]

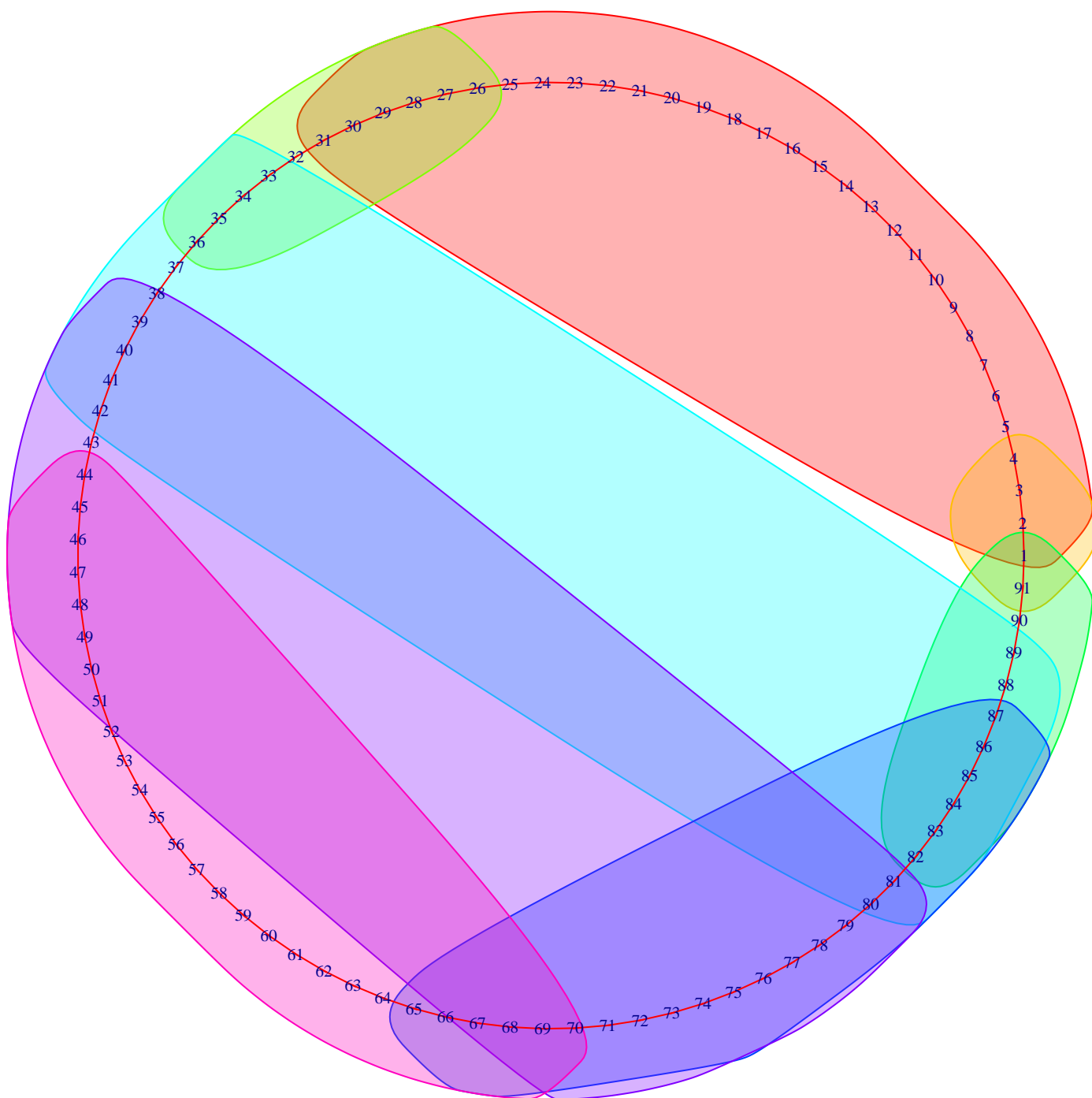

Supplement: Supplementary file 1 [file brainsci-09-00144-s001.zip › Supplementary 2/Mapper_graphs/151526_graph0B.pdf]

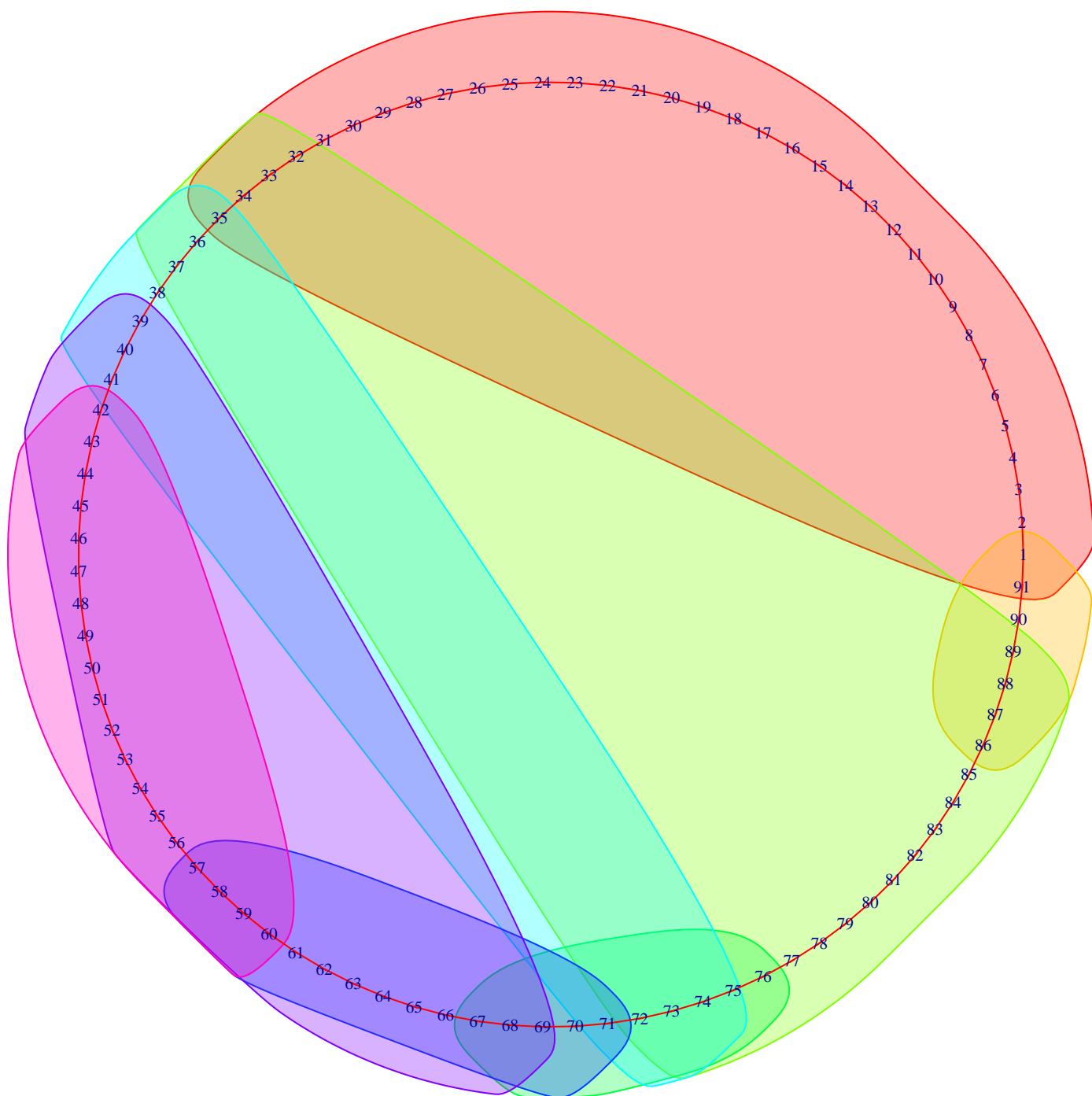

Supplement: Supplementary file 1 [file brainsci-09-00144-s001.zip › Supplementary 2/Mapper_graphs/223929_0B.pdf]

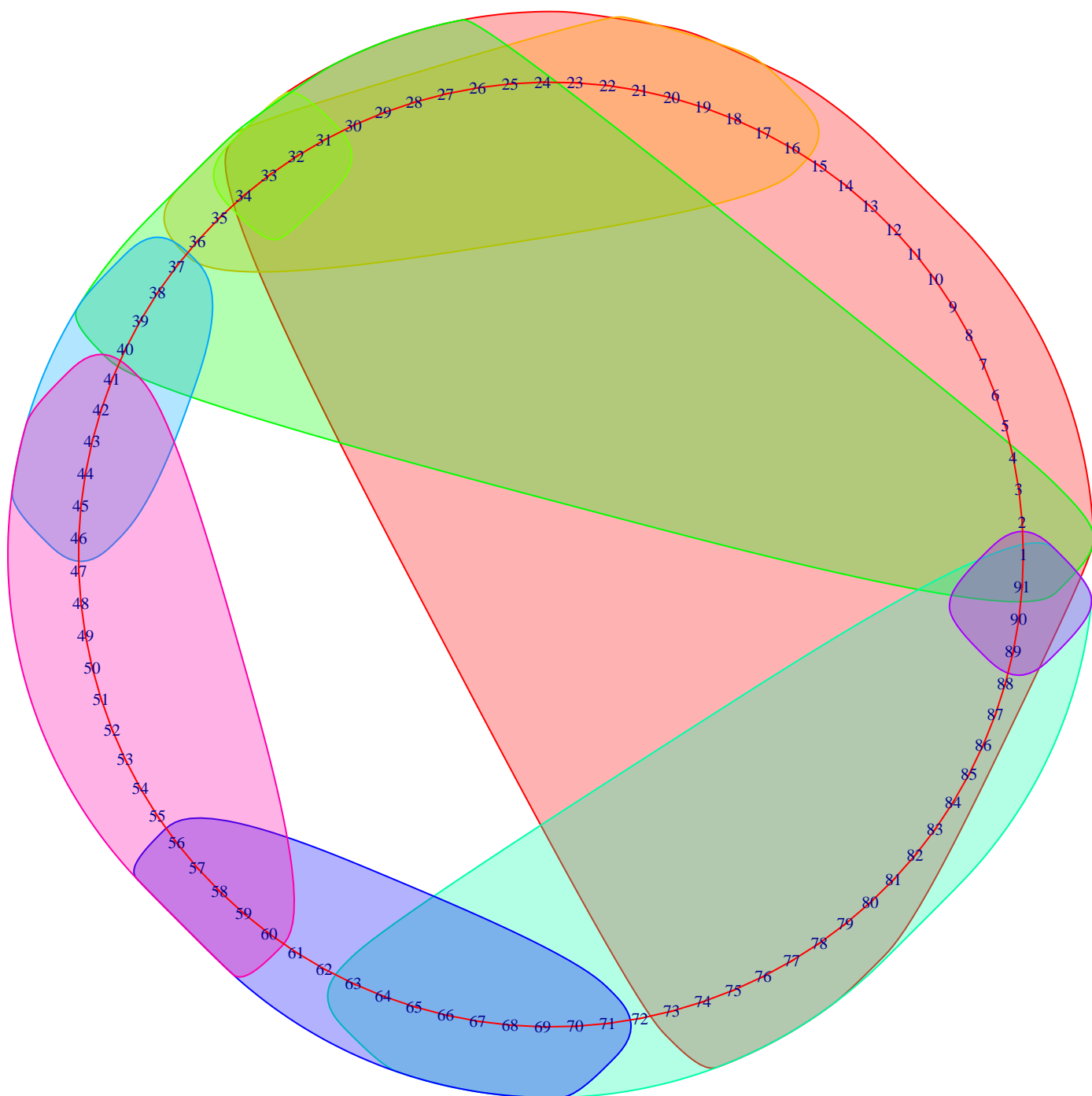

Supplement: Supplementary file 1 [file brainsci-09-00144-s001.zip › Supplementary 2/Mapper_graphs/162026_graph0B.pdf]

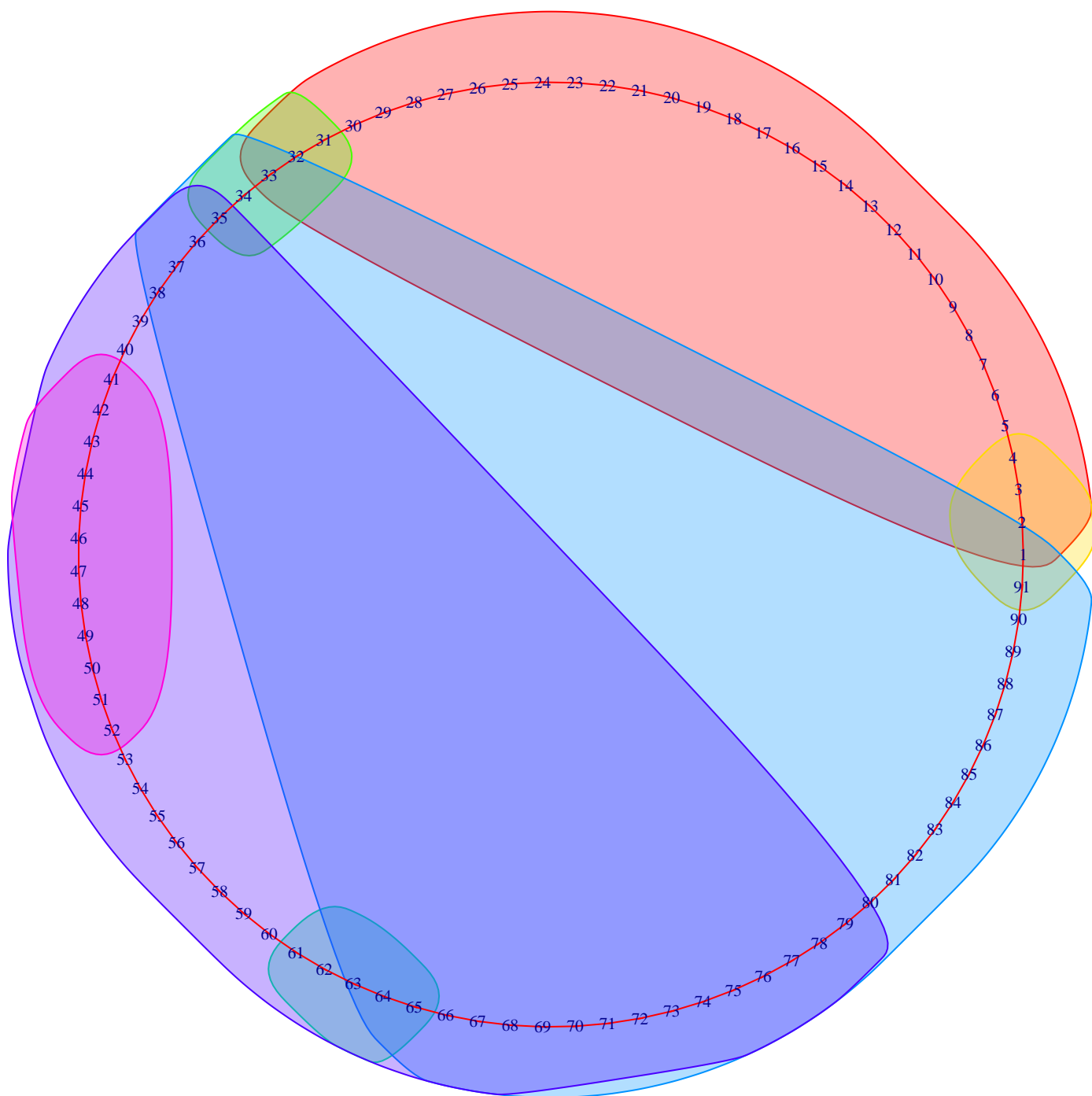

Supplement: Supplementary file 1 [file brainsci-09-00144-s001.zip › Supplementary 2/Mapper_graphs/100816_0B.pdf]
